# MTFR2-dependent mitochondrial fission promotes HCC progression

**DOI:** 10.1186/s12967-023-04845-6

**Published:** 2024-01-18

**Authors:** La Zhang, Xiuzhen Zhang, Haichuan Liu, Changhong Yang, Jiyao Yu, Wei Zhao, Jiao Guo, Baoyong Zhou, Ning Jiang

**Affiliations:** 1 Department of Hepatobiliary Surgery, The First Affiliated Hospital of Chongqing Medical University, Chongqing, People’s Republic of China; 2 School of Basic Medical Science, Chongqing Medical University, Chongqing, People’s Republic of China; 3 Department of Bioinformatics, Chongqing Medical University, Chongqing, People’s Republic of China; 4 The Second Clinical College of Chongqing Medical University, Chongqing, People’s Republic of China; 5 Department of Pathology, School of Basic Medical Science, Chongqing Medical University, Chongqing, People’s Republic of China; 6 Molecular Medicine Diagnostic and Testing Center, Chongqing Medical University, Chongqing, China; 7 Department of Pathology, The First Affiliated Hospital of Chongqing Medical University, Chongqing, China

**Keywords:** Hepatocellular carcinoma, Mitochondrial dynamics, Fission, Prognostic model, MTFR2

## Abstract

**Background:**

The role of mitochondrial dynamics, encompassing fission, fusion, and mitophagy, in cancer progression has been extensively studied. However, the specific impact of mitochondrial dynamics on hepatocellular carcinoma (HCC) is still under investigation.

**Methods:**

In this study, mitochondrial dynamic genes were obtained from the MitoCarta 3.0 database, and gene expression data were collected from The Cancer Genome Atlas (TCGA) database. Based on the expression of these dynamic genes and differentially expressed genes (DEGs), patients were stratified into two clusters. Subsequently, a prognostic model was constructed using univariate COX regression and the least absolute shrinkage and selection operator (LASSO) regression, and the prognostic signature was evaluated. We analyzed the interaction between these model genes and dynamic genes to identify hub genes and reveal mitochondrial status. Furthermore, we assessed immune infiltration, tumor mutational burden (TMB), tumor stemness indices (TSI), and the response to immune checkpoint block (ICB) therapy using the TIDE algorithm and risk scores. Additionally, transmission electron microscopy (TEM), hematoxylin-eosin (H&E) staining, immunohistochemistry (IHC), western blotting (WB), and immunofluorescence (IF) were conducted to afford detailed visualization of the morphology of the mitochondria and the expression patterns of fission-associated proteins.

**Results:**

Patients in Cluster 2 exhibited heightened mitochondrial fission and had a worse prognosis. The up-regulated dynamic genes in Cluster 2 were identified as fission genes. GO/KEGG analyses reconfirmed the connection of Cluster 2 to augmented mitochondrial fission activities. Subsequently, a ten-gene prognostic signature based on the differentially expressed genes between the two clusters was generated, with all ten genes being up-regulated in the high-risk group. Moreover, the potential links between these ten signature genes and mitochondrial dynamics were explored, suggesting their involvement in mediating mitochondrial fission through interaction with MTFR2. Further investigation revealed that the high-risk group had an unfavorable prognosis, with a higher mutation frequency of TP53, increased immune checkpoint expression, a higher TIS score, and a lower TIDE score. The mitochondrial imbalance characterized by increased fission and upregulated MTFR2 and DNM1L expression was substantiated in both HCC specimens and cell lines.

**Conclusions:**

In conclusion, we developed a novel MTFR2-related prognostic signature comprising ten mitochondrial dynamics genes. These genes play crucial roles in mitochondrial fission and have the potential to serve as important predictors and therapeutic targets for HCC.

**Supplementary Information:**

The online version contains supplementary material available at 10.1186/s12967-023-04845-6.

## Introduction

Liver cancer, specifically hepatocellular carcinoma (HCC), stands as the second leading etiology of cancer-related mortality worldwide and is characterized by a rising prevalence and constrained therapeutic modalities [1]. The development of HCC is often associated with underlying liver cirrhosis resulting from factors such as viral hepatitis, alcohol consumption, and nonalcoholic steatohepatitis. The presence of this background complicates tumor progression, hampers early detection, and limits therapeutic choices [2]. Consequently, there is an urgent need to identify potential biomarkers that can effectively predict the diagnostic and prognostic information of patients with HCC.

Mitochondria, which are highly dynamic organelles with a double-membrane structure, exerts instrumental influence on fundamental biological functions, including energy production, metabolism modulation, calcium ions storage, and cellular proliferation regulation. Abnormalities in mitochondria have been identified as early and widespread features of liver cirrhosis and fibrosis, highlighting their fundamental involvement in HCC development [3, 4]. The maintenance of mitochondrial integrity and homeostasis is crucial and relies on mitochondrial dynamics [5]. Mitochondrial dynamics, which include fusion and fission, are catalyzed by a substantial number of proteins [6]. Mitochondrial fission, inducing the selective elimination of dysfunctional mitochondria, is mainly mediated by the recruitment of dynamin-related protein 1 (DRP1, encoded by DNM1L) to the outer mitochondrial membrane (OMM) [7]. DRP1 self-assembles to form a ring-like structure around the mitochondria and promotes GTP hydrolysis to mediate membrane constriction. Fusion begins with mitofusins 1/2 (MFN1/2)-mediated OMM fusion, and subsequent inner mitochondrial membrane (IMM) fusion is carried out by the interaction between left-optic atrophy 1 (L-OPA1) and cardiolipin [8]. Fusion restores impaired mitochondrial function and prevents excessive clearance of mitochondria [9]. As the critical role of mitochondria dynamics in maintaining cellular homeostasis, defects in mitochondrial dynamics are related to many pathological conditions, including neuropathies, neurodegenerative disorders, atherosclerosis, metabolic diseases, and cancers [10]. It was most demonstrated that increased fission links to tumorigenesis, metastatic progression and immune surveillance [11–16]. Mitochondrial fission caused by the overexpression of DNM1L is demonstrated to be positively regulated by the activation of oncogenic mutations and correlated with accelerated tumor progression, resistance to chemotherapy, and metabolic deregulation [17].

**Fig. 1:**
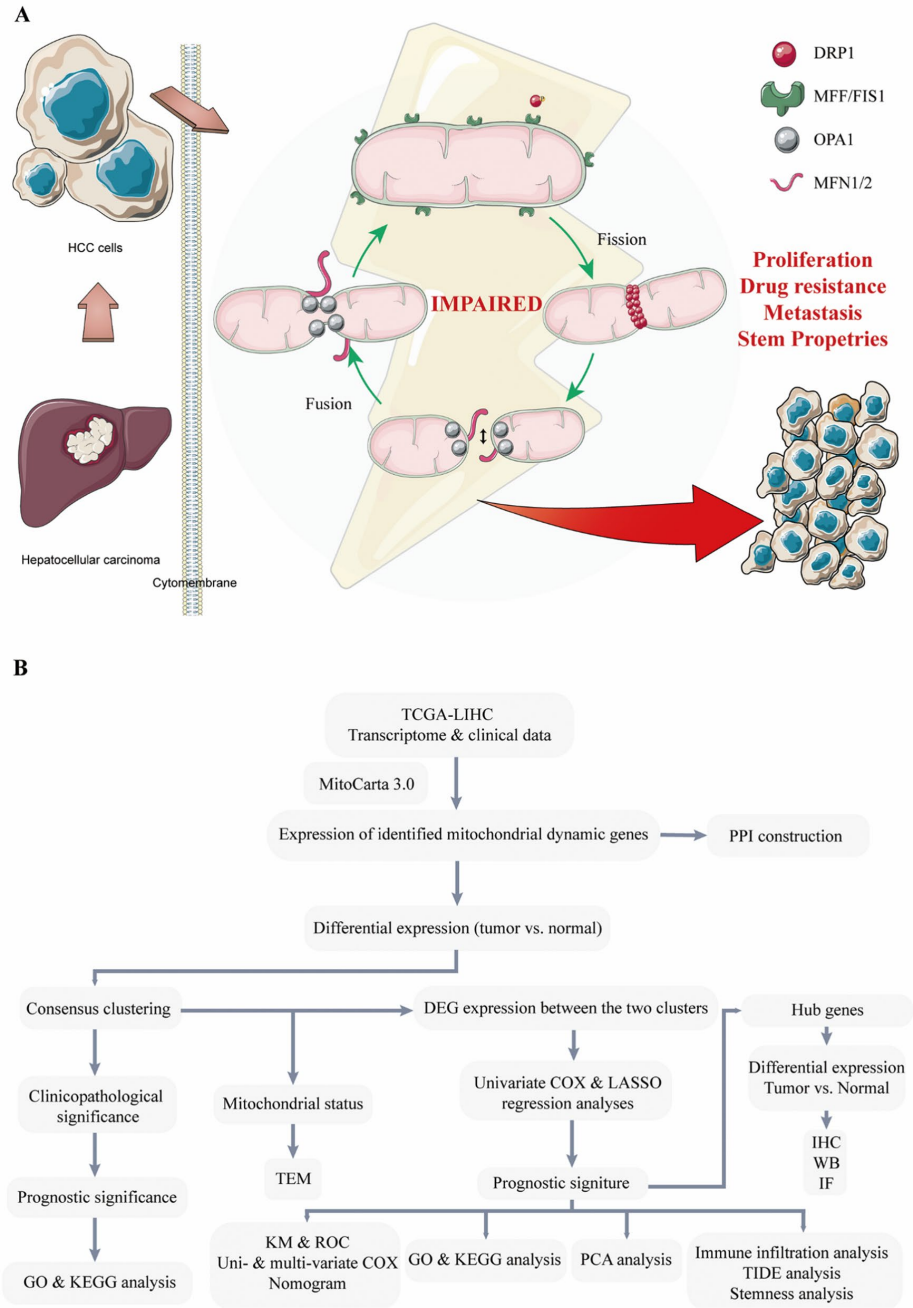
**A** The mitochondrial dynamic paradigm. Mitochondria undergo dynamic changes in shape by the two opposite processes of fission and fusion. Mitochondrial fusion of the outer membrane is mediated by MFNs. Fusion of the inner mitochondrial membrane is regulated by OPA1. Mitochondrial fission requires DRP1 phosphorylation and recruitment. In HCC, impaired mitochondrial dynamic equilibrium induces tumor proliferation, drug resistance, metastasis, and stem properties. **B** Flow chart of construction and validation of a prognostic signature for LIHC. *MFNs* mitofusins, *OPA1* optic atrophy 1, *DRP1* dynamin-related protein 1, *HCC* hepatocellular carcinoma. *LIHC* liver hepatocellular carcinoma

In HCC, Bao et al. reported that overexpressed DRP1 promoted tumor-associated macrophage (TAM) infiltration and tumor aggression through mitochondrial fission-induced mitochondrial DNA (mtDNA) stress [18]. Additionally, DRP1-driven mitochondrial fission modulated T cell growth and migration, underscoring its potential implications for immunotherapeutic strategies [19]. Mitochondrial fission regulator 2 (MTFR2, also termed DUFD1 or FAM54A), regulating fission in a DRP1-dependent manner, is prognostically associated with oncological adversity. Besides, according to Fan et al., upregulation of DRP1 impaired the mitochondria and inhibited pituitary adenoma growth by inducing apoptosis and autophagy. The cumulative evidence strongly indicated that mitochondrial dynamics critically influence tumor progression and immune infiltration. In HCC, an imbalance in mitochondrial dynamics is implicated in tumor proliferation, drug resistance, metastasis, and the acquisition of stem-like properties (Fig. 1A). However, the regulation mechanism of mitochondrial dynamics on HCC remains largely unknown.

In this study, the flow diagram is shown in Fig. 1B. Dynamic mitochondrial genes were collected from MitoCarta 3.0, and differential expression between normal and HCC samples was examined using RNA sequencing data from The Cancer Genome Atlas (TCGA) LIHC dataset. The top differentially expressed genes were fission genes, and their interactions were assessed. Then, consensus clustering based on the expression of dynamic genes was employed to stratify patients into two distinct cohorts, which held significant discrepancies in clinical outcomes, and a prognostic signature was constructed according to the differentially expressed genes of the two clusters. Subsequently, GO/KEGG analyses were applied to explore the links between signature genes and mitochondrial dynamics. In addition, immune infiltration, tumor mutational burden (TMB), tumor stemness indices (TSI), and the response to immune checkpoint blockade (ICB) therapy based on the TIDE algorithm and risk scores were evaluated. Furthermore, the expression of signature genes was verified in clinical HCC tissues and cell lines. Herein, comprehensive analyses of mitochondrial dynamics will facilitate prognostic prediction and insight into precision immunotherapy for HCC.

## Materials and methods

### Ethics statement

The study protocol was approved by the Institutional Research Ethics Committee of Chongqing Medical University. Liver tissues from HCC patients undergoing hepatic resection were obtained at the first affiliated of Chongqing Medical University and written informed consent was obtained from all patients.

### Datasets acquisition

The RNA-sequencing data (50 normal liver tissues and 369 HCC tissues) and corresponding clinicopathology data (shown in Table 1) for the TCGA-LIHC cohort were acquired from The Cancer Genome Atlas (TCGA) database (https://portal.gdc.cancer.gov/ ).

**Table 1:** Clinicopathological parameters of patients with HCC

Feature	N (377)	%
Age (years)		
≤ 65	235	62.3
＞65	141	37.4
Unknown	1	0.3
Gender		
Male	255	67.6
Female	122	32.4
Histologic grade		
G1	55	14.6
G2	180	47.8
G3	124	32.9
G4	13	3.4
Unknown	5	1.3
TNM stage		
I	175	46.4
II	87	23.1
III	86	22.8
IV	5	1.3
Unknown	24	6.4
T		
1	185	49.1
2	95	25.2
3	81	21.5
4	13	33.4
X	1	0.3
Unknown	2	0.5
N		
0	316	83.8
1	2	0.5
X	59	15.6
M		
0	272	72.1
1	4	1.1
X	101	26.8

### Collection of differentially expressed mitochondrial dynamic genes

Twenty-three mitochondria dynamics genes were identified from MitoCarta 3.0 [20], presented in Additional file 2: Table S1. The “limma” R package was conducted to figure out differentially expressed genes (DEGs) of mitochondrial dynamic genes between normal and tumor tissues with the threshold of *P* < 0.05 [21]. The co-expression network of the DEGs was explored by the “igraph” R package [22]. Using the STRING (https://string-db.org/) database, the protein-protein interaction (PPI) network of proteins coded by the mitochondrial dynamic DEGs was constructed and visualized.

### Consensus clustering analysis

According to the RNA sequences of the 21 mitochondrial dynamic genes in HCC patient tissues, consensus clustering was implemented to cluster the HCC patients. The patients were clustered into different molecular sub-groups by performing the consensus clustering (K-means) algorithm based on the R package “ConsensusClusterPlus” [23]. The algorithm was repeated 50 times to obtain reliable mitochondrial dynamic subtypes. The consensus cumulative distribution function (CDF), delta area, and cluster-consensus (CLC) were conducted to identify the optimum number of sub-clusters. The HCC patients in the TCGA cohort were divided into two clusters according to the consensus of mitochondrial dynamic genes. And the overall survival (OS) was compared between the two sub-clusters via Kaplan–Meier (KM) analysis (“survival” and “survminer” R packages) [24, 25].

### Differences in clinicopathology features and pathways of the mitochondrial dynamic sub-clusters

The “limma”, “pheatmap” and “ggpubr” R packages were performed to analyze and present the differences in the clinical and pathological variables between the two sub-clusters. R packages “survival” and “survminer” were conducted to analyze the prognosis of patients with different molecular sub-clusters. The DEGs of the two sub-clusters were point out by “limma” R package [|log2FC| > 1 and false discovery rate (FDR) < 0.001]. Gene ontology (GO) enrichment and Kyoto Encyclopaedia of Genes and Genomes (KEGG) pathway analyses of DEGs of the two sub-clusters, were performed to explore the potential biological processes and pathways using the “clusterProfiler” R package [26].

### Generation of the mitochondrial dynamic prognostic signature

Considering that quantitation of mitochondrial dynamic subtypes would contribute to clinical application, the expression of the DEGs of the two sub-clusters with the threshold of |log2 fold change| (|log2FC|) > 1 and false discovery rate (FDR) < 0.05 was analyzed to evaluate the correlations with the survival outcomes by using the univariate Cox regression (R package “survival”). Then the prognostic signature was conducted by performing the least absolute shrinkage and selection operator (LASSO) regression analysis (R package “glmnet”) [27]. The formula is as follows: risk score = Σ (βi × Expi) (β: coefficient, Exp: RNA expression level). In the light of the risk score, the median value of the risk score for HCC patients was identified, and patients were classified into low- and high-risk groups.

### Predictive power of the prognostic model for HCC

The receiver operating characteristic (ROC) curves were generated to evaluate the predictive performance for OS by different clinicopathological factors and the risk model using the “survivalROC” R package. We evaluated the accuracy of the prognostic model compared with the clinicopathological characteristics through the concordance index (C-index) with “pec” R package [28]. KM survival curves were plotted to describe the OS difference between the two risk sub-groups. And the KM curves of these ten genes building up the prognostic model were generated using the GEPIA database (http://gepia.cancer-pku.cn/). Principal component analysis (PCA) and t-distributed stochastic neighbor embedding (t-SNE) were conducted to explore the distribution of different groups performing the “Rtsne” R package [29]. Univariate and multivariate Cox regression analyses (“Survival” R package) were used to evaluate the predictive ability of the risk score calculated using the constructed prognostic model. A nomogram was constructed integrating the risk score and relevant clinical varieties, for predictive of 1, 3, and 5-year OS of HCC patients via “rms” R package [30].

### Mitochondrial status of the prognostic model predicted through functional enrichment analysis and the hub gene exploration

To obtain the biological functions associated with the risk signature defined by the mitochondrial dynamic cluster, DEGs were selected between the low- and high-risk groups using “limma” package (|log2FC|) > 2 and FDR < 0.05). Then, the PCA analysis was performed to evaluate potential differences between low- and high-risk groups, based on the R package “scatterplot3d” [31]. The co-expressing correlation between the 23 dynamic genes and 10 model genes was detected using Spearman’s correlation test (|R2| > 0.4 and adjusted P < 0.001) for double-checking the mitochondrial status. The network was then displayed using Cytoscape software [32]. CytoHubba, a Cytoscape plugin, was used to identify the hub gene through the 11 topological analysis methods (including Maximal Clique Centrality (MCC), Density of Maximum Neighborhood Component (DMNC), Maximum Neighborhood Component (MNC), Degree, Percolated Component (EPC), BottleNeck (BN), EcCentricity, Closeness, Radiality, Betweenness, Stress), aiming to find the potential molecular mechanisms of selected model genes. At last, the R package “clusterProfiler” was used to perform GO and KEGG enrichment analyses.

### Analyses of relation between tumor mutation burden, tumor stemness indices, and risk score and prediction of the effective response of postoperative immunotherapy

Seven algorithms (TIMER, XCELL, MCPcounter, QUANTISEQ, CIBERSORT, EPIC, and CIBERSORT-ABS) were applied to estimate the infiltration status of different types of immune cells between the two risk groups [33]. And ssGSEA was performed to explore the different infiltration degrees of immune checkpoints in the two risk groups using the R package “GSVA” [34]. Tumor stemness indices (TSI) were calculated based on the mRNA expression by machine learning from previous studies [35]. The stem cell gene set was also gained from a previous study [36]. In addition, the TIDE algorithm was performed to assess immune checkpoint blockade therapy (ICB therapy) responses and evaluate the ability to serve as a neoantigen (http://tide.dfci.harvard.edu) [37]. The TIDE algorithm gathers two primary mechanisms of immune evasions: T cell dysfunction and T cell exclusion. A higher TIDE score suggests a more substantial potential for tumor immune escape and worse immunotherapy response [37]. The somatic mutation data (MuTect2 Variant Aggregation and Masking) was downloaded from the TCGA-LIHC dataset, and the frequency of gene mutation in specific patients was calculated by the R package “maftools” [38]. Significantly mutated genes (SMGs) were also identified by the “maftools” package. And tumor mutation burden (TMB) was defined as the total number of somatic mutations per million bases and analyzed by the “maftools” package, too. The KM analysis was conducted to reveal different OS based on the different TMB groups.

### Transmission electron microscopy (TEM)

The HCC cancer tissue and paired para-cancerous tissue were collected and prefixed with a 3% glutaraldehyde immediately after surgical resection. Then the tissues were postfixed in 1% osmium tetroxide, dehydrated in series acetone, infiltrated in Epox 812 for a longer, and embeded. Then the methylene blue was used to stain the semithin sections, which were then cut to the ultrathin sections with a diamond knife. After being stained with uranyl acetate and lead citrate, the ultrathin sections were examined with JEM-1400-FLASH Transmission Electron Microscope.

### Histological analysis

Ten paired cancerous and para-cancerous tissues were collected and prepared for generating paraffin-embedded sections. For the hematoxylin-eosin (H&E) processing, the de-paraffinized sections were stained with hematoxylin for 5 min, washed and followed by being stained with eosin for 1 min. The paraffin sections were incubated with the MTFR2 primary antibody (1:200; Merck; HPA29794) and the DNM1L primary antibody (1:200; Sangon biotech; D222247) at 4 ℃ overnight and with secondary antibodies at 37 ℃ for 30 min. After washing, the samples were counterstained with hematoxylin, dehydrated through an ethanol-xylene series, and covered with neutral balsam (Biosharp; BL704A).

### Western blotting

Human HCC cell lines Huh-7, SK-Hep-1, and the immortal human hepatocyte cell line LO2 were obtained from the Institute of Infectious Diseases of Chongqing Medical University. Protein lysates were prepared by lysing the cells and run on sodium dodecyl sulfate-polyacrylamide gels for electrophoresis (Bio-Rad). Separated proteins were then transferred to the polyvinylidene fluoride (PVDF) membranes. The membranes were blocked with 5% skim milk and incubated with the primary antibody in the blocking buffer (overnight at 4℃) followed by horseradish-peroxidase-conjugated secondary antibodies (Proteintech) for 1 h at room temperature. The blots were developed by performing the enhanced chemiluminescence detection reagents on the membranes and the signals were detected by the ECL blotting analysis system (Bio-OI, Guangzhou, China). The band intensity was quantified by ImageJ 2.0.0 (ImageJ, NIH). Beta-actin in Western blotting was used as the endogenous loading control. Anti-MTFR2 polyclonal antibody (1:500; Sigma; HPA29794), anti-DNM1L polyclonal antibody (1:1000; Sangon biotech; D222247) and anti-β-actin monoclonal antibody (1:1000; Proteintech; 66009-1-Ig) were used.

### Immunofluorescence microscopy

For visualizing MTFR2 and DNM1L, cells were seeded on coverslips at 37℃ with 5% CO_2_ for 12 h, washed once in PBS and then fixed with 4% paraformaldehyde by incubation for 30 min. Flowing three washes in PBS, cells were permeabilized with 0.3% TritonX-100 for 20 min and blocked in 5% BSA (Boster Biotech, Wuhan, China) for 1 h. Fluorescent antibody incubation (1:100) was performed after washing twice, overnight at 4℃ in the dark. Moreover, the secondary antibodies (1:200; Proteintech; SA00013-4) were conducted for 1 h at room temperature. Subsequently, the cells were mounted with DAPI (Beyotime; Haimen, China) for nuclei staining. Cells were imaged using Leica DMi8 microscope. Anti-MTFR2 polyclonal antibody (1:100; Sigma; HPA29794), anti-DNM1L polyclonal antibody (1:100; Sangon biotech; D222247) were used.

### Statistical analysis

Statistical analysis was conducted by R Studio (version 4.0.0). Continuous variables such as gene expression levels were analyzed using the single-factor analysis method. For categorical variables, Pearson’s chi-square was used. The Kaplan-Meier method was applied to compare the OS of patients in the two groups. Univariate and multivariate Cox regression analyses were employed to evaluate the prognostic value of the risk signature model. The correlation between TMB and risk score was evaluated with Spearman rank correlation. The correlations between the risk score, expression of the single gene in the signature, and the expression of immune checkpoints were calculated with Spearman rank correlation. Two-way analysis of variance (ANOVA) was performed by using Prism 8 (GraphPad) for analyzing the quantitative western blotting data.

## Results

### The signature of mitochondrial dynamic genes in HCC

**Fig. 2:**
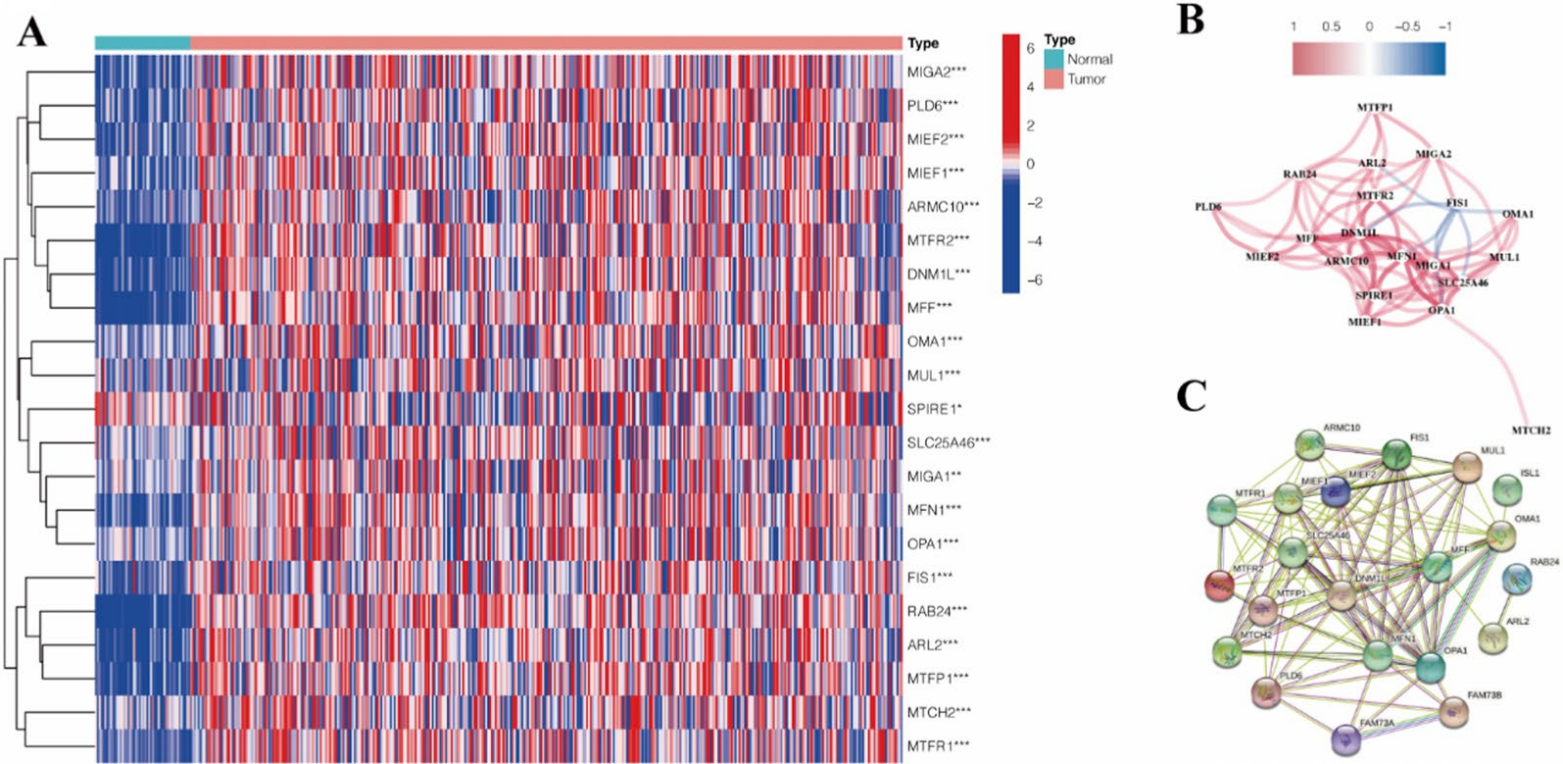
**A** The heatmap shows the expression of 21 differentially expressed mitochondrial dynamic genes. **B** The co-expression network of the 21 DEGs. **C** PPI network offers the interaction among the 21 DEGs by using the STRING database. *DEGs* differentially expressed genes, *PPI* protein-protein interaction

Among the 23 mitochondrial dynamic genes, 21 genes (DNM1L, MTFR2, ARL2, MFN1, MIGA1, MIGA2, MTCH2, OMA1, OPA1, PLD6, ARMC10, FIS1, MFF, MIEF1, MIEF2, MTFP1, MTFR1, MUL1, RAB24, SLC25A46, SPIRE1) significantly up-regulated in HCC tissues compared with the normal, and the heatmap of their expression levels displayed in Fig. 2A. The co-expression network of the DEGs shows a positive correlation between the expression level of MTFR2 and DNM1L (Fig. 2B). As well as the interaction between these proteins coded by these 21 DEGs is shown in Fig. 2C.

### Consensus clustering of mitochondrial dynamic genes identified two clusters of HCC patients with different pathological features, mitochondrial status, and clinical prognoses

**Fig. 3:**
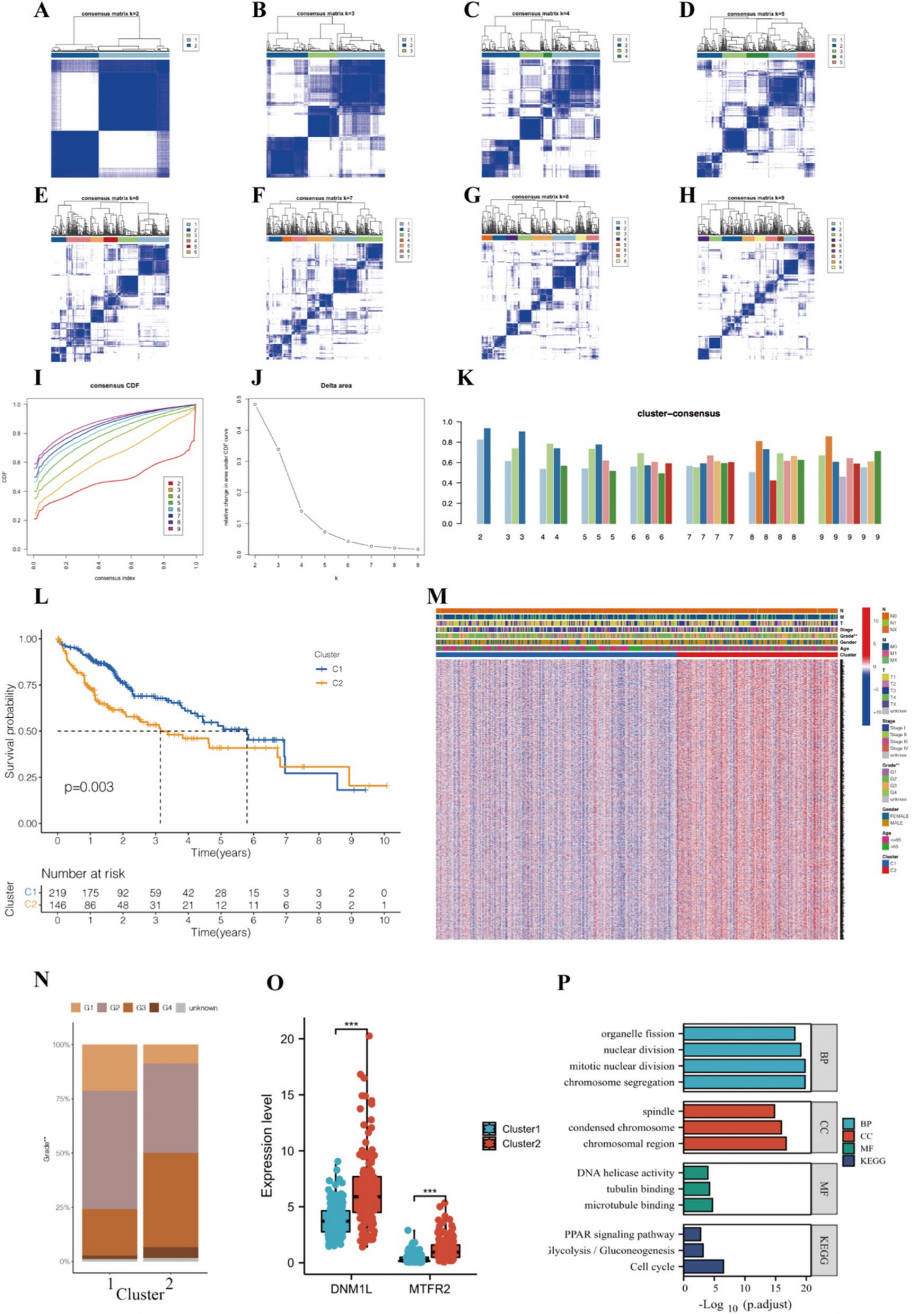
**A**–**H** Consensus matrix plots for k = 2 to 9. **I** Consensus clustering CDF for k = 2 to 9. **J** Relative change in area under CDF curve for k = 2 to 9. K = 2 was determined as the optimal clustering number. **K** Histograms of each sample when k ranges from 2 to 9. **L** The Kaplan–Meier survival analysis for the two clusters. **M** The heatmap of DEG expressions of the two clusters and clinicopathological features between the two clusters. **N** Comparison of the tumor grades between the two clusters. **O** The bar graph displays the expression differences of MTFR2 and DNM1L between the two clusters. **P** Barplot graph for GO enrichment and KEGG pathway analysis. *CDF* cumulative distribution function, *DEG* differentially expressed gene, *GO* Gene Ontology, *KEGG* Kyoto Encyclopaedia of Genes and Genomes, *BP* biological process, *CC* cellular component, *MF* molecular function. ** *P* < 0.01, *** *P* < 0.001

Based on the expression of the known dynamic genes, a “cleanest” clustering was observed when k = 2 (Fig. 3A–H). And the clusters had the highest correlation within groups and the least interference between groups when k = 2 (Fig. 3I, J). Thus, the HCC patients were classified into two sub-clusters: Cluster1 (*n* = 219) and Cluster2 (*n* = 146). The KM curves showed a significant survival advantage for Cluster1 patients (*P* = 0.003, Fig. 3L), and most DEGs were highly expressed in Cluster2, shown in the heatmap (Fig. 3M, Additional file 2: Table S2). In addition, HCC patients with more server grades were more represented in Cluster2 (*P* < 0.01), while other clinicopathological features were not significantly different between the two sub-clusters (Fig. 3M). In Cluster2, patients with pathological grade 3 account for nearly half of the total (Fig. 3N). When the threshold was changed to |log2FC| > 0.7, among the twenty-three mitochondrial dynamic genes, only DNM1L and MTFR2, which were proven to prompt mitochondrial fission, expressed significantly different between the two subgroups (Fig. 3O, Additional file 2: Table S3), suggesting morphology of mitochondria preferred to showing as fission in Cluster2. Results of GO and KEGG further revealed that Cluster2 was more likely to involve the mitochondria fission and cell cycle pathway (Fig. 3P). Previous studies demonstrated the essential role of DNM1L in mitochondrial fission, and MTFR2 played as the upstream of DNM1L, inducing mitochondrial fission [39, 40]. Based on these, it is reasonable to identify the DEGs between the two clusters as mitochondrial dynamics-related genes and speculate a strong correlation between MTFR2/DNM1L-related mitochondrial fission and the worse differentiation grade and prognosis.

### Construction of a risk signature containing ten selected mitochondria dynamics-related genes

**Fig. 4:**
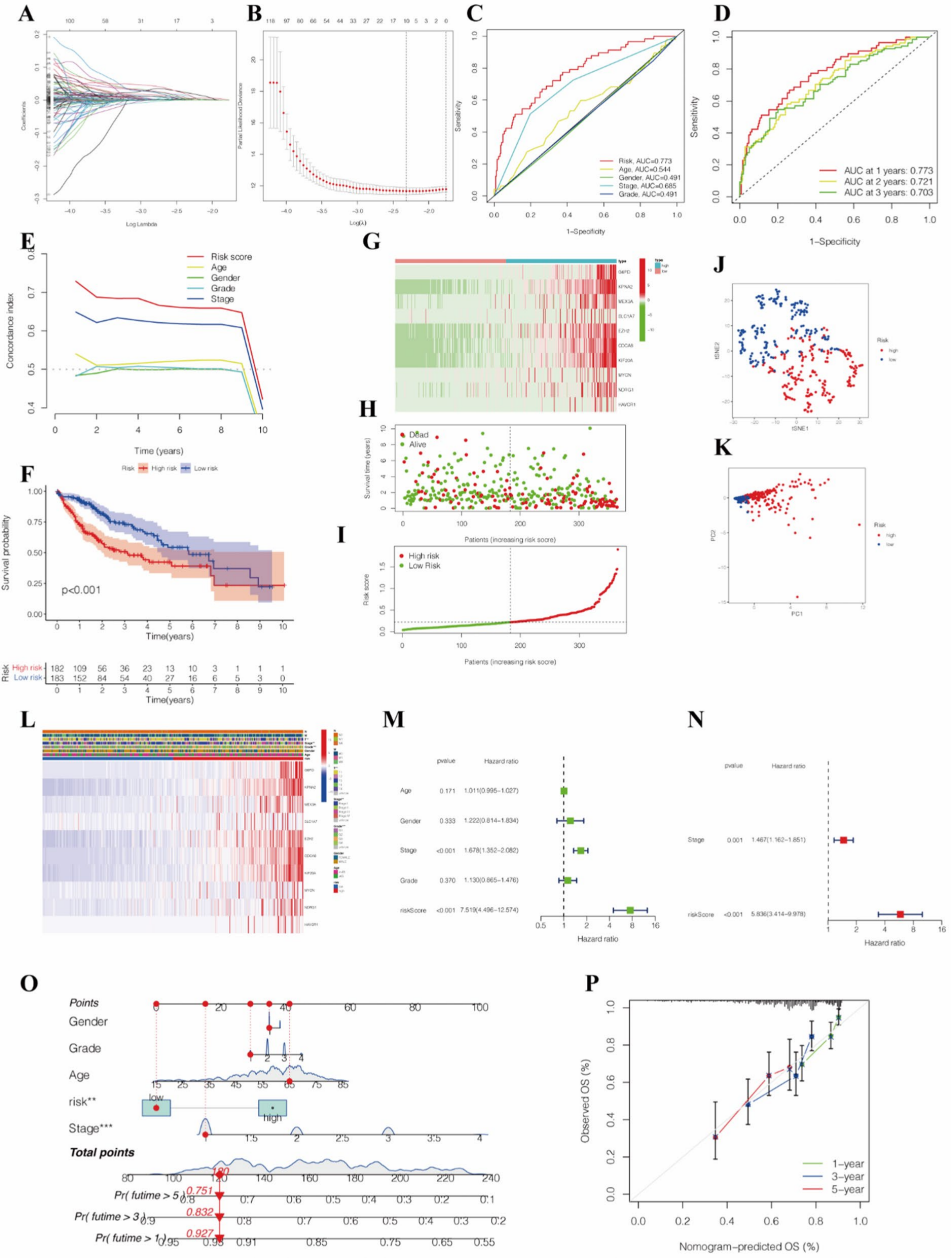
**A** LASSO regression of the mitochondrial dynamics-related genes. **B** Cross-validation for tuning the parameter selection in the LASSO regression. **C** The ROC curves of risk score and clinicopathological characteristics. **D** The ROC curves of the prognostic signature at 1, 3, and 5 years. **E** C-index of the prognostic signature compared with the clinicopathological characteristics. **F** The KM curves for the OS of patients in the high- and low-risk groups. **G** The heatmap showed the expression profiles of mitochondrial dynamics-related genes in the low- and high-risk groups. **H** The scatterplot based on the survival status of each sample. **I** The risk curve based on the risk score of each sample. **J** Dot plot for low- and high-risk groups identified by the t-SNE algorithm. **K** PCA plot. **L** The heatmap of model gene expressions and the clinicopathology factors between the two risk groups. **M** Univariate analysis. **N** Multivariate analysis. **O** A nomogram to facilitate the prognosis prediction. **P** Calibration curves of the nomogram for predicting 1, 3, 5-years OS. *LASSO regression* the least absolute shrinkage and selection operator regression, *ROC curve* receiver operating characteristic curve, *KM curve* Kaplan–Meier curve, *OS* overall survival, *t-SNE* t-distributed stochastic neighbor embedding, *PCA* principal component analysis. ** *P* < 0.01, *** *P* < 0.001

Selected from the 2143 DEGs by univariate Cox regression analysis, 775 genes met the criteria of *P* < 0.05 and were further analyzed (Additional file 2: Table S2, S4). LASSO regression analysis was conducted to build a ten-gene signature according to the optimum λ value (Fig. 4A, B). The calculating method of the risk score could be seen in Table 2.

**Table 2:** The genes involved in the signature and their coefficients

No.	Gene name	Coef
1	G6PD	0.00405315
2	KPNA2	0.00536788
3	MEX3A	0.01218156
4	SLC1A7	0.00017274
5	EZH2	0.00179814
6	CDCA8	0.02103184
7	KIF20A	0.00474807
8	MYCN	0.01449545
9	NDRG1	0.00055843
10	HAVCR1	0.00454085

### Multi-omics validation of the risk signature

The ROC curves were generated to assess the predictive sensitivity and specificity of the prognostic model. It showed that the AUC of the risk score was 0.773, followed by the AUC of age and gender. In addition, AUC was separately 0.773 for 1-year survival, 0.721 for 3-year survival, and 0.703 for 5-year survival (Fig. 4C, D). Compared with other age and stage features in the comprehensive prognosis prediction, the signature gave an advantage in the C-index (Fig. 4E). The survival status is shown in the KM curves (Fig. 4F), indicating that patients in the high-risk groups had a worse prognosis than those in the low-risk groups (The KM curves of each gene are shown in Additional file 1: Figure S1). The survival status and risk score curves for the HCC patients showed that risk score was proportional to the number of deaths in HCC patients (Fig. 4F–I). Combined with PCA and t-SNE projections (Fig. 4J, K), the risk model had a reliable clustering ability for the risk score in the two groups. The heatmap showed that the expressions of model genes were up-regulated in the high-risk group (Fig. 4L). The risk score was indicated as an independent factor capable of predicting poor survival by the univariate Cox regression analysis. And the multivariate analysis revealed the risk score as a prognostic factor after adjusting for other confounding factors (Fig. 4M, N). A nomogram was built to facilitate the prognosis prediction (Fig. 4O), whose high sensitivity and accuracy were demonstrated by the calibration curves (Fig. 4P).

### Evaluation of the mitochondrial status through functional enrichment analysis

**Fig. 5:**
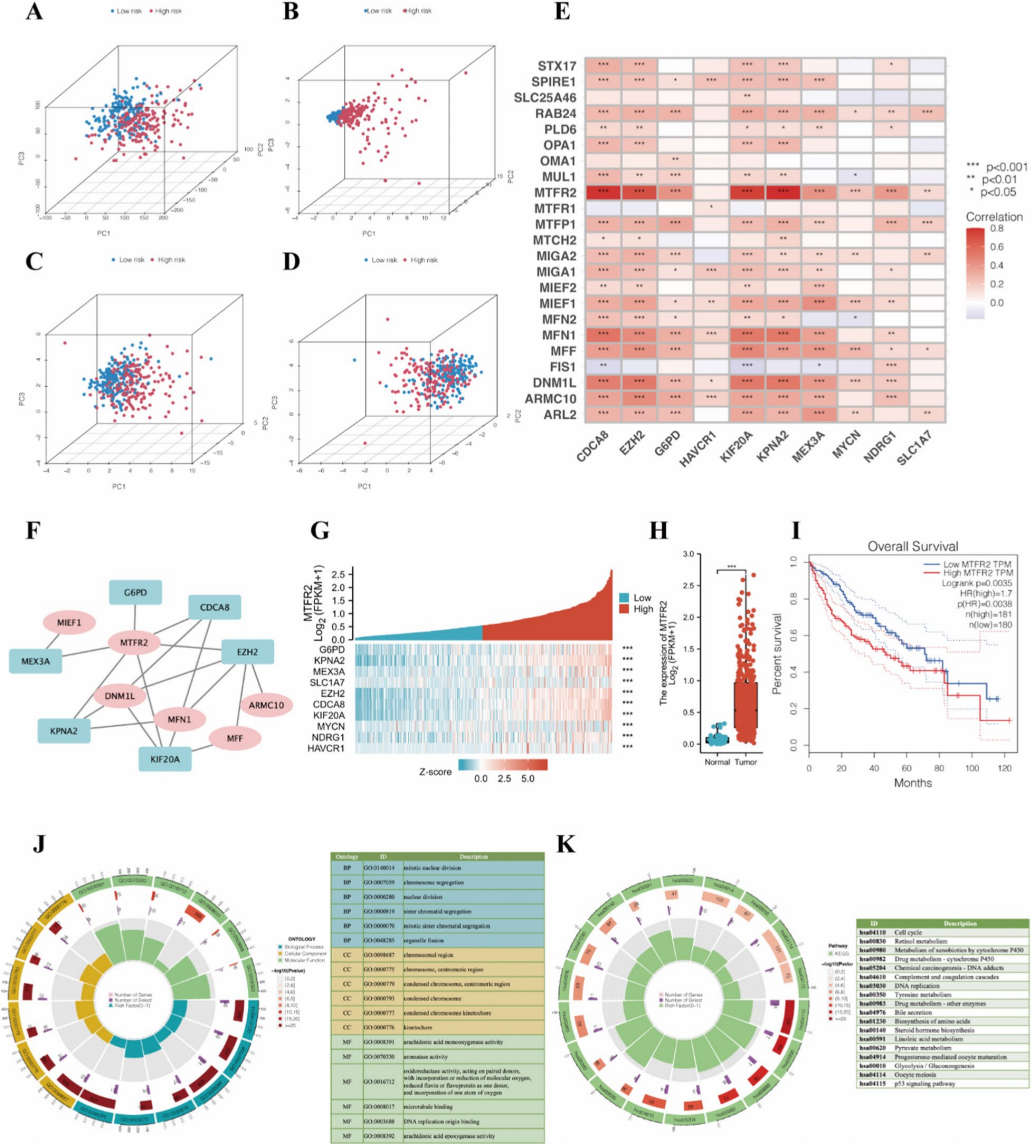
**A**–**D** Principal component analysis between low- and high-risk groups based on the expression of all genes, ten model genes, mitochondrial fission genes, and mitochondrial fusion genes. **E** Correlations between mitochondrial dynamics genes and ten model genes. **F** The network between mitochondrial dynamic genes and ten signature genes. **G** Heatmap for the co-expression of the ten model genes with MTFR2. **H** The differential expressions of MTFR2 in normal and HCC tissues. **I** The KM curves for the expression of MTFR2. **J** GO analysis of ten mitochondrial dynamics-related genes. **K** KEGG analysis of ten mitochondrial dynamics-related genes. *KM curves* Kaplan–Meier curves, GO enrichment and KEGG pathway analysis. *CDF* cumulative distribution function; *DEG* differentially expressed gene, *GO* Gene Ontology; *KEGG* Kyoto Encyclopaedia of Genes and Genomes, *BP* biological process, *CC* cellular component, *MF* molecular function. * *P* < 0.05, ** *P* < 0.01, *** *P* < 0.001

The results of PCA showed no significant difference between the low- and high-risk groups in the expression of all genes (Fig. 5A). However, there was a significant difference in the partitions made by the ten mitochondrial dynamics genes used in the prognostic signature (Fig. 5B). Moreover, the results of the patient compartmentalization according to the ten-gene model were highly consistent with those of compartmentalization based on the mitochondrial fission genes, and highly opposite according to the mitochondrial fusion genes (Fig. 5C, D). Indeed, the correlation of the expression level between the mitochondrial dynamic genes and the model genes was visualized in Fig. 5E, and the correlation network was then visualized (Fig. 5F). MTFR2 is considered the fission gene, powerfully and positively co-expressed with the risk signature genes, and was identified as the hub gene after screening with the 11 types of topology analyses (Additional file 2: Table S5). In addition, the significant co-expression of the 10 model genes with MTFR2 is shown in the heatmap (Fig. 5G).

MTFR2 was overexpressed in HCC tissue (Fig. 5H), and the patient with a high expression level of MTFR2 had a shorter OS (Fig. 5I). GO, and KEGG analyses were performed for the ten mitochondrial dynamics-related genes to explore potential biological processes associated with HCC. GO analysis detected that these ten genes were primarily associated with mitochondrial fission (Fig. 5J). The results of KEGG analysis showed that these genes were related principally to the cell cycle pathway (Fig. 5K). Therefore, we speculated that the ten risk genes promote HCC progression and take a toll on the prognosis by mediating mitochondrial fission via regulating MTFR2.

### Evaluation of immune infiltration, TMB, TSI, and immunotherapy response

**Fig. 6:**
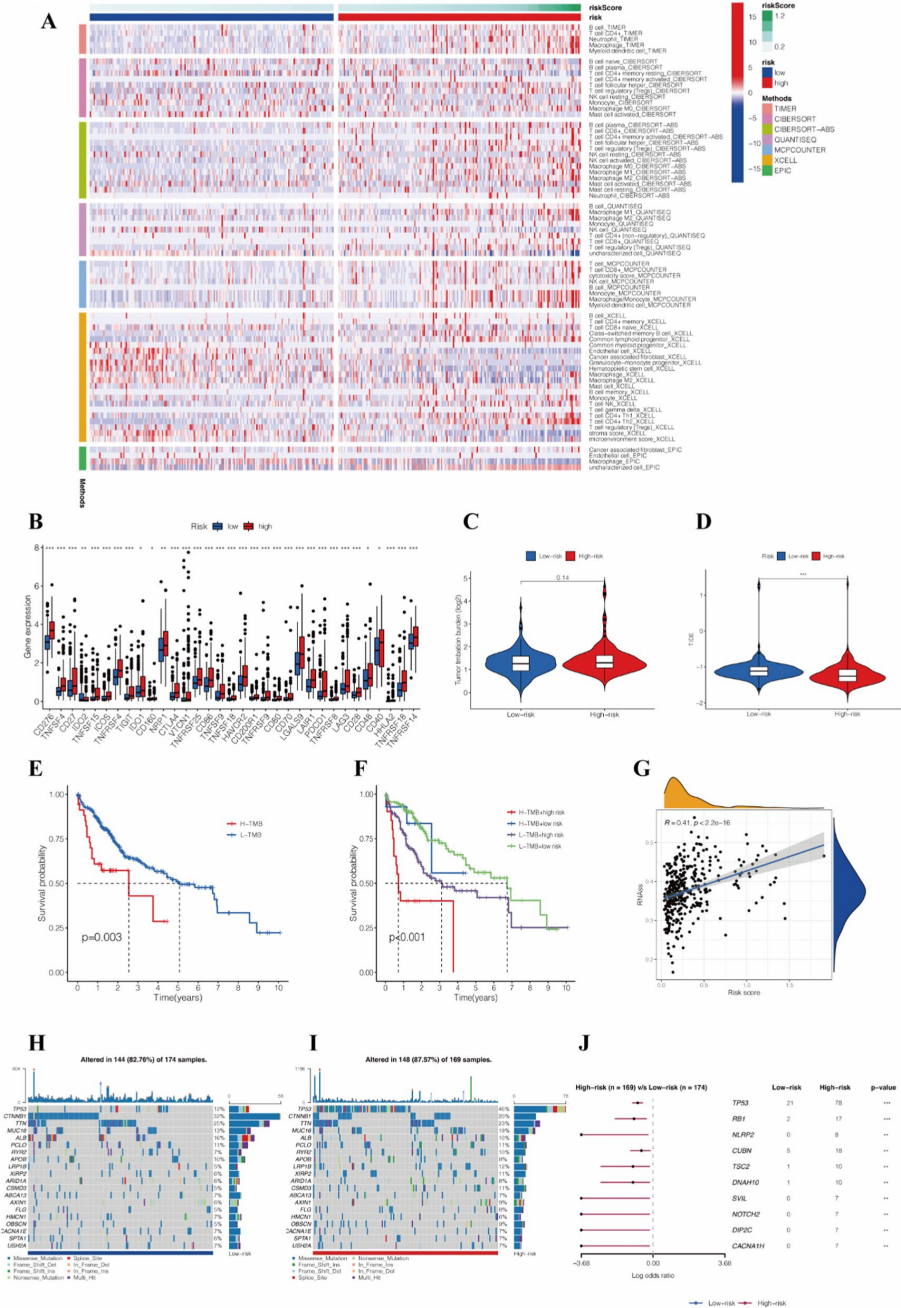
**A** The heatmap of the correlation between risk score and tumor-infiltrating immune cells. **B** Differences in the expression of the immune checkpoints between the two risk groups. **C** Differences in TMB of HCC patients in the two risk groups. **D** Differences in TIDE score of HCC patients in the two risk groups. **E** The KM curves for the patients with high- and low-TMB. **F** The KM curves for patients with high TMB + high-risk score, high TMB + low-risk score, low TMB + high-risk score, and low TMB + low-risk score. **G** Differences in TSI of HCC patients in the two risk groups. **H** Visualization of gene mutations in low-risk groups. **I** Visualization of gene mutations in high-risk groups. **J** The forest plot for the significantly different mutations between the two risk groups. *TMB* tumor mutational burden, *KM curves* Kaplan–Meier curves, *TSI* tumor stemness indices, *HCC* hepatocellular carcinoma. * *P* < 0.05, ** *P* < 0.01, *** *P* < 0.001

Since the intrinsic and intimate connection among HCC progression, mitochondrial dynamics and immune infiltration^14^, the heatmap of immune cells based on seven algorithms is shown in Fig. 6A, indicating that risk scores are correlated with immune cell infiltration in HCC. Moreover, the immune checkpoints exhibited significantly higher expression in the high-risk group (Fig. 6B). After screening for specific checkpoints, patients in the high-risk group displayed superior expression of CD274 (programmed death-ligand 1, PD-L1), and cytotoxic T-lymphocyte-associated protein 4 (CTLA4). We observed similar TMB in two risk groups (Fig. 6C), while TP53 was the most frequently altered gene in all patients, and patients in the high-risk group harbored high TP53 mutation, followed by RB1 (Fig. 6H–J). In addition, Fig. 6E showed high TMB was associated with a worse prognosis, and Fig. 6F shows the combination of risk score and TMB; patients were classified into four groups with markedly different prognoses (*P* < 0.001). The TIDE score was significantly lower in the high-risk group than in the low-risk group (Fig. 6D), suggesting that patients in the high-risk group correlated with a better response to ICB therapy. Next, the cancer stemness index was calculated, which revealed stemness index was positively associated with the risk score (Fig. 6G).

### Validation of the mitochondrial status and protein expression

**Fig. 7:**
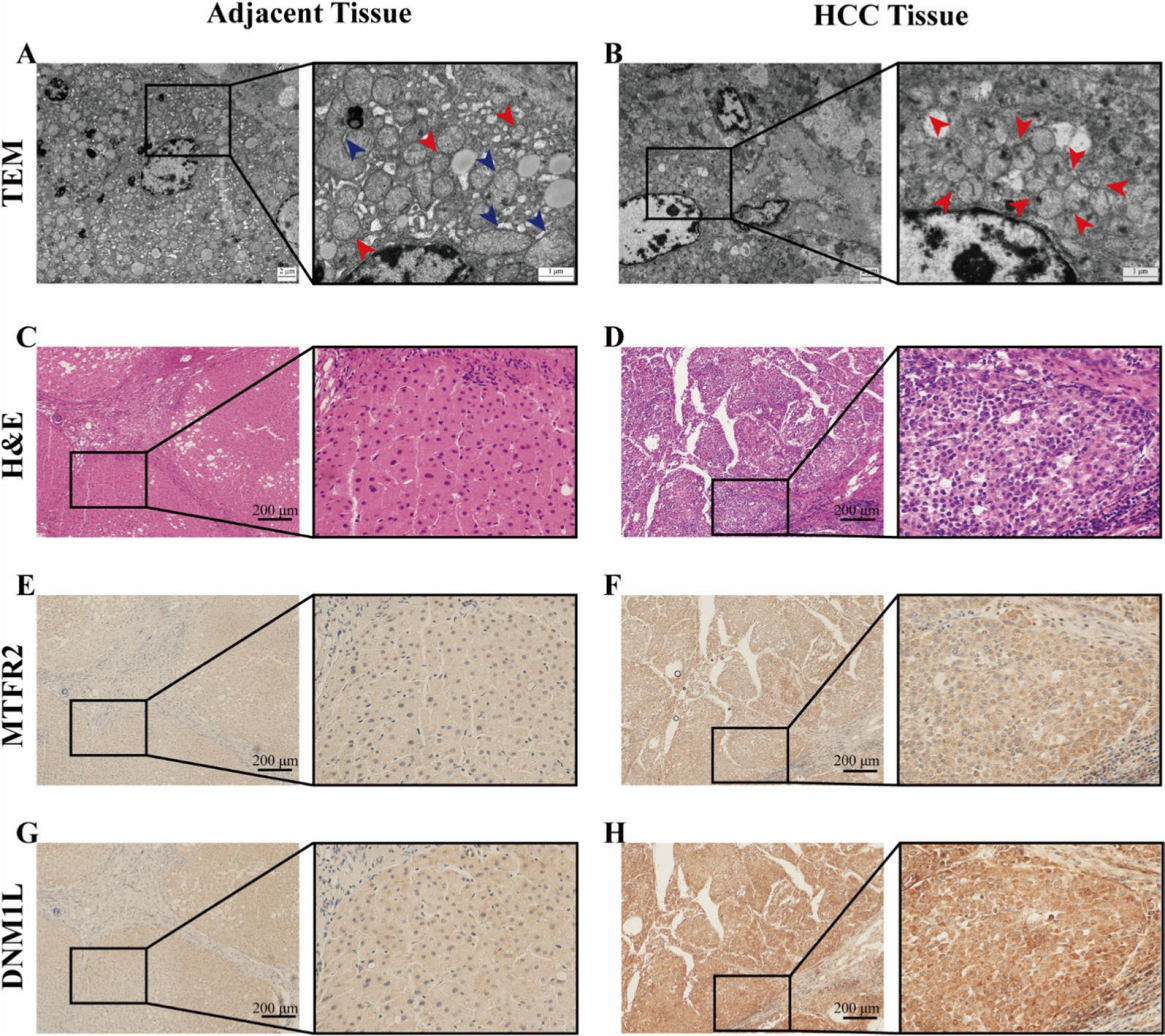
**A**, **B** Images of TEM upon cancerous and para-cancerous tissues from one HCC patient. Scale bars, 2 μm and 1 μm. **C**–**H** The representative images of H&E staining and IHC analysis for MTFR2 and DNM1L from one HCC patient. Scale bars, 200 μm and 50 μm. *TEM* transmission electron microscopy, *HCC* hepatocellular carcinoma, *H&E* hematoxylin-eosin, *IHC* immunohistochemistry

**Fig. 8:**
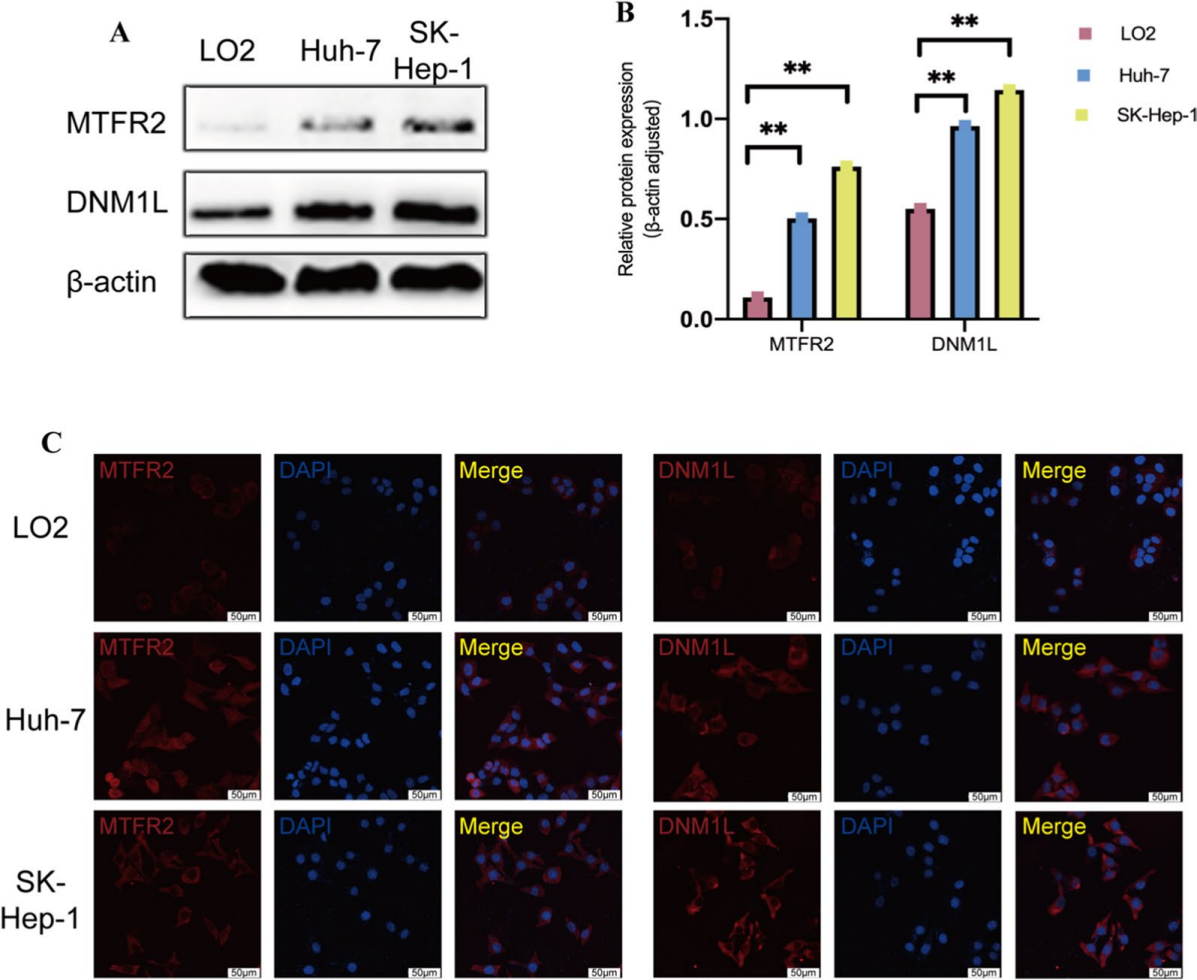
**A** Protein expression levels of MTFR2 and DNM1L in LO2, Huh-7 and SK-Hep-1 cell lines were analyzed by western blotting. **B** Quantification of western blotting results of MTFR2 and DNM1L. **C** The representative immunofluorescence image for staining of MTFR2 (left, red) and DNM1L (right, red). The nuclear was stained with DAPI (blue). Scale bar represents 50 mm. ** *P* < 0.01

The analysis above indicated that mitochondrial fission with higher expression levels of MTFR2 and DNM1L was related to a worse prognosis. To further clarify the mitochondrial morphology, TEM was used, and TEM images confirmed that mitochondrial morphology showed a balance of fusion and fission in the adjacent tissue (Fig. 7A), while mitochondrial status in the HCC tissue was fission reflected by the shrunken and fragmented mitochondria (Fig. 7B). H&E staining (Fig. 7C, D) and immunohistochemical staining (IHC) (Fig. 7E–H), showed that the protein expressions of MTFR2 and DNM1L in the HCC tissue were higher than in the adjacent tissue. The upregulated expression levels of MTFR2 and DNM1L in HCC were further confirmed in vitro, with the darker band in western blotting results (Fig. 8A, B) and stronger immunofluorescence signals in immunofluorescence images (Fig. 8C). Moreover, the presence of MTFR2 and DNM1L located in the cytoplasm was also figured out, as shown in Fig. 8C.

## Discussion

Mitochondria are central to various cellular processes, such as oxidative stress, metabolism, and apoptosis, all of which are known to be altered in cancer cells. Mitochondrial dynamics, involving fission and fusion processes, are essential for the maintenance of mitochondrial integrity and significantly impact cellular fate. Accumulating evidence indicates that mitochondrial dynamics are fundamentally involved in tumorigenesis, tumor progression, and the metabolic adaptability of cancer cells [41, 42]. However, the specific mitochondrial state that regulates cancer cell movement remains unclear. Therefore, it is crucial to uncover the detailed molecular mechanisms underlying mitochondrial dynamics and determine whether mitochondrial fusion or fission is more involved in HCC progression. This could lead to the identification of novel therapeutic targets and strategies for treating HCC. Additionally, GO/KEGG analyses revealed that Cluster 2 was highly associated with mitochondrial fission. Consequently, a ten-gene prognostic signature was generated based on the differentially expressed genes between the two clusters, all ten genes were overexpressed in the high-risk group. Furthermore, we investigated the intrinsic links between these ten signature genes and mitochondrial dynamics, suggesting their potential role in mediating mitochondrial fission through interaction with MTFR2. Finally, the expression of these ten genes was verified in HCC tissues and cell lines, confirming the results.

In this study, we initially screened 23 genes from MitoCarta 3.0 that were known to be associated with mitochondrial dynamics, and found that 21 of these genes were significantly overexpressed in tumor tissues. Notably, the expression of MTFR2 and DNM1L showed a positive correlation, and their encoded proteins exhibited an interaction. Based on the expression profiles of the 23 mitochondrial dynamic genes in HCC, we performed consensus clustering analysis and identified two distinct clusters. Interestingly, the worse prognostic cluster, Cluster2, was characterized by the upregulation of all identified fission genes, indicating its potential involvement in mitochondrial fission processes. This finding was further supported by GO/KEGG analyses, which confirmed that Cluster2 was indeed associated with mitochondrial fission. Consequently, we proposed that the two clusters, Cluster1 and Cluster2, exhibited different prognoses and mitochondrial characteristics, with Cluster2 showing higher expression of fission-promoting proteins and a worse prognosis for HCC patients. Moreover, among the dynamic genes, MTFR2 and DNM1L were significantly more highly expressed in Cluster2 compared to Cluster1. We identified the DEGs between the two clusters, which were considered to be related to mitochondrial dynamics, aiming to explore the molecular mechanism of mitochondrial dynamics in HCC development. Utilizing univariate Cox analysis, we screened for genes associated with OS, and subsequently, we used the LASSO regression model to construct a prognostic model consisting of ten selected mitochondrial dynamics-related genes. HCC patients were then classified into low- and high-risk groups based on their individual risk scores relative to the median value. According to this model, patients with a calculated high-risk score were associated with poor overall survival.

Furthermore, according to the GO/KEGG analyses, the high-risk group was significantly enriched in organelle fission (including mitochondrial fission) and cell cycle pathways. Notably, our findings suggest that MTFR2 can act as the central hub gene among these ten mitochondrial dynamics-related genes in HCC. Previous research has indicated that MTFR2 is associated with the mitochondrial outer membrane [43] and promotes mitochondrial fission activity through its interaction with DRP1 [39]. The available data strongly suggest that these ten genes contribute to mitochondrial fission by upregulating the expression of MTFR2 and DNM1L, subsequently exacerbating tumor progression through the cell cycle pathway. Additionally, the high-risk group exhibited universally increased levels of infiltrating immune cells and elevated expression of immune checkpoint molecules, suggesting an improved response to immune checkpoint blockade therapy. The experimental data further confirmed the morphological imbalance of mitochondria in HCC tissues, with a notable inclination towards fission. Moreover, the expression levels of MTFR2 and DNM1L were found to be upregulated in HCC tissues at the translational level. In addition, the protein expression levels of MTFR2 and DNM1L were increased in HCC cell lines. In this study, we first elaborate that the mitochondria in patients with poor prognosis are more inclined to fission by using the expression clustering of mitochondrial dynamics genes and combining it with the survival data of patients. Mitochondrial dynamics, including the two opposite processes of fusion and fission, are fundamental for cancer, including cell proliferation, invasion, and migration. MTFR2 was identified as the hub gene and booms the mitochondrial fission via up-regulating DRP1 in the high-risk group, where patients have a worse prognosis. Previous studies disclose high expression of MTFR2 is correlated with tumor proliferation in breast cancer and gastric cancer [44, 45]. However, the deep mechanism between MTFR2 and mitochondrial fission remains to be interrogated.

Mutation of the tumor suppressor gene TP53 is the general genetic change in cancers [46, 47], associated with an adverse prognosis [48]. The crucial role of TP53 as a tumor suppressor is to block cell cycle progression by delaying the G1/S phase transition [49]. When TP53 is downregulated or mutated, the p53 pathway is dysfunctional and responds to the transient cell cycle. Prior study has testified that p53 (encoded by TP53) was an upstream transcriptional activator of DRP1 and that mutant TP53 contributed to DRP1 transcriptional activation, leading to excessive mitochondrial fission [50]. Similar to previous findings, patients in the high-risk group with poorer prognoses possessed the highest somatic mutation frequency, 46%, for the TP53 mutation. In this study, we also found that patients in the high-risk group exhibited a higher expression of the ten risk dynamics-related genes, which interacted more with MTFR2, a more enrichment biological process in mitochondrial fission, and more involvement in the cell cycle pathway. Therefore, these findings suggest that an underlying mechanism of MTFR2/DRP1-mediated mitochondrial fission activated by TP53 mutation exacerbates HCC prognosis via the cell cycle pathway. In addition, Brahmer et al. revealed patients who harbored TP53 mutations were more sensitive to checkpoint blockade [51]. Combined with our finding that the TIDE score was lower in the high-risk group, we may underlie the response to ICB therapy for patients in the high-risk group because of the high frequency of TP53 mutation. As a universal way of immunotherapy, ICB treatment has made conducive progress, which aims to facilitate immune surveillance and target specific immune checkpoints, such as PD-L1 and CTLA4, which are widely used for HCC therapy [52]. Multiple studies have demonstrated a positive correlation between PD-L1 expression and response to ICB therapy [52]. Targeting these specific molecules might have more robust benefits for patients in the high-risk group. Overall, the patients in the high-risk group demonstrated better responses to immunotherapy.

Soaring studies have demonstrated the vital role of mitochondrial dynamics in cancer stem-like cells. However, the effects of mitochondrial fission versus fusion are conflicting [53]. For instance, Caino et al. revealed that inhibition of mitochondrial fission reduces the expression of stemness markers, such as NANOG and Oct4 [54]. In HCC, Liu et al. reported that downregulation of DRP1 weakens stemness by inhibiting mitochondrial fission [55]. Consistently, we found a positive correlation between the risk score and the TSI, indicating that the high-risk group, which exhibited more mitochondrial fission, was associated with enhanced stemness. On the other hand, a recent study of the drosophila neural stem cell-derived tumor model demonstrated that inhibiting mitochondrial fusion prevents tumorigenesis by blocking the immortalization of tumor-initiating cells [56]. Overall, the contradictory effects of mitochondrial fission and fusion on cancer stem-like cells might be induced by the response to a specific environment and meet distant functional requirements. Irrespective of that, our finding sheds light on the hypothesis that MTFR2/DRP1 axis-induced mitochondrial fission evokes stemness maintenance and promotes HCC formation and development.

In conclusion, we performed a new prognostic signature based on mitochondrial status. We showed that mitochondrial fission was involved more in HCC progress and was associated more with the cell cycle pathway, the maintenance of tumor stemness, TP53 mutation, and immune infiltration. Understanding the precise role of MTFR2 in the regulation of mitochondrial fission will not only advance our knowledge of HCC pathogenesis but also facilitate the development of new therapeutic strategies.

### Supplementary Information

**Additional file 1: Figure S1.** The KM curves of the ten genes of the prognostic model. **A** G6PD. **B** SLC1A7. **C** KPNA2. **D** NDRG1. **E** MYCN. **F** HAVCR1. **G** KIF20A. **H** MEX3A. **I** EZH2.

**Additional file 2: Table S1.** The symbols of mitochondrial dynamical genes. **Table S2.** The differentially expressed genes in the two clusters (|log2FC| > 1 and false discovery rate (FDR) < 0.001). **Table S3.** The differentially expressed genes in the two clusters (|log2FC| > 0.7 and false discovery rate (FDR) < 0.001). **Table S4.** The genes selected from the differentially expressed genes of the two clusters by univariate Cox regression analysis (P < 0.05). **Table S5**. The 11 topological analysis method results calculated by CytoHubba.

## Data Availability

By request.

## References

[1] Hepatocellular carcinoma (2016). Nat Rev Dis Primers.

[2] Roncalli M, Bianchi P, Bruni B, Laghi L, Destro A, Di Gioia S (2002). Methylation framework of cell cycle gene inhibitors in cirrhosis and associated hepatocellular carcinoma. Hepatology.

[3] Grattagliano I, Russmann S, Diogo C, Bonfrate L, Oliveira PJ, Wang DQ (2011). Mitochondria in chronic liver disease. Curr Drug Targets.

[4] Turkseven S, Bolognesi M, Brocca A, Pesce P, Angeli P, Di Pascoli M (2020). Mitochondria-targeted antioxidant mitoquinone attenuates liver inflammation and fibrosis in cirrhotic rats. Am J Physiol Gastrointest Liver Physiol.

[5] Adebayo M, Singh S, Singh AP, Dasgupta S (2021). Mitochondrial fusion and fission: the fine-tune balance for cellular homeostasis. FASEB J.

[6] Hernández-Alvarez MI, Zorzano A (2021). Mitochondrial dynamics and liver cancer. Cancers.

[7] Kraus F, Roy K, Pucadyil TJ, Ryan MT (2021). Function and regulation of the divisome for mitochondrial fission. Nature.

[8] Wang S, Tan J, Miao Y, Zhang Q (2022). Mitochondrial dynamics, mitophagy, and mitochondria-endoplasmic reticulum contact sites crosstalk under hypoxia. Front Cell Dev Biol (Review).

[9] Vona R, Mileo AM, Matarrese P (2021). Microtubule-based mitochondrial dynamics as a valuable therapeutic target in cancer. Cancers (Basel).

[10] Hocaoglu H, Sieber M (2022). Mitochondrial respiratory quiescence: a new model for examining the role of mitochondrial metabolism in development. Semin Cell Dev Biol.

[11] Xiong X, Hasani S, Young LEA, Rivas DR, Skaggs AT, Martinez R (2022). Activation of Drp1 promotes fatty acids-induced metabolic reprograming to potentiate wnt signaling in colon cancer. Cell Death Differ.

[12] Li Y, Chen H, Yang Q, Wan L, Zhao J, Wu Y (2022). Increased Drp1 promotes autophagy and ESCC progression by mtDNA stress mediated cGAS-STING pathway. J Exp Clin Cancer Res.

[13] Gbetuwa M, Lu LS, Wang TJ, Chen YJ, Chiou JF, Su TY (2022). Nucleus Near-Infrared (nNIR) irradiation of single A549 cells induces DNA damage and activates EGFR leading to mitochondrial fission. Cells.

[14] Romani P, Nirchio N, Arboit M, Barbieri V, Tosi A, Michielin F (2022). Mitochondrial fission links ECM mechanotransduction to metabolic redox homeostasis and metastatic chemotherapy resistance. Nat Cell Biol.

[15] Yu Y, Peng XD, Qian XJ, Zhang KM, Huang X, Chen YH (2021). Fis1 phosphorylation by Met promotes mitochondrial fission and hepatocellular carcinoma metastasis. Signal Transduct Target Ther.

[16] Song J, Yi X, Gao R, Sun L, Wu Z, Zhang S (2022). Impact of Drp1-mediated mitochondrial dynamics on T cell immune modulation. Front Immunol.

[17] Padder RA, Bhat ZI, Ahmad Z, Singh N, Husain M (2020). DRP1 promotes BRAF(V600E)-driven tumor progression and metabolic reprogramming in colorectal cancer. Front Oncol.

[18] Bao D, Zhao J, Zhou X, Yang Q, Chen Y, Zhu J (2019). Mitochondrial fission-induced mtDNA stress promotes tumor-associated macrophage infiltration and HCC progression. Oncogene.

[19] Simula L, Pacella I, Colamatteo A, Procaccini C, Cancila V, Bordi M (2018). Drp1 controls effective T cell immune-surveillance by regulating T cell migration, proliferation, and cmyc-dependent metabolic reprogramming. Cell Rep.

[20] Rath S, Sharma R, Gupta R, Ast T, Chan C, Durham TJ (2021). MitoCarta3.0: an updated mitochondrial proteome now with sub-organelle localization and pathway annotations. Nucleic Acids Res.

[21] Smyth GK (2005). Limma: linear models for microarray data. Bioinformatics and computational biology solutions using R and bioconductor.

[22] Mora A, Donaldson IM (2011). iRefR: an R package to manipulate the iRefIndex consolidated protein interaction database. BMC Bioinform.

[23] Wilkerson MD, Hayes DN (2010). ConsensusClusterPlus: a class discovery tool with confidence assessments and item tracking. Bioinformatics.

[24] Kassambara A, Kosinski M, Biecek P, Fabian S. Package ‘survminer’. Drawing survival curves using ‘ggplot2’(R package version 03 1) 2017.

[25] Therneau TM, Lumley T (2015). Package ‘survival’. R Top Doc.

[26] Yu G, Wang LG, Han Y, He QY (2012). clusterProfiler: an R package for comparing biological themes among gene clusters. OMICS.

[27] Engebretsen S, Bohlin J (2019). Statistical predictions with glmnet. Clin Epigenet.

[28] Gerds TA. Package ‘pec’2022.

[29] Krijthe J, van der Maaten L, Krijthe MJ. Package ‘Rtsne’. GitHub, 2018.

[30] Harrell FE Jr, Harrell MFE Jr, Hmisc D. Package ‘rms’. Vanderbilt Univ 2017; 229.

[31] Ligges U, Mächler M. Scatterplot3d-an r package for visualizing multivariate data. Technical Report, 2002.

[32] Kohl M, Wiese S, Warscheid B (2011). Cytoscape: software for visualization and analysis of biological networks. Data mining in proteomics.

[33] Li T, Fu J, Zeng Z, Cohen D, Li J, Chen Q (2020). TIMER2.0 for analysis of tumor-infiltrating immune cells. Nucleic Acids Res.

[34] Subramanian A, Tamayo P, Mootha VK, Mukherjee S, Ebert BL, Gillette MA (2005). Gene set enrichment analysis: a knowledge-based approach for interpreting genome-wide expression profiles. Proc Natl Acad Sci U S A.

[35] Malta TM, Sokolov A, Gentles AJ, Burzykowski T, Poisson L, Weinstein JN (2018). Machine learning identifies stemness features associated with oncogenic dedifferentiation. Cell.

[36] Miranda A, Hamilton PT, Zhang AW, Pattnaik S, Becht E, Mezheyeuski A (2019). Cancer stemness, intratumoral heterogeneity, and immune response across cancers. Proc Natl Acad Sci U S A.

[37] Jiang P, Gu S, Pan D, Fu J, Sahu A, Hu X (2018). Signatures of T cell dysfunction and exclusion predict cancer immunotherapy response. Nat Med.

[38] Mayakonda A, Lin D-C, Assenov Y, Plass C, Koeffler HP (2018). Maftools: efficient and comprehensive analysis of somatic variants in cancer. Genome Res.

[39] Luo Y, Liu S-T. MTFR2 regulates mitochondrial fission and impacts spindle integrity during mitosis. bioRxiv. 2020: 2020.2009.2013.293621.

[40] Park SJ, Bae JE, Jo DS, Kim JB, Park NY, Fang J (2021). Increased O-GlcNAcylation of Drp1 by amyloid-beta promotes mitochondrial fission and dysfunction in neuronal cells. Mol Brain.

[41] Trotta AP, Chipuk JE (2017). Mitochondrial dynamics as regulators of cancer biology. Cell Mol Life Sci.

[42] Su JF, Concilla A, Zhang DZ, Zhao F, Shen FF, Zhang H (2021). PIWI-interacting RNAs: mitochondria-based biogenesis and functions in cancer. Genes Dis.

[43] Go CD, Knight JDR, Rajasekharan A, Rathod B, Hesketh GG, Abe KT (2021). A proximity-dependent biotinylation map of a human cell. Nature.

[44] Lu G, Lai Y, Wang T, Lin W, Lu J, Ma Y (2019). Mitochondrial fission regulator 2 (MTFR2) promotes growth, migration, invasion and tumour progression in breast cancer cells. Aging.

[45] Li B, Wang W, Li Z, Chen Z, Zhi X, Xu J (2017). MicroRNA-148a-3p enhances cisplatin cytotoxicity in gastric cancer through mitochondrial fission induction and cyto-protective autophagy suppression. Cancer Lett.

[46] Yang C, Huang X, Li Y, Chen J, Lv Y, Dai S (2021). Prognosis and personalized treatment prediction in TP53-mutant hepatocellular carcinoma: an in silico strategy towards precision oncology. Brief Bioinform..

[47] Zhou Y, Jin J, Ji Y, Zhang J, Fu N, Chen M (2023). TP53 missense mutation reveals gain-of-function properties in small-sized KRAS transformed pancreatic ductal adenocarcinoma. J Transl Med.

[48] Li VD, Li KH, Li JT (2019). TP53 mutations as potential prognostic markers for specific cancers: analysis of data from the cancer genome atlas and the international agency for research on cancer TP53 database. J Cancer Res Clin Oncol.

[49] el-Deiry WS, Tokino T, Velculescu VE, Levy DB, Parsons R, Trent JM (1993). WAF1, a potential mediator of p53 tumor suppression. Cell.

[50] Zhou H, Zhu P, Wang J, Toan S, Ren J (2019). DNA-PKcs promotes alcohol-related liver disease by activating Drp1-related mitochondrial fission and repressing FUNDC1-required mitophagy. Signal Transduct Target Ther.

[51] Brahmer JR, Tykodi SS, Chow LQ, Hwu WJ, Topalian SL, Hwu P (2012). Safety and activity of anti-PD-L1 antibody in patients with advanced cancer. N Engl J Med.

[52] Ribas A, Wolchok JD (2018). Cancer immunotherapy using checkpoint blockade. Science.

[53] Tang M, Yang M, Wu G, Mo S, Wu X, Zhang S (2021). Epigenetic induction of mitochondrial fission is required for maintenance of liver cancer-initiating cells. Cancer Res.

[54] Caino MC, Seo JH, Aguinaldo A, Wait E, Bryant KG, Kossenkov AV (2016). A neuronal network of mitochondrial dynamics regulates metastasis. Nat Commun.

[55] Liu G, Luo Q, Li H, Liu Q, Ju Y, Song G (2020). Increased oxidative phosphorylation is required for stemness maintenance in liver cancer stem cells from hepatocellular carcinoma cell line HCCLM3 cells. Int J Mol Sci.

[56] Bonnay F, Veloso A, Steinmann V, Köcher T, Abdusselamoglu MD, Bajaj S (2020). Oxidative metabolism drives immortalization of neural stem cells during tumorigenesis. Cell.

