# Supplementary material for: MTFR2-dependent mitochondrial fission promotes HCC progression
Source: J Transl Med. 2024 Jan 18;22:73. doi: 10.1186/s12967-023-04845-6 (PMC10795309; doi:10.1186/s12967-023-04845-6)
Supplement: Supplementary file 1 — Additional file 1: Figure S1. The KM curves of the ten genes of the prognostic model. A G6PD. B SLC1A7. C KPNA2. D NDRG1. E MYCN. F HAVCR1. G KIF20A. H MEX3A. I EZH2. [file 12967_2023_4845_MOESM1_ESM.docx]

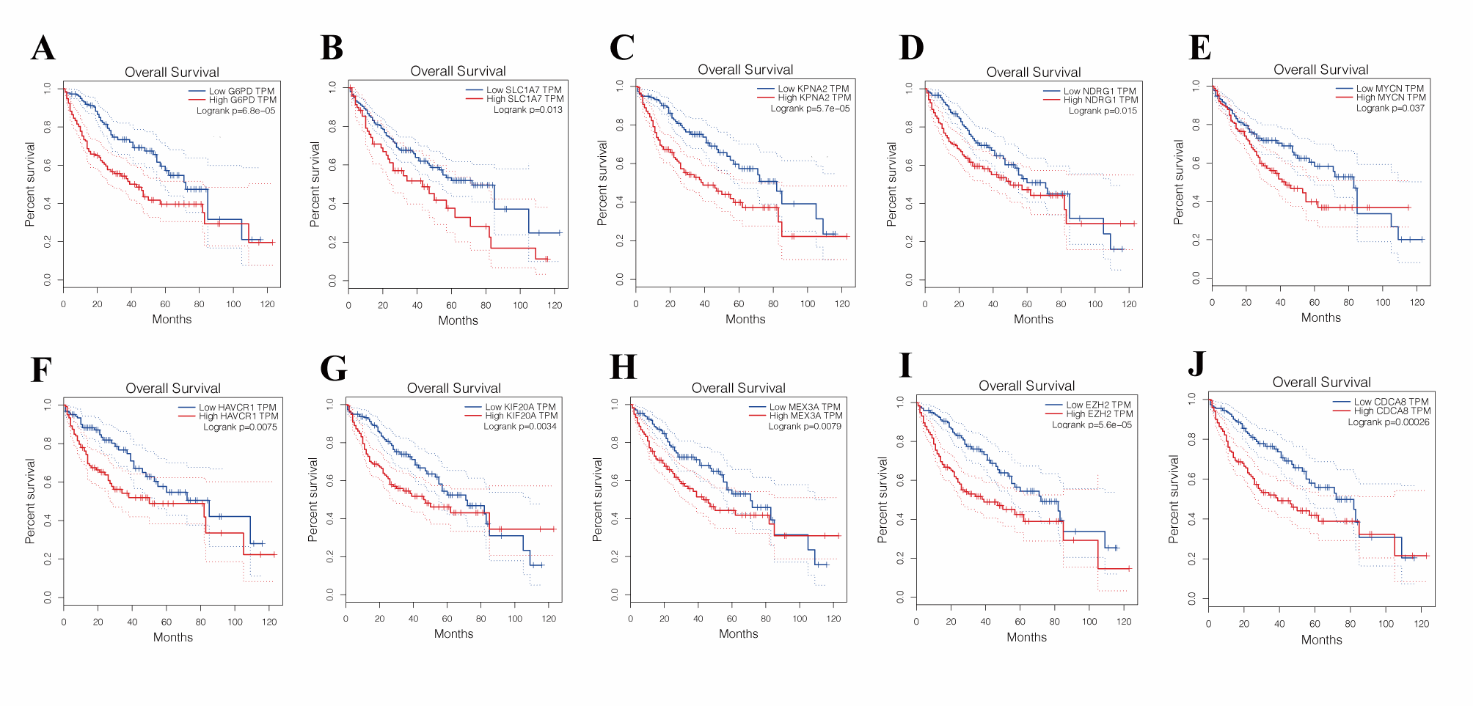


Figure S1. The KM curves of the ten genes of the prognostic model. (A). G6PD. (B). SLC1A7. (C). KPNA2. (D). NDRG1. (E). MYCN. (F). HAVCR1. (G). KIF20A. (H). MEX3A. (I). EZH2.
