# Supplementary material for: MTFR2-dependent mitochondrial fission promotes HCC progression
Source: J Transl Med. 2024 Jan 18;22:73. doi: 10.1186/s12967-023-04845-6 (PMC10795309; doi:10.1186/s12967-023-04845-6)
Supplement: Supplementary file 2 — Additional file 2: Table S1. The symbols of mitochondrial dynamical genes. Table S2. The differentially expressed genes in the two clusters (|log2FC| > 1 and false discovery rate (FDR) < 0.001). Table S3. The differentially expressed genes in the two clusters (|log2FC| > 0.7 and false discovery rate (FDR) < 0.001). Table S4. The genes selected from the differentially expressed genes of the two clusters by univariate Cox regression analysis (P < 0.05). Table S5. The 11 topological analysis method results calculated by CytoHubba. [file 12967_2023_4845_MOESM2_ESM.zip › Supplementary tables/Table S1.docx]

Table S1. The symbols of mitochondrial dynamical genes.

| Fission genes | Fusion genes | |
| --- | --- | --- |
| ARMC10 | ARL2 | |
| DNM1L | MFN1 | |
| FIS1 | MFN2 | |
| MFF | MIGA1 | |
| MIEF1 | MIGA2 | |
| MIEF2 | MTCH2 | |
| MTFP1 | OMA1 | |
| MTFR1 | OPA1 | |
| MTFR2 | PLD6 | |
| MUL1 |  | |
| OMA1 |  | |
| RAB24 |  | |
| SLC25A46 |  | |
| SPIRE1 |  |  |
| STX17 |  |  |
