# Supplementary material for: MTFR2-dependent mitochondrial fission promotes HCC progression
Source: J Transl Med. 2024 Jan 18;22:73. doi: 10.1186/s12967-023-04845-6 (PMC10795309; doi:10.1186/s12967-023-04845-6)
Supplement: Supplementary file 2 — Additional file 2: Table S1. The symbols of mitochondrial dynamical genes. Table S2. The differentially expressed genes in the two clusters (|log2FC| > 1 and false discovery rate (FDR) < 0.001). Table S3. The differentially expressed genes in the two clusters (|log2FC| > 0.7 and false discovery rate (FDR) < 0.001). Table S4. The genes selected from the differentially expressed genes of the two clusters by univariate Cox regression analysis (P < 0.05). Table S5. The 11 topological analysis method results calculated by CytoHubba. [file 12967_2023_4845_MOESM2_ESM.zip › Supplementary tables/Table S2.docx]

Table S2. The differentially expressed genes in the two clusters (|log2FC| > 1 and false discovery rate (FDR) < 0.001).

| Gene | Mean1 | Mean2 | logFC | pValue | fdr |
| --- | --- | --- | --- | --- | --- |
| BCORL1 | 1.2476066 | 2.51322834 | 1.01037866 | 1.22E-16 | 1.04E-15 |
| PGF | 0.93158973 | 2.42113611 | 1.37791754 | 3.56E-15 | 2.38E-14 |
| FJX1 | 1.00721474 | 2.91937906 | 1.53529024 | 9.07E-09 | 2.49E-08 |
| PDX1 | 0.89106353 | 3.52163277 | 1.98264428 | 1.26E-08 | 3.39E-08 |
| RRAD | 1.24780283 | 2.74222982 | 1.1359595 | 1.57E-07 | 3.68E-07 |
| DPYSL3 | 1.65658939 | 3.70182068 | 1.16001895 | 5.70E-05 | 9.82E-05 |
| SULT1C4 | 0.42865463 | 1.72863069 | 2.01174206 | 0.00055527 | 0.00084808 |
| TMC5 | 0.83391618 | 3.31902442 | 1.99278496 | 4.45E-09 | 1.27E-08 |
| XXYLT1 | 0.78020028 | 1.88218498 | 1.27049201 | 4.05E-26 | 2.80E-24 |
| ASPDH | 32.308963 | 12.1262322 | -1.4138031 | 5.66E-20 | 9.00E-19 |
| NEURL3 | 1.00760032 | 5.57855823 | 2.46896882 | 3.03E-22 | 7.81E-21 |
| TGFB2 | 0.45754174 | 1.78085749 | 1.96059682 | 1.77E-05 | 3.24E-05 |
| ARNT2 | 0.36130322 | 1.39864165 | 1.95274434 | 2.35E-15 | 1.62E-14 |
| TRIP10 | 3.45857409 | 8.05037916 | 1.21887937 | 1.52E-25 | 8.98E-24 |
| CHST4 | 0.55738315 | 3.13649059 | 2.49240994 | 2.25E-06 | 4.59E-06 |
| CIITA | 0.55821186 | 1.12912839 | 1.01632486 | 9.93E-06 | 1.88E-05 |
| GLIPR2 | 1.7591421 | 4.11159791 | 1.22482716 | 7.33E-14 | 4.02E-13 |
| ELFN1 | 6.14808254 | 2.84932894 | -1.1095144 | 2.60E-06 | 5.26E-06 |
| TMEM51 | 1.62639415 | 7.10912199 | 2.12799446 | 2.23E-24 | 9.35E-23 |
| SAA1 | 1710.65892 | 557.482887 | -1.6175527 | 9.49E-05 | 0.00015905 |
| TNNI2 | 1.13395514 | 3.28303494 | 1.53366654 | 9.19E-22 | 2.13E-20 |
| UCP2 | 6.12021218 | 13.4905855 | 1.14029939 | 9.34E-11 | 3.26E-10 |
| ANXA4 | 14.2135902 | 30.1440487 | 1.0846022 | 5.06E-06 | 9.89E-06 |
| AC007938.3 | 0.20627821 | 1.05850555 | 2.3593655 | 1.74E-13 | 9.01E-13 |
| CD52 | 7.6430003 | 17.7430344 | 1.21504177 | 3.57E-09 | 1.03E-08 |
| SFN | 17.437096 | 38.2226148 | 1.13226669 | 0.00048296 | 0.00074318 |
| FMNL2 | 1.36308019 | 3.61629709 | 1.40764277 | 8.27E-12 | 3.35E-11 |
| HK2 | 1.19753135 | 3.05881349 | 1.35290871 | 1.71E-13 | 8.88E-13 |
| DOCK2 | 0.43941158 | 0.89130862 | 1.02035217 | 5.90E-06 | 1.14E-05 |
| AP000346.2 | 0.32787149 | 0.84937657 | 1.37327386 | 3.88E-13 | 1.91E-12 |
| TPM2 | 6.93029352 | 19.4579632 | 1.48937234 | 1.58E-17 | 1.57E-16 |
| ZFAS1 | 13.1656528 | 28.8187301 | 1.13022771 | 1.33E-19 | 1.98E-18 |
| C6 | 66.4908303 | 23.084273 | -1.5262451 | 3.92E-23 | 1.22E-21 |
| RIBC2 | 0.44963887 | 1.33015558 | 1.56475632 | 2.91E-15 | 1.97E-14 |
| EBF4 | 1.87599784 | 3.88662772 | 1.05086076 | 8.27E-06 | 1.58E-05 |
| FZD2 | 0.26932039 | 1.32269917 | 2.29608962 | 3.17E-18 | 3.62E-17 |
| KRT20 | 2.38109843 | 7.3434011 | 1.62482114 | 0.02147212 | 0.02709871 |
| SORD2P | 5.36210878 | 2.56908062 | -1.0615483 | 4.94E-10 | 1.58E-09 |
| FANCI | 1.21132957 | 2.71857772 | 1.16626064 | 9.33E-23 | 2.73E-21 |
| TEDC2 | 1.18338015 | 2.85073845 | 1.26842208 | 3.23E-18 | 3.67E-17 |
| DOK2 | 2.06799843 | 4.22725906 | 1.03148743 | 1.60E-08 | 4.26E-08 |
| RASEF | 1.07456005 | 2.26994214 | 1.07890942 | 2.31E-05 | 4.18E-05 |
| ITPKA | 2.94648223 | 6.29623541 | 1.09549592 | 2.82E-10 | 9.27E-10 |
| SEPTIN4 | 6.6326297 | 3.01231198 | -1.1387098 | 0.00031525 | 0.00049565 |
| SPI1 | 4.07996634 | 9.3633987 | 1.19847504 | 1.33E-13 | 7.02E-13 |
| WDR91 | 2.53574731 | 5.10536136 | 1.00960209 | 9.21E-11 | 3.22E-10 |
| AC006329.1 | 3.83946637 | 1.34852348 | -1.5095252 | 4.00E-16 | 3.11E-15 |
| MEST | 5.82377239 | 13.4292212 | 1.20534977 | 1.75E-08 | 4.63E-08 |
| SAA2-SAA4 | 53.7441088 | 19.3133215 | -1.4765103 | 0.00015323 | 0.00025039 |
| PDP1 | 0.85517455 | 2.11470193 | 1.3061635 | 1.18E-10 | 4.06E-10 |
| TRAIP | 0.81814235 | 1.95668293 | 1.2579862 | 1.26E-22 | 3.59E-21 |
| AC092868.1 | 0.76548921 | 1.64778853 | 1.10607715 | 4.12E-06 | 8.14E-06 |
| IGKV1-5 | 16.9060453 | 54.0427153 | 1.67656094 | 0.0013888 | 0.00202985 |
| CHRNA4 | 1.80895411 | 0.78729908 | -1.2001721 | 0.00018633 | 0.00030141 |
| PON1 | 126.57507 | 54.7513486 | -1.2090269 | 5.91E-21 | 1.13E-19 |
| SKA3 | 0.94347261 | 2.41282631 | 1.35467152 | 2.91E-22 | 7.55E-21 |
| NAT14 | 2.1308364 | 5.44360623 | 1.35314288 | 8.14E-15 | 5.16E-14 |
| CAPG | 7.93744233 | 21.3306865 | 1.42618429 | 1.63E-18 | 1.99E-17 |
| TPRG1-AS1 | 8.57320402 | 3.20987114 | -1.4173191 | 1.82E-14 | 1.09E-13 |
| CCDC170 | 2.02952276 | 1.01115616 | -1.0051347 | 0.0006816 | 0.00103263 |
| HIC2 | 0.72026706 | 1.56859204 | 1.12286636 | 1.76E-16 | 1.45E-15 |
| AC092071.1 | 0.99434652 | 0.23787547 | -2.0635422 | 1.07E-09 | 3.33E-09 |
| RGS2 | 6.1392619 | 13.910207 | 1.18000677 | 1.52E-15 | 1.08E-14 |
| DCXR | 395.021467 | 169.970327 | -1.2166482 | 5.38E-17 | 4.85E-16 |
| FHOD1 | 1.24600561 | 2.49266964 | 1.00038113 | 1.07E-12 | 4.96E-12 |
| PRSS2 | 0.53348573 | 19.3502687 | 5.1807601 | 1.30E-07 | 3.08E-07 |
| ZNF43 | 0.33542055 | 0.81832983 | 1.28671134 | 2.82E-11 | 1.05E-10 |
| ATP13A2 | 2.55431356 | 5.48559374 | 1.10271214 | 2.22E-23 | 7.19E-22 |
| MUC5B | 2.11980498 | 6.21332309 | 1.55143353 | 0.00038216 | 0.00059621 |
| MARCKS | 14.1706008 | 32.4925279 | 1.19720707 | 2.04E-22 | 5.47E-21 |
| LAPTM5 | 17.9538646 | 39.3772625 | 1.1330684 | 5.37E-13 | 2.59E-12 |
| RAB42 | 0.36946114 | 1.33816355 | 1.85675992 | 2.63E-18 | 3.05E-17 |
| UICLM | 0.40814284 | 0.90463569 | 1.14826276 | 0.0107987 | 0.01414507 |
| NOP56P1 | 0.35874971 | 0.83793434 | 1.22385952 | 0.00990103 | 0.01302977 |
| AP000757.2 | 0.12779384 | 1.51231965 | 3.56487296 | 1.20E-15 | 8.66E-15 |
| TNFAIP6 | 0.18583533 | 1.33179943 | 2.84128014 | 1.30E-11 | 5.10E-11 |
| LRP12 | 0.35087202 | 0.79895559 | 1.1871704 | 2.91E-06 | 5.86E-06 |
| PROM1 | 0.1684889 | 2.47764959 | 3.87824672 | 3.08E-15 | 2.08E-14 |
| ITGAX | 1.31059042 | 2.62718031 | 1.00329833 | 2.75E-10 | 9.05E-10 |
| ZNF579 | 2.17925427 | 4.479464 | 1.03949158 | 1.04E-20 | 1.89E-19 |
| NCKAP1L | 0.86574714 | 1.75925716 | 1.02294876 | 1.08E-06 | 2.27E-06 |
| PCDHB5 | 0.52489621 | 1.29880426 | 1.30707994 | 0.00258602 | 0.00365506 |
| TRBJ2-7 | 0.4453124 | 0.92277503 | 1.05116116 | 0.01298516 | 0.01684009 |
| ISYNA1 | 4.53325188 | 16.5065664 | 1.86442182 | 2.05E-12 | 9.07E-12 |
| LINC01348 | 5.22744131 | 2.51437795 | -1.0559034 | 2.81E-07 | 6.37E-07 |
| PRR36 | 0.24630569 | 0.97298388 | 1.98196594 | 2.88E-18 | 3.30E-17 |
| ELF4 | 0.74693195 | 2.80879052 | 1.91090031 | 1.04E-15 | 7.58E-15 |
| ZWINT | 5.42305461 | 11.4140706 | 1.07363578 | 6.24E-18 | 6.61E-17 |
| C19orf48 | 11.4014219 | 24.418798 | 1.09877842 | 1.83E-26 | 1.42E-24 |
| AC253536.2 | 0.68751579 | 0.32920535 | -1.0624051 | 8.98E-08 | 2.16E-07 |
| FAM117B | 0.71836715 | 1.91252947 | 1.4126887 | 3.04E-19 | 4.24E-18 |
| AC060766.3 | 0.43421073 | 1.0262796 | 1.24095656 | 2.39E-09 | 7.06E-09 |
| IGHV3-73 | 1.55559265 | 10.0490201 | 2.6915186 | 0.00955043 | 0.0125907 |
| FABP5 | 1.85292454 | 5.90120324 | 1.67120502 | 1.62E-18 | 1.97E-17 |
| SNRPD2 | 52.2633442 | 116.050352 | 1.15087955 | 1.07E-24 | 4.83E-23 |
| ARMCX6 | 0.88010376 | 2.14526848 | 1.28541269 | 5.37E-12 | 2.24E-11 |
| CCNJ | 0.61892171 | 1.31069881 | 1.08250736 | 2.80E-10 | 9.22E-10 |
| IGLV2-23 | 12.177824 | 62.9143488 | 2.36913272 | 0.00253329 | 0.0035848 |
| CYP39A1 | 6.23129654 | 2.1565642 | -1.5307977 | 4.13E-09 | 1.18E-08 |
| RDH16 | 59.8618433 | 20.0159432 | -1.5804871 | 4.34E-19 | 5.90E-18 |
| PI3 | 4.68829974 | 9.82182412 | 1.06692618 | 8.67E-09 | 2.39E-08 |
| TCTN2 | 0.55381695 | 1.52903049 | 1.46513608 | 3.56E-15 | 2.38E-14 |
| AP001505.1 | 2.47326654 | 5.71908529 | 1.2093667 | 2.38E-18 | 2.79E-17 |
| ABR | 0.84538553 | 2.36923507 | 1.48674002 | 4.75E-12 | 2.00E-11 |
| RFLNA | 0.23746506 | 2.46365773 | 3.37501468 | 2.20E-08 | 5.74E-08 |
| SLC7A7 | 1.31038405 | 3.20511429 | 1.2903861 | 3.27E-14 | 1.89E-13 |
| ORC6 | 0.4812624 | 1.3762551 | 1.5158523 | 6.03E-24 | 2.26E-22 |
| LINC01485 | 38.2819447 | 14.8924441 | -1.3620836 | 2.16E-18 | 2.55E-17 |
| NPFFR2 | 0.39769948 | 0.94609494 | 1.25030628 | 5.96E-05 | 0.00010239 |
| COX7A1 | 2.92712033 | 11.302446 | 1.94908107 | 0.0007125 | 0.00107631 |
| CARMIL1 | 0.96442018 | 2.0096192 | 1.05918841 | 1.90E-09 | 5.68E-09 |
| PCED1B-AS1 | 0.68312288 | 1.50753112 | 1.14197077 | 9.19E-10 | 2.87E-09 |
| AFAP1 | 0.65765376 | 1.45364761 | 1.14427744 | 1.32E-10 | 4.51E-10 |
| SLC16A5 | 0.43924623 | 1.04495234 | 1.25033534 | 8.35E-07 | 1.78E-06 |
| SNORA33 | 3.03108144 | 6.47349898 | 1.09471309 | 8.06E-18 | 8.37E-17 |
| FSCN1 | 5.86986307 | 17.2027447 | 1.55124001 | 5.22E-12 | 2.18E-11 |
| HDAC7 | 2.03201191 | 4.85558662 | 1.25673675 | 1.35E-25 | 8.26E-24 |
| TRIM6 | 0.41438473 | 0.87563267 | 1.07935496 | 9.92E-10 | 3.09E-09 |
| PGC | 42.6876326 | 98.2146701 | 1.20212038 | 2.32E-14 | 1.37E-13 |
| SSC4D | 1.53879611 | 3.92718569 | 1.35169373 | 3.13E-05 | 5.58E-05 |
| SELL | 1.33831472 | 3.00857817 | 1.16866442 | 0.00695126 | 0.00932191 |
| GPR160 | 0.66480296 | 1.73818195 | 1.38658039 | 7.21E-17 | 6.35E-16 |
| CUX2 | 6.73889125 | 3.01817641 | -1.1588341 | 1.85E-11 | 7.08E-11 |
| CD79B | 1.1306067 | 2.74468755 | 1.27954477 | 4.13E-09 | 1.18E-08 |
| NRGN | 1.74546256 | 3.79022725 | 1.11867494 | 1.18E-12 | 5.42E-12 |
| UPP2 | 5.1070026 | 1.36532063 | -1.903237 | 1.08E-07 | 2.58E-07 |
| G6PC | 194.96049 | 93.1757445 | -1.0651554 | 2.82E-14 | 1.65E-13 |
| C1QC | 49.3993152 | 101.132541 | 1.03368434 | 2.22E-05 | 4.02E-05 |
| LAMP3 | 0.61631675 | 1.35022511 | 1.13145604 | 2.46E-10 | 8.15E-10 |
| IGKV2-29 | 0.31305727 | 3.8836017 | 3.63289673 | 0.00026646 | 0.0004231 |
| AC068533.2 | 1.2176344 | 0.60820031 | -1.0014626 | 3.74E-06 | 7.43E-06 |
| MCCD1 | 0.82077391 | 4.90072968 | 2.5779398 | 3.09E-07 | 6.95E-07 |
| KIF2C | 1.71070845 | 5.21469379 | 1.60798863 | 9.76E-25 | 4.47E-23 |
| UGT2B15 | 156.350343 | 66.0292839 | -1.2436045 | 8.13E-18 | 8.43E-17 |
| STMN3 | 1.60098424 | 4.44028005 | 1.47169156 | 1.98E-10 | 6.62E-10 |
| RNF125 | 1.8842725 | 0.89090644 | -1.0806618 | 4.57E-15 | 3.01E-14 |
| REN | 2.13485653 | 0.67521346 | -1.6607235 | 1.64E-08 | 4.36E-08 |
| SMIM10 | 0.69377169 | 1.61835894 | 1.22199875 | 2.49E-05 | 4.48E-05 |
| AC004812.2 | 0.35650867 | 0.72096161 | 1.01598525 | 5.16E-21 | 1.01E-19 |
| RAB6B | 0.43898093 | 1.23106805 | 1.48768035 | 3.54E-20 | 5.83E-19 |
| BLVRA | 7.98152411 | 18.1019975 | 1.18141274 | 9.39E-24 | 3.39E-22 |
| CYP3A4 | 513.178377 | 109.802215 | -2.2245532 | 2.34E-13 | 1.19E-12 |
| RNF24 | 0.72402307 | 1.77700489 | 1.29534007 | 3.94E-25 | 2.03E-23 |
| TES | 4.03438628 | 8.24973162 | 1.03199796 | 4.30E-12 | 1.82E-11 |
| WASF1 | 2.07235547 | 4.49839806 | 1.11813984 | 1.11E-15 | 8.02E-15 |
| CCL13 | 0.35796201 | 0.92005866 | 1.36191935 | 4.38E-05 | 7.67E-05 |
| MMP10 | 0.28394227 | 1.89266004 | 2.73674577 | 3.82E-16 | 2.99E-15 |
| CCNB1 | 5.47811286 | 14.9761432 | 1.45091524 | 3.06E-25 | 1.64E-23 |
| B3GNT7 | 0.57925675 | 2.30926687 | 1.99516004 | 1.87E-10 | 6.26E-10 |
| B3GNT8 | 0.35528585 | 1.37307537 | 1.95035868 | 5.66E-18 | 6.07E-17 |
| LRFN1 | 0.37184365 | 1.26448699 | 1.76578416 | 2.54E-20 | 4.30E-19 |
| NRM | 4.17425689 | 11.78412 | 1.49725273 | 3.22E-28 | 4.24E-26 |
| GOLGA7B | 0.41651124 | 1.29732834 | 1.63911631 | 3.09E-13 | 1.54E-12 |
| AC020978.4 | 2.86790792 | 0.94966706 | -1.594505 | 6.00E-15 | 3.88E-14 |
| AC008708.1 | 0.90387735 | 0.35738772 | -1.3386369 | 1.58E-08 | 4.20E-08 |
| SYT8 | 0.46920336 | 3.18529007 | 2.76313952 | 3.75E-17 | 3.47E-16 |
| CA14 | 2.54975479 | 1.11358858 | -1.1951422 | 7.97E-07 | 1.71E-06 |
| HUNK | 0.27158156 | 1.39823996 | 2.36415453 | 1.83E-11 | 6.99E-11 |
| ADAM23 | 0.42722815 | 1.00627731 | 1.23594931 | 9.61E-06 | 1.82E-05 |
| AC015912.3 | 0.80944684 | 2.38200922 | 1.55717075 | 2.67E-14 | 1.56E-13 |
| EXO1 | 0.80573391 | 1.98699641 | 1.3022139 | 5.98E-19 | 7.88E-18 |
| IGSF1 | 0.50272885 | 2.68365045 | 2.41634438 | 1.07E-10 | 3.72E-10 |
| SLCO3A1 | 1.00940464 | 2.1241346 | 1.07337056 | 2.57E-07 | 5.83E-07 |
| IGLV2-14 | 12.2709438 | 38.7988634 | 1.66076817 | 0.00029179 | 0.00046145 |
| IFNGR2 | 11.952748 | 24.0261214 | 1.00726143 | 8.14E-26 | 5.20E-24 |
| MFGE8 | 2.82961639 | 5.8314336 | 1.04324412 | 1.53E-12 | 6.92E-12 |
| IGHV4-61 | 0.53367163 | 1.46199162 | 1.45391081 | 0.00728586 | 0.00974836 |
| COL9A2 | 0.45124402 | 2.49564671 | 2.467434 | 2.89E-26 | 2.12E-24 |
| PHLDB1 | 0.47663585 | 1.13877355 | 1.25652151 | 2.80E-10 | 9.22E-10 |
| MKI67 | 1.73284657 | 4.33055365 | 1.32140756 | 2.78E-21 | 5.75E-20 |
| EVPL | 0.25970148 | 1.22204804 | 2.23437486 | 1.07E-10 | 3.71E-10 |
| AC016405.3 | 0.64036366 | 1.34212645 | 1.06755725 | 3.11E-05 | 5.53E-05 |
| TMEM237 | 0.68794244 | 1.47970842 | 1.10495316 | 6.05E-23 | 1.82E-21 |
| CITED4 | 18.620572 | 38.0646164 | 1.03155315 | 7.45E-06 | 1.43E-05 |
| TBKBP1 | 1.25311492 | 2.62918475 | 1.0690968 | 1.93E-19 | 2.80E-18 |
| GLYATL1 | 19.5686918 | 5.9242861 | -1.7238341 | 9.70E-28 | 1.11E-25 |
| CD3E | 2.25448851 | 4.82511168 | 1.09776217 | 1.30E-05 | 2.43E-05 |
| TUBB6 | 2.52187915 | 6.51560879 | 1.36940084 | 1.16E-08 | 3.13E-08 |
| DMKN | 0.36191866 | 3.56638756 | 3.30072609 | 1.78E-17 | 1.75E-16 |
| AC006077.2 | 0.58186293 | 1.18954359 | 1.03165689 | 4.23E-11 | 1.55E-10 |
| KCNF1 | 0.32746445 | 1.38479092 | 2.08025797 | 4.60E-14 | 2.61E-13 |
| OXTR | 0.14904746 | 1.51148416 | 3.34212215 | 2.75E-14 | 1.61E-13 |
| RELT | 0.32524982 | 0.7992916 | 1.29717369 | 8.05E-22 | 1.89E-20 |
| SLC39A4 | 3.08643733 | 6.30042469 | 1.02950658 | 5.08E-08 | 1.26E-07 |
| SFT2D1 | 2.73519015 | 5.48206115 | 1.00307729 | 7.89E-26 | 5.05E-24 |
| SULT1C2 | 1.51382049 | 3.49802691 | 1.20834725 | 3.05E-14 | 1.77E-13 |
| G6PD | 6.06781367 | 24.769043 | 2.02928944 | 6.49E-28 | 7.89E-26 |
| VAV3 | 0.49457284 | 1.25138116 | 1.33926638 | 1.39E-05 | 2.59E-05 |
| CDC25B | 9.14947305 | 19.1570279 | 1.06611319 | 3.76E-18 | 4.19E-17 |
| MYG1-AS1 | 0.50229259 | 1.0496774 | 1.06334612 | 2.23E-20 | 3.80E-19 |
| CYP4F2 | 38.5980766 | 16.3134285 | -1.2424689 | 8.00E-19 | 1.03E-17 |
| LOX | 1.26050049 | 2.72150535 | 1.1104082 | 1.19E-07 | 2.83E-07 |
| RHEX | 0.60626771 | 2.42749737 | 2.00144284 | 4.67E-06 | 9.16E-06 |
| DDR1 | 2.28928938 | 9.78759921 | 2.09605518 | 6.15E-16 | 4.65E-15 |
| NAALADL1 | 0.86906223 | 3.13412621 | 1.85053188 | 6.35E-15 | 4.08E-14 |
| OLFML2B | 2.05443148 | 5.82763117 | 1.50417036 | 9.43E-13 | 4.41E-12 |
| CD27 | 0.97171863 | 2.83891368 | 1.54672845 | 3.31E-08 | 8.39E-08 |
| TRBJ2-3 | 0.41195312 | 0.86060937 | 1.06287838 | 0.00074294 | 0.00112002 |
| HLA-DQA1 | 4.71471485 | 11.095388 | 1.2347177 | 1.11E-07 | 2.65E-07 |
| CRYBB1 | 0.26995392 | 5.36495459 | 4.3127809 | 4.73E-06 | 9.28E-06 |
| FXYD3 | 1.03264999 | 5.81114305 | 2.49247063 | 9.02E-15 | 5.66E-14 |
| HLA-DPB1 | 33.0053772 | 70.6438917 | 1.09786374 | 1.35E-08 | 3.62E-08 |
| ZNF607 | 0.40879116 | 0.87334184 | 1.09518245 | 8.85E-11 | 3.11E-10 |
| KPNA2 | 13.6658585 | 29.2258175 | 1.09666729 | 2.94E-22 | 7.61E-21 |
| CKAP2L | 0.71479413 | 1.5442893 | 1.11134335 | 8.35E-16 | 6.17E-15 |
| EME1 | 0.51310644 | 1.2484616 | 1.2828214 | 2.61E-22 | 6.82E-21 |
| EPHB6 | 0.76045059 | 2.7134094 | 1.83518031 | 5.11E-09 | 1.45E-08 |
| ZNF14 | 0.50337662 | 1.23190496 | 1.29118084 | 8.29E-16 | 6.13E-15 |
| HLA-DRA | 183.566629 | 368.986062 | 1.00726251 | 7.04E-05 | 0.00012001 |
| TYMS | 6.15918222 | 12.7469867 | 1.04934552 | 1.43E-15 | 1.01E-14 |
| IGHV1-24 | 3.29884151 | 18.0204853 | 2.44960649 | 9.75E-05 | 0.00016307 |
| SLFN13 | 0.39175949 | 1.80753173 | 2.20598085 | 4.87E-10 | 1.56E-09 |
| ENO2 | 0.62158626 | 3.0261334 | 2.28344908 | 1.19E-20 | 2.13E-19 |
| ANKS6 | 0.92350742 | 2.24008176 | 1.27835593 | 4.31E-20 | 6.98E-19 |
| CFHR3 | 37.4242144 | 13.440749 | -1.4773585 | 2.09E-12 | 9.25E-12 |
| PHF19 | 1.27432999 | 2.98911688 | 1.22998039 | 1.01E-24 | 4.58E-23 |
| PIGS | 2.43752795 | 5.47912283 | 1.16852619 | 1.58E-24 | 6.88E-23 |
| ECM2 | 6.42089352 | 2.82865207 | -1.1826593 | 1.30E-19 | 1.93E-18 |
| LINC01667 | 0.27107278 | 1.26342354 | 2.2205862 | 0.00082186 | 0.00123305 |
| ANXA10 | 10.7948886 | 3.99322134 | -1.4347234 | 3.00E-16 | 2.39E-15 |
| AC007099.1 | 0.22543496 | 1.22146828 | 2.43783321 | 0.0008215 | 0.00123265 |
| PRR15L | 2.88972371 | 10.0855837 | 1.80329111 | 5.11E-08 | 1.27E-07 |
| AC114786.2 | 0.38286625 | 1.35139679 | 1.81953894 | 3.25E-07 | 7.28E-07 |
| IGKV3D-15 | 0.27120744 | 1.46136479 | 2.42984769 | 0.00049288 | 0.00075795 |
| RNF227 | 0.34347979 | 0.79608391 | 1.21269528 | 4.61E-17 | 4.20E-16 |
| TRBV7-9 | 0.47273228 | 0.95050398 | 1.00766929 | 0.00032233 | 0.00050644 |
| AJ009632.2 | 1.6176906 | 0.73054067 | -1.1468992 | 7.48E-05 | 0.00012721 |
| APBA1 | 1.7208587 | 0.85498621 | -1.0091556 | 4.70E-09 | 1.34E-08 |
| LAMP5 | 0.12485072 | 3.40854595 | 4.77088035 | 7.10E-18 | 7.43E-17 |
| PKMYT1 | 0.84142216 | 2.07615695 | 1.3030138 | 4.56E-20 | 7.32E-19 |
| NAT2 | 6.79279464 | 2.05980313 | -1.7214988 | 4.88E-15 | 3.20E-14 |
| WIPF1 | 1.52895229 | 3.24644488 | 1.08631732 | 9.49E-10 | 2.96E-09 |
| HOXC10 | 0.23054154 | 1.07151862 | 2.21655827 | 3.68E-06 | 7.31E-06 |
| ZNF107 | 0.37675876 | 0.82728326 | 1.13474032 | 7.56E-13 | 3.57E-12 |
| PACS1 | 3.48125668 | 7.12313538 | 1.03290422 | 6.05E-22 | 1.45E-20 |
| PCK1 | 137.468418 | 39.6593565 | -1.793367 | 7.19E-24 | 2.66E-22 |
| SERPINC1 | 2015.93478 | 751.199179 | -1.4241816 | 1.56E-28 | 2.38E-26 |
| PBK | 1.9525298 | 4.59692407 | 1.23532427 | 1.12E-14 | 6.96E-14 |
| MIR7111 | 0.45213753 | 1.25924074 | 1.47772055 | 2.88E-15 | 1.95E-14 |
| NUDT1 | 3.26489296 | 8.13135763 | 1.31646055 | 1.55E-27 | 1.68E-25 |
| IGLV8-61 | 2.39065022 | 21.1653377 | 3.14622854 | 0.00246465 | 0.00349328 |
| ZDHHC1 | 0.8343131 | 2.54032603 | 1.60635286 | 2.95E-14 | 1.72E-13 |
| ASF1B | 2.67683496 | 7.22750842 | 1.43297019 | 1.37E-23 | 4.65E-22 |
| PKM | 8.57640328 | 49.6779312 | 2.53416044 | 3.09E-31 | 1.45E-28 |
| ENDOD1 | 1.83178814 | 4.19117524 | 1.19410219 | 0.00087786 | 0.00131162 |
| CD5 | 0.70883257 | 1.43929208 | 1.02184258 | 1.46E-05 | 2.71E-05 |
| PIGZ | 1.19564763 | 2.45889899 | 1.0402202 | 1.66E-11 | 6.41E-11 |
| LINC02362 | 3.35274572 | 1.24183037 | -1.4328749 | 1.37E-13 | 7.24E-13 |
| ABCG2 | 7.0504594 | 3.18828312 | -1.1449375 | 4.84E-13 | 2.35E-12 |
| FCGR1A | 0.33642923 | 1.07888663 | 1.68116832 | 3.76E-10 | 1.22E-09 |
| ARHGEF25 | 0.65997162 | 1.6428306 | 1.31570783 | 5.71E-07 | 1.24E-06 |
| ANO9 | 0.30447443 | 1.93780529 | 2.67003063 | 8.59E-21 | 1.59E-19 |
| MTHFD1 | 30.4785081 | 12.9778415 | -1.2317418 | 5.08E-25 | 2.56E-23 |
| ILDR1 | 0.40104633 | 1.32956556 | 1.72911409 | 1.15E-10 | 3.97E-10 |
| IGHV3-13 | 0.60449428 | 2.10792209 | 1.80202095 | 0.00482327 | 0.0065994 |
| SLC2A1 | 1.32473795 | 4.35113877 | 1.71568603 | 4.65E-11 | 1.68E-10 |
| SLC7A8 | 0.71918514 | 1.74787386 | 1.28116596 | 1.68E-06 | 3.46E-06 |
| PIMREG | 0.27586282 | 1.37931955 | 2.3219338 | 9.49E-28 | 1.10E-25 |
| BEND3 | 0.38251033 | 0.91808568 | 1.2631301 | 3.33E-23 | 1.05E-21 |
| LINC02453 | 1.3224572 | 0.57860828 | -1.1925622 | 1.66E-08 | 4.40E-08 |
| RDH5 | 3.0514863 | 1.43855354 | -1.0848932 | 4.83E-15 | 3.17E-14 |
| FANCG | 2.11838312 | 4.31948018 | 1.02789418 | 2.86E-24 | 1.16E-22 |
| KCNS3 | 1.22351373 | 2.52977069 | 1.04797633 | 0.00323762 | 0.00451486 |
| VPS9D1-AS1 | 0.55694078 | 1.22509606 | 1.13729903 | 0.00021676 | 0.00034838 |
| UGT1A10 | 0.30063533 | 0.9590427 | 1.6735805 | 0.0059237 | 0.00802483 |
| MITF | 0.4061628 | 1.02707775 | 1.33841539 | 0.00423402 | 0.00582369 |
| DUSP9 | 3.95786486 | 20.7749097 | 2.39204795 | 3.02E-15 | 2.04E-14 |
| AL390198.1 | 0.87823784 | 1.83922436 | 1.06641387 | 2.91E-08 | 7.45E-08 |
| IGHV4-28 | 0.92987616 | 2.67670633 | 1.52534836 | 0.00962961 | 0.01268915 |
| RETREG1 | 4.04398245 | 1.78054844 | -1.1834551 | 7.42E-06 | 1.42E-05 |
| GPR35 | 0.66669416 | 1.93208225 | 1.53505952 | 8.08E-15 | 5.12E-14 |
| AL355102.4 | 1.16814422 | 4.38295999 | 1.9076871 | 5.70E-13 | 2.74E-12 |
| KIF23 | 0.69385433 | 1.93191996 | 1.4773306 | 7.80E-21 | 1.46E-19 |
| IL2RG | 4.62794786 | 12.1876373 | 1.39697396 | 4.59E-12 | 1.93E-11 |
| ZNF580 | 2.77158534 | 5.81742928 | 1.06967034 | 1.05E-27 | 1.17E-25 |
| ALDOB | 1862.87816 | 739.906738 | -1.332118 | 3.41E-18 | 3.85E-17 |
| AC141557.1 | 0.375759 | 1.14738617 | 1.61047145 | 1.77E-17 | 1.74E-16 |
| SLC4A11 | 0.24407328 | 1.11570852 | 2.19257387 | 1.21E-18 | 1.51E-17 |
| SCD5 | 0.43473544 | 4.63758591 | 3.4151644 | 4.27E-06 | 8.42E-06 |
| BCL2A1 | 1.11723675 | 2.66143741 | 1.25227071 | 5.18E-09 | 1.46E-08 |
| COL11A1 | 0.14704154 | 1.20853698 | 3.03896593 | 5.30E-09 | 1.50E-08 |
| SCUBE1 | 2.26389976 | 1.06363297 | -1.0898097 | 2.09E-05 | 3.79E-05 |
| TMEM159 | 0.69663195 | 2.16782202 | 1.63777778 | 7.69E-10 | 2.41E-09 |
| AACS | 0.72047613 | 1.50985787 | 1.0673902 | 3.28E-22 | 8.36E-21 |
| AURKB | 2.01286853 | 7.39985091 | 1.87824326 | 4.46E-29 | 8.05E-27 |
| PRKAR1B-AS1 | 0.56707439 | 1.26043833 | 1.15231563 | 6.58E-14 | 3.64E-13 |
| Z99572.1 | 0.63559606 | 0.3159302 | -1.0085043 | 1.33E-05 | 2.47E-05 |
| LAMA5-AS1 | 7.24214688 | 3.47164374 | -1.0607985 | 7.72E-12 | 3.14E-11 |
| KEL | 0.52918245 | 2.05647962 | 1.95833966 | 5.95E-06 | 1.15E-05 |
| PNCK | 0.25654526 | 1.85176766 | 2.85161782 | 1.38E-18 | 1.70E-17 |
| TMEM91 | 1.10799623 | 2.39200958 | 1.1102702 | 4.49E-17 | 4.10E-16 |
| AC060780.1 | 0.52553632 | 1.23355253 | 1.23095678 | 0.00410076 | 0.00564977 |
| VXN | 0.89484897 | 0.38384453 | -1.2211221 | 8.16E-14 | 4.43E-13 |
| TRPM2 | 0.38745417 | 1.16110303 | 1.58339841 | 2.44E-19 | 3.45E-18 |
| PLA2G7 | 2.71590472 | 6.00276498 | 1.14419432 | 5.66E-08 | 1.40E-07 |
| TK1 | 13.7734636 | 29.6704485 | 1.10713534 | 3.49E-16 | 2.74E-15 |
| ELMO3 | 3.24745365 | 6.82250601 | 1.07099283 | 0.00012695 | 0.0002096 |
| HTRA3 | 1.5902895 | 4.53178992 | 1.51079156 | 5.63E-11 | 2.02E-10 |
| GSTM1 | 28.9993669 | 6.74245703 | -2.1046751 | 0.02756011 | 0.03423943 |
| CGAS | 0.33061108 | 0.80392064 | 1.28191803 | 4.92E-07 | 1.08E-06 |
| CDK19 | 1.44467793 | 2.99285228 | 1.05077317 | 3.53E-18 | 3.97E-17 |
| SCTR-AS1 | 0.65248341 | 1.51252003 | 1.21294112 | 5.51E-05 | 9.50E-05 |
| CLIP2 | 1.86976911 | 4.75298889 | 1.3459749 | 1.39E-12 | 6.33E-12 |
| ANXA13 | 4.42783777 | 14.497956 | 1.71117525 | 5.49E-08 | 1.36E-07 |
| TC2N | 1.35577121 | 2.94481281 | 1.11906219 | 0.00034338 | 0.00053803 |
| MXRA5 | 0.62100653 | 1.63634665 | 1.39779807 | 0.01460769 | 0.01881587 |
| SGO2 | 0.49915259 | 1.26218737 | 1.33837327 | 2.14E-21 | 4.52E-20 |
| OCA2 | 0.61344056 | 1.47170135 | 1.26248949 | 1.03E-10 | 3.60E-10 |
| GRPR | 0.7803777 | 0.25083196 | -1.6374513 | 0.01093152 | 0.01431107 |
| GPRIN1 | 0.66306816 | 1.65909516 | 1.32316755 | 4.72E-19 | 6.36E-18 |
| SCTR | 0.36919436 | 6.81993954 | 4.20730653 | 3.70E-06 | 7.34E-06 |
| AC023043.3 | 0.57134779 | 1.32616587 | 1.21482012 | 7.76E-15 | 4.94E-14 |
| ACKR2 | 2.22046512 | 1.05513663 | -1.0734321 | 1.63E-16 | 1.36E-15 |
| CYP8B1 | 100.390188 | 23.2462268 | -2.1105498 | 1.31E-22 | 3.71E-21 |
| AEBP1 | 12.3877867 | 26.7686997 | 1.11162861 | 0.00313325 | 0.00437884 |
| LFNG | 1.24228733 | 2.8212147 | 1.18331757 | 1.08E-11 | 4.31E-11 |
| ESPL1 | 0.94984336 | 1.97642497 | 1.05713167 | 1.16E-14 | 7.16E-14 |
| GPD1 | 22.8109395 | 10.8116783 | -1.0771354 | 3.18E-12 | 1.37E-11 |
| AC095057.3 | 0.3654221 | 0.84399406 | 1.20766896 | 5.64E-12 | 2.34E-11 |
| CD2 | 2.96826606 | 6.50040624 | 1.13090947 | 9.75E-07 | 2.07E-06 |
| MTCL1 | 0.25120266 | 1.06459347 | 2.08337896 | 2.66E-15 | 1.81E-14 |
| ITGA3 | 1.02146963 | 5.28359962 | 2.37087484 | 1.49E-09 | 4.55E-09 |
| PIK3CD | 0.77477207 | 1.8136844 | 1.22707958 | 4.47E-11 | 1.62E-10 |
| CYP26A1 | 0.8716969 | 0.35567128 | -1.2932821 | 0.02363734 | 0.02963233 |
| ANKRD13D | 1.52958811 | 3.5480394 | 1.21387882 | 1.50E-34 | 1.61E-31 |
| PLPP2 | 1.61879866 | 7.3199645 | 2.17691309 | 8.10E-14 | 4.41E-13 |
| PTPN14 | 0.43854942 | 0.97658182 | 1.15500149 | 1.99E-09 | 5.96E-09 |
| RENBP | 3.43366532 | 11.7331855 | 1.77277342 | 1.83E-14 | 1.10E-13 |
| ABHD17C | 1.9089467 | 4.14287597 | 1.11785581 | 4.19E-16 | 3.25E-15 |
| GNMT | 95.592583 | 33.7247078 | -1.5030927 | 1.45E-11 | 5.66E-11 |
| IGHV3-66 | 0.82222223 | 4.85964383 | 2.56325029 | 0.0089835 | 0.01188442 |
| CHEK1 | 0.8732504 | 1.93677854 | 1.14919169 | 2.85E-22 | 7.41E-21 |
| CSAG1 | 2.78857373 | 7.58406463 | 1.44344384 | 0.0342048 | 0.04199121 |
| EMP3 | 4.79898431 | 12.8886498 | 1.42530013 | 1.93E-13 | 9.96E-13 |
| AP000445.1 | 0.67959624 | 0.2439146 | -1.4783017 | 2.89E-15 | 1.96E-14 |
| ZNF385B | 2.22571282 | 0.98516947 | -1.1758236 | 1.72E-09 | 5.19E-09 |
| CDH24 | 0.65863794 | 1.49108521 | 1.17880518 | 3.64E-11 | 1.34E-10 |
| AC004816.1 | 0.68468662 | 1.43290704 | 1.0654293 | 1.59E-10 | 5.38E-10 |
| IFI27L2 | 2.80031571 | 7.69733327 | 1.45876923 | 2.27E-20 | 3.87E-19 |
| LRRC8B | 0.76670653 | 1.59576728 | 1.0575039 | 9.13E-12 | 3.67E-11 |
| CIB2 | 0.88622981 | 2.25084615 | 1.3447147 | 1.42E-15 | 1.01E-14 |
| VNN2 | 4.62216446 | 9.43629353 | 1.02965171 | 1.90E-05 | 3.46E-05 |
| ZNF117 | 0.41642315 | 0.91499294 | 1.13571033 | 1.36E-11 | 5.34E-11 |
| TACC3 | 2.88431079 | 7.53901392 | 1.38614921 | 2.79E-29 | 5.70E-27 |
| TRBV28 | 2.05043694 | 4.74887536 | 1.21165452 | 2.94E-07 | 6.63E-07 |
| CD8A | 1.25843752 | 2.97976317 | 1.24356408 | 7.43E-05 | 0.00012651 |
| TNFRSF4 | 1.53102159 | 3.15973636 | 1.04530956 | 8.41E-17 | 7.32E-16 |
| DUSP2 | 1.32445855 | 2.84317222 | 1.1020988 | 2.04E-11 | 7.77E-11 |
| TMEM151A | 0.35786881 | 1.94961614 | 2.44568739 | 1.15E-08 | 3.11E-08 |
| CD79A | 0.94410002 | 4.71196242 | 2.31931642 | 3.90E-07 | 8.66E-07 |
| SPARCL1 | 57.0357945 | 25.3275486 | -1.1711602 | 6.22E-05 | 0.00010666 |
| TAX1BP3 | 5.98574972 | 16.0604382 | 1.42390739 | 3.76E-23 | 1.18E-21 |
| PROK1 | 1.99900803 | 0.2751665 | -2.8609075 | 0.00564173 | 0.00766568 |
| IL2RB | 1.13719625 | 2.34112892 | 1.04172314 | 0.00086834 | 0.00129863 |
| TMEM44 | 0.86597471 | 2.07044197 | 1.25754197 | 4.82E-25 | 2.44E-23 |
| FAM110A | 1.69662783 | 3.66151984 | 1.10977249 | 3.40E-26 | 2.42E-24 |
| UGT2B27P | 2.77546316 | 1.20231328 | -1.2069157 | 5.30E-14 | 2.98E-13 |
| TOP2A | 5.02244967 | 11.8085133 | 1.23336423 | 3.53E-18 | 3.97E-17 |
| ZNF124 | 0.35643517 | 0.79009711 | 1.14839029 | 1.63E-16 | 1.36E-15 |
| LMNTD2-AS1 | 0.50789607 | 1.30299165 | 1.35922263 | 2.42E-08 | 6.26E-08 |
| QSOX1 | 5.3531264 | 21.5565412 | 2.00967209 | 1.66E-13 | 8.63E-13 |
| LRCOL1 | 3.91740073 | 1.68601195 | -1.216282 | 1.34E-07 | 3.17E-07 |
| TMIE | 0.84037612 | 1.80350203 | 1.10169398 | 8.77E-05 | 0.00014761 |
| AADAT | 3.15310634 | 1.36064986 | -1.212478 | 1.42E-08 | 3.78E-08 |
| PRR11 | 0.94402022 | 2.76650303 | 1.55117384 | 5.65E-25 | 2.82E-23 |
| SLC41A1 | 1.64878708 | 3.58689951 | 1.12133222 | 9.23E-14 | 4.97E-13 |
| LARP6 | 0.49092268 | 1.57263555 | 1.67961664 | 2.03E-12 | 9.01E-12 |
| S100P | 33.4397412 | 78.2152218 | 1.22588572 | 3.64E-09 | 1.05E-08 |
| GALNT3 | 0.16661378 | 1.27502906 | 2.93595051 | 3.39E-07 | 7.57E-07 |
| DOK1 | 0.9225318 | 2.18452067 | 1.24364622 | 2.51E-22 | 6.59E-21 |
| RPS3AP5 | 0.48216101 | 1.56412811 | 1.6977718 | 0.00034472 | 0.00053994 |
| IGHJ3 | 1.43585503 | 6.13833391 | 2.09593703 | 0.00270926 | 0.00381771 |
| IGLV2-18 | 0.48108497 | 1.65394907 | 1.78155118 | 0.00138874 | 0.00202985 |
| GMIP | 1.85173304 | 3.83916949 | 1.05191813 | 1.40E-16 | 1.18E-15 |
| PRODH | 2.44635796 | 0.78126446 | -1.6467526 | 0.00143374 | 0.00209379 |
| ZSWIM4 | 0.94453383 | 2.1812655 | 1.20749101 | 2.20E-12 | 9.69E-12 |
| PIP4P2 | 1.38117584 | 3.25127318 | 1.23510777 | 3.81E-13 | 1.88E-12 |
| MAT1A | 267.230797 | 126.066173 | -1.0839051 | 2.53E-22 | 6.63E-21 |
| SRPX2 | 1.17390342 | 4.5219248 | 1.94562328 | 1.28E-07 | 3.02E-07 |
| APCDD1 | 1.61606323 | 4.85987729 | 1.58843624 | 1.68E-12 | 7.56E-12 |
| DNMT3B | 0.46092192 | 1.07755852 | 1.22517194 | 1.18E-15 | 8.53E-15 |
| GLS | 2.84492058 | 6.15744315 | 1.11394302 | 1.03E-13 | 5.50E-13 |
| ZNF296 | 0.40411739 | 1.50645345 | 1.89830975 | 8.81E-30 | 2.26E-27 |
| CDT1 | 2.6103659 | 6.21699147 | 1.25196455 | 2.52E-21 | 5.28E-20 |
| CHST1 | 0.77079941 | 2.30897771 | 1.58282687 | 5.67E-11 | 2.03E-10 |
| AC026740.1 | 0.76112678 | 2.20240227 | 1.53286932 | 4.64E-14 | 2.63E-13 |
| OGDHL | 18.7878913 | 8.73946804 | -1.1041858 | 1.45E-14 | 8.86E-14 |
| FKBP10 | 3.50846833 | 15.5956258 | 2.1522282 | 1.86E-13 | 9.62E-13 |
| KIFC1 | 3.07058452 | 8.13394508 | 1.40544193 | 1.31E-21 | 2.94E-20 |
| IGLV3-25 | 7.21653232 | 27.2807005 | 1.91850303 | 0.00034531 | 0.00054081 |
| AL450998.1 | 1.5299783 | 3.09097276 | 1.01454975 | 4.71E-05 | 8.20E-05 |
| LIPH | 0.3216763 | 1.07009155 | 1.73405265 | 6.54E-05 | 0.000112 |
| HGD | 151.221869 | 72.1405619 | -1.0677842 | 3.75E-19 | 5.15E-18 |
| KNTC1 | 0.88025996 | 1.77928652 | 1.01529729 | 2.43E-15 | 1.67E-14 |
| FA2H | 0.25966112 | 1.34735604 | 2.37542925 | 7.36E-13 | 3.48E-12 |
| RAB34 | 3.27298364 | 11.5717623 | 1.82193029 | 6.43E-14 | 3.56E-13 |
| HLA-DQB1-AS1 | 0.44887971 | 1.03336067 | 1.20294308 | 1.06E-06 | 2.23E-06 |
| SLC7A5 | 3.52907446 | 7.23760059 | 1.03622163 | 9.70E-07 | 2.06E-06 |
| GABRP | 0.10308903 | 1.90578865 | 4.20842547 | 0.01163553 | 0.01517495 |
| BCAT1 | 0.35554322 | 1.27630974 | 1.84388163 | 1.27E-11 | 5.01E-11 |
| DEF6 | 1.65334709 | 4.05813791 | 1.29542827 | 2.01E-17 | 1.96E-16 |
| TPBG | 0.20530094 | 0.94755915 | 2.20647577 | 1.29E-12 | 5.92E-12 |
| DEFB132 | 1.09794279 | 0.38598818 | -1.5081743 | 4.62E-10 | 1.49E-09 |
| IGLV3-21 | 7.98760725 | 35.7777702 | 2.16322818 | 0.00015582 | 0.00025432 |
| TNFRSF21 | 5.48215311 | 16.3841712 | 1.57948817 | 1.24E-17 | 1.25E-16 |
| AC007663.1 | 0.8674516 | 0.36459369 | -1.2504937 | 0.00195399 | 0.00280249 |
| SH2D3A | 0.39627429 | 1.69374002 | 2.09564116 | 2.33E-15 | 1.61E-14 |
| LYPD1 | 2.01314018 | 4.75098933 | 1.23878033 | 1.34E-14 | 8.24E-14 |
| TSPO | 25.4059923 | 52.8324172 | 1.0562546 | 1.39E-07 | 3.26E-07 |
| PLG | 233.195204 | 101.567883 | -1.1990938 | 9.05E-23 | 2.66E-21 |
| CCND2 | 0.89846003 | 2.69713069 | 1.58589919 | 3.62E-08 | 9.16E-08 |
| CCND2P1 | 10.3973307 | 5.17476479 | -1.006648 | 2.88E-16 | 2.30E-15 |
| KIF3C | 0.31479239 | 1.47945191 | 2.23259025 | 2.06E-19 | 2.97E-18 |
| TMEM65 | 1.45511569 | 3.20415546 | 1.13881029 | 4.48E-16 | 3.46E-15 |
| MAPRE1 | 12.5754304 | 26.8009712 | 1.0916775 | 4.88E-30 | 1.46E-27 |
| AC012073.1 | 0.34183934 | 0.7914717 | 1.21121932 | 3.22E-25 | 1.70E-23 |
| DBN1 | 2.68879474 | 11.44484 | 2.08966577 | 2.29E-23 | 7.38E-22 |
| ZNF468 | 1.11852118 | 2.48287487 | 1.15041899 | 1.09E-12 | 5.06E-12 |
| EXPH5 | 0.68745284 | 0.32793131 | -1.0678671 | 1.77E-05 | 3.25E-05 |
| UBE2T | 6.00031724 | 13.4397325 | 1.16339374 | 2.48E-22 | 6.53E-21 |
| COL5A2 | 5.08064263 | 10.9882136 | 1.11287397 | 1.34E-08 | 3.58E-08 |
| SMYD3 | 0.85995764 | 1.82437047 | 1.08506122 | 2.72E-20 | 4.57E-19 |
| FCGBP | 0.47746574 | 2.24662589 | 2.23429079 | 4.18E-15 | 2.76E-14 |
| ACMSD | 26.5444267 | 13.2140793 | -1.0063331 | 2.35E-16 | 1.90E-15 |
| AP000240.1 | 0.42440782 | 0.95031453 | 1.16295383 | 7.38E-18 | 7.70E-17 |
| MYBL2 | 4.04295665 | 16.8707553 | 2.06104193 | 4.00E-30 | 1.25E-27 |
| MEAK7 | 0.38279082 | 0.88357635 | 1.20679859 | 1.61E-13 | 8.39E-13 |
| ZFPM2-AS1 | 0.98065191 | 2.22058678 | 1.17912791 | 0.01110865 | 0.01452678 |
| PLEKHO1 | 2.58588303 | 5.91383285 | 1.19343645 | 5.92E-19 | 7.82E-18 |
| LINC02768 | 2.04284536 | 0.60653129 | -1.751926 | 3.17E-14 | 1.83E-13 |
| GRAMD1B | 0.61690715 | 1.71187412 | 1.47245134 | 6.18E-10 | 1.96E-09 |
| GAS5 | 23.5824822 | 47.3489162 | 1.00561582 | 6.73E-16 | 5.07E-15 |
| AREG | 0.74758437 | 1.72325929 | 1.20483149 | 6.90E-08 | 1.69E-07 |
| F11 | 21.8415495 | 9.30854214 | -1.2304481 | 3.11E-27 | 3.11E-25 |
| KLK11 | 0.07479378 | 1.26512173 | 4.08021413 | 0.00038924 | 0.00060679 |
| SMTN | 2.11170193 | 4.25558137 | 1.01095003 | 1.84E-19 | 2.67E-18 |
| ZBTB16 | 1.79589097 | 0.820516 | -1.1300964 | 5.86E-14 | 3.27E-13 |
| LRRC56 | 0.35995111 | 0.80047864 | 1.15306193 | 4.15E-10 | 1.34E-09 |
| CENPA | 0.96417653 | 3.07262567 | 1.6721028 | 2.18E-25 | 1.21E-23 |
| TBX19 | 0.37874469 | 0.76670577 | 1.01744737 | 2.25E-25 | 1.24E-23 |
| ADAM9 | 3.6586611 | 8.25404565 | 1.17378563 | 7.72E-08 | 1.88E-07 |
| LIMD2 | 1.8771539 | 5.69457055 | 1.60104011 | 5.79E-21 | 1.11E-19 |
| ZIC2 | 1.28554844 | 2.76190939 | 1.10328202 | 4.04E-09 | 1.16E-08 |
| PDE5A | 0.30286115 | 0.80433832 | 1.40914593 | 1.91E-06 | 3.91E-06 |
| TUBA1C | 6.14960297 | 13.2990308 | 1.11275593 | 3.09E-19 | 4.31E-18 |
| Z97056.1 | 3.30719236 | 1.42617915 | -1.2134517 | 2.74E-09 | 8.03E-09 |
| PDGFRL | 0.48684172 | 1.31908244 | 1.43801003 | 6.63E-14 | 3.67E-13 |
| IGLV1-44 | 8.38423468 | 28.629778 | 1.77176548 | 8.37E-05 | 0.00014144 |
| SSTR5 | 0.497177 | 1.35483552 | 1.44628624 | 3.92E-07 | 8.71E-07 |
| PARPBP | 0.51094258 | 1.16870371 | 1.19367614 | 6.31E-19 | 8.27E-18 |
| MPP3 | 0.36573793 | 0.9210829 | 1.33252076 | 7.00E-18 | 7.34E-17 |
| LINC01480 | 0.16367078 | 1.99436196 | 3.60705857 | 1.61E-12 | 7.28E-12 |
| ASIC1 | 0.42294111 | 0.84821645 | 1.00397567 | 5.75E-11 | 2.06E-10 |
| CDCA2 | 0.40517038 | 1.14280296 | 1.49597606 | 7.09E-21 | 1.34E-19 |
| ADAMDEC1 | 0.41287784 | 1.46387778 | 1.8260082 | 2.72E-07 | 6.17E-07 |
| BORA | 0.39139049 | 0.80641911 | 1.04292112 | 1.44E-14 | 8.81E-14 |
| CORO1A | 3.44501601 | 8.468286 | 1.29755931 | 4.79E-12 | 2.01E-11 |
| ADA | 1.87299744 | 3.88846825 | 1.05385303 | 1.92E-21 | 4.14E-20 |
| SLC28A1 | 14.258124 | 6.05698018 | -1.2351136 | 2.01E-14 | 1.20E-13 |
| VEPH1 | 0.0979984 | 1.27251682 | 3.69878277 | 1.69E-16 | 1.40E-15 |
| LINC02732 | 0.92349255 | 0.26263418 | -1.8140456 | 2.18E-11 | 8.26E-11 |
| RACGAP1 | 2.50662942 | 5.4414609 | 1.11824531 | 1.17E-19 | 1.77E-18 |
| LINC02298 | 0.45357774 | 1.08301206 | 1.25562757 | 1.94E-06 | 3.96E-06 |
| IGHG4 | 13.8235052 | 53.7579745 | 1.9593553 | 2.32E-07 | 5.31E-07 |
| KAZALD1 | 0.60253709 | 1.39000297 | 1.20596601 | 1.55E-07 | 3.64E-07 |
| ENPP5 | 0.40137438 | 1.49830369 | 1.90030964 | 5.69E-08 | 1.40E-07 |
| MAP3K21 | 0.78443511 | 1.88184715 | 1.26242343 | 1.71E-05 | 3.15E-05 |
| C2CD4A | 1.09156795 | 2.76456608 | 1.34065112 | 0.02688914 | 0.03345148 |
| PFKP | 1.89623877 | 9.35978561 | 2.30333485 | 4.00E-12 | 1.70E-11 |
| MSX1 | 0.69615162 | 2.02215681 | 1.53842141 | 1.08E-09 | 3.34E-09 |
| LARGE2 | 1.66930099 | 3.93968008 | 1.23883437 | 1.04E-07 | 2.48E-07 |
| DNM1 | 0.35881137 | 1.75885819 | 2.29334166 | 2.95E-18 | 3.39E-17 |
| NPTX2 | 1.97261114 | 9.96006149 | 2.33604807 | 1.34E-07 | 3.16E-07 |
| CXCR3 | 0.69528259 | 1.63624436 | 1.23471686 | 5.53E-07 | 1.21E-06 |
| SPATS2 | 1.90348399 | 3.81788284 | 1.00413039 | 8.87E-23 | 2.61E-21 |
| IGKV1-9 | 4.53681761 | 28.4856902 | 2.6504848 | 0.00167251 | 0.00242241 |
| VANGL2 | 0.32109282 | 1.13075595 | 1.81622527 | 8.19E-12 | 3.32E-11 |
| KIF7 | 0.47393622 | 0.96356266 | 1.02368558 | 5.92E-13 | 2.84E-12 |
| C16orf89 | 0.15000072 | 3.88291855 | 4.69410006 | 3.71E-08 | 9.35E-08 |
| USH1C | 2.21635693 | 5.16730523 | 1.22122187 | 9.29E-10 | 2.90E-09 |
| CCDC183 | 0.29416759 | 0.91810104 | 1.64201463 | 6.46E-16 | 4.88E-15 |
| CCNF | 1.01333482 | 2.16978253 | 1.09843951 | 1.80E-21 | 3.89E-20 |
| ABCB11 | 9.548306 | 2.42818607 | -1.9753658 | 2.89E-21 | 5.95E-20 |
| TMEM100 | 1.81640508 | 0.6031566 | -1.5904814 | 3.88E-08 | 9.76E-08 |
| SPP1 | 234.856806 | 505.337041 | 1.10546453 | 1.33E-06 | 2.77E-06 |
| SMOX | 2.51155243 | 6.77814127 | 1.43231031 | 3.12E-22 | 8.02E-21 |
| DCAF16 | 1.98857084 | 4.12134581 | 1.05138357 | 1.15E-19 | 1.74E-18 |
| ZNF813 | 0.42459492 | 0.94331298 | 1.15164942 | 1.20E-14 | 7.44E-14 |
| PCSK1N | 0.89374166 | 8.41676271 | 3.23533566 | 1.68E-15 | 1.18E-14 |
| LBH | 4.23466537 | 8.65138996 | 1.03068397 | 1.15E-08 | 3.10E-08 |
| SERPINE2 | 1.67145812 | 5.74533305 | 1.78128332 | 6.43E-10 | 2.04E-09 |
| C3orf85 | 0.82369313 | 0.3027501 | -1.4439795 | 1.53E-13 | 7.98E-13 |
| OSBPL3 | 0.76710895 | 1.82850266 | 1.25315933 | 2.65E-17 | 2.53E-16 |
| WDHD1 | 0.51068106 | 1.14030437 | 1.15892452 | 2.79E-17 | 2.64E-16 |
| EGFR-AS1 | 0.38921884 | 1.50578275 | 1.95186018 | 4.46E-06 | 8.79E-06 |
| BASP1 | 2.76178196 | 8.48199494 | 1.6188042 | 9.61E-08 | 2.31E-07 |
| CD53 | 5.52616299 | 11.7014601 | 1.08233854 | 3.44E-06 | 6.86E-06 |
| COTL1 | 4.18704141 | 9.74122058 | 1.21817137 | 9.36E-13 | 4.38E-12 |
| CNKSR1 | 0.33012288 | 1.18686641 | 1.84608252 | 5.57E-09 | 1.57E-08 |
| ALDH6A1 | 38.0883746 | 14.1542376 | -1.4281167 | 3.93E-29 | 7.49E-27 |
| AL772337.3 | 0.83033328 | 2.04422841 | 1.29979398 | 4.46E-08 | 1.11E-07 |
| AC005332.4 | 0.37717526 | 0.80289571 | 1.08997756 | 1.37E-15 | 9.78E-15 |
| SLC27A5 | 69.0209181 | 21.3685864 | -1.6915422 | 1.74E-26 | 1.35E-24 |
| DACT2 | 1.18534537 | 3.02624927 | 1.35222335 | 0.00012572 | 0.00020772 |
| DTNBP1 | 2.65191899 | 5.35436776 | 1.01367953 | 1.19E-27 | 1.31E-25 |
| AC008610.1 | 0.6225042 | 1.63415624 | 1.39239045 | 1.57E-17 | 1.56E-16 |
| SH2D2A | 0.7433354 | 1.86650769 | 1.32825624 | 3.82E-14 | 2.19E-13 |
| IGHV3-15 | 5.74925622 | 14.4426743 | 1.32889067 | 0.00283343 | 0.00398473 |
| TMEM156 | 1.52829038 | 3.18601765 | 1.05983558 | 1.68E-07 | 3.91E-07 |
| PIWIL4 | 0.17906203 | 0.99917549 | 2.48027862 | 5.70E-12 | 2.37E-11 |
| FAAP24 | 0.78922028 | 1.66482411 | 1.07686984 | 1.12E-25 | 6.98E-24 |
| CTSE | 0.72954211 | 3.61653263 | 2.309544 | 1.35E-08 | 3.62E-08 |
| IGHV3-20 | 0.33485377 | 2.2495779 | 2.7480512 | 0.00483709 | 0.00661719 |
| LINC01436 | 0.74792025 | 3.11562285 | 2.05856425 | 0.00033534 | 0.00052601 |
| ADM2 | 3.02156566 | 6.22542949 | 1.04287708 | 1.45E-12 | 6.60E-12 |
| CHST11 | 1.12679446 | 3.12941603 | 1.47366909 | 8.55E-14 | 4.62E-13 |
| RBP2 | 0.34890838 | 3.01507964 | 3.11127596 | 5.69E-10 | 1.82E-09 |
| AC010247.2 | 0.32102736 | 1.54266251 | 2.26465432 | 2.61E-14 | 1.53E-13 |
| AC107959.3 | 0.3060642 | 0.80336429 | 1.39222005 | 2.01E-12 | 8.91E-12 |
| MST1R | 0.39007242 | 1.15125377 | 1.56139197 | 5.90E-06 | 1.14E-05 |
| TLCD5 | 0.46430404 | 1.11833607 | 1.26821205 | 9.01E-21 | 1.66E-19 |
| CMTM4 | 1.1874318 | 2.52619778 | 1.08912294 | 7.09E-09 | 1.97E-08 |
| C7orf31 | 0.49143325 | 1.27333229 | 1.37354157 | 5.10E-20 | 8.17E-19 |
| TNFAIP8L3 | 1.07576658 | 2.25802087 | 1.06969374 | 1.70E-11 | 6.57E-11 |
| IQGAP1 | 2.51226172 | 5.50894089 | 1.13278821 | 3.39E-10 | 1.11E-09 |
| TPSB2 | 0.95865203 | 2.90949467 | 1.60168946 | 0.0037173 | 0.00514611 |
| PALM | 1.95330683 | 5.48888968 | 1.49059575 | 1.30E-11 | 5.10E-11 |
| TEAD2 | 4.21157399 | 12.5869078 | 1.57949248 | 1.81E-23 | 5.94E-22 |
| TRIM47 | 6.99078125 | 15.9586977 | 1.19081732 | 1.44E-16 | 1.21E-15 |
| CD3D | 3.28278514 | 8.2375379 | 1.32729287 | 2.82E-10 | 9.27E-10 |
| E2F5 | 0.70153551 | 1.44011772 | 1.0375987 | 1.12E-12 | 5.16E-12 |
| EMB | 0.69462325 | 1.70243167 | 1.29329428 | 1.50E-06 | 3.10E-06 |
| PGAP4 | 0.98123394 | 2.81798655 | 1.52199569 | 5.19E-11 | 1.87E-10 |
| ST14 | 9.52297012 | 21.513589 | 1.17576471 | 2.68E-05 | 4.81E-05 |
| TUSC3 | 1.56547696 | 3.81524383 | 1.28517298 | 1.31E-05 | 2.44E-05 |
| GPT2 | 36.4031559 | 17.7585692 | -1.0355482 | 1.25E-17 | 1.26E-16 |
| CNTNAP1 | 0.44261068 | 1.28673458 | 1.53960431 | 1.52E-15 | 1.08E-14 |
| AC026765.3 | 1.07762267 | 0.50680464 | -1.0883505 | 0.00055084 | 0.00084167 |
| CRHBP | 1.75579139 | 0.62132902 | -1.4986921 | 0.000594 | 0.00090535 |
| MECOM | 0.57139098 | 1.15244692 | 1.01215014 | 0.0115034 | 0.01501235 |
| ALOX5AP | 1.3143315 | 3.83232808 | 1.54389188 | 2.69E-11 | 1.01E-10 |
| H4C5 | 0.46228619 | 0.9981987 | 1.11054076 | 5.34E-08 | 1.32E-07 |
| AL391056.1 | 0.37231459 | 1.14615994 | 1.62221433 | 3.05E-10 | 1.00E-09 |
| MEX3A | 0.62468651 | 2.08083565 | 1.73595875 | 1.18E-16 | 1.01E-15 |
| ARHGEF39 | 0.55753556 | 1.31984035 | 1.24322771 | 3.47E-25 | 1.80E-23 |
| IGHV1-69D | 4.23530403 | 13.2057324 | 1.64062687 | 0.00492634 | 0.00673324 |
| GALNT7 | 0.35253335 | 1.02360756 | 1.53783107 | 1.12E-07 | 2.66E-07 |
| PLTP | 7.97296178 | 25.5038742 | 1.67752876 | 7.13E-11 | 2.53E-10 |
| BUB1 | 0.94670455 | 2.33273583 | 1.30103678 | 4.50E-19 | 6.09E-18 |
| UAP1L1 | 0.72885216 | 3.67234378 | 2.33300302 | 1.69E-20 | 2.94E-19 |
| LPAR2 | 0.70080144 | 3.62822075 | 2.3721846 | 1.52E-24 | 6.64E-23 |
| C4BPA | 563.771143 | 241.725502 | -1.2217399 | 1.44E-17 | 1.44E-16 |
| LPCAT1 | 4.07536584 | 10.9707564 | 1.42866152 | 3.41E-20 | 5.64E-19 |
| MFSD6 | 1.35598977 | 3.20435985 | 1.24068988 | 1.46E-16 | 1.22E-15 |
| MTHFD2 | 0.79127241 | 1.8486038 | 1.22418969 | 1.85E-12 | 8.24E-12 |
| SNHG7 | 4.21998893 | 9.90355547 | 1.23070735 | 2.77E-24 | 1.12E-22 |
| KIF18A | 0.38205768 | 1.06305606 | 1.4763553 | 8.00E-20 | 1.24E-18 |
| CLDN4 | 5.23510561 | 15.7143997 | 1.58579662 | 5.41E-08 | 1.34E-07 |
| SLC1A7 | 1.01054319 | 3.79180446 | 1.90775359 | 3.21E-06 | 6.43E-06 |
| AC010336.2 | 0.93686091 | 0.33353686 | -1.4899887 | 1.48E-16 | 1.24E-15 |
| LDOC1 | 1.70722206 | 8.59008056 | 2.33102093 | 2.09E-10 | 6.97E-10 |
| EIF5A2 | 0.90166457 | 1.98818338 | 1.1407881 | 1.36E-09 | 4.16E-09 |
| LDLRAD3 | 0.54140036 | 1.26728913 | 1.22697796 | 2.75E-07 | 6.24E-07 |
| GZMB | 0.97822733 | 2.10813346 | 1.10772453 | 0.00258605 | 0.00365506 |
| LINC02365 | 2.05697114 | 4.20050822 | 1.03004234 | 3.18E-06 | 6.36E-06 |
| IFT57 | 1.46514305 | 3.29207471 | 1.16795555 | 1.11E-15 | 8.02E-15 |
| MIR4653 | 0.69224247 | 1.39226492 | 1.00808439 | 1.39E-05 | 2.58E-05 |
| TOR4A | 1.17696806 | 3.27557508 | 1.47667305 | 1.24E-12 | 5.68E-12 |
| SPRED1 | 1.10354939 | 2.35126737 | 1.09128741 | 2.26E-11 | 8.55E-11 |
| HSD17B6 | 228.331313 | 83.2049801 | -1.4563869 | 2.39E-28 | 3.37E-26 |
| SEL1L3 | 2.53604222 | 10.8319593 | 2.09464355 | 2.91E-14 | 1.69E-13 |
| SASH3 | 1.8989371 | 4.23669347 | 1.15774663 | 2.20E-08 | 5.74E-08 |
| RASSF9 | 0.31485133 | 0.91803424 | 1.54387719 | 0.01237685 | 0.01609116 |
| SOX12 | 4.1975119 | 8.83111188 | 1.07306068 | 1.52E-18 | 1.86E-17 |
| IGHV3-72 | 0.56155561 | 1.66109922 | 1.56463745 | 0.00092054 | 0.0013735 |
| CCL20 | 21.4842055 | 43.6640161 | 1.0231684 | 2.87E-09 | 8.39E-09 |
| COLCA2 | 0.81886715 | 2.71331458 | 1.72835499 | 4.36E-23 | 1.35E-21 |
| HPSE | 0.31465719 | 0.77958522 | 1.30892584 | 1.01E-12 | 4.70E-12 |
| EMILIN2 | 0.68487893 | 1.82901027 | 1.41714228 | 2.50E-19 | 3.54E-18 |
| CDC6 | 1.80094685 | 4.52726556 | 1.32988433 | 1.10E-20 | 1.99E-19 |
| PRR7 | 1.08885311 | 2.53668578 | 1.22013549 | 5.27E-18 | 5.70E-17 |
| STK17A | 2.70300063 | 5.4182885 | 1.00327537 | 1.77E-22 | 4.85E-21 |
| AC002398.1 | 0.56543883 | 1.37660654 | 1.28367342 | 9.66E-25 | 4.45E-23 |
| IGLV1-40 | 20.643492 | 41.7225733 | 1.01514111 | 0.000889 | 0.00132757 |
| CLDN10 | 0.50736788 | 4.35731743 | 3.10233613 | 0.00059396 | 0.00090535 |
| DCXR-DT | 6.87209294 | 2.91325919 | -1.2381155 | 5.90E-14 | 3.30E-13 |
| MDK | 51.9087045 | 116.982585 | 1.17224539 | 1.80E-12 | 8.02E-12 |
| TM4SF1 | 21.6023478 | 55.9511864 | 1.37298061 | 1.37E-08 | 3.66E-08 |
| GLYAT | 30.8991885 | 7.7419599 | -1.9967982 | 3.11E-24 | 1.25E-22 |
| LGALS1 | 74.5373818 | 163.709954 | 1.135106 | 1.24E-11 | 4.88E-11 |
| EPPK1 | 0.48519904 | 1.14747379 | 1.24181259 | 5.11E-08 | 1.27E-07 |
| OPN1SW | 0.98625028 | 2.06618017 | 1.06694035 | 2.63E-15 | 1.80E-14 |
| CIP2A | 0.49809859 | 1.34083753 | 1.4286312 | 4.39E-20 | 7.09E-19 |
| AC079360.1 | 2.08069778 | 0.46874544 | -2.1501909 | 1.71E-17 | 1.68E-16 |
| TMSB4X | 223.170306 | 478.951388 | 1.10173415 | 1.00E-18 | 1.27E-17 |
| BACE2 | 1.83361055 | 7.96246474 | 2.11852783 | 3.12E-09 | 9.07E-09 |
| SRD5A2 | 5.66430731 | 2.15579291 | -1.3936809 | 2.31E-11 | 8.72E-11 |
| AC008549.1 | 14.6725539 | 6.02635121 | -1.2837633 | 1.01E-10 | 3.51E-10 |
| PPP1R37 | 3.01118061 | 6.13445736 | 1.02660649 | 1.58E-25 | 9.29E-24 |
| TCF3 | 4.10916407 | 8.77677495 | 1.09484598 | 5.23E-30 | 1.54E-27 |
| CCDC88C | 0.46287118 | 1.04502784 | 1.17485875 | 6.98E-12 | 2.86E-11 |
| TMSB10P1 | 0.28988724 | 0.83372786 | 1.52408472 | 1.31E-12 | 5.99E-12 |
| PRMT1 | 9.16487875 | 19.7046292 | 1.1043469 | 2.94E-37 | 1.04E-33 |
| HLF | 17.2718974 | 8.50570697 | -1.0219235 | 1.75E-09 | 5.28E-09 |
| CCL28 | 0.99579469 | 2.37170965 | 1.25200717 | 4.22E-05 | 7.40E-05 |
| PTN | 1.03578092 | 3.85638005 | 1.89652834 | 0.00299715 | 0.00419779 |
| IER5L | 2.23090077 | 5.805502 | 1.37979448 | 1.29E-16 | 1.09E-15 |
| MGAT5 | 2.03898198 | 4.19457739 | 1.04067644 | 1.32E-09 | 4.04E-09 |
| CLEC2D | 0.40973665 | 0.82384349 | 1.00767335 | 1.19E-09 | 3.67E-09 |
| HP | 1705.13447 | 685.730148 | -1.3141727 | 2.05E-16 | 1.68E-15 |
| AL035446.1 | 1.07392514 | 2.47434323 | 1.20415221 | 0.02092306 | 0.02645076 |
| HAO1 | 122.218837 | 50.0343192 | -1.2884768 | 2.66E-29 | 5.67E-27 |
| SKA1 | 1.13144246 | 3.31342609 | 1.55016052 | 3.16E-20 | 5.25E-19 |
| SHCBP1 | 0.6380483 | 1.60522477 | 1.33103778 | 2.12E-18 | 2.51E-17 |
| PAX8 | 0.37252863 | 0.81938532 | 1.13719074 | 7.11E-07 | 1.53E-06 |
| MMP1 | 0.46966846 | 4.26911488 | 3.18422238 | 1.51E-11 | 5.87E-11 |
| AC021146.9 | 14.412747 | 4.22118801 | -1.7716243 | 3.86E-21 | 7.74E-20 |
| MT2P1 | 6.71246113 | 2.67064362 | -1.3296544 | 0.00102147 | 0.00151719 |
| LY96 | 9.86510814 | 19.7405916 | 1.00075845 | 2.83E-07 | 6.40E-07 |
| PACSIN1 | 0.26969739 | 1.04739477 | 1.95739184 | 9.20E-18 | 9.47E-17 |
| PON3 | 39.6871097 | 17.9532941 | -1.1444219 | 3.65E-21 | 7.37E-20 |
| CYP2C9 | 158.092575 | 60.637635 | -1.3824842 | 5.71E-18 | 6.12E-17 |
| LPA | 4.49466908 | 2.18453309 | -1.0408899 | 2.43E-09 | 7.18E-09 |
| CTNND2 | 0.39555296 | 3.75826325 | 3.24812334 | 6.77E-17 | 5.99E-16 |
| ZNF793-AS1 | 0.35504414 | 0.8584436 | 1.27372496 | 1.92E-05 | 3.50E-05 |
| FCGR3A | 7.78271887 | 15.7485053 | 1.01686876 | 9.61E-05 | 0.00016093 |
| ABCB4 | 22.6975602 | 6.63297624 | -1.774809 | 3.87E-30 | 1.24E-27 |
| GPC5 | 0.45407774 | 0.91331403 | 1.00817169 | 0.00028888 | 0.00045705 |
| HPX | 736.28649 | 322.404422 | -1.1913957 | 1.49E-22 | 4.20E-21 |
| PTGER4 | 0.59965843 | 1.4140684 | 1.23763903 | 2.18E-09 | 6.47E-09 |
| LILRB4 | 0.68624602 | 1.7573731 | 1.35662273 | 2.79E-09 | 8.17E-09 |
| TMEM158 | 0.30088538 | 2.04367865 | 2.76388245 | 1.81E-13 | 9.35E-13 |
| SOD3 | 4.32247797 | 16.2402888 | 1.90964677 | 5.18E-09 | 1.46E-08 |
| RAB3IL1 | 1.77353897 | 5.38037725 | 1.60107631 | 1.28E-23 | 4.38E-22 |
| GLIS2 | 1.83086405 | 4.77695129 | 1.3835655 | 6.42E-13 | 3.06E-12 |
| CTBP2 | 0.46327322 | 1.50762451 | 1.70234195 | 5.63E-08 | 1.39E-07 |
| BAAT | 230.443875 | 107.52142 | -1.0997913 | 3.50E-20 | 5.78E-19 |
| TEAD4 | 1.49479808 | 4.20218689 | 1.49118971 | 4.10E-14 | 2.34E-13 |
| CENPK | 0.454561 | 1.04239836 | 1.19736089 | 8.70E-17 | 7.56E-16 |
| PRAME | 0.9949433 | 6.15479815 | 2.62902533 | 1.36E-18 | 1.68E-17 |
| POSTN | 3.01141673 | 6.35338024 | 1.077082 | 4.22E-06 | 8.32E-06 |
| XDH | 12.3550247 | 5.59177644 | -1.1437193 | 2.22E-12 | 9.75E-12 |
| AMIGO2 | 0.89143561 | 2.24652032 | 1.33348961 | 8.26E-08 | 2.00E-07 |
| NPNT | 1.62948973 | 6.05944182 | 1.89476465 | 2.04E-05 | 3.71E-05 |
| IGKV2-30 | 0.3570473 | 1.48090094 | 2.05228803 | 0.01878965 | 0.02389946 |
| CD300LF | 0.50785743 | 1.09559951 | 1.10922507 | 1.07E-11 | 4.25E-11 |
| HLA-DRB1 | 125.538815 | 258.169991 | 1.04018782 | 6.07E-06 | 1.18E-05 |
| TRIM50 | 1.76833444 | 4.62589078 | 1.38734005 | 3.98E-07 | 8.82E-07 |
| SULT1B1 | 3.56042635 | 1.33513291 | -1.4150666 | 8.34E-15 | 5.27E-14 |
| PLP2 | 19.6847338 | 53.0491852 | 1.43025339 | 2.67E-17 | 2.54E-16 |
| MMD | 3.93939257 | 9.08233116 | 1.20508945 | 3.99E-24 | 1.56E-22 |
| CDC42BPG | 0.5314379 | 1.69282385 | 1.67145883 | 1.82E-07 | 4.23E-07 |
| TM6SF2 | 8.18035618 | 3.90179279 | -1.0680265 | 1.33E-17 | 1.34E-16 |
| CTLA4 | 0.36220358 | 0.89609037 | 1.30684343 | 1.40E-11 | 5.48E-11 |
| AL663070.1 | 0.76775373 | 0.20220023 | -1.924859 | 0.00030057 | 0.00047417 |
| NKD2 | 0.65726829 | 1.60639279 | 1.28927042 | 0.00040064 | 0.00062353 |
| IQANK1 | 0.28138128 | 1.14207381 | 2.02105766 | 5.03E-05 | 8.74E-05 |
| MCM10 | 0.48159767 | 1.2747821 | 1.40435034 | 4.78E-20 | 7.66E-19 |
| OSCAR | 0.51129836 | 1.17168048 | 1.19634191 | 3.32E-12 | 1.43E-11 |
| SIRPG | 0.42479479 | 1.14371762 | 1.42889293 | 3.76E-10 | 1.22E-09 |
| F13B | 59.7123597 | 27.1462328 | -1.1372776 | 1.10E-20 | 1.99E-19 |
| KCNK5 | 2.75363807 | 6.55146021 | 1.25047755 | 0.00162822 | 0.00236166 |
| AC004832.5 | 1.55601371 | 0.5694377 | -1.4502448 | 1.80E-18 | 2.17E-17 |
| IGKV1-12 | 0.19643782 | 1.10714157 | 2.49469505 | 0.00171362 | 0.00247763 |
| PLEKHG4 | 0.21172864 | 0.94313432 | 2.1552468 | 1.43E-16 | 1.20E-15 |
| CMTM3 | 3.07380436 | 8.57792275 | 1.48060298 | 7.64E-24 | 2.81E-22 |
| FCER1G | 12.9031989 | 36.6436529 | 1.50583456 | 1.43E-12 | 6.51E-12 |
| RABL6 | 2.12710807 | 4.95859758 | 1.22103881 | 2.48E-32 | 1.52E-29 |
| STK39 | 2.01629114 | 4.67385448 | 1.21290884 | 1.20E-15 | 8.66E-15 |
| TMEM82 | 13.036463 | 5.86898641 | -1.1513692 | 1.23E-08 | 3.30E-08 |
| CYP4A22 | 27.816634 | 8.60761441 | -1.6922625 | 6.41E-25 | 3.14E-23 |
| SEZ6 | 0.62662032 | 2.88331581 | 2.20206541 | 0.00033754 | 0.0005294 |
| ZNF83 | 1.18822489 | 2.928627 | 1.30141655 | 6.64E-12 | 2.73E-11 |
| AC084375.1 | 5.30028308 | 2.1396583 | -1.308689 | 0.02789474 | 0.0346338 |
| SLC1A5 | 3.47105578 | 16.6687487 | 2.26369935 | 3.79E-26 | 2.66E-24 |
| CDC20 | 5.35898453 | 18.0728738 | 1.75379437 | 3.63E-24 | 1.43E-22 |
| UGT1A4 | 33.4677015 | 9.57544708 | -1.8053577 | 2.80E-16 | 2.24E-15 |
| OIP5 | 1.30364504 | 2.84089537 | 1.1237946 | 1.14E-17 | 1.15E-16 |
| AL390728.5 | 2.55516577 | 6.03092412 | 1.23896219 | 2.25E-22 | 5.95E-21 |
| TMEM119 | 1.040712 | 2.4184679 | 1.2165225 | 3.29E-06 | 6.59E-06 |
| MYLK-AS1 | 0.47945335 | 1.01597826 | 1.08340717 | 2.06E-18 | 2.46E-17 |
| FCGR2A | 1.68165775 | 3.45171205 | 1.037428 | 2.41E-10 | 8.00E-10 |
| MICAL1 | 1.36085005 | 3.65400126 | 1.42496903 | 5.64E-23 | 1.71E-21 |
| RASSF8 | 0.76995866 | 1.91465112 | 1.31422863 | 4.84E-06 | 9.49E-06 |
| CA9 | 2.41910392 | 23.935168 | 3.30658728 | 9.91E-23 | 2.88E-21 |
| H2AX | 11.004681 | 27.8116282 | 1.33757088 | 3.17E-33 | 2.63E-30 |
| DRAM1 | 2.56218492 | 5.80125356 | 1.17899008 | 2.42E-20 | 4.11E-19 |
| RIN1 | 0.43899965 | 1.06959354 | 1.28477098 | 9.52E-14 | 5.12E-13 |
| RCC2 | 8.0527874 | 18.7222805 | 1.21719603 | 1.55E-29 | 3.63E-27 |
| LAT2 | 1.07782906 | 2.32584656 | 1.10962754 | 2.27E-12 | 9.96E-12 |
| CHGA | 0.12643398 | 5.19206763 | 5.35985302 | 0.02047672 | 0.02591438 |
| NBL1 | 1.11207159 | 3.59978503 | 1.69466109 | 6.19E-13 | 2.96E-12 |
| PKDCC | 3.38976383 | 8.66857477 | 1.35461005 | 5.28E-15 | 3.44E-14 |
| GPC3 | 172.676399 | 411.900418 | 1.25422468 | 8.34E-09 | 2.30E-08 |
| ATP5MFP2 | 0.38561072 | 1.7271332 | 2.1631623 | 0.02238144 | 0.02816812 |
| AC124798.1 | 0.31927402 | 1.02918701 | 1.68863808 | 6.14E-09 | 1.72E-08 |
| SLC45A4 | 0.78910066 | 2.19237886 | 1.47421589 | 1.04E-11 | 4.16E-11 |
| P3H3 | 0.69587287 | 2.13554684 | 1.61770986 | 2.97E-09 | 8.66E-09 |
| SPINK4 | 0.22409066 | 0.99497014 | 2.15057071 | 1.41E-07 | 3.31E-07 |
| GPSM3 | 4.37679427 | 8.95092386 | 1.03216203 | 2.98E-12 | 1.29E-11 |
| NCK2 | 4.20483088 | 13.6085992 | 1.69439889 | 1.02E-20 | 1.86E-19 |
| HS6ST2 | 0.39271669 | 0.91460972 | 1.21966734 | 1.66E-15 | 1.17E-14 |
| SPDYC | 3.66679375 | 1.29165899 | -1.5052939 | 5.28E-15 | 3.44E-14 |
| SLC34A2 | 0.23643769 | 13.4907768 | 5.83436956 | 1.88E-09 | 5.65E-09 |
| FOLH1B | 1.51979967 | 0.32294341 | -2.2345279 | 1.47E-10 | 5.01E-10 |
| MIR4292 | 1.43062739 | 2.90246385 | 1.02063013 | 3.68E-08 | 9.29E-08 |
| BAK1 | 5.14537848 | 10.8464711 | 1.07587663 | 2.46E-26 | 1.84E-24 |
| RECQL4 | 3.51401371 | 7.91890866 | 1.1721818 | 5.61E-19 | 7.45E-18 |
| AC016739.1 | 4.43540128 | 9.39128219 | 1.08225751 | 1.53E-07 | 3.58E-07 |
| MUC1 | 0.36538164 | 4.61464982 | 3.65874513 | 3.38E-13 | 1.67E-12 |
| IGHV5-51 | 8.60484516 | 21.7979781 | 1.34097319 | 0.00193582 | 0.00277867 |
| EVC | 0.86931621 | 2.40068601 | 1.46549377 | 1.13E-11 | 4.49E-11 |
| LINC01857 | 0.32357729 | 0.85582599 | 1.40320712 | 1.18E-06 | 2.47E-06 |
| SLC25A24 | 0.69438925 | 1.59302949 | 1.19795645 | 2.86E-06 | 5.75E-06 |
| FMO4 | 12.1622591 | 5.58620833 | -1.1224699 | 4.63E-19 | 6.26E-18 |
| PPP1R13L | 2.17015688 | 4.75969392 | 1.13306946 | 8.33E-11 | 2.93E-10 |
| CSF2RA | 0.68116655 | 1.38220157 | 1.02088852 | 4.32E-11 | 1.57E-10 |
| HLA-DQB1 | 9.41030359 | 19.8139494 | 1.0742033 | 3.79E-05 | 6.68E-05 |
| IGLV4-69 | 4.64096402 | 20.8493849 | 2.16750841 | 0.00190901 | 0.00274186 |
| TMPRSS3 | 1.01154184 | 3.24166749 | 1.68018012 | 3.03E-08 | 7.73E-08 |
| EHHADH | 56.3750994 | 20.9299585 | -1.4294886 | 7.70E-29 | 1.28E-26 |
| NMB | 2.69543729 | 8.05800478 | 1.57990332 | 3.87E-21 | 7.74E-20 |
| RFC4 | 3.93203935 | 7.9790967 | 1.02094767 | 1.33E-23 | 4.52E-22 |
| HCK | 2.01236802 | 4.2707631 | 1.0855997 | 1.30E-08 | 3.50E-08 |
| TYRO3 | 0.43710015 | 1.97113106 | 2.17298791 | 4.78E-27 | 4.40E-25 |
| NRSN2 | 2.86463266 | 8.49436642 | 1.5681562 | 1.36E-18 | 1.68E-17 |
| NFKBID | 0.40590369 | 0.85751688 | 1.07902759 | 2.65E-18 | 3.07E-17 |
| AC022415.1 | 0.41354951 | 0.83836677 | 1.01952148 | 1.67E-11 | 6.45E-11 |
| RGS1 | 3.21366674 | 8.09012739 | 1.33194209 | 1.69E-11 | 6.53E-11 |
| SUZ12P1 | 0.65355519 | 1.40969589 | 1.10900299 | 7.94E-30 | 2.07E-27 |
| S100A6 | 50.7547349 | 171.839029 | 1.75944342 | 2.26E-15 | 1.56E-14 |
| DAGLA | 0.6034079 | 1.45022541 | 1.26507168 | 1.45E-14 | 8.86E-14 |
| TCEAL9 | 14.6430175 | 32.7662178 | 1.16199627 | 8.62E-20 | 1.33E-18 |
| GUCA2A | 0.36479436 | 2.82468072 | 2.95293247 | 5.46E-07 | 1.19E-06 |
| VTCN1 | 0.34012438 | 6.37225086 | 4.22766874 | 6.18E-08 | 1.52E-07 |
| CXCL14 | 0.97266952 | 4.29723492 | 2.14338702 | 9.14E-06 | 1.73E-05 |
| LCK | 1.0577113 | 2.30878996 | 1.12619104 | 1.86E-06 | 3.82E-06 |
| CCR7 | 0.47749876 | 1.1433546 | 1.25970403 | 0.00662013 | 0.00890581 |
| ZNF731P | 2.58836891 | 5.74080654 | 1.14921019 | 4.53E-11 | 1.64E-10 |
| GMNC | 0.69710415 | 0.29302594 | -1.2503458 | 8.14E-14 | 4.43E-13 |
| ANKRD13B | 0.40761927 | 1.03823205 | 1.34883476 | 1.28E-16 | 1.08E-15 |
| GLS2 | 2.59672606 | 0.57927926 | -2.1643629 | 6.82E-09 | 1.90E-08 |
| DEFB1 | 155.942133 | 357.032808 | 1.19504588 | 0.0069301 | 0.00929447 |
| SGO1 | 0.57121756 | 1.3036572 | 1.19045232 | 1.23E-16 | 1.05E-15 |
| SOBP | 0.59672909 | 1.89604625 | 1.66784615 | 5.47E-09 | 1.54E-08 |
| MYL9 | 16.2525598 | 64.3049078 | 1.98426189 | 2.80E-06 | 5.64E-06 |
| PYCR1 | 4.627662 | 13.4169666 | 1.53570314 | 5.98E-11 | 2.14E-10 |
| SNRPB | 80.5864291 | 167.747833 | 1.05768532 | 5.27E-31 | 2.32E-28 |
| TPRN | 4.57102017 | 9.66154723 | 1.07973806 | 5.72E-17 | 5.12E-16 |
| FMO1 | 0.99929656 | 2.62999307 | 1.3960742 | 1.96E-08 | 5.14E-08 |
| FCHO1 | 0.25136845 | 1.04404364 | 2.05430653 | 5.61E-18 | 6.03E-17 |
| AC011468.1 | 0.80595147 | 1.68241112 | 1.06176541 | 2.03E-18 | 2.42E-17 |
| PDLIM7 | 3.31519773 | 8.00822093 | 1.27238685 | 1.36E-25 | 8.31E-24 |
| NCF1C | 0.44228427 | 1.0981949 | 1.31208828 | 1.28E-10 | 4.37E-10 |
| IGKV3-11 | 11.8863481 | 57.6171768 | 2.27719343 | 4.36E-05 | 7.64E-05 |
| TTC36 | 18.9236753 | 2.80024266 | -2.7565686 | 1.66E-22 | 4.59E-21 |
| ITGB2 | 4.54070996 | 9.97912867 | 1.13599596 | 7.76E-09 | 2.15E-08 |
| IGFBPL1 | 0.3028486 | 1.00400473 | 1.72909744 | 1.09E-07 | 2.59E-07 |
| BUB1B | 0.92054602 | 2.62949324 | 1.51422304 | 4.38E-21 | 8.68E-20 |
| KCTD17 | 2.63720341 | 8.53126051 | 1.69375006 | 3.12E-26 | 2.24E-24 |
| SLC44A2 | 5.28497745 | 12.3535255 | 1.2249536 | 7.01E-09 | 1.95E-08 |
| MFSD2A | 15.2916551 | 3.35684214 | -2.187568 | 1.07E-12 | 4.96E-12 |
| COL3A1 | 36.9747089 | 80.3082678 | 1.11900973 | 8.73E-05 | 0.00014704 |
| FOLR1 | 0.31566775 | 2.35005932 | 2.89621838 | 7.93E-13 | 3.73E-12 |
| HKDC1 | 6.65370238 | 16.3576805 | 1.29773895 | 3.04E-11 | 1.13E-10 |
| TMED3 | 1.01377185 | 3.37840724 | 1.73661024 | 9.63E-21 | 1.76E-19 |
| HELLS | 0.56252091 | 1.23125539 | 1.13015141 | 2.31E-17 | 2.22E-16 |
| MCM7 | 14.3638955 | 29.7005923 | 1.04804464 | 2.74E-19 | 3.88E-18 |
| CDCA7 | 0.44701288 | 2.42534337 | 2.4398007 | 4.89E-23 | 1.51E-21 |
| BICC1 | 1.66429404 | 7.24585395 | 2.12224538 | 2.56E-11 | 9.64E-11 |
| TPPP2 | 0.7212599 | 0.21776128 | -1.7277717 | 6.10E-16 | 4.62E-15 |
| TRIM71 | 0.57390556 | 1.68732093 | 1.55584914 | 1.18E-10 | 4.05E-10 |
| COL1A1 | 35.4485565 | 74.5989245 | 1.07342795 | 2.89E-07 | 6.53E-07 |
| SCGB2A1 | 0.3419458 | 1.07407211 | 1.6512513 | 6.50E-10 | 2.06E-09 |
| AC120042.1 | 0.81889355 | 0.32524856 | -1.3321333 | 4.38E-16 | 3.40E-15 |
| TREM2 | 3.1581788 | 8.80681199 | 1.47952702 | 8.04E-14 | 4.38E-13 |
| FCN2 | 2.65038429 | 1.0646826 | -1.3157782 | 0.00019153 | 0.00030942 |
| ADAM8 | 0.8273808 | 2.21785751 | 1.4225433 | 3.42E-19 | 4.73E-18 |
| ARL4C | 3.43455176 | 9.99381864 | 1.54091421 | 1.76E-18 | 2.12E-17 |
| POPDC3 | 0.27482692 | 1.15815088 | 2.075228 | 9.33E-06 | 1.77E-05 |
| ADAMTS12 | 0.32372593 | 0.79433638 | 1.29497716 | 0.00030318 | 0.0004777 |
| AGAP2-AS1 | 0.77409472 | 2.12962137 | 1.46001494 | 1.10E-08 | 2.98E-08 |
| LINC02754 | 1.92962647 | 0.68711766 | -1.4896925 | 4.41E-14 | 2.51E-13 |
| RNF145 | 3.71223664 | 7.53354962 | 1.02104111 | 2.84E-14 | 1.66E-13 |
| MGP | 11.7441357 | 24.7481314 | 1.07537905 | 9.37E-05 | 0.00015727 |
| ESRP1 | 0.41417344 | 3.99860703 | 3.27119056 | 2.64E-12 | 1.15E-11 |
| IGKV1D-13 | 0.37700327 | 2.46234554 | 2.70738428 | 0.00107214 | 0.00158793 |
| IGLV5-45 | 0.95078759 | 4.22392529 | 2.15138933 | 0.00235064 | 0.0033411 |
| C1QTNF1 | 4.11814346 | 10.619384 | 1.36663409 | 0.00064713 | 0.00098251 |
| HSPA12A | 0.33615661 | 0.77697502 | 1.20873471 | 6.23E-13 | 2.98E-12 |
| DCLRE1C | 0.46194288 | 0.96387935 | 1.0611381 | 5.25E-23 | 1.61E-21 |
| COLEC12 | 0.43705072 | 1.14221241 | 1.38595834 | 3.10E-09 | 9.02E-09 |
| JCHAIN | 6.73949932 | 18.6901875 | 1.47156772 | 0.00538225 | 0.00732937 |
| LMNB2 | 3.07407673 | 9.14807839 | 1.57331555 | 1.47E-35 | 2.60E-32 |
| HLA-DMB | 3.21607732 | 6.75967816 | 1.07165247 | 2.98E-07 | 6.73E-07 |
| AMDHD1 | 30.6986382 | 12.9399828 | -1.246339 | 4.19E-16 | 3.25E-15 |
| MXRA8 | 2.82445511 | 7.57432228 | 1.42314424 | 5.14E-07 | 1.13E-06 |
| E2F2 | 0.35949064 | 0.84635451 | 1.23530789 | 4.30E-18 | 4.74E-17 |
| YJEFN3 | 0.45583953 | 1.07716345 | 1.24063924 | 1.76E-16 | 1.45E-15 |
| DGKA | 0.44309469 | 0.90132715 | 1.02443579 | 1.29E-09 | 3.95E-09 |
| PHLDA2 | 4.95516474 | 13.6256694 | 1.45932218 | 8.75E-15 | 5.50E-14 |
| FUT2 | 0.49765295 | 1.44629187 | 1.53914682 | 3.42E-13 | 1.69E-12 |
| KIRREL2 | 0.02936021 | 3.56286152 | 6.92303018 | 5.21E-07 | 1.14E-06 |
| S100A4 | 12.3621786 | 34.7956253 | 1.49297292 | 3.47E-17 | 3.24E-16 |
| RAD51 | 0.73182259 | 1.86849107 | 1.35230782 | 7.91E-25 | 3.76E-23 |
| SYT13 | 0.46190344 | 4.09310992 | 3.1475342 | 1.10E-10 | 3.81E-10 |
| AL138847.2 | 4.75942391 | 2.33143202 | -1.0295706 | 2.67E-17 | 2.54E-16 |
| FNDC1 | 0.58000136 | 1.17987866 | 1.02451032 | 8.40E-05 | 0.00014192 |
| CYBA | 10.5831973 | 34.0731646 | 1.6868604 | 3.15E-19 | 4.38E-18 |
| MROH2A | 1.74790391 | 0.51840333 | -1.753479 | 2.11E-06 | 4.30E-06 |
| PSTPIP1 | 0.46793906 | 1.12683935 | 1.26788928 | 2.73E-11 | 1.02E-10 |
| MT1DP | 16.7860829 | 3.6578944 | -2.1981803 | 0.01830232 | 0.02331958 |
| CNFN | 1.14170806 | 2.59487707 | 1.1844724 | 4.17E-11 | 1.53E-10 |
| PAQR5 | 1.41138757 | 3.48936023 | 1.30584833 | 7.54E-10 | 2.37E-09 |
| KRBA1 | 0.55416438 | 1.40833245 | 1.34560206 | 1.96E-21 | 4.21E-20 |
| B4GALNT4 | 0.17776907 | 2.4777863 | 3.80097554 | 6.19E-14 | 3.44E-13 |
| MZB1 | 1.22023933 | 4.91748806 | 2.01075741 | 3.70E-08 | 9.33E-08 |
| TAT-AS1 | 1.16126733 | 0.41523298 | -1.4837072 | 8.91E-11 | 3.13E-10 |
| TAP1 | 11.9241751 | 23.9431843 | 1.00572557 | 4.11E-08 | 1.03E-07 |
| IGHV4-55 | 0.34676773 | 1.21619625 | 1.8103345 | 0.00569351 | 0.00773083 |
| CCL16 | 62.4618193 | 29.0701406 | -1.1034365 | 1.85E-15 | 1.29E-14 |
| AC016394.1 | 0.5554596 | 1.21196688 | 1.12559638 | 2.56E-18 | 2.98E-17 |
| IGLV2-11 | 9.59468319 | 25.4467511 | 1.40717439 | 0.00800297 | 0.01065626 |
| TTK | 0.76303313 | 2.03790941 | 1.41727231 | 2.12E-19 | 3.05E-18 |
| NCEH1 | 1.3048419 | 5.17845549 | 1.98864685 | 1.66E-09 | 5.04E-09 |
| ARMCX2 | 0.59543388 | 1.72660976 | 1.53592884 | 3.56E-06 | 7.09E-06 |
| WNK2 | 0.23293794 | 3.02586493 | 3.69933006 | 2.51E-23 | 8.05E-22 |
| FCHSD1 | 0.49707607 | 1.12469141 | 1.17799068 | 3.56E-14 | 2.05E-13 |
| LZTS2 | 3.52475916 | 7.86411495 | 1.15775972 | 1.05E-29 | 2.64E-27 |
| OLFM4 | 0.59238692 | 5.91715377 | 3.3202917 | 3.93E-09 | 1.13E-08 |
| ZNF682 | 0.43214213 | 0.90418808 | 1.06511702 | 1.34E-08 | 3.58E-08 |
| S1PR4 | 0.67336357 | 1.37228295 | 1.02712039 | 0.00029965 | 0.00047277 |
| PRKX | 0.75021275 | 2.01984125 | 1.42887023 | 1.03E-11 | 4.11E-11 |
| LEFTY1 | 0.40797662 | 1.78158442 | 2.12660248 | 2.75E-09 | 8.06E-09 |
| ZNF496 | 1.13081737 | 2.50622524 | 1.14815013 | 2.08E-17 | 2.02E-16 |
| PRKCD | 4.37965499 | 9.12708478 | 1.05933691 | 1.47E-24 | 6.47E-23 |
| SNHG12 | 1.14928599 | 2.68575644 | 1.22459064 | 2.89E-28 | 3.92E-26 |
| TRDC | 0.72691881 | 2.72275739 | 1.90520229 | 0.01009403 | 0.01326765 |
| CXCL6 | 2.49591732 | 7.6464343 | 1.615217 | 0.00240769 | 0.00341874 |
| OLFML3 | 2.61152749 | 7.26166338 | 1.47540616 | 3.32E-12 | 1.43E-11 |
| ADHFE1 | 10.0722915 | 4.77193926 | -1.0777444 | 5.71E-22 | 1.39E-20 |
| EFHD1 | 7.97435894 | 3.91343115 | -1.0269345 | 0.01088832 | 0.01425716 |
| C15orf39 | 2.50172081 | 5.97905281 | 1.25699616 | 2.02E-32 | 1.29E-29 |
| TSPAN15 | 3.89131713 | 13.2888041 | 1.77188081 | 8.61E-15 | 5.42E-14 |
| SORD | 38.0720282 | 18.3543151 | -1.0526121 | 2.29E-16 | 1.86E-15 |
| AC004862.1 | 2.3041637 | 0.82480917 | -1.4821109 | 4.97E-12 | 2.08E-11 |
| IGKV3-15 | 7.08204936 | 22.1998888 | 1.64831365 | 0.00040177 | 0.0006252 |
| CENPE | 0.43292899 | 0.95218912 | 1.13711774 | 8.08E-16 | 6.00E-15 |
| NEBL | 0.31735804 | 1.00874131 | 1.66837294 | 2.17E-05 | 3.94E-05 |
| MMP9 | 4.4034049 | 16.3108322 | 1.88913897 | 1.87E-18 | 2.24E-17 |
| KRTCAP3 | 3.12385973 | 8.40256054 | 1.42749936 | 1.84E-06 | 3.78E-06 |
| KIF4A | 1.65822251 | 4.49795532 | 1.43963172 | 2.47E-20 | 4.18E-19 |
| SIGLEC9 | 0.46497089 | 0.98438288 | 1.08207916 | 9.98E-09 | 2.73E-08 |
| AC087379.2 | 2.24011061 | 0.52387932 | -2.0962636 | 0.00140764 | 0.00205632 |
| GIT1 | 4.02374978 | 8.3847309 | 1.05922389 | 2.71E-35 | 3.82E-32 |
| SEMA4D | 0.35186945 | 0.93241302 | 1.40592889 | 1.81E-17 | 1.78E-16 |
| AL162413.1 | 2.59346105 | 6.61163805 | 1.35012904 | 0.01012785 | 0.01330838 |
| FGFR3 | 10.2476588 | 28.174532 | 1.4590973 | 1.25E-14 | 7.71E-14 |
| CYP2A7 | 39.5134417 | 5.20067608 | -2.9255724 | 2.25E-13 | 1.15E-12 |
| RTL8B | 1.03530747 | 2.61897096 | 1.33894077 | 1.79E-09 | 5.40E-09 |
| CKAP4 | 23.7960475 | 50.4573929 | 1.0843437 | 2.90E-25 | 1.56E-23 |
| LINC01093 | 6.51456667 | 1.15719291 | -2.4930398 | 1.79E-11 | 6.86E-11 |
| MTFR2 | 0.37948418 | 1.21666054 | 1.68081505 | 6.35E-29 | 1.07E-26 |
| CCR5 | 0.67451523 | 1.43203633 | 1.08614515 | 2.60E-06 | 5.26E-06 |
| SNHG3 | 1.77855664 | 5.46014226 | 1.61823162 | 4.33E-27 | 4.07E-25 |
| AVPR1A | 4.77848957 | 2.16056356 | -1.145147 | 6.14E-05 | 0.00010537 |
| AQP7P1 | 2.11317255 | 0.95997779 | -1.1383376 | 1.16E-06 | 2.44E-06 |
| BTK | 0.4683416 | 0.9720303 | 1.05344009 | 6.16E-08 | 1.51E-07 |
| CDK16 | 5.50652652 | 11.6713222 | 1.08375354 | 1.69E-34 | 1.61E-31 |
| MILR1 | 0.6311498 | 1.27620608 | 1.01580696 | 1.23E-09 | 3.78E-09 |
| GSTP1 | 9.46347339 | 41.9967626 | 2.14983642 | 1.74E-12 | 7.81E-12 |
| HAUS7 | 0.42657977 | 1.01547631 | 1.25126911 | 7.85E-20 | 1.22E-18 |
| A1BG | 28.9858275 | 12.4003515 | -1.2249667 | 1.97E-19 | 2.84E-18 |
| SLC25A47 | 76.9922834 | 27.0315779 | -1.5100681 | 3.72E-09 | 1.07E-08 |
| VSIG1 | 0.48375112 | 2.05255504 | 2.085084 | 3.58E-08 | 9.04E-08 |
| AC055822.1 | 0.48184414 | 1.02001928 | 1.08195798 | 1.93E-16 | 1.58E-15 |
| TFPI2 | 0.58055845 | 1.58307627 | 1.44721754 | 0.00505557 | 0.00690117 |
| AP001783.1 | 12.466096 | 3.48635414 | -1.8382187 | 7.61E-12 | 3.10E-11 |
| GNAZ | 2.30545283 | 4.78832863 | 1.05447202 | 1.14E-14 | 7.06E-14 |
| AC138207.3 | 0.34114903 | 1.3525816 | 1.98724163 | 4.48E-17 | 4.09E-16 |
| AC135584.1 | 1.15305718 | 0.48557043 | -1.2477116 | 0.00280792 | 0.00395122 |
| GINS4 | 0.42501801 | 0.86214833 | 1.02041212 | 2.86E-11 | 1.07E-10 |
| SLC16A3 | 1.50738298 | 5.33012467 | 1.82212327 | 2.18E-22 | 5.82E-21 |
| SPINT2 | 2.88003569 | 17.5510662 | 2.60740008 | 1.65E-10 | 5.56E-10 |
| LINC00992 | 0.24182378 | 0.97277321 | 2.00814735 | 3.75E-13 | 1.85E-12 |
| AL161785.1 | 0.51323223 | 1.13164903 | 1.14074291 | 1.37E-08 | 3.67E-08 |
| RASSF3 | 5.26431659 | 10.5564008 | 1.00379987 | 2.17E-17 | 2.10E-16 |
| CD86 | 1.00795195 | 2.12575375 | 1.07654761 | 1.68E-09 | 5.10E-09 |
| DLG3 | 0.49218887 | 1.91221621 | 1.95796172 | 2.85E-18 | 3.28E-17 |
| PLEKHG2 | 1.16791665 | 2.512138 | 1.10497839 | 2.39E-22 | 6.29E-21 |
| AC010332.1 | 0.33422662 | 0.88331357 | 1.40209903 | 3.45E-07 | 7.72E-07 |
| FAM118A | 1.51861001 | 3.30312701 | 1.12108103 | 6.08E-21 | 1.16E-19 |
| AC022150.2 | 0.37077331 | 0.80597473 | 1.12019722 | 1.16E-08 | 3.13E-08 |
| ALDOA | 43.1811273 | 114.365866 | 1.40518372 | 3.15E-21 | 6.44E-20 |
| AC087164.2 | 0.79551951 | 0.32681568 | -1.2834201 | 9.00E-08 | 2.17E-07 |
| LOXL1 | 1.05786459 | 4.3048266 | 2.02480016 | 3.34E-06 | 6.68E-06 |
| OGFRL1 | 0.83569149 | 1.76718882 | 1.08041385 | 2.23E-14 | 1.32E-13 |
| ASNS | 1.3160699 | 4.31198018 | 1.71211443 | 3.44E-18 | 3.88E-17 |
| CSF1 | 4.40739988 | 9.156122 | 1.05480889 | 4.21E-09 | 1.20E-08 |
| SPHK1 | 1.54830011 | 10.7089994 | 2.79006663 | 2.51E-23 | 8.05E-22 |
| CHN1 | 0.44129188 | 1.21968594 | 1.46670461 | 0.00073382 | 0.00110697 |
| CDC45 | 1.49153087 | 3.64272727 | 1.28822515 | 1.69E-20 | 2.94E-19 |
| DSG1 | 2.33701693 | 0.73043537 | -1.6778396 | 7.81E-17 | 6.84E-16 |
| GLYATL1P4 | 1.01053896 | 0.19951768 | -2.3405365 | 1.57E-05 | 2.91E-05 |
| PTTG1 | 5.1791526 | 14.659443 | 1.50104231 | 3.84E-23 | 1.20E-21 |
| MMP14 | 12.5350446 | 37.3570093 | 1.57541183 | 7.26E-10 | 2.29E-09 |
| AC010326.4 | 1.79579277 | 3.6380478 | 1.01854362 | 1.42E-23 | 4.77E-22 |
| BX842568.3 | 1.44165027 | 0.70328715 | -1.0355355 | 1.18E-09 | 3.63E-09 |
| MUC6 | 1.89162397 | 7.21288558 | 1.93095121 | 7.67E-05 | 0.00013028 |
| ADORA1 | 0.42293376 | 1.61652553 | 1.93439265 | 1.92E-07 | 4.45E-07 |
| SFRP5 | 0.82861351 | 17.5861775 | 4.40759879 | 3.52E-08 | 8.91E-08 |
| TRARG1 | 0.01036692 | 1.642337 | 7.30761851 | 0.00252546 | 0.00357444 |
| ZNF816 | 0.465913 | 1.10483167 | 1.24569408 | 8.67E-16 | 6.38E-15 |
| IFI27 | 95.322122 | 47.3820017 | -1.0084719 | 0.00159467 | 0.00231467 |
| AZGP1P2 | 0.94724413 | 0.26611707 | -1.8316753 | 1.84E-19 | 2.67E-18 |
| CPVL | 4.72579826 | 12.3030787 | 1.38038943 | 2.58E-10 | 8.51E-10 |
| ARHGAP11A | 0.98771996 | 2.10725381 | 1.09319013 | 2.53E-16 | 2.04E-15 |
| CARD11 | 0.48773834 | 1.51022925 | 1.63058828 | 1.29E-09 | 3.95E-09 |
| AKR1D1 | 26.3895224 | 10.5162903 | -1.3273394 | 2.93E-08 | 7.49E-08 |
| INHA | 0.34749214 | 3.19081613 | 3.19887324 | 1.37E-09 | 4.18E-09 |
| CA12 | 2.63197205 | 5.28799234 | 1.00657592 | 8.70E-07 | 1.86E-06 |
| POLD1 | 3.28867524 | 6.73428577 | 1.0340184 | 7.74E-27 | 6.87E-25 |
| RUSC1 | 3.0666969 | 6.24734069 | 1.02655662 | 7.11E-24 | 2.65E-22 |
| IGSF3 | 1.39081987 | 3.90622617 | 1.4898399 | 5.33E-11 | 1.92E-10 |
| HROB | 0.72560887 | 1.73551449 | 1.25809941 | 1.08E-23 | 3.82E-22 |
| VPREB3 | 0.84371382 | 1.72670872 | 1.03319909 | 0.01906575 | 0.02422005 |
| SCT | 0.35386313 | 1.04530405 | 1.5626593 | 0.00166403 | 0.00241037 |
| PPP1R14C | 0.05957672 | 1.24170913 | 4.38143476 | 2.28E-12 | 1.00E-11 |
| FYTTD1P1 | 1.31715602 | 0.56076046 | -1.2319697 | 2.11E-07 | 4.85E-07 |
| HMGA1 | 23.1539391 | 62.2361854 | 1.42649598 | 1.02E-25 | 6.36E-24 |
| UGT2B10 | 131.326407 | 36.9814013 | -1.8282852 | 3.09E-26 | 2.23E-24 |
| IGHA1 | 56.8220209 | 151.510254 | 1.41489339 | 0.00136745 | 0.00199988 |
| CDCA4 | 1.80907328 | 4.32289542 | 1.25674709 | 1.10E-28 | 1.73E-26 |
| AL627309.6 | 0.70595255 | 1.53737096 | 1.12282221 | 2.72E-09 | 7.98E-09 |
| GAL3ST4 | 0.51116367 | 1.40425535 | 1.45794808 | 1.05E-10 | 3.63E-10 |
| IGLC2 | 36.3835098 | 167.241995 | 2.20058053 | 1.76E-05 | 3.23E-05 |
| LRRC17 | 0.58449129 | 1.45680584 | 1.31755518 | 0.00261206 | 0.00368923 |
| UPP2-IT1 | 0.78397345 | 0.23354761 | -1.7470881 | 6.86E-05 | 0.00011717 |
| SLC6A8 | 3.08040594 | 10.5248838 | 1.77261192 | 8.85E-16 | 6.51E-15 |
| PKP3 | 0.24323319 | 2.73687431 | 3.49211718 | 9.06E-13 | 4.24E-12 |
| SLCO1B1 | 67.0368659 | 31.4048135 | -1.093969 | 2.02E-17 | 1.97E-16 |
| KCNIP3 | 0.33380982 | 0.75525044 | 1.17792874 | 4.11E-07 | 9.11E-07 |
| JAK3 | 0.75346836 | 1.91180206 | 1.34331433 | 5.02E-13 | 2.43E-12 |
| PCLAF | 1.69130912 | 4.15450546 | 1.29653639 | 1.14E-20 | 2.06E-19 |
| ADAMTS9 | 0.88008626 | 1.82649071 | 1.05335758 | 5.22E-07 | 1.14E-06 |
| CPA2 | 0.065969 | 9.04359249 | 7.09896404 | 2.41E-10 | 7.98E-10 |
| SDS | 228.320017 | 71.7541312 | -1.6699235 | 1.90E-08 | 5.00E-08 |
| SNX20 | 0.36037307 | 0.79798755 | 1.14687502 | 1.44E-08 | 3.85E-08 |
| RP9P | 1.08732289 | 2.60051833 | 1.25801878 | 6.99E-30 | 1.93E-27 |
| RAI1 | 1.20514834 | 2.41821295 | 1.00473056 | 6.83E-12 | 2.80E-11 |
| MLLT3 | 0.30095902 | 0.93423322 | 1.6342157 | 3.13E-09 | 9.12E-09 |
| IYD | 2.37649752 | 1.1127911 | -1.0946541 | 1.47E-13 | 7.69E-13 |
| STMN1 | 9.41201976 | 19.1954222 | 1.02818604 | 2.31E-16 | 1.87E-15 |
| SNHG15 | 1.26949686 | 2.69380686 | 1.08538959 | 1.73E-12 | 7.76E-12 |
| ORAI2 | 0.46348577 | 1.52920563 | 1.72218546 | 3.19E-20 | 5.29E-19 |
| C9 | 120.463452 | 45.5115941 | -1.4042895 | 5.07E-05 | 8.79E-05 |
| MX2 | 0.47473759 | 1.10318546 | 1.21647315 | 7.93E-12 | 3.22E-11 |
| ZBTB12 | 1.17834674 | 2.99712507 | 1.34681516 | 3.44E-18 | 3.88E-17 |
| EDA | 0.54932456 | 1.14562212 | 1.06040054 | 1.79E-07 | 4.17E-07 |
| MIR8071-2 | 0.51596113 | 1.83915872 | 1.83371169 | 0.00045949 | 0.00070985 |
| CCL26 | 0.28004135 | 1.08391632 | 1.9525416 | 5.15E-13 | 2.49E-12 |
| PRC1 | 2.59474386 | 5.45869618 | 1.07296427 | 2.78E-18 | 3.20E-17 |
| LINC00342 | 0.74443726 | 1.68442285 | 1.17803217 | 1.03E-15 | 7.49E-15 |
| IGKV2D-40 | 0.74356524 | 4.65754676 | 2.64703903 | 0.03465291 | 0.04249323 |
| AC011462.4 | 0.32225069 | 0.81188974 | 1.33310036 | 5.21E-19 | 6.96E-18 |
| FHL3 | 4.41535667 | 9.04998657 | 1.03538567 | 2.00E-21 | 4.27E-20 |
| SUSD1 | 0.89922281 | 1.81606878 | 1.0140683 | 6.46E-18 | 6.82E-17 |
| CEACAM21 | 0.36396214 | 0.76267727 | 1.06728433 | 3.86E-07 | 8.57E-07 |
| ANO1 | 13.4721876 | 6.30289255 | -1.0958982 | 3.02E-09 | 8.81E-09 |
| LGI4 | 0.82203143 | 1.81462724 | 1.14240776 | 0.00267368 | 0.00377059 |
| PKHD1 | 0.27819515 | 1.14019658 | 2.03511338 | 0.01277203 | 0.01657437 |
| CTH | 41.6535433 | 12.6745046 | -1.7165099 | 1.34E-21 | 2.98E-20 |
| AP003174.1 | 0.69128166 | 0.34520364 | -1.001826 | 7.74E-07 | 1.66E-06 |
| LHFPL3-AS2 | 0.11009848 | 1.16445456 | 3.40278783 | 3.01E-21 | 6.18E-20 |
| HSD11B1 | 274.548857 | 77.5375148 | -1.8240965 | 1.38E-16 | 1.16E-15 |
| JAML | 0.99671132 | 2.23212296 | 1.16316889 | 6.09E-14 | 3.39E-13 |
| CYP2B6 | 41.3337745 | 11.0287957 | -1.9060459 | 7.52E-15 | 4.79E-14 |
| IGFBP6 | 1.72433086 | 4.9592261 | 1.52407838 | 6.49E-11 | 2.30E-10 |
| IGHV4-39 | 11.3160271 | 40.2084337 | 1.82913061 | 0.00112551 | 0.00166296 |
| IGLL5 | 2.95407243 | 9.2858107 | 1.65232267 | 0.00023094 | 0.00037011 |
| SLC52A2 | 7.00801513 | 15.9693774 | 1.18823028 | 7.19E-24 | 2.66E-22 |
| SOAT2 | 3.37385238 | 8.00139364 | 1.24585445 | 0.00416671 | 0.00573418 |
| KCNJ8 | 20.0948193 | 9.68604372 | -1.0528442 | 5.37E-15 | 3.49E-14 |
| AC011477.1 | 0.64113034 | 1.48766817 | 1.21436318 | 1.57E-14 | 9.54E-14 |
| CCDC102A | 1.07756754 | 2.26055794 | 1.0689006 | 1.28E-12 | 5.88E-12 |
| PTPRE | 0.4704042 | 1.13091385 | 1.26551618 | 1.20E-08 | 3.24E-08 |
| GINS1 | 1.49272363 | 3.86344942 | 1.37194243 | 3.77E-22 | 9.52E-21 |
| FBL | 35.4791934 | 75.5460486 | 1.09038309 | 1.46E-21 | 3.22E-20 |
| IGHV1-46 | 2.05160592 | 10.2853642 | 2.32576733 | 0.0015493 | 0.00225299 |
| C19orf33 | 1.6478052 | 6.05672402 | 1.87799198 | 1.27E-12 | 5.81E-12 |
| HCST | 4.27806518 | 9.21760931 | 1.10743416 | 7.02E-08 | 1.71E-07 |
| CYP26B1 | 0.26042997 | 1.33407525 | 2.35687267 | 2.94E-08 | 7.53E-08 |
| PFN2 | 2.75556531 | 7.65284175 | 1.47364725 | 6.93E-07 | 1.49E-06 |
| AATK | 0.39436013 | 0.84564598 | 1.10054012 | 2.38E-07 | 5.43E-07 |
| CENPL | 0.70371056 | 1.40783241 | 1.00042153 | 6.49E-19 | 8.46E-18 |
| TESC | 7.85751902 | 27.975161 | 1.83200067 | 9.55E-15 | 5.97E-14 |
| SSUH2 | 0.64497892 | 1.3252233 | 1.03891157 | 5.39E-07 | 1.18E-06 |
| RAB25 | 0.52501829 | 10.0907055 | 4.26451556 | 3.29E-09 | 9.55E-09 |
| PCK2 | 119.10268 | 50.1014956 | -1.2492803 | 1.41E-25 | 8.51E-24 |
| BEX4 | 3.77225934 | 9.52345186 | 1.33605572 | 5.37E-15 | 3.49E-14 |
| TUBB2B | 0.9044066 | 2.10625868 | 1.2196392 | 3.79E-05 | 6.68E-05 |
| SOX4 | 3.39820496 | 12.8899299 | 1.92339964 | 1.35E-21 | 3.00E-20 |
| DLGAP5 | 1.12197637 | 2.96604753 | 1.40249943 | 7.16E-21 | 1.35E-19 |
| AC099560.2 | 0.95868445 | 1.9578347 | 1.03013102 | 7.63E-05 | 0.00012964 |
| BX322562.1 | 1.30874126 | 3.04105353 | 1.21639131 | 2.71E-07 | 6.15E-07 |
| JPT1 | 9.41293249 | 22.8316523 | 1.27831912 | 1.16E-27 | 1.29E-25 |
| AC008592.3 | 1.24950601 | 0.53360413 | -1.2275161 | 7.77E-07 | 1.67E-06 |
| SYNGR1 | 1.48308133 | 3.12509966 | 1.07530448 | 2.77E-09 | 8.12E-09 |
| IGKV3-7 | 0.23051857 | 0.92817559 | 2.00951477 | 0.00294801 | 0.00413472 |
| CKS2 | 19.6439354 | 46.3141291 | 1.2373684 | 3.92E-23 | 1.22E-21 |
| KRT19 | 2.33686029 | 32.1368898 | 3.78158692 | 1.25E-18 | 1.55E-17 |
| FYB1 | 1.01374208 | 2.08234561 | 1.0385189 | 0.00044933 | 0.000695 |
| PLAC8 | 0.34938407 | 1.05962604 | 1.60066946 | 8.89E-07 | 1.89E-06 |
| H19 | 113.437645 | 413.500739 | 1.86599043 | 5.03E-06 | 9.85E-06 |
| THRSP | 58.8424252 | 22.5666346 | -1.3826654 | 1.19E-09 | 3.67E-09 |
| CD248 | 3.82185348 | 7.9192533 | 1.05109194 | 4.57E-10 | 1.47E-09 |
| OTC | 75.7929612 | 33.9329336 | -1.1593777 | 2.95E-19 | 4.15E-18 |
| RNASE2 | 0.48231996 | 1.2415952 | 1.36413245 | 8.79E-15 | 5.53E-14 |
| TNFSF9 | 0.30453688 | 0.83244929 | 1.45074543 | 1.08E-12 | 4.99E-12 |
| EZH2 | 2.02624694 | 4.17185729 | 1.0418798 | 1.40E-19 | 2.06E-18 |
| ZNF883 | 0.33867485 | 0.76314556 | 1.17205741 | 5.84E-09 | 1.64E-08 |
| ABHD1 | 0.94635142 | 0.42833289 | -1.1436436 | 2.08E-05 | 3.78E-05 |
| B3GALNT1 | 0.4215344 | 1.2199994 | 1.53315816 | 4.42E-17 | 4.03E-16 |
| CPED1 | 1.9915253 | 0.91021994 | -1.1295867 | 4.47E-14 | 2.54E-13 |
| UGT1A3 | 3.00553985 | 0.83579597 | -1.8464014 | 1.44E-15 | 1.02E-14 |
| ARHGEF3 | 1.42540395 | 3.08675913 | 1.11472208 | 8.69E-12 | 3.51E-11 |
| UNC119 | 2.82980912 | 5.82595525 | 1.04178988 | 3.87E-26 | 2.70E-24 |
| DEPDC1 | 0.69593475 | 1.40630231 | 1.01488281 | 5.52E-12 | 2.30E-11 |
| NUMBL | 0.83542317 | 1.817351 | 1.12125802 | 7.50E-16 | 5.60E-15 |
| PKN1 | 17.6936901 | 36.7138009 | 1.05308752 | 3.29E-26 | 2.36E-24 |
| LPCAT4 | 0.92139658 | 2.76576723 | 1.58578559 | 8.84E-21 | 1.63E-19 |
| TMEM252 | 1.00008751 | 0.15276366 | -2.7107529 | 2.48E-06 | 5.04E-06 |
| ADH1B | 303.145241 | 103.789139 | -1.5463537 | 1.88E-19 | 2.72E-18 |
| APOC1P1 | 79.815519 | 34.0391635 | -1.2294737 | 1.43E-06 | 2.97E-06 |
| AL021707.6 | 0.8464335 | 1.71729988 | 1.02067336 | 1.58E-19 | 2.31E-18 |
| HSPA7 | 1.20744997 | 2.74396091 | 1.18429652 | 3.42E-09 | 9.88E-09 |
| HORMAD2-AS1 | 6.3960846 | 2.37656204 | -1.428313 | 3.89E-08 | 9.77E-08 |
| CD24 | 22.6918841 | 83.8414256 | 1.88548685 | 1.15E-19 | 1.74E-18 |
| GRB7 | 2.73888497 | 7.28263091 | 1.41087106 | 7.84E-13 | 3.69E-12 |
| RNASET2 | 3.36654073 | 7.02541026 | 1.06131555 | 2.81E-21 | 5.80E-20 |
| LINC00189 | 0.42693133 | 0.91805812 | 1.10458144 | 0.0040261 | 0.00555288 |
| IGKV1-27 | 1.88769843 | 10.102413 | 2.41999971 | 0.00327897 | 0.00457027 |
| AC113410.3 | 0.38361763 | 0.95833119 | 1.32085529 | 6.57E-13 | 3.13E-12 |
| HLA-DPB2 | 0.31530572 | 0.93465352 | 1.5676803 | 1.95E-10 | 6.54E-10 |
| PLXNA1 | 1.30025021 | 3.33085267 | 1.35710228 | 1.98E-23 | 6.50E-22 |
| AC004687.1 | 0.36540915 | 1.01398004 | 1.4724446 | 6.23E-12 | 2.57E-11 |
| YBX3 | 2.58201489 | 5.83485534 | 1.17619957 | 4.63E-10 | 1.49E-09 |
| ARID3C | 2.85902921 | 1.36697738 | -1.064536 | 6.23E-12 | 2.57E-11 |
| IGF2BP3 | 0.30063044 | 0.85504689 | 1.50801244 | 1.81E-08 | 4.77E-08 |
| IGHM | 13.8155557 | 47.8887503 | 1.79339319 | 0.00056262 | 0.00085891 |
| PSRC1 | 1.29037061 | 2.98036913 | 1.20770554 | 5.61E-20 | 8.93E-19 |
| SINHCAF | 1.30706096 | 3.76963529 | 1.52809852 | 1.54E-14 | 9.33E-14 |
| SRC | 3.56786827 | 8.0303841 | 1.17040664 | 1.61E-20 | 2.83E-19 |
| AP1M2 | 5.78200405 | 12.4439272 | 1.10580034 | 6.69E-07 | 1.45E-06 |
| FBP1 | 182.05409 | 79.4080809 | -1.1970094 | 3.28E-13 | 1.63E-12 |
| MARCHF3 | 0.32530124 | 0.92943843 | 1.51458297 | 1.20E-21 | 2.73E-20 |
| GNAO1 | 1.07836877 | 0.49458329 | -1.1245652 | 5.06E-05 | 8.77E-05 |
| SEMA6A | 0.86045624 | 3.19780443 | 1.89390798 | 4.36E-07 | 9.64E-07 |
| TMIGD3 | 0.41250548 | 1.00419694 | 1.28355705 | 9.24E-09 | 2.53E-08 |
| ITGB4 | 1.42162986 | 6.89196975 | 2.27737048 | 1.84E-14 | 1.11E-13 |
| C8B | 135.617209 | 63.2847636 | -1.0996101 | 2.53E-25 | 1.37E-23 |
| EMID1 | 1.99102605 | 4.19798844 | 1.07618609 | 0.00011122 | 0.00018487 |
| CD7 | 1.50876901 | 8.58702272 | 2.50878606 | 5.54E-13 | 2.67E-12 |
| NIBAN2 | 8.43411542 | 21.2215314 | 1.3312201 | 2.84E-14 | 1.66E-13 |
| RTP3 | 29.3911158 | 13.2557564 | -1.1487611 | 3.56E-17 | 3.32E-16 |
| HAVCR2 | 1.16706444 | 2.91108597 | 1.31867323 | 2.10E-10 | 7.01E-10 |
| LINC01836 | 0.49108962 | 1.46805086 | 1.57984373 | 4.61E-09 | 1.31E-08 |
| ARHGEF19 | 0.69541662 | 1.41338718 | 1.02320727 | 1.70E-10 | 5.74E-10 |
| UGT1A2P | 4.32216972 | 0.78061968 | -2.469064 | 1.55E-14 | 9.41E-14 |
| CTXN1 | 0.25140539 | 1.82233998 | 2.85770465 | 2.74E-15 | 1.87E-14 |
| HAO2 | 32.2348149 | 16.0228407 | -1.0084898 | 1.95E-10 | 6.54E-10 |
| IGHV3-53 | 1.33381794 | 5.79999919 | 2.12049094 | 0.00559212 | 0.00760194 |
| PTGDR2 | 0.6573338 | 1.85720322 | 1.49843362 | 0.00126988 | 0.00186435 |
| ALOX5 | 1.06943027 | 3.9225277 | 1.87494122 | 3.36E-13 | 1.66E-12 |
| INSIG1 | 188.30624 | 75.8887497 | -1.3111229 | 2.18E-19 | 3.14E-18 |
| DAO | 16.6780046 | 7.98263387 | -1.0630099 | 1.10E-15 | 7.97E-15 |
| GALNT15 | 0.78587148 | 0.36439642 | -1.1087846 | 3.50E-10 | 1.14E-09 |
| XRCC2 | 0.36666742 | 0.87488791 | 1.2546261 | 1.14E-19 | 1.73E-18 |
| IBSP | 0.15902324 | 1.5388604 | 3.27455281 | 8.49E-06 | 1.62E-05 |
| ARFGEF3 | 0.39649944 | 1.07277175 | 1.4359524 | 4.69E-12 | 1.97E-11 |
| AP3M2 | 0.91883825 | 1.85262703 | 1.01168965 | 7.00E-18 | 7.34E-17 |
| AGXT2 | 11.8291305 | 5.41679826 | -1.1268318 | 7.92E-17 | 6.92E-16 |
| PPP1R18 | 6.4418127 | 13.0990277 | 1.02392111 | 2.08E-12 | 9.19E-12 |
| C6orf223 | 0.99591483 | 2.84172896 | 1.51267469 | 6.22E-15 | 4.00E-14 |
| PMEPA1 | 1.76411553 | 9.00290557 | 2.35144565 | 2.67E-08 | 6.87E-08 |
| LINC02331 | 0.28796318 | 0.92881367 | 1.68950487 | 0.00092593 | 0.00138125 |
| RAB27B | 0.29719508 | 0.91554265 | 1.62321685 | 0.00010826 | 0.00018018 |
| WDR54 | 1.02238111 | 2.58103461 | 1.3360164 | 3.38E-22 | 8.60E-21 |
| MAD2L1 | 1.10290273 | 2.24887288 | 1.02789655 | 6.00E-16 | 4.55E-15 |
| IGHGP | 4.16894501 | 14.5567159 | 1.80393066 | 2.00E-06 | 4.08E-06 |
| CYP2A6 | 408.828713 | 93.274594 | -2.1319404 | 4.82E-18 | 5.26E-17 |
| LSP1 | 3.14449856 | 7.15752764 | 1.18663136 | 8.27E-12 | 3.35E-11 |
| DTX3 | 1.03979574 | 2.99866002 | 1.52801782 | 1.42E-15 | 1.01E-14 |
| AC068987.3 | 1.03679278 | 2.47697978 | 1.25645452 | 3.96E-09 | 1.14E-08 |
| CDK1 | 2.60736816 | 6.69564578 | 1.3606289 | 1.26E-22 | 3.59E-21 |
| CHST2 | 0.65441141 | 1.6956714 | 1.37358681 | 1.15E-09 | 3.55E-09 |
| NCDN | 2.32776325 | 4.70608195 | 1.01558211 | 1.48E-17 | 1.47E-16 |
| ZNF385A | 2.83318543 | 7.03348272 | 1.31181421 | 1.64E-20 | 2.87E-19 |
| CYTOR | 2.84967767 | 6.12383517 | 1.10363671 | 1.26E-16 | 1.06E-15 |
| SH3BGRL3 | 37.7018552 | 75.8051205 | 1.00765979 | 1.85E-18 | 2.23E-17 |
| IGHV3-48 | 1.17341344 | 3.22891541 | 1.46033823 | 0.00191909 | 0.00275549 |
| ZMYND12 | 1.41482333 | 0.64287566 | -1.1380103 | 1.58E-18 | 1.92E-17 |
| TMSB4XP8 | 4.3148623 | 9.34119527 | 1.11429264 | 1.10E-10 | 3.81E-10 |
| ARHGEF2 | 2.12011317 | 5.28526015 | 1.31783321 | 4.47E-27 | 4.18E-25 |
| FLNA | 12.805791 | 35.4265326 | 1.4680339 | 1.08E-07 | 2.58E-07 |
| SNCG | 11.7849372 | 36.0147343 | 1.61164319 | 7.28E-05 | 0.00012395 |
| UBXN10 | 3.04404725 | 1.35656173 | -1.166036 | 1.25E-10 | 4.29E-10 |
| MRC2 | 1.56482501 | 4.60981249 | 1.55870674 | 2.42E-08 | 6.26E-08 |
| SLAMF7 | 0.9439329 | 2.64113861 | 1.4844038 | 4.55E-07 | 1.00E-06 |
| MPDZ | 5.36626077 | 2.54201447 | -1.0779449 | 2.21E-14 | 1.31E-13 |
| BIRC5 | 3.74650205 | 11.7224926 | 1.64566322 | 1.95E-24 | 8.27E-23 |
| ZNF532 | 0.64958677 | 1.46069581 | 1.16906162 | 1.72E-10 | 5.80E-10 |
| UCA1 | 1.33556595 | 3.92798412 | 1.55633788 | 2.83E-10 | 9.29E-10 |
| NEK2 | 1.83064182 | 4.52950048 | 1.30700241 | 1.52E-19 | 2.24E-18 |
| SPINT1-AS1 | 0.40168713 | 2.2225059 | 2.4680431 | 2.58E-14 | 1.52E-13 |
| CTF1 | 0.40069242 | 0.85083346 | 1.08638154 | 4.97E-05 | 8.64E-05 |
| ACSM5 | 27.8765167 | 9.52468086 | -1.5493076 | 1.55E-22 | 4.33E-21 |
| DUSP4 | 0.54249848 | 1.42176326 | 1.38999026 | 1.50E-09 | 4.58E-09 |
| SNCAIP | 0.27114218 | 0.95499666 | 1.81644611 | 0.04035728 | 0.04914197 |
| HPR | 245.315304 | 95.2863567 | -1.3642957 | 1.04E-18 | 1.31E-17 |
| PDGFRA | 0.97211712 | 1.95444256 | 1.00755515 | 0.01228908 | 0.01598149 |
| BLMH | 3.48536357 | 7.69348344 | 1.14232781 | 5.29E-17 | 4.78E-16 |
| NUAK2 | 2.55894497 | 5.83118329 | 1.18823955 | 8.15E-09 | 2.25E-08 |
| COL5A1 | 3.99869382 | 8.02618893 | 1.0051863 | 6.10E-06 | 1.18E-05 |
| CLDN11 | 0.40049726 | 0.92029727 | 1.20030757 | 6.65E-06 | 1.28E-05 |
| UPB1 | 31.8095764 | 15.8602413 | -1.0040464 | 3.81E-17 | 3.53E-16 |
| ACE2 | 3.04622017 | 1.27329412 | -1.2584545 | 0.00081901 | 0.00122918 |
| CELF2 | 0.46911853 | 1.02941608 | 1.13380182 | 1.75E-05 | 3.22E-05 |
| TRIM54 | 0.45734316 | 1.10578632 | 1.27372366 | 0.00331179 | 0.00461236 |
| SGPP2 | 0.36923809 | 2.42888275 | 2.71766955 | 8.47E-15 | 5.34E-14 |
| MUC12-AS1 | 0.20836847 | 1.26584346 | 2.60289013 | 7.35E-16 | 5.50E-15 |
| ADGRE5 | 4.40815022 | 10.4659916 | 1.24746371 | 2.54E-17 | 2.43E-16 |
| ST6GALNAC4 | 2.07177224 | 4.74666546 | 1.19604896 | 4.83E-21 | 9.46E-20 |
| HAPLN3 | 0.58615873 | 1.75262961 | 1.58015783 | 8.35E-14 | 4.52E-13 |
| SLC25A36 | 0.36404735 | 1.05381587 | 1.53342479 | 1.94E-09 | 5.82E-09 |
| IGF2BP1 | 1.00627985 | 3.08317291 | 1.61538422 | 2.15E-14 | 1.28E-13 |
| MMP12 | 0.94836271 | 3.10772166 | 1.71234646 | 7.32E-10 | 2.30E-09 |
| PARVG | 0.53110971 | 1.16332375 | 1.13117085 | 2.04E-11 | 7.77E-11 |
| EPCAM | 7.34203097 | 43.0808928 | 2.55279704 | 1.05E-12 | 4.88E-12 |
| HES4 | 2.04647962 | 5.82498288 | 1.50910951 | 2.30E-14 | 1.36E-13 |
| OSBPL7 | 0.38723882 | 0.99836583 | 1.36634498 | 1.54E-16 | 1.29E-15 |
| SEMA4A | 0.32796887 | 0.9922214 | 1.5971032 | 1.96E-22 | 5.27E-21 |
| PFKFB3 | 3.65294038 | 11.6812498 | 1.67706453 | 1.80E-07 | 4.19E-07 |
| AGR2 | 4.37160983 | 16.1383088 | 1.88425285 | 1.69E-07 | 3.94E-07 |
| IGKV1-17 | 3.35367066 | 13.7074746 | 2.03114988 | 0.00069008 | 0.0010448 |
| GBP5 | 0.83536795 | 3.0954857 | 1.8896821 | 8.80E-05 | 0.00014819 |
| ISLR | 3.27629203 | 8.36569007 | 1.35242059 | 6.99E-06 | 1.34E-05 |
| SMARCD3 | 0.88027983 | 3.16625003 | 1.84674107 | 2.55E-16 | 2.06E-15 |
| PLCD3 | 0.55781023 | 2.23118652 | 1.99996481 | 8.16E-14 | 4.43E-13 |
| IGLV6-57 | 2.67111659 | 7.2110351 | 1.43276342 | 0.00120118 | 0.00176901 |
| CDH17 | 0.21324222 | 1.46947996 | 2.78474071 | 9.20E-12 | 3.70E-11 |
| DOK3 | 0.54410806 | 1.29871278 | 1.25511729 | 1.53E-14 | 9.26E-14 |
| ZNF404 | 0.39351071 | 0.97171953 | 1.30413707 | 1.81E-15 | 1.26E-14 |
| DRD4 | 0.51707955 | 1.95163166 | 1.91622264 | 3.21E-05 | 5.70E-05 |
| AC010205.1 | 0.66951637 | 0.32666094 | -1.0353254 | 1.73E-08 | 4.58E-08 |
| SELPLG | 2.89791064 | 5.94698293 | 1.03714483 | 7.49E-09 | 2.08E-08 |
| AC007423.1 | 4.13320897 | 1.44607545 | -1.5151195 | 9.05E-05 | 0.00015216 |
| SCP2 | 56.2884116 | 27.1101077 | -1.0540071 | 5.87E-23 | 1.77E-21 |
| GFRA1 | 4.39800748 | 2.04885222 | -1.1020341 | 1.74E-09 | 5.25E-09 |
| FBLIM1 | 3.5848417 | 9.81769407 | 1.4534748 | 5.30E-20 | 8.47E-19 |
| HID1 | 1.37893692 | 4.6701505 | 1.75991259 | 8.54E-22 | 2.00E-20 |
| FOXJ1 | 0.34242136 | 3.06632623 | 3.1626666 | 3.26E-19 | 4.52E-18 |
| ZNF185 | 0.545306 | 1.28577973 | 1.23750558 | 8.49E-16 | 6.26E-15 |
| CEL | 0.36901496 | 0.76910272 | 1.05949699 | 1.15E-05 | 2.15E-05 |
| HLA-DOA | 1.95323592 | 4.64186773 | 1.2488392 | 2.50E-05 | 4.49E-05 |
| IGHV3-64 | 0.29677798 | 1.18996393 | 2.0034619 | 0.02081125 | 0.02631649 |
| AP000424.2 | 0.37441693 | 0.92937835 | 1.31162036 | 1.68E-05 | 3.09E-05 |
| TFF3 | 4.22083765 | 12.592698 | 1.57698617 | 4.97E-08 | 1.23E-07 |
| AC091133.5 | 0.27604652 | 0.87140755 | 1.6584362 | 4.87E-12 | 2.04E-11 |
| SLC6A6 | 0.86513109 | 2.76004302 | 1.67370009 | 7.79E-13 | 3.67E-12 |
| TPX2 | 5.74334472 | 14.2023984 | 1.30617152 | 1.23E-19 | 1.83E-18 |
| SLC38A1 | 2.58242939 | 7.01292587 | 1.44128757 | 1.42E-12 | 6.46E-12 |
| RBL1 | 0.71174719 | 1.53344049 | 1.10733537 | 8.88E-22 | 2.07E-20 |
| KRT80 | 0.35814561 | 3.05513287 | 3.09261697 | 1.56E-11 | 6.05E-11 |
| TMC6 | 1.37687811 | 4.85979524 | 1.81949468 | 1.83E-21 | 3.96E-20 |
| IGLV7-43 | 1.00919068 | 5.72822001 | 2.50488812 | 0.00165385 | 0.00239712 |
| AC127024.5 | 0.54727015 | 1.35219534 | 1.30497851 | 1.18E-23 | 4.07E-22 |
| AP002807.1 | 0.5804726 | 1.23746346 | 1.09208604 | 3.17E-14 | 1.83E-13 |
| CA4 | 0.78830975 | 0.25524159 | -1.6268992 | 0.00050733 | 0.00077889 |
| ITM2C | 14.2808203 | 33.4654378 | 1.22859303 | 3.38E-21 | 6.86E-20 |
| ZIC5 | 0.26169553 | 0.90201601 | 1.78526373 | 4.24E-12 | 1.80E-11 |
| SEC14L6 | 0.24480818 | 0.98267494 | 2.00506249 | 6.51E-08 | 1.60E-07 |
| PTPRS | 1.08460227 | 2.53868388 | 1.22691467 | 4.95E-05 | 8.60E-05 |
| AOX1 | 160.471077 | 64.9256546 | -1.3054527 | 1.38E-13 | 7.28E-13 |
| SNORD60 | 0.92211015 | 2.08767135 | 1.1788836 | 1.19E-12 | 5.48E-12 |
| CDH16 | 0.62635102 | 1.31769184 | 1.07296971 | 0.00036407 | 0.0005693 |
| MYO10 | 0.65079467 | 2.07884224 | 1.67550594 | 0.00916043 | 0.01210258 |
| ACNATP | 0.72820093 | 0.30952103 | -1.2342992 | 4.40E-13 | 2.15E-12 |
| KIF15 | 0.51204243 | 1.20504462 | 1.23475131 | 2.29E-16 | 1.86E-15 |
| F9 | 92.1624129 | 31.0827476 | -1.5680644 | 5.09E-18 | 5.52E-17 |
| SDC3 | 4.93136844 | 10.0954835 | 1.03365006 | 3.89E-15 | 2.58E-14 |
| BCAS4 | 0.36402004 | 1.02824326 | 1.49809183 | 1.93E-27 | 2.08E-25 |
| IGLV3-10 | 3.05963483 | 22.4404505 | 2.87467026 | 0.00231142 | 0.00328734 |
| CRLF1 | 0.31867869 | 2.02194614 | 2.6655701 | 6.68E-10 | 2.11E-09 |
| KIAA1841 | 0.39728452 | 0.80852469 | 1.02511924 | 5.41E-23 | 1.65E-21 |
| HNF1B | 2.19137115 | 7.12830847 | 1.70172592 | 3.89E-12 | 1.66E-11 |
| AC137723.1 | 1.56417088 | 0.52569378 | -1.5731036 | 4.84E-14 | 2.74E-13 |
| UHRF1 | 0.84038658 | 2.22181546 | 1.40261397 | 3.68E-17 | 3.42E-16 |
| DNASE1L3 | 5.99344966 | 2.9129041 | -1.0409284 | 5.85E-09 | 1.64E-08 |
| CRMP1 | 0.57495751 | 1.84822449 | 1.68461275 | 1.87E-18 | 2.24E-17 |
| CDCA5 | 2.18980484 | 5.41323109 | 1.30568768 | 8.34E-25 | 3.92E-23 |
| COL6A3 | 3.2365997 | 6.58603504 | 1.02493124 | 5.19E-05 | 8.99E-05 |
| SULT2B1 | 0.23087389 | 1.12739696 | 2.28781862 | 1.04E-06 | 2.19E-06 |
| MSRB3 | 1.05504624 | 2.12420364 | 1.00961585 | 7.04E-06 | 1.35E-05 |
| PDIA2 | 1.08198096 | 2.61215699 | 1.2715665 | 4.96E-06 | 9.72E-06 |
| ABAT | 35.1237044 | 14.2474766 | -1.3017386 | 8.97E-28 | 1.05E-25 |
| ZNF213 | 0.80693374 | 1.78824267 | 1.14802041 | 1.71E-20 | 2.97E-19 |
| GSDME | 0.77261603 | 1.61478525 | 1.06351879 | 1.20E-10 | 4.13E-10 |
| AC026401.3 | 2.18658746 | 4.81238874 | 1.13807213 | 3.83E-21 | 7.70E-20 |
| C1QB | 52.246422 | 106.658088 | 1.02958923 | 5.30E-05 | 9.17E-05 |
| AC048341.2 | 0.67684906 | 1.67503358 | 1.30728398 | 2.70E-21 | 5.61E-20 |
| FXYD5 | 7.12981774 | 15.4662469 | 1.11718605 | 2.64E-12 | 1.15E-11 |
| SPINDOC | 2.00914499 | 5.3092534 | 1.40192732 | 2.71E-28 | 3.70E-26 |
| DSCC1 | 1.12988463 | 2.3145185 | 1.03453662 | 1.53E-17 | 1.52E-16 |
| DMPK | 1.52821521 | 3.6738736 | 1.26545426 | 4.53E-25 | 2.29E-23 |
| SH3YL1 | 0.64990255 | 2.17135711 | 1.74030171 | 0.0001514 | 0.00024751 |
| MT1E | 145.769651 | 57.980178 | -1.3300587 | 0.0022612 | 0.00322013 |
| CTSC | 5.09870881 | 10.9039431 | 1.09664608 | 6.06E-12 | 2.51E-11 |
| AC010547.2 | 0.32490999 | 2.4065961 | 2.88888204 | 3.49E-05 | 6.18E-05 |
| MYL6B | 5.28997599 | 11.0129536 | 1.05786837 | 4.75E-23 | 1.46E-21 |
| MNX1-AS1 | 0.15947215 | 1.01818842 | 2.67462815 | 2.28E-14 | 1.35E-13 |
| VEGFB | 20.0983582 | 43.3208041 | 1.10798237 | 4.05E-17 | 3.73E-16 |
| PNMA1 | 3.25668832 | 8.49156813 | 1.38262534 | 2.27E-22 | 6.00E-21 |
| SPC25 | 1.15688325 | 2.9924503 | 1.37108401 | 6.02E-25 | 2.98E-23 |
| MTMR7 | 0.46304058 | 1.38608715 | 1.58180744 | 7.44E-09 | 2.06E-08 |
| IGHD | 0.79788556 | 2.16187428 | 1.43802888 | 0.00027816 | 0.00044083 |
| BPIFB2 | 5.39370345 | 12.5933812 | 1.22331757 | 1.15E-08 | 3.10E-08 |
| AL354872.1 | 11.4328068 | 5.52290609 | -1.0496801 | 1.17E-20 | 2.10E-19 |
| AC083809.1 | 1.29954118 | 4.7609928 | 1.8732601 | 0.00656094 | 0.0088363 |
| AKR1C6P | 6.01214435 | 2.75466968 | -1.1260003 | 2.65E-17 | 2.53E-16 |
| IGHV3-49 | 2.71265261 | 18.4671518 | 2.76718517 | 0.00289411 | 0.00406397 |
| RNF144A | 0.65208186 | 1.72119602 | 1.40028641 | 5.04E-18 | 5.47E-17 |
| SLC22A1 | 116.394774 | 32.8453417 | -1.8252656 | 1.31E-15 | 9.43E-15 |
| CDC42EP1 | 40.9660655 | 82.5914686 | 1.01156342 | 1.69E-20 | 2.94E-19 |
| SRRM3 | 0.28815998 | 1.317938 | 2.19334062 | 1.24E-10 | 4.24E-10 |
| METRNL | 3.25114712 | 7.69967208 | 1.24384816 | 1.31E-11 | 5.13E-11 |
| UCHL1 | 2.73835509 | 14.3126126 | 2.3859056 | 6.62E-11 | 2.35E-10 |
| VASP | 12.220391 | 25.6349239 | 1.06882017 | 1.77E-30 | 6.23E-28 |
| MEP1A | 1.94492774 | 5.95143786 | 1.61352171 | 2.79E-10 | 9.18E-10 |
| TRIM31 | 2.06003647 | 4.43084885 | 1.10491324 | 6.90E-10 | 2.18E-09 |
| HJV | 110.730012 | 54.660899 | -1.0184652 | 1.17E-14 | 7.22E-14 |
| PLEK | 2.51497941 | 5.72854473 | 1.1876221 | 2.44E-06 | 4.94E-06 |
| AP1G2 | 1.22952438 | 2.93289346 | 1.25422433 | 2.80E-18 | 3.23E-17 |
| ANXA1 | 4.43400558 | 8.94774478 | 1.01291352 | 0.00184351 | 0.00265427 |
| SLC35F2 | 0.3128463 | 1.14591118 | 1.87296927 | 7.27E-14 | 3.99E-13 |
| TRIP13 | 0.91789953 | 3.11103278 | 1.76098545 | 3.91E-24 | 1.53E-22 |
| LINC01554 | 28.7719301 | 6.87738905 | -2.0647291 | 7.34E-13 | 3.47E-12 |
| DUOX1 | 0.34576062 | 0.73537777 | 1.088712 | 1.19E-09 | 3.67E-09 |
| AP000424.1 | 0.51133159 | 1.04622791 | 1.03286611 | 0.00201115 | 0.00288151 |
| OSMR | 2.93601419 | 6.11911131 | 1.0594632 | 3.82E-05 | 6.73E-05 |
| DMBT1 | 0.04483794 | 2.11270764 | 5.55822929 | 5.53E-14 | 3.10E-13 |
| AP006216.2 | 1.9203049 | 0.93417057 | -1.0395775 | 1.89E-12 | 8.41E-12 |
| SALL2 | 0.50978786 | 1.6655392 | 1.70802038 | 7.15E-20 | 1.12E-18 |
| CYP4A11 | 100.229622 | 34.1978954 | -1.5513295 | 1.42E-24 | 6.29E-23 |
| SPIB | 0.10858452 | 2.1729995 | 4.32279752 | 1.46E-19 | 2.16E-18 |
| SCX | 0.62640161 | 1.77132677 | 1.49967056 | 9.33E-19 | 1.18E-17 |
| APOL5 | 1.02892824 | 0.51398683 | -1.0013391 | 0.03630221 | 0.0444268 |
| RHOV | 0.20976252 | 3.20893339 | 3.93526504 | 4.55E-18 | 4.98E-17 |
| COL16A1 | 0.77677054 | 2.05746714 | 1.405309 | 3.61E-11 | 1.33E-10 |
| AGPAT4 | 0.36155618 | 0.87584626 | 1.27645783 | 7.88E-15 | 5.01E-14 |
| ADRA1A | 1.25210747 | 0.40890692 | -1.614514 | 1.00E-10 | 3.50E-10 |
| SLC22A15 | 0.49795217 | 1.19601885 | 1.26416105 | 8.70E-11 | 3.06E-10 |
| CD72 | 0.79181148 | 1.61588544 | 1.02909603 | 4.40E-05 | 7.70E-05 |
| GABBR1 | 0.31113254 | 1.18989797 | 1.9352367 | 1.28E-07 | 3.02E-07 |
| CYP3A43 | 1.94394304 | 0.65700388 | -1.5650121 | 5.12E-11 | 1.84E-10 |
| SYK | 0.94189806 | 2.79651474 | 1.56998711 | 2.15E-13 | 1.10E-12 |
| MYO1A | 0.54065587 | 1.24141736 | 1.19920572 | 7.28E-06 | 1.39E-05 |
| PFKM | 1.54495291 | 3.11714559 | 1.01266267 | 2.09E-05 | 3.79E-05 |
| MAP4K1 | 0.77366734 | 1.69603096 | 1.13237723 | 5.44E-09 | 1.53E-08 |
| PTGES | 1.60557342 | 5.69215281 | 1.82588575 | 1.96E-12 | 8.71E-12 |
| LINC01116 | 0.21322296 | 0.94890507 | 2.153901 | 4.07E-05 | 7.15E-05 |
| LINC01018 | 16.6696804 | 4.60638544 | -1.8555194 | 5.89E-19 | 7.79E-18 |
| CDCA8 | 2.47243082 | 5.93464677 | 1.26323201 | 3.20E-18 | 3.65E-17 |
| PROM2 | 0.0826264 | 1.24061613 | 3.9083102 | 4.86E-13 | 2.36E-12 |
| NUGGC | 4.81532021 | 2.2361843 | -1.1065926 | 1.91E-13 | 9.83E-13 |
| PSMC3IP | 0.52983383 | 1.20964186 | 1.19096811 | 1.26E-18 | 1.56E-17 |
| CHIT1 | 0.47116391 | 1.94627584 | 2.04641524 | 1.75E-06 | 3.60E-06 |
| STK26 | 1.48190758 | 3.33708094 | 1.1711312 | 9.77E-16 | 7.15E-15 |
| ARHGAP4 | 3.95285039 | 8.11854164 | 1.03832724 | 1.21E-13 | 6.40E-13 |
| PWWP2B | 3.81182451 | 8.8833476 | 1.22062174 | 3.74E-20 | 6.12E-19 |
| CTSV | 0.57161411 | 2.70041987 | 2.24007031 | 1.58E-21 | 3.46E-20 |
| SEC14L2 | 35.1036556 | 10.3588364 | -1.7607593 | 1.55E-22 | 4.33E-21 |
| CYP2S1 | 0.82563629 | 1.91701564 | 1.21528382 | 9.34E-08 | 2.25E-07 |
| ESR1 | 0.81508301 | 0.18886314 | -2.1096058 | 2.08E-15 | 1.44E-14 |
| LINC02037 | 1.77642656 | 0.82996818 | -1.0978501 | 2.89E-14 | 1.68E-13 |
| HVCN1 | 0.53220052 | 1.06975925 | 1.00724433 | 7.58E-09 | 2.10E-08 |
| CALHM6 | 2.36502588 | 5.85879095 | 1.308747 | 6.01E-07 | 1.31E-06 |
| S100A2 | 0.31362784 | 1.09083152 | 1.79830276 | 1.81E-15 | 1.26E-14 |
| TRPV2 | 1.99866023 | 4.75530239 | 1.25050385 | 4.65E-12 | 1.96E-11 |
| FZD7 | 0.60727154 | 1.96078598 | 1.6910184 | 2.19E-12 | 9.63E-12 |
| MT1A | 43.8902893 | 17.4164733 | -1.3334493 | 0.00011983 | 0.00019856 |
| BSPRY | 0.86892108 | 3.08227138 | 1.82669683 | 4.52E-07 | 9.99E-07 |
| C14orf132 | 0.44385687 | 1.06956353 | 1.26885574 | 7.81E-08 | 1.90E-07 |
| CENPH | 1.42682046 | 3.06211472 | 1.10172453 | 1.18E-23 | 4.07E-22 |
| TIMP2 | 11.1988368 | 30.5690634 | 1.44872346 | 3.06E-09 | 8.92E-09 |
| SNORD99 | 1.29107062 | 3.34495851 | 1.3734204 | 3.46E-18 | 3.89E-17 |
| ENPP3 | 0.97262224 | 2.18816884 | 1.16977257 | 0.00305196 | 0.00427244 |
| ADGRG1 | 2.0185249 | 5.67081647 | 1.49025508 | 2.72E-05 | 4.87E-05 |
| MYLIP | 1.78294408 | 3.64880806 | 1.0331638 | 1.90E-12 | 8.48E-12 |
| HMCN2 | 0.64463873 | 0.28860916 | -1.1593738 | 0.00025206 | 0.00040164 |
| DDX39A | 10.6920099 | 21.8156032 | 1.02882729 | 1.90E-30 | 6.53E-28 |
| TGFA | 0.60028525 | 2.55421214 | 2.08915822 | 2.60E-11 | 9.76E-11 |
| B3GNT3 | 3.49840479 | 14.1210349 | 2.01307669 | 1.86E-11 | 7.12E-11 |
| APOA5 | 144.165653 | 64.4780048 | -1.1608485 | 1.22E-16 | 1.04E-15 |
| PNMA3 | 0.88381478 | 2.87027889 | 1.69937496 | 1.96E-08 | 5.14E-08 |
| SLC46A3 | 14.5722785 | 5.81527314 | -1.3253076 | 5.42E-14 | 3.04E-13 |
| DSG2 | 4.12590126 | 11.4312811 | 1.4702059 | 6.30E-10 | 2.00E-09 |
| FUT8 | 0.41678857 | 1.05298125 | 1.33709211 | 1.14E-10 | 3.94E-10 |
| AL356356.1 | 0.54443883 | 1.20231319 | 1.14297088 | 7.12E-09 | 1.98E-08 |
| C5orf46 | 0.32968999 | 0.99898202 | 1.59934861 | 1.39E-08 | 3.72E-08 |
| C6orf141 | 0.37758726 | 1.734852 | 2.19993062 | 0.01797496 | 0.02292735 |
| PEG10 | 17.1107766 | 53.0541784 | 1.63256114 | 1.98E-09 | 5.92E-09 |
| AL590326.1 | 0.53976668 | 1.10470821 | 1.03325755 | 2.71E-10 | 8.93E-10 |
| KIF18B | 0.87440285 | 2.61527564 | 1.580593 | 1.08E-23 | 3.82E-22 |
| ZAP70 | 0.61437873 | 1.3119282 | 1.0944886 | 5.68E-06 | 1.10E-05 |
| BHMT | 122.329098 | 43.4443592 | -1.4935268 | 4.87E-15 | 3.19E-14 |
| GAST | 1.48061143 | 5.01892569 | 1.76118551 | 1.12E-10 | 3.88E-10 |
| FAM241B | 2.10255184 | 5.34181161 | 1.34518772 | 1.39E-21 | 3.08E-20 |
| LINC01139 | 0.39940539 | 1.59114288 | 1.99413767 | 0.00158019 | 0.0022953 |
| NCAPG | 1.39183483 | 3.46235786 | 1.31476683 | 2.44E-19 | 3.45E-18 |
| C4orf48 | 1.71659655 | 6.5185747 | 1.92500554 | 6.34E-17 | 5.63E-16 |
| PHLDA3 | 4.79139914 | 9.72288822 | 1.02093794 | 0.01473107 | 0.01896128 |
| CCNB2 | 2.39107461 | 6.41499144 | 1.4237882 | 7.97E-24 | 2.92E-22 |
| CD300C | 0.36396173 | 0.75612855 | 1.05484476 | 8.91E-09 | 2.45E-08 |
| DPEP1 | 0.53046177 | 1.60841165 | 1.600316 | 3.27E-07 | 7.33E-07 |
| NR1I2 | 7.42033376 | 3.16832148 | -1.2277654 | 3.68E-17 | 3.42E-16 |
| PAGE4 | 21.9880018 | 3.33620529 | -2.7204365 | 1.85E-06 | 3.79E-06 |
| STX3 | 2.56115632 | 5.94859531 | 1.21575372 | 2.21E-17 | 2.13E-16 |
| VPS37B | 1.5269384 | 3.19356233 | 1.06452475 | 4.30E-19 | 5.86E-18 |
| IGKV1-8 | 0.43804761 | 1.86784698 | 2.09221667 | 0.01007007 | 0.01323863 |
| LAD1 | 14.9704351 | 37.9544785 | 1.34215398 | 1.17E-07 | 2.78E-07 |
| AC009275.1 | 0.48577029 | 1.09176535 | 1.16831664 | 5.42E-06 | 1.06E-05 |
| MACROH2A2 | 4.59721078 | 15.0167739 | 1.70774419 | 2.98E-19 | 4.18E-18 |
| TMEM130 | 0.04601984 | 1.25212368 | 4.76597743 | 7.36E-09 | 2.04E-08 |
| TCEAL3 | 1.61095574 | 3.90444522 | 1.27720072 | 4.64E-15 | 3.05E-14 |
| KCNN4 | 0.28592018 | 1.06345773 | 1.89507836 | 4.33E-13 | 2.12E-12 |
| TBC1D30 | 0.38627464 | 0.77375997 | 1.00225914 | 2.40E-09 | 7.10E-09 |
| IGHV3-23 | 12.4316456 | 39.9977849 | 1.68590283 | 0.00135096 | 0.00197742 |
| FPR1 | 0.633717 | 1.41560554 | 1.1595087 | 3.53E-06 | 7.02E-06 |
| SLC5A1 | 0.21347746 | 0.93772274 | 2.13507763 | 7.35E-06 | 1.41E-05 |
| SERPINI1 | 1.75543958 | 4.59623686 | 1.3886208 | 4.15E-12 | 1.76E-11 |
| RTL8A | 13.6963782 | 28.0591983 | 1.03467934 | 3.75E-19 | 5.15E-18 |
| HNF4A-AS1 | 7.89864601 | 3.09802084 | -1.3502585 | 8.81E-15 | 5.53E-14 |
| SLC2A6 | 2.04196568 | 4.73915008 | 1.21466973 | 1.97E-16 | 1.61E-15 |
| ACSM2A | 31.2163525 | 10.0493112 | -1.6352054 | 8.53E-33 | 6.33E-30 |
| IGKV3D-11 | 0.21810458 | 1.24379532 | 2.51165713 | 0.00328542 | 0.00457744 |
| TMEM74B | 1.42125155 | 3.21299246 | 1.17675567 | 6.32E-12 | 2.61E-11 |
| CLIC1 | 63.1665781 | 151.862955 | 1.26553666 | 1.32E-30 | 5.03E-28 |
| MAMSTR | 0.3747348 | 1.10418806 | 1.55904402 | 1.58E-19 | 2.31E-18 |
| OTULINL | 0.53378434 | 1.10121356 | 1.04476539 | 7.05E-09 | 1.96E-08 |
| CYP2B7P | 28.1456737 | 6.65174822 | -2.0811077 | 3.20E-10 | 1.05E-09 |
| SNORA26 | 0.52545959 | 1.1781976 | 1.16492979 | 5.62E-10 | 1.79E-09 |
| FANCD2 | 0.65995299 | 1.5221172 | 1.20564428 | 5.12E-22 | 1.26E-20 |
| C20orf204 | 0.81648115 | 1.6973613 | 1.05580221 | 7.22E-08 | 1.76E-07 |
| CXCL1 | 4.43049326 | 15.4202701 | 1.7992888 | 6.31E-06 | 1.22E-05 |
| ADRA2C | 1.86887797 | 4.6469463 | 1.3141106 | 3.94E-07 | 8.75E-07 |
| FBLL1 | 0.76740568 | 1.75953715 | 1.19713463 | 3.02E-06 | 6.06E-06 |
| FBXO5 | 0.74358919 | 1.58962703 | 1.09611061 | 9.48E-17 | 8.19E-16 |
| PLGLA | 2.64589169 | 0.96127713 | -1.4607297 | 7.92E-12 | 3.22E-11 |
| CLSTN1 | 6.96506939 | 16.9017522 | 1.27896319 | 1.68E-22 | 4.65E-21 |
| FAR1 | 0.58773048 | 1.44818865 | 1.30102293 | 1.88E-08 | 4.95E-08 |
| SNHG1 | 3.90980611 | 8.94946187 | 1.19470387 | 1.93E-29 | 4.32E-27 |
| PAPLN | 1.27027179 | 3.43290341 | 1.43429206 | 1.00E-11 | 4.00E-11 |
| RAD51AP1 | 1.20987544 | 2.54083748 | 1.07044557 | 2.06E-15 | 1.43E-14 |
| NCAPH | 1.20824344 | 3.26024743 | 1.4320703 | 5.91E-21 | 1.13E-19 |
| SCAMP5 | 2.78058475 | 5.81807148 | 1.06515271 | 9.92E-10 | 3.09E-09 |
| MAMLD1 | 0.84613716 | 2.18332304 | 1.36756215 | 1.17E-06 | 2.45E-06 |
| UPK3A | 6.02071191 | 15.3312677 | 1.348471 | 3.18E-12 | 1.37E-11 |
| TBX2-AS1 | 0.49992974 | 1.15618158 | 1.20957074 | 3.88E-07 | 8.61E-07 |
| IGLV3-1 | 5.82454191 | 18.2440771 | 1.64721168 | 0.00015553 | 0.00025397 |
| DMGDH | 20.8507572 | 7.75203055 | -1.4274536 | 9.76E-25 | 4.47E-23 |
| EFNA5 | 0.17568179 | 1.34800065 | 2.9397846 | 3.95E-12 | 1.68E-11 |
| LINC02381 | 0.68640565 | 4.57702105 | 2.73727558 | 1.18E-17 | 1.19E-16 |
| ZDHHC13 | 0.34109716 | 1.13020298 | 1.72832726 | 2.68E-21 | 5.56E-20 |
| CDCA3 | 0.84251915 | 2.41629814 | 1.5200171 | 1.42E-25 | 8.56E-24 |
| AC099850.3 | 1.17790913 | 3.35006868 | 1.50796243 | 8.04E-16 | 5.98E-15 |
| IGF1R | 0.29036096 | 0.93235677 | 1.68303461 | 1.04E-06 | 2.19E-06 |
| IL4I1 | 1.03409823 | 3.21181627 | 1.63501612 | 2.95E-21 | 6.06E-20 |
| APOC4 | 0.98085765 | 0.38914838 | -1.3337234 | 1.07E-16 | 9.18E-16 |
| CUZD1 | 0.06764245 | 1.24674741 | 4.20409653 | 1.36E-12 | 6.20E-12 |
| PLK1 | 1.55836163 | 4.5060739 | 1.53184091 | 3.49E-24 | 1.38E-22 |
| CBX2 | 0.59811238 | 1.72327263 | 1.52666248 | 1.28E-13 | 6.78E-13 |
| CEMIP | 0.58008303 | 1.44309913 | 1.31483909 | 0.00122138 | 0.00179726 |
| KCNJ4 | 2.35992135 | 1.12319134 | -1.0711351 | 1.37E-07 | 3.24E-07 |
| SNRPN | 10.6531393 | 25.5772009 | 1.26357976 | 2.32E-13 | 1.18E-12 |
| IER3 | 16.1912432 | 40.6113547 | 1.32666939 | 2.02E-13 | 1.04E-12 |
| AL390719.1 | 0.6910618 | 1.45894485 | 1.07803871 | 1.11E-11 | 4.41E-11 |
| ADORA2BP1 | 2.76430333 | 0.9383429 | -1.5587288 | 3.85E-16 | 3.01E-15 |
| TGFBR3L | 1.89211485 | 0.92531629 | -1.0319812 | 8.61E-11 | 3.03E-10 |
| CLIP3 | 0.90589072 | 2.25129335 | 1.31334513 | 3.98E-11 | 1.46E-10 |
| MYRF | 3.94971847 | 9.74153733 | 1.30239964 | 4.18E-12 | 1.77E-11 |
| ARHGAP39 | 0.64223643 | 1.42102485 | 1.14575538 | 1.11E-17 | 1.13E-16 |
| H1-12P | 0.51530316 | 1.033616 | 1.00420695 | 0.00124819 | 0.00183461 |
| HENMT1 | 0.7942253 | 1.69501646 | 1.09367906 | 1.14E-06 | 2.39E-06 |
| ABCC1 | 1.32466199 | 3.98825586 | 1.59013369 | 2.11E-15 | 1.46E-14 |
| FMOD | 3.27377591 | 6.88343396 | 1.07217289 | 2.11E-06 | 4.30E-06 |
| ACSS1 | 1.27670019 | 3.73139253 | 1.54729436 | 2.24E-18 | 2.63E-17 |
| FGD3 | 0.53868119 | 1.66507997 | 1.62808788 | 6.98E-19 | 9.02E-18 |
| MAFG-DT | 0.89662163 | 2.25873938 | 1.33294661 | 2.77E-15 | 1.88E-14 |
| LHFPL2 | 1.13838345 | 2.63970999 | 1.21339285 | 8.84E-19 | 1.13E-17 |
| ZNF239 | 0.39000086 | 1.22152404 | 1.64713303 | 1.35E-16 | 1.14E-15 |
| EFNA3 | 1.16050037 | 2.50686935 | 1.11113984 | 2.93E-13 | 1.47E-12 |
| LGALS9 | 3.5198569 | 8.62221977 | 1.29254256 | 6.77E-15 | 4.34E-14 |
| IL12RB1 | 0.41963167 | 0.895838 | 1.0941143 | 1.46E-07 | 3.43E-07 |
| GRAMD1A | 5.2151617 | 12.9708833 | 1.31449284 | 1.17E-24 | 5.19E-23 |
| CXCL5 | 1.1328761 | 6.80802689 | 2.58724665 | 2.59E-08 | 6.69E-08 |
| DDX11 | 1.00694654 | 2.32716653 | 1.20858737 | 2.00E-21 | 4.27E-20 |
| SNHG25 | 2.81713924 | 8.3040178 | 1.55957866 | 1.45E-14 | 8.86E-14 |
| HSD17B13 | 77.8568676 | 19.0250158 | -2.0329267 | 8.47E-09 | 2.33E-08 |
| SNX25P1 | 0.40047073 | 0.8607634 | 1.10391995 | 1.36E-15 | 9.72E-15 |
| LRRC1 | 1.36537506 | 3.89520799 | 1.51240306 | 1.01E-21 | 2.31E-20 |
| CCL2 | 5.54047653 | 11.3164638 | 1.03034124 | 0.00087786 | 0.00131162 |
| IGKC | 80.747921 | 340.703739 | 2.07702076 | 5.16E-06 | 1.01E-05 |
| GLIS3 | 0.51191451 | 1.68620196 | 1.71980253 | 2.28E-07 | 5.22E-07 |
| TTYH3 | 9.57746313 | 19.2643467 | 1.00821779 | 1.24E-11 | 4.91E-11 |
| AMPD3 | 0.3379307 | 0.85117699 | 1.33273174 | 3.07E-15 | 2.07E-14 |
| XCL1 | 0.3214236 | 1.08982367 | 1.76154696 | 1.51E-12 | 6.83E-12 |
| CALML3 | 0.87754407 | 0.41270009 | -1.0883778 | 0.00175077 | 0.00252803 |
| CA11 | 1.07599532 | 2.41778311 | 1.16801304 | 2.60E-11 | 9.76E-11 |
| IGF2BP2 | 2.46195557 | 6.23168364 | 1.33981727 | 5.27E-18 | 5.70E-17 |
| IGLV1-51 | 8.33602013 | 36.6845426 | 2.13774163 | 0.0001629 | 0.00026523 |
| PAGE2 | 4.92788205 | 13.104615 | 1.41103534 | 0.00014876 | 0.00024348 |
| CST2 | 0.35506686 | 1.09644357 | 1.62666893 | 1.16E-06 | 2.43E-06 |
| MSLN | 0.10647337 | 2.48655855 | 4.54558579 | 0.0003651 | 0.00057085 |
| PLEKHB1 | 0.2517988 | 3.6513596 | 3.85809043 | 8.05E-24 | 2.93E-22 |
| WDR62 | 0.42573542 | 1.33835727 | 1.65243426 | 3.43E-29 | 6.71E-27 |
| PODXL2 | 2.72053174 | 8.43906661 | 1.63319478 | 3.04E-11 | 1.13E-10 |
| CD38 | 0.4925449 | 1.21629734 | 1.30416881 | 0.00902591 | 0.01193716 |
| PLEKHB2 | 4.03096787 | 8.43239058 | 1.06481541 | 6.46E-18 | 6.82E-17 |
| CDCA7L | 1.07781059 | 2.72708829 | 1.33925774 | 3.64E-11 | 1.34E-10 |
| LAMB1 | 6.91941034 | 16.6239505 | 1.26454226 | 7.39E-12 | 3.02E-11 |
| SMIM22 | 0.14984625 | 3.06231765 | 4.35306902 | 6.12E-13 | 2.93E-12 |
| PLAU | 2.12240898 | 6.85085367 | 1.69058109 | 1.70E-10 | 5.74E-10 |
| MELK | 1.59877108 | 3.90219844 | 1.28732376 | 1.23E-19 | 1.83E-18 |
| TLDC2 | 0.67692814 | 2.05060648 | 1.59897607 | 2.65E-18 | 3.07E-17 |
| CYBB | 2.71209824 | 6.26914368 | 1.20885895 | 9.85E-07 | 2.09E-06 |
| MTND4P20 | 14.5125186 | 6.40166586 | -1.1807786 | 1.50E-12 | 6.80E-12 |
| DLK1 | 17.2971432 | 44.3635595 | 1.35884134 | 0.01142813 | 0.01491965 |
| TNC | 1.55100379 | 4.51534689 | 1.54163462 | 8.26E-05 | 0.00013971 |
| TRPV4 | 1.25727503 | 3.11668287 | 1.30971109 | 3.07E-08 | 7.82E-08 |
| LEPR | 18.4226905 | 5.58588853 | -1.7216251 | 3.42E-17 | 3.21E-16 |
| PRAMEF33 | 0.76841095 | 0.29010343 | -1.4053107 | 2.79E-08 | 7.15E-08 |
| PDE9A | 1.02849096 | 3.44913062 | 1.74570365 | 8.82E-15 | 5.53E-14 |
| RAC2 | 4.37565407 | 11.1911971 | 1.35479377 | 1.32E-11 | 5.17E-11 |
| SFXN3 | 2.13911821 | 5.15498304 | 1.26895147 | 6.02E-18 | 6.42E-17 |
| MT2A | 460.556819 | 202.161769 | -1.187869 | 0.00029848 | 0.00047108 |
| AZGP1 | 344.345106 | 166.32119 | -1.0498832 | 4.85E-19 | 6.51E-18 |
| PDE7A | 0.91157905 | 2.20243519 | 1.27265989 | 2.61E-24 | 1.06E-22 |
| PIK3CD-AS2 | 0.38152815 | 1.2432055 | 1.70420339 | 1.81E-17 | 1.78E-16 |
| ARL14 | 0.6003038 | 3.72940567 | 2.63518104 | 4.75E-07 | 1.05E-06 |
| MIR210HG | 0.87255837 | 2.23265037 | 1.35543379 | 1.02E-10 | 3.55E-10 |
| CAPN6 | 0.51203757 | 2.92751771 | 2.51535633 | 6.60E-10 | 2.09E-09 |
| TPM3P9 | 0.95249284 | 2.483548 | 1.38262247 | 3.15E-21 | 6.44E-20 |
| LINC01671 | 0.37168216 | 1.15672973 | 1.63791048 | 8.00E-12 | 3.24E-11 |
| KRT7 | 9.75710482 | 24.6539109 | 1.33729149 | 0.02190032 | 0.02760698 |
| MAGEA6 | 3.15694474 | 6.98329338 | 1.14537858 | 0.02570742 | 0.03208478 |
| NES | 5.21162821 | 10.5942566 | 1.02347628 | 1.38E-09 | 4.21E-09 |
| CSGALNACT1 | 0.94904671 | 1.99737801 | 1.07355639 | 0.00148221 | 0.00216077 |
| IGKV3-20 | 22.6098617 | 116.250737 | 2.36221579 | 0.00042106 | 0.00065349 |
| AP003555.2 | 0.89985125 | 0.38959594 | -1.2077079 | 2.25E-06 | 4.58E-06 |
| CDKN3 | 3.36101886 | 7.93846823 | 1.23996202 | 1.84E-18 | 2.21E-17 |
| FOXQ1 | 2.76890583 | 6.3721378 | 1.20246148 | 8.91E-08 | 2.15E-07 |
| CRYAB | 4.70968816 | 11.7470956 | 1.31860065 | 0.00137226 | 0.00200651 |
| IGLV10-54 | 0.61258309 | 4.99413206 | 3.02725653 | 0.00604895 | 0.00818664 |
| GCNT3 | 0.51198355 | 2.84544157 | 2.4744832 | 1.31E-12 | 6.00E-12 |
| B3GNT9 | 0.72099673 | 1.7843781 | 1.30735673 | 1.67E-11 | 6.45E-11 |
| CRACR2B | 1.30174009 | 3.16777079 | 1.28302653 | 1.39E-08 | 3.72E-08 |
| MYRIP | 6.24001393 | 2.71635806 | -1.1998756 | 3.04E-13 | 1.52E-12 |
| SAPCD2 | 0.42105488 | 1.80906798 | 2.10316644 | 1.29E-29 | 3.08E-27 |
| FOXM1 | 2.44264388 | 6.20947154 | 1.34602695 | 4.33E-18 | 4.77E-17 |
| ALDH1L1 | 50.6132325 | 25.2603353 | -1.0026408 | 2.59E-14 | 1.52E-13 |
| SULF1 | 1.33046312 | 3.48447069 | 1.389011 | 5.26E-06 | 1.03E-05 |
| NEIL3 | 0.43749048 | 1.09994916 | 1.33011332 | 1.03E-14 | 6.44E-14 |
| RCAN3 | 0.73386053 | 1.59694476 | 1.1217366 | 3.38E-18 | 3.82E-17 |
| RAB3D | 1.09859203 | 2.60846398 | 1.24754479 | 3.92E-11 | 1.44E-10 |
| SNORD104 | 20.9860918 | 48.795017 | 1.21730031 | 2.15E-12 | 9.51E-12 |
| ADH1C | 318.923088 | 114.264887 | -1.4808264 | 2.71E-14 | 1.59E-13 |
| APOF | 47.9788699 | 15.3666194 | -1.6425994 | 2.51E-16 | 2.03E-15 |
| LAIR1 | 1.00142292 | 2.2229907 | 1.15045053 | 1.90E-10 | 6.37E-10 |
| AC239859.5 | 0.35431958 | 1.36404006 | 1.94476292 | 1.20E-08 | 3.23E-08 |
| AL109933.4 | 1.14665241 | 0.55376657 | -1.0500783 | 1.94E-11 | 7.42E-11 |
| IGKV1-6 | 1.79064609 | 13.0025514 | 2.86024261 | 0.00037835 | 0.00059039 |
| BEX2 | 3.76194083 | 11.4413532 | 1.60470862 | 5.31E-18 | 5.74E-17 |
| PDCD1 | 0.53022204 | 2.39889408 | 2.1777009 | 5.38E-14 | 3.02E-13 |
| TROAP | 1.40973743 | 4.2946541 | 1.60711546 | 4.09E-26 | 2.81E-24 |
| DTYMK | 7.98412562 | 16.0806123 | 1.01011601 | 2.11E-26 | 1.62E-24 |
| PFKFB4 | 0.47548442 | 1.17934208 | 1.31051227 | 1.89E-13 | 9.76E-13 |
| STIL | 0.49503585 | 0.99664119 | 1.00954118 | 4.01E-15 | 2.66E-14 |
| LINC01503 | 0.87923146 | 2.26518008 | 1.36531083 | 4.75E-11 | 1.72E-10 |
| ETNPPL | 29.5599654 | 10.7129608 | -1.4642873 | 1.18E-19 | 1.78E-18 |
| TRNP1 | 6.87216934 | 18.5689087 | 1.43405154 | 1.76E-12 | 7.86E-12 |
| DUOX2 | 1.47449984 | 9.16244796 | 2.63550743 | 0.00081684 | 0.00122605 |
| TTYH1 | 0.0665334 | 1.43146159 | 4.42726634 | 6.60E-14 | 3.65E-13 |
| SH2B2 | 0.80301687 | 1.81534066 | 1.1767381 | 1.75E-15 | 1.23E-14 |
| AC026403.1 | 6.6642839 | 15.1182626 | 1.18177058 | 1.44E-09 | 4.39E-09 |
| AC027796.4 | 0.36007988 | 0.90228819 | 1.32527129 | 1.30E-12 | 5.94E-12 |
| AC004832.4 | 0.6829267 | 0.29962049 | -1.1885944 | 1.30E-11 | 5.13E-11 |
| H2AC11 | 2.14763786 | 1.02003223 | -1.074136 | 0.00065193 | 0.00098951 |
| GYS2 | 18.4252661 | 4.194324 | -2.1351752 | 1.61E-28 | 2.44E-26 |
| MELTF | 0.57879476 | 2.97011079 | 2.35939299 | 4.18E-21 | 8.33E-20 |
| PPP1R1B | 0.2271146 | 1.68584546 | 2.89197991 | 4.82E-08 | 1.20E-07 |
| LINC01702 | 19.2173901 | 7.86825562 | -1.2882967 | 8.74E-14 | 4.72E-13 |
| ENTPD2 | 1.06710887 | 3.3014765 | 1.629404 | 1.62E-12 | 7.30E-12 |
| AC009005.1 | 0.90629066 | 2.11996362 | 1.22599378 | 2.19E-11 | 8.28E-11 |
| MYO1G | 0.35364854 | 0.82506752 | 1.22219587 | 6.23E-11 | 2.22E-10 |
| RPP25 | 1.85762966 | 3.83052588 | 1.04407956 | 3.11E-11 | 1.16E-10 |
| AC136601.2 | 2.08535984 | 0.72110549 | -1.5320141 | 2.83E-13 | 1.41E-12 |
| PACRG | 1.00004419 | 0.44601473 | -1.1649005 | 3.79E-06 | 7.51E-06 |
| HOXA5 | 0.49689028 | 1.06447073 | 1.09913707 | 1.07E-05 | 2.02E-05 |
| AC011445.1 | 1.67517153 | 4.4980427 | 1.42498853 | 2.23E-10 | 7.42E-10 |
| TRAF5 | 0.52118007 | 1.35590254 | 1.37939966 | 2.66E-23 | 8.50E-22 |
| BICDL1 | 1.77727518 | 3.71481914 | 1.0636249 | 3.35E-09 | 9.71E-09 |
| RGS4 | 0.48657176 | 1.63063201 | 1.74470674 | 3.21E-05 | 5.70E-05 |
| CCL19 | 9.74740423 | 28.721912 | 1.55906181 | 0.00596245 | 0.00807345 |
| GAS7 | 0.4009634 | 1.20816716 | 1.59127762 | 2.03E-12 | 9.01E-12 |
| CLIC6 | 0.39863282 | 1.00722738 | 1.33725699 | 1.90E-08 | 5.00E-08 |
| LRFN4 | 0.49976498 | 1.88659969 | 1.91646661 | 1.72E-11 | 6.61E-11 |
| PLAUR | 0.88523629 | 3.18951353 | 1.84920189 | 9.82E-20 | 1.51E-18 |
| F11-AS1 | 2.79467053 | 1.32222904 | -1.0797061 | 1.77E-20 | 3.06E-19 |
| SH3BP1 | 0.78356261 | 2.29738656 | 1.55187316 | 1.48E-26 | 1.20E-24 |
| CTHRC1 | 2.76964037 | 8.15130262 | 1.55733197 | 1.73E-12 | 7.76E-12 |
| KMO | 4.27225129 | 2.07044315 | -1.0450569 | 2.08E-09 | 6.21E-09 |
| CENPF | 1.39546283 | 3.7542736 | 1.42779009 | 3.55E-19 | 4.90E-18 |
| NPM3 | 10.2772275 | 21.9560063 | 1.09516454 | 3.07E-16 | 2.44E-15 |
| BEX1 | 4.21701928 | 13.9424178 | 1.72518524 | 2.50E-05 | 4.50E-05 |
| LGALS2 | 2.6691563 | 5.74403106 | 1.10567976 | 9.02E-14 | 4.86E-13 |
| SLC12A9-AS1 | 0.34124536 | 0.8409778 | 1.30125828 | 2.82E-10 | 9.26E-10 |
| ANKRD1 | 1.11503007 | 3.82945371 | 1.78005599 | 5.75E-05 | 9.90E-05 |
| CSF2RB | 0.59624128 | 1.28905475 | 1.11234537 | 7.78E-06 | 1.48E-05 |
| CYP2C8 | 145.843512 | 37.112773 | -1.9744335 | 9.29E-24 | 3.36E-22 |
| LAG3 | 1.0863585 | 2.50272095 | 1.20399717 | 5.71E-07 | 1.24E-06 |
| TMSB4XP4 | 0.58459141 | 1.27910647 | 1.12963581 | 1.65E-14 | 9.98E-14 |
| CLCF1 | 1.57488128 | 3.18160152 | 1.01451008 | 1.58E-08 | 4.19E-08 |
| ACADL | 3.01917575 | 1.16444845 | -1.374508 | 2.73E-16 | 2.19E-15 |
| IGKV1D-8 | 0.24589381 | 1.24666416 | 2.34196554 | 0.00045097 | 0.00069734 |
| FCMR | 0.89647212 | 2.1422444 | 1.25679246 | 1.44E-05 | 2.67E-05 |
| AC010761.1 | 0.50464411 | 1.10657487 | 1.13276284 | 2.66E-23 | 8.50E-22 |
| ADCY5 | 0.46947366 | 1.12329923 | 1.25862618 | 1.15E-05 | 2.16E-05 |
| S100A1 | 2.56534575 | 5.27439536 | 1.03985243 | 0.00161136 | 0.00233842 |
| S100A3 | 0.27628851 | 1.22844153 | 2.15258174 | 9.43E-13 | 4.40E-12 |
| CAVIN3 | 3.49967858 | 8.82675745 | 1.33466113 | 1.08E-08 | 2.93E-08 |
| UBE2S | 3.53101589 | 9.78273536 | 1.4701546 | 1.76E-28 | 2.61E-26 |
| IGF2-AS | 0.19397356 | 1.33185257 | 2.77950246 | 0.00215405 | 0.00307562 |
| BHLHE41 | 0.47754246 | 2.07107602 | 2.1166796 | 5.08E-09 | 1.44E-08 |
| SLC13A2 | 0.94582418 | 2.1607397 | 1.19188136 | 0.0017616 | 0.00254283 |
| NREP | 3.01699102 | 6.50266039 | 1.10791968 | 1.24E-19 | 1.84E-18 |
| HAGHL | 0.24414847 | 0.99518136 | 2.02720075 | 6.49E-19 | 8.46E-18 |
| IGF2 | 173.806308 | 683.783149 | 1.97605843 | 0.01822813 | 0.02323345 |
| GFRA3 | 0.27695306 | 1.10665744 | 1.99849532 | 2.25E-13 | 1.15E-12 |
| MISP | 0.7591218 | 6.87361466 | 3.17866569 | 1.85E-20 | 3.18E-19 |
| DCDC2 | 3.17258474 | 15.114435 | 2.25219645 | 2.77E-08 | 7.12E-08 |
| NDRG3 | 5.54691934 | 11.7452896 | 1.08232363 | 5.11E-29 | 8.90E-27 |
| COL11A2 | 0.12283075 | 1.23913953 | 3.33459494 | 7.93E-07 | 1.70E-06 |
| CDC25A | 0.67018926 | 1.74544725 | 1.38095629 | 1.21E-19 | 1.81E-18 |
| PFKFB1 | 10.6017831 | 4.03253346 | -1.3945485 | 4.92E-21 | 9.60E-20 |
| SLC7A10 | 0.52104889 | 1.45396814 | 1.48050501 | 4.12E-14 | 2.35E-13 |
| RGS10 | 3.64734006 | 8.6530849 | 1.24636984 | 1.05E-17 | 1.07E-16 |
| AC008736.1 | 0.75694599 | 1.56674819 | 1.04951106 | 3.72E-07 | 8.28E-07 |
| LPAR1 | 0.34003502 | 0.8106679 | 1.25342771 | 0.00070075 | 0.00105992 |
| AC009686.2 | 0.43290877 | 1.03089546 | 1.25176311 | 9.77E-08 | 2.35E-07 |
| ADAMTS16 | 0.5053584 | 1.08423563 | 1.1012995 | 0.00661631 | 0.00890581 |
| QPCT | 0.94366796 | 2.49291905 | 1.40148481 | 2.77E-12 | 1.21E-11 |
| IGLV3-9 | 0.81468473 | 5.6299321 | 2.78880376 | 0.00736356 | 0.00984858 |
| ZNF506 | 0.47109348 | 1.02076952 | 1.1155719 | 1.39E-12 | 6.33E-12 |
| PAGE1 | 7.49289716 | 15.2069583 | 1.02113606 | 0.0001248 | 0.00020642 |
| TMEM125 | 0.34052462 | 1.63815492 | 2.26624076 | 0.01996837 | 0.02531422 |
| AC040970.1 | 0.35002254 | 1.06015698 | 1.59875815 | 1.30E-18 | 1.61E-17 |
| CPEB3 | 1.68600131 | 0.75056923 | -1.1675486 | 2.30E-19 | 3.29E-18 |
| ORC1 | 0.90891224 | 2.11215555 | 1.21650319 | 1.52E-18 | 1.86E-17 |
| TSPAN10 | 0.45161963 | 1.02606984 | 1.18394884 | 3.71E-08 | 9.35E-08 |
| PCED1B | 1.2826321 | 2.90898476 | 1.18140832 | 2.02E-13 | 1.04E-12 |
| CYP1A2 | 37.7547095 | 6.80304521 | -2.472404 | 7.68E-11 | 2.71E-10 |
| CFHR5 | 74.5454752 | 31.2045288 | -1.2563654 | 1.01E-11 | 4.06E-11 |
| FRAS1 | 0.16121749 | 1.25466415 | 2.96022105 | 1.61E-17 | 1.60E-16 |
| DOK7 | 0.53643626 | 1.36997935 | 1.35267548 | 0.00218605 | 0.00311941 |
| ANLN | 1.32523562 | 3.73726166 | 1.49573268 | 1.46E-17 | 1.46E-16 |
| MAPK13 | 1.21596373 | 5.38695862 | 2.14737079 | 9.33E-22 | 2.15E-20 |
| TUBA3C | 0.98102669 | 5.39942428 | 2.46044129 | 0.02928031 | 0.03626802 |
| SLC38A5 | 0.34973404 | 0.98020885 | 1.48683096 | 2.00E-11 | 7.62E-11 |
| IFITM10 | 1.58080566 | 4.79489133 | 1.6008381 | 3.53E-17 | 3.29E-16 |
| FBXO46 | 3.75260624 | 7.59357782 | 1.01688688 | 7.05E-25 | 3.39E-23 |
| ADAP1 | 0.35625148 | 2.01946277 | 2.50300363 | 8.86E-20 | 1.36E-18 |
| TRBV20-1 | 0.62058475 | 1.46220807 | 1.23644847 | 6.53E-05 | 0.00011182 |
| SPECC1 | 0.40236538 | 1.1960866 | 1.57174376 | 3.31E-08 | 8.39E-08 |
| AL158071.2 | 0.42099935 | 0.89224031 | 1.08361432 | 1.29E-13 | 6.83E-13 |
| GYPC | 3.40437675 | 7.68410502 | 1.17448653 | 9.09E-11 | 3.18E-10 |
| MNDA | 1.01739368 | 2.12286962 | 1.06113773 | 5.27E-07 | 1.15E-06 |
| S100A11 | 51.0523404 | 212.210409 | 2.05544642 | 4.34E-21 | 8.60E-20 |
| HAGLR | 0.64054561 | 2.68092676 | 2.06535859 | 6.41E-08 | 1.57E-07 |
| MFAP2 | 0.45676994 | 2.15715988 | 2.23959348 | 1.41E-14 | 8.66E-14 |
| IGKV4-1 | 13.4366098 | 83.3596157 | 2.63317945 | 0.00014648 | 0.00023988 |
| SLC25A6 | 81.7376528 | 165.227582 | 1.01538182 | 1.67E-25 | 9.60E-24 |
| AOAH | 1.09125502 | 2.32296587 | 1.08997967 | 7.00E-06 | 1.35E-05 |
| SLC7A1 | 0.86198723 | 2.25215177 | 1.38556564 | 9.59E-14 | 5.15E-13 |
| ACSM2B | 40.7955113 | 16.1567611 | -1.3362724 | 1.04E-26 | 8.89E-25 |
| GAS2L3 | 0.56699667 | 1.49322772 | 1.39702202 | 4.59E-22 | 1.14E-20 |
| ITGAE | 0.93606628 | 1.891141 | 1.01457435 | 6.93E-26 | 4.50E-24 |
| ZNF529-AS1 | 0.63495969 | 1.30597428 | 1.04038957 | 2.92E-26 | 2.14E-24 |
| STX1A | 0.40398536 | 1.07832997 | 1.4164238 | 1.30E-23 | 4.45E-22 |
| TINAGL1 | 3.74609645 | 7.93411002 | 1.08268036 | 3.47E-05 | 6.15E-05 |
| CDR2L | 1.01111165 | 3.23538737 | 1.67799614 | 1.05E-08 | 2.87E-08 |
| HEPACAM | 3.39891456 | 0.64848299 | -2.3899334 | 2.42E-05 | 4.36E-05 |
| CPNE7 | 0.55762921 | 1.87416468 | 1.74886969 | 2.70E-09 | 7.93E-09 |
| ADRA2A | 0.51088014 | 1.50835282 | 1.56191716 | 0.00054288 | 0.00083007 |
| AC106900.1 | 0.32027295 | 0.92118009 | 1.52418127 | 2.35E-08 | 6.10E-08 |
| IKZF1 | 0.40140525 | 0.81944927 | 1.02959514 | 1.84E-05 | 3.38E-05 |
| IGHV3-33 | 4.2099118 | 9.52867576 | 1.17848572 | 0.0075174 | 0.0100429 |
| NFE2L3 | 1.0786344 | 3.57778721 | 1.72986163 | 1.38E-20 | 2.46E-19 |
| SLC51A | 20.8630295 | 8.54280596 | -1.2881667 | 1.33E-12 | 6.08E-12 |
| SNHG14 | 0.35222052 | 1.02338415 | 1.53879691 | 1.46E-11 | 5.69E-11 |
| ADH4 | 310.406555 | 110.378424 | -1.4917008 | 1.37E-12 | 6.25E-12 |
| FUT3 | 0.13004852 | 1.47102859 | 3.49970343 | 7.43E-08 | 1.81E-07 |
| CEP55 | 0.6259817 | 2.72908967 | 2.12422742 | 9.45E-29 | 1.53E-26 |
| IGLV2-8 | 1.47355152 | 6.84617131 | 2.21599989 | 0.00756402 | 0.01009945 |
| TMEM201 | 1.14617674 | 2.35103381 | 1.03646575 | 4.78E-27 | 4.40E-25 |
| EPO | 1.56525898 | 4.58026779 | 1.54903057 | 4.38E-09 | 1.25E-08 |
| ZNF160 | 0.67343934 | 1.36053811 | 1.01455746 | 3.69E-11 | 1.36E-10 |
| IGHV4-31 | 3.21625038 | 10.6543557 | 1.72799172 | 0.00631324 | 0.0085255 |
| SPINT1 | 3.06177016 | 23.6570289 | 2.949831 | 1.65E-15 | 1.16E-14 |
| CYP2E1 | 334.308336 | 163.623456 | -1.0307998 | 0.00054495 | 0.00083293 |
| FDCSP | 1.82316356 | 26.4407087 | 3.85824494 | 3.28E-10 | 1.07E-09 |
| GTSE1 | 0.76672095 | 2.40200126 | 1.64746339 | 2.66E-24 | 1.08E-22 |
| FNDC10 | 1.0620454 | 2.37027986 | 1.15821197 | 1.60E-13 | 8.34E-13 |
| AC008537.3 | 2.72209322 | 0.91559882 | -1.571929 | 3.11E-10 | 1.02E-09 |
| AQP9 | 178.67354 | 62.743492 | -1.5097883 | 7.09E-21 | 1.34E-19 |
| IGKV2D-29 | 1.12348788 | 2.34063289 | 1.05891412 | 0.00284027 | 0.00399315 |
| SMG9 | 1.97532003 | 4.31403251 | 1.12695064 | 7.70E-35 | 9.05E-32 |
| GPC4 | 0.95999561 | 3.16367565 | 1.72050198 | 1.47E-08 | 3.91E-08 |
| RARG | 0.88397166 | 1.85140596 | 1.06654926 | 3.19E-09 | 9.28E-09 |
| DQX1 | 0.2141968 | 1.75271961 | 3.0325864 | 4.29E-22 | 1.07E-20 |
| SSTR2 | 0.49427101 | 1.24252295 | 1.32989832 | 1.44E-07 | 3.38E-07 |
| ANGPTL3 | 119.255032 | 58.0341051 | -1.0390773 | 1.01E-21 | 2.31E-20 |
| AASS | 5.65847929 | 1.84108013 | -1.619862 | 4.61E-18 | 5.05E-17 |
| ELFN1-AS1 | 0.38235835 | 1.74576665 | 2.19086344 | 9.83E-05 | 0.00016447 |
| MIR23AHG | 1.27959652 | 3.11409396 | 1.28312351 | 2.11E-12 | 9.31E-12 |
| AC006042.1 | 0.45867611 | 0.92722028 | 1.01543637 | 6.14E-06 | 1.19E-05 |
| LRRC37A7P | 1.52211796 | 0.71821045 | -1.0836016 | 1.40E-08 | 3.74E-08 |
| RPL39L | 3.93030856 | 10.3072161 | 1.39094024 | 2.37E-10 | 7.85E-10 |
| PLEKHA2 | 1.30020399 | 2.80713986 | 1.11036296 | 2.00E-16 | 1.63E-15 |
| VAV1 | 0.7617052 | 1.79311264 | 1.23516146 | 3.54E-09 | 1.02E-08 |
| IGHV6-1 | 0.22538006 | 0.92784761 | 2.04152796 | 0.00358418 | 0.00497109 |
| TNNC1 | 1.2158397 | 4.61468344 | 1.92427865 | 8.18E-07 | 1.75E-06 |
| GLDN | 0.54519133 | 1.09509787 | 1.0062253 | 6.98E-12 | 2.86E-11 |
| C2CD4B | 0.33851671 | 0.82610282 | 1.28709433 | 0.00011617 | 0.00019275 |
| LINC01589 | 0.36900556 | 0.80637335 | 1.12780541 | 1.39E-13 | 7.30E-13 |
| LINC00665 | 0.74728832 | 2.29511285 | 1.61882821 | 2.75E-12 | 1.20E-11 |
| AC012379.1 | 1.12936861 | 0.25587963 | -2.1419793 | 1.04E-05 | 1.96E-05 |
| ITGA2 | 0.89022094 | 1.98795453 | 1.15904942 | 4.85E-07 | 1.07E-06 |
| CPA3 | 0.77546789 | 1.73717483 | 1.163604 | 0.01217578 | 0.01584436 |
| APOBEC3C | 2.15413014 | 5.38294865 | 1.32129125 | 8.19E-10 | 2.57E-09 |
| UNC13D | 0.59948513 | 2.63967503 | 2.13856445 | 3.78E-17 | 3.50E-16 |
| RBP4 | 3958.75126 | 1898.11019 | -1.0604817 | 4.18E-26 | 2.86E-24 |
| NFKBIE | 6.11299134 | 12.3773143 | 1.01774788 | 4.92E-21 | 9.60E-20 |
| IGHG1 | 99.5022393 | 407.137193 | 2.03271412 | 1.87E-05 | 3.42E-05 |
| LAMC2 | 0.42578839 | 3.43061774 | 3.01025985 | 2.11E-07 | 4.86E-07 |
| WFDC2 | 2.59489573 | 10.6944515 | 2.04311402 | 1.05E-08 | 2.86E-08 |
| IL18 | 1.21994842 | 3.85611421 | 1.66032763 | 1.06E-08 | 2.88E-08 |
| MSC | 5.64972094 | 14.470561 | 1.35686934 | 1.54E-07 | 3.60E-07 |
| SYDE1 | 1.39356677 | 2.95342161 | 1.08360519 | 1.55E-12 | 7.01E-12 |
| FGFR2 | 6.01792685 | 12.721832 | 1.07996797 | 4.61E-06 | 9.07E-06 |
| FCRLA | 0.2076333 | 1.19928068 | 2.5300596 | 5.77E-06 | 1.12E-05 |
| RND2 | 1.20312141 | 3.87902477 | 1.68891175 | 1.66E-06 | 3.42E-06 |
| HOTAIRM1 | 0.4499484 | 0.96583258 | 1.10201357 | 2.24E-09 | 6.67E-09 |
| TTPA | 27.1969422 | 12.3337216 | -1.1408363 | 4.91E-18 | 5.35E-17 |
| LAYN | 0.45727565 | 0.94788258 | 1.05164426 | 2.11E-07 | 4.86E-07 |
| TNFAIP8L2 | 1.7453139 | 3.68497167 | 1.078167 | 1.78E-12 | 7.97E-12 |
| FBXL19 | 1.48816607 | 3.11180378 | 1.06421556 | 8.25E-25 | 3.89E-23 |
| SLAMF8 | 1.61511652 | 3.93297405 | 1.28398241 | 2.98E-09 | 8.71E-09 |
| RAB38 | 0.35534123 | 0.89739719 | 1.33654157 | 2.30E-10 | 7.66E-10 |
| PTP4A3 | 7.11518335 | 15.416966 | 1.11554604 | 4.94E-14 | 2.79E-13 |
| LIF | 0.85683496 | 3.39766179 | 1.987453 | 9.03E-11 | 3.16E-10 |
| MTMR2 | 1.24498142 | 2.84922631 | 1.19444601 | 2.20E-25 | 1.22E-23 |
| OSBP2 | 0.47373173 | 1.16675203 | 1.30035575 | 2.83E-16 | 2.26E-15 |
| GPX7 | 2.98692819 | 6.39622975 | 1.09855921 | 7.03E-10 | 2.22E-09 |
| C6orf132 | 0.16206519 | 1.50327972 | 3.21346729 | 8.06E-17 | 7.03E-16 |
| ATP1A1 | 51.210746 | 107.593589 | 1.07107363 | 4.81E-13 | 2.34E-12 |
| KRT17 | 0.86013749 | 3.85706766 | 2.16486526 | 7.03E-12 | 2.88E-11 |
| CLEC11A | 2.91875428 | 7.96204158 | 1.44778565 | 1.06E-08 | 2.88E-08 |
| HS1BP3-IT1 | 7.02060842 | 3.33919943 | -1.0720938 | 8.40E-08 | 2.03E-07 |
| AL109615.3 | 0.33969846 | 1.15499244 | 1.76555681 | 5.92E-20 | 9.36E-19 |
| SAMSN1 | 0.74605084 | 1.53439608 | 1.04032508 | 1.14E-05 | 2.14E-05 |
| LAPTM4B | 27.3010292 | 59.4362661 | 1.12238815 | 6.05E-13 | 2.90E-12 |
| FERMT3 | 3.39580105 | 7.29229533 | 1.10262105 | 3.02E-13 | 1.51E-12 |
| DHDH | 0.29020269 | 0.88261285 | 1.60471987 | 1.11E-12 | 5.13E-12 |
| AC007773.1 | 0.4326054 | 1.12579697 | 1.37982309 | 1.34E-14 | 8.24E-14 |
| HJURP | 1.41033717 | 3.8007598 | 1.43024775 | 1.43E-23 | 4.80E-22 |
| AC023090.1 | 0.19419571 | 1.26168345 | 2.69976673 | 6.30E-19 | 8.27E-18 |
| RUNDC3B | 1.92995465 | 0.86105992 | -1.1643814 | 2.43E-21 | 5.09E-20 |
| TIGD1 | 0.80312253 | 1.93247365 | 1.26675672 | 3.78E-25 | 1.95E-23 |
| AFAP1L2 | 0.40697721 | 0.88908929 | 1.12738029 | 0.00048113 | 0.00074045 |
| SAMD11 | 0.46900528 | 0.98171863 | 1.06570543 | 2.43E-05 | 4.38E-05 |
| FNDC5 | 15.0235618 | 2.5378134 | -2.565569 | 1.33E-13 | 7.02E-13 |
| SIT1 | 0.68478667 | 1.66058803 | 1.27796768 | 7.07E-07 | 1.52E-06 |
| CHST3 | 0.51216766 | 1.75900939 | 1.78007511 | 2.03E-07 | 4.70E-07 |
| CRABP2 | 0.71479921 | 3.11727589 | 2.1246759 | 2.65E-11 | 9.95E-11 |
| BARD1 | 0.48073277 | 1.02821536 | 1.0968354 | 1.37E-16 | 1.15E-15 |
| PAK4 | 4.39752232 | 8.85978918 | 1.01058147 | 2.02E-27 | 2.16E-25 |
| SLC1A2 | 7.38230794 | 2.43879475 | -1.5979036 | 3.23E-12 | 1.39E-11 |
| SLC44A4 | 0.40238983 | 1.76931926 | 2.13652866 | 2.81E-07 | 6.35E-07 |
| TTR | 1165.78064 | 569.475182 | -1.0335915 | 7.44E-14 | 4.08E-13 |
| MYOF | 1.26943904 | 3.14546732 | 1.30908325 | 0.00095759 | 0.00142592 |
| HILPDA | 1.84360336 | 5.27521523 | 1.51670166 | 4.35E-20 | 7.04E-19 |
| CENPI | 0.34880098 | 0.87145176 | 1.32101673 | 1.50E-17 | 1.50E-16 |
| PLXDC1 | 0.52812049 | 1.40501659 | 1.41164816 | 1.12E-13 | 5.96E-13 |
| IGHV1-58 | 1.49772789 | 3.56043301 | 1.24927717 | 0.00328665 | 0.0045787 |
| AR | 8.08195155 | 3.3122847 | -1.286877 | 3.74E-20 | 6.12E-19 |
| PLA2G2D | 0.47788281 | 2.19007575 | 2.19625198 | 4.97E-06 | 9.73E-06 |
| RANGRF | 3.12475135 | 6.31761784 | 1.01563928 | 6.33E-20 | 9.97E-19 |
| LILRB1 | 0.43993613 | 0.94253059 | 1.09924536 | 5.31E-06 | 1.04E-05 |
| TPPP3 | 1.49131339 | 4.19831809 | 1.49322801 | 2.79E-09 | 8.17E-09 |
| PTGS1 | 0.63467533 | 1.39168518 | 1.13274221 | 2.90E-12 | 1.26E-11 |
| MEIS2 | 1.06861349 | 2.2831878 | 1.0953094 | 6.75E-07 | 1.46E-06 |
| NTS | 31.8831811 | 85.5745147 | 1.42438563 | 8.69E-06 | 1.65E-05 |
| SLCO4C1 | 0.58251932 | 1.29941142 | 1.15748048 | 1.22E-08 | 3.29E-08 |
| MYCL | 5.23632561 | 2.50157443 | -1.0657184 | 3.13E-09 | 9.12E-09 |
| PARM1 | 1.03976056 | 2.32930004 | 1.16364515 | 0.00052088 | 0.00079822 |
| PPP1R14D | 0.51152255 | 2.3519207 | 2.20096968 | 1.10E-13 | 5.90E-13 |
| MIR3685 | 2.06055088 | 4.88182924 | 1.24439174 | 2.78E-15 | 1.89E-14 |
| PAFAH1B3 | 6.48291958 | 25.0037121 | 1.94742671 | 2.80E-32 | 1.58E-29 |
| NKG7 | 6.18073904 | 13.0442387 | 1.07756149 | 0.02053669 | 0.02598328 |
| PRSS22 | 0.09407257 | 2.45762253 | 4.70734536 | 5.71E-13 | 2.75E-12 |
| AL161668.4 | 3.56759583 | 1.02904523 | -1.7936458 | 4.40E-16 | 3.41E-15 |
| ZC2HC1A | 0.45398326 | 1.01073877 | 1.15469917 | 4.98E-14 | 2.81E-13 |
| TFR2 | 171.878937 | 82.807535 | -1.0535588 | 4.81E-26 | 3.21E-24 |
| MAPK12 | 0.85761634 | 1.74439645 | 1.02432367 | 1.02E-07 | 2.45E-07 |
| BLM | 0.35778259 | 0.86395409 | 1.27187148 | 3.69E-23 | 1.15E-21 |
| MIR621 | 111.542089 | 52.7599225 | -1.0800738 | 1.21E-12 | 5.55E-12 |
| MCUB | 0.79263721 | 1.94495682 | 1.29500552 | 9.52E-14 | 5.12E-13 |
| RERG | 0.47365936 | 1.22575137 | 1.37174458 | 0.00043417 | 0.00067288 |
| C11orf49 | 1.00567454 | 2.19857959 | 1.12840827 | 1.53E-18 | 1.88E-17 |
| RHPN1 | 1.22772277 | 2.89252635 | 1.23634528 | 3.07E-11 | 1.14E-10 |
| CRISP3 | 0.93098194 | 2.03633421 | 1.12914927 | 0.00049984 | 0.00076807 |
| AL137798.1 | 2.50787502 | 1.0878158 | -1.2050312 | 4.14E-05 | 7.27E-05 |
| ACTL8 | 0.47237662 | 2.45107618 | 2.37540587 | 7.83E-05 | 0.00013292 |
| FXYD2 | 1.55867363 | 8.90737335 | 2.51468119 | 1.03E-18 | 1.30E-17 |
| P2RY6 | 0.38391882 | 1.13065388 | 1.55828415 | 1.09E-11 | 4.33E-11 |
| IGLC3 | 31.1542333 | 92.0642026 | 1.56321209 | 0.00034404 | 0.000539 |
| MELTF-AS1 | 0.50587879 | 1.62847654 | 1.68665927 | 9.46E-25 | 4.39E-23 |
| NBEAL2 | 1.40759537 | 3.19577356 | 1.18293251 | 1.34E-21 | 2.98E-20 |
| LINC02041 | 0.56011284 | 1.93705909 | 1.79007855 | 6.55E-12 | 2.69E-11 |
| ITPR3 | 0.65882717 | 2.97682624 | 2.17580306 | 7.08E-12 | 2.89E-11 |
| SLC10A3 | 3.97906459 | 9.47711708 | 1.25201894 | 2.69E-29 | 5.67E-27 |
| C1orf116 | 0.29273028 | 2.6430695 | 3.17457048 | 1.44E-15 | 1.02E-14 |
| TNFRSF10C | 0.57997796 | 1.30433907 | 1.16924897 | 4.78E-10 | 1.53E-09 |
| PCDHB16 | 0.35757315 | 0.73427927 | 1.03809046 | 3.58E-05 | 6.33E-05 |
| DTL | 1.78358897 | 3.61248772 | 1.0182095 | 1.20E-14 | 7.44E-14 |
| CD48 | 1.49608433 | 3.25811438 | 1.12284576 | 6.19E-06 | 1.20E-05 |
| ETV4 | 3.92675272 | 10.0475063 | 1.35542883 | 1.29E-13 | 6.83E-13 |
| AC099508.2 | 0.76114028 | 0.22883485 | -1.7338556 | 3.01E-12 | 1.30E-11 |
| AC016735.1 | 0.80537238 | 4.31924053 | 2.42304976 | 2.51E-13 | 1.27E-12 |
| CENPW | 4.89114964 | 13.151499 | 1.42698173 | 1.62E-25 | 9.41E-24 |
| PHETA2 | 1.10418675 | 2.77386841 | 1.32891516 | 1.25E-07 | 2.96E-07 |
| MTHFD1L | 1.68768788 | 3.69620274 | 1.13099577 | 3.01E-24 | 1.21E-22 |
| DAPL1 | 0.01771965 | 1.45664446 | 6.36115455 | 3.99E-05 | 7.01E-05 |
| RAP1GAP2 | 0.37255388 | 1.26017446 | 1.7581025 | 6.49E-11 | 2.30E-10 |
| NDN | 2.533677 | 7.36735851 | 1.53991483 | 1.98E-07 | 4.58E-07 |
| MTATP8P1 | 5.12303138 | 1.12326254 | -2.1893026 | 0.00112964 | 0.00166855 |
| CSF3R | 0.44806412 | 1.37263861 | 1.61517474 | 4.41E-11 | 1.60E-10 |
| KIF12 | 5.06176893 | 17.175997 | 1.76268029 | 2.38E-18 | 2.79E-17 |
| TMEM86A | 1.09669047 | 2.2567325 | 1.04107902 | 2.18E-08 | 5.68E-08 |
| DES | 0.3675776 | 2.86566164 | 2.96274751 | 6.48E-06 | 1.25E-05 |
| VSIG4 | 2.81863451 | 5.70335723 | 1.01681498 | 0.0065196 | 0.00878566 |
| S100B | 0.33194403 | 1.00043146 | 1.59161041 | 7.63E-11 | 2.69E-10 |
| NCAPD2 | 3.23095571 | 7.72106231 | 1.25683838 | 2.07E-23 | 6.74E-22 |
| FUT1 | 0.54047838 | 1.24219457 | 1.20058234 | 4.59E-11 | 1.66E-10 |
| UGT1A1 | 21.0777247 | 8.55595763 | -1.3007179 | 3.03E-12 | 1.31E-11 |
| KIF20A | 1.76904879 | 4.70787689 | 1.41210276 | 2.44E-19 | 3.45E-18 |
| LINGO1 | 1.03802631 | 2.79334598 | 1.42815126 | 2.37E-13 | 1.20E-12 |
| IQCD | 0.43257798 | 0.89294419 | 1.04560979 | 4.23E-11 | 1.55E-10 |
| PTDSS2 | 2.95032511 | 5.91373786 | 1.00319636 | 1.57E-29 | 3.63E-27 |
| ABCA6 | 6.57775975 | 2.56725032 | -1.3573723 | 5.77E-25 | 2.86E-23 |
| RAVER1 | 1.04357837 | 2.24610165 | 1.10588428 | 8.15E-09 | 2.25E-08 |
| HNRNPA1P16 | 0.38419482 | 0.98233143 | 1.3543718 | 9.61E-22 | 2.21E-20 |
| SIGLEC10 | 0.46468417 | 1.17132433 | 1.33381818 | 1.18E-10 | 4.06E-10 |
| COL1A2 | 17.4697793 | 39.5654163 | 1.17937856 | 1.55E-06 | 3.21E-06 |
| ATP1B3 | 7.97731586 | 18.1995245 | 1.18992545 | 1.26E-19 | 1.88E-18 |
| CES2 | 137.330272 | 53.6108974 | -1.3570515 | 3.32E-27 | 3.25E-25 |
| GCGR | 13.6947335 | 5.51301818 | -1.3127069 | 3.01E-08 | 7.69E-08 |
| IGLV9-49 | 0.73365653 | 2.40678064 | 1.71392794 | 0.0021907 | 0.00312574 |
| FOXS1 | 1.17198128 | 3.4455902 | 1.5558016 | 1.40E-11 | 5.48E-11 |
| CD37 | 1.408385 | 3.37573926 | 1.26116171 | 1.42E-11 | 5.55E-11 |
| COL10A1 | 0.23319374 | 0.93101301 | 1.99727227 | 0.00018991 | 0.00030704 |
| CEACAM5 | 0.33462149 | 1.25469334 | 1.90673281 | 7.46E-05 | 0.00012684 |
| CES4A | 1.79913891 | 0.6732416 | -1.4181103 | 3.26E-13 | 1.62E-12 |
| MME | 7.76201383 | 3.66348354 | -1.0832149 | 0.00028365 | 0.00044936 |
| ZNF300 | 0.46547077 | 0.9614994 | 1.04659539 | 2.12E-09 | 6.32E-09 |
| TYROBP | 25.4533627 | 53.1942928 | 1.0634152 | 1.21E-12 | 5.57E-12 |
| AGRN | 12.0618021 | 24.4910681 | 1.02181022 | 1.82E-08 | 4.79E-08 |
| B3GALT4 | 0.99470812 | 2.17242403 | 1.12696056 | 8.88E-14 | 4.79E-13 |
| SCRN1 | 1.18559706 | 3.86613719 | 1.70527906 | 2.25E-08 | 5.87E-08 |
| DLK2 | 0.51996625 | 1.26156977 | 1.27873012 | 3.21E-12 | 1.38E-11 |
| AFAP1-AS1 | 0.77639277 | 2.22222212 | 1.51714444 | 0.0005014 | 0.00077038 |
| CHD3 | 2.07150514 | 5.41403918 | 1.38602593 | 6.79E-19 | 8.81E-18 |
| S100A9 | 15.2774583 | 97.9551119 | 2.68071624 | 1.34E-08 | 3.60E-08 |
| PELATON | 0.35256336 | 1.05420721 | 1.58020401 | 1.41E-09 | 4.31E-09 |
| HSPB8 | 4.94423668 | 10.3004907 | 1.05889336 | 1.37E-06 | 2.86E-06 |
| AP003119.3 | 3.74161863 | 1.8396579 | -1.024225 | 3.59E-11 | 1.32E-10 |
| PPM1H | 1.08224806 | 2.76044207 | 1.35086811 | 4.09E-10 | 1.32E-09 |
| NUF2 | 1.3159128 | 3.5229442 | 1.42071773 | 2.60E-21 | 5.43E-20 |
| SAA2 | 218.577624 | 48.4487499 | -2.1736144 | 0.00042272 | 0.00065592 |
| LDHD | 41.0845927 | 20.1183829 | -1.0300831 | 1.10E-21 | 2.51E-20 |
| CD8B | 0.59881495 | 1.55919143 | 1.38061593 | 1.74E-05 | 3.19E-05 |
| LRRN2 | 0.52953252 | 1.20096617 | 1.18140433 | 1.87E-06 | 3.84E-06 |
| JAG1 | 2.88871114 | 7.4485268 | 1.36652916 | 1.54E-07 | 3.60E-07 |
| EVA1B | 3.76415345 | 8.52153061 | 1.17878714 | 9.33E-23 | 2.73E-21 |
| CYP2A13 | 3.51104081 | 0.65948112 | -2.4124955 | 5.54E-08 | 1.37E-07 |
| CPS1 | 172.765491 | 82.0002031 | -1.0751157 | 2.35E-12 | 1.03E-11 |
| BOK-AS1 | 0.76105145 | 0.28764799 | -1.4036896 | 9.95E-08 | 2.39E-07 |
| TRAC | 4.19531715 | 9.90780651 | 1.23978582 | 1.48E-07 | 3.47E-07 |
| S100A14 | 10.6223705 | 46.3731957 | 2.12618539 | 4.08E-09 | 1.17E-08 |
| ECT2 | 1.73590973 | 4.17372051 | 1.26564207 | 6.62E-17 | 5.86E-16 |
| IGKV6-21 | 1.2347163 | 2.49986342 | 1.01766968 | 6.71E-05 | 0.00011478 |
| DAB2 | 4.25253623 | 9.42644539 | 1.14839032 | 3.31E-09 | 9.60E-09 |
| P3H2 | 0.4418133 | 1.33791116 | 1.59847357 | 0.01473107 | 0.01896128 |
| SLC27A2 | 63.641682 | 27.7644747 | -1.1967319 | 6.26E-21 | 1.19E-19 |
| LINC00844 | 32.1164663 | 6.10793461 | -2.3945566 | 3.79E-15 | 2.52E-14 |
| SLC25A15 | 23.4826128 | 10.865154 | -1.1118843 | 8.16E-14 | 4.43E-13 |
| LINC00205 | 0.8757796 | 2.12090731 | 1.27604182 | 5.58E-23 | 1.70E-21 |
| NCS1 | 1.09268425 | 3.25943975 | 1.57674744 | 9.72E-17 | 8.39E-16 |
| BBOX1 | 7.09279709 | 3.3956734 | -1.062657 | 0.00015705 | 0.00025627 |
| NCF2 | 2.89401252 | 5.82350133 | 1.00881566 | 4.72E-12 | 1.99E-11 |
| TRIM45 | 0.69629959 | 1.64287081 | 1.23843895 | 4.02E-13 | 1.97E-12 |
| IGHG3 | 18.6515218 | 59.7851358 | 1.68049349 | 5.00E-06 | 9.78E-06 |
| PLAC9 | 1.93651973 | 7.05045501 | 1.86425016 | 0.00015514 | 0.00025337 |
| PFN1P11 | 5.82281595 | 2.38102734 | -1.2901328 | 4.14E-05 | 7.26E-05 |
| MICALL2 | 1.67507403 | 3.66467536 | 1.12946055 | 1.89E-14 | 1.13E-13 |
| PTK7 | 0.91518478 | 3.1628774 | 1.78910267 | 6.79E-12 | 2.78E-11 |
| SLBP-DT | 0.32621081 | 0.87937717 | 1.43067748 | 2.52E-27 | 2.61E-25 |
| KCNH2 | 0.14402802 | 1.31037415 | 3.18555742 | 4.72E-14 | 2.67E-13 |
| TCN1 | 0.19316538 | 0.97858168 | 2.34085571 | 1.41E-11 | 5.51E-11 |
| CMTM7 | 1.6576934 | 3.93038305 | 1.24549273 | 4.91E-26 | 3.27E-24 |
| TTLL4 | 1.89507274 | 5.312675 | 1.48718523 | 1.59E-26 | 1.27E-24 |
| FBLN1 | 3.51135884 | 13.6783709 | 1.96179507 | 1.92E-13 | 9.90E-13 |
| PITX1 | 0.74487382 | 3.12352135 | 2.06810542 | 5.03E-17 | 4.56E-16 |
| FAM24B | 0.27592643 | 0.91731837 | 1.7331389 | 9.77E-15 | 6.10E-14 |
| RMI2 | 1.96714763 | 3.95098308 | 1.00610643 | 6.85E-16 | 5.15E-15 |
| HLA-DQB2 | 1.96799646 | 5.91120039 | 1.58672351 | 1.25E-10 | 4.29E-10 |
| MFSD10 | 4.43509868 | 15.9864526 | 1.84981173 | 1.98E-38 | 1.16E-34 |
| LOXL4 | 5.16861032 | 13.5848399 | 1.39414922 | 1.88E-08 | 4.95E-08 |
| PLA2G1B | 1.8865293 | 4.1186797 | 1.12644743 | 0.00139167 | 0.00203363 |
| REEP4 | 4.66583465 | 9.95104525 | 1.09271289 | 5.49E-22 | 1.34E-20 |
| FAM83A-AS1 | 10.5518777 | 1.50252441 | -2.8120394 | 8.04E-11 | 2.83E-10 |
| AC092535.5 | 4.1421791 | 9.68617601 | 1.22553729 | 1.77E-13 | 9.15E-13 |
| GDF11 | 0.39303008 | 0.93583172 | 1.25160941 | 7.32E-16 | 5.48E-15 |
| AP005262.1 | 0.52896868 | 1.18813567 | 1.16744538 | 0.00028378 | 0.00044949 |
| NIBAN1 | 0.85423383 | 1.74439668 | 1.03002521 | 0.00039455 | 0.00061458 |
| IKZF3 | 0.44866815 | 0.96407577 | 1.10349777 | 0.00266479 | 0.00375882 |
| PMAIP1 | 0.23921315 | 0.94743459 | 1.98572966 | 7.05E-14 | 3.88E-13 |
| SMIM32 | 0.76874941 | 7.27589904 | 3.24254023 | 1.93E-07 | 4.48E-07 |
| SOX9 | 5.53896085 | 16.4218173 | 1.56792655 | 4.18E-15 | 2.76E-14 |
| GTSF1 | 1.6510619 | 4.06585491 | 1.30016452 | 0.00411774 | 0.00567094 |
| APOL4 | 0.60956816 | 1.51740118 | 1.31574311 | 4.27E-07 | 9.44E-07 |
| PTAFR | 0.84504665 | 2.33273627 | 1.46492032 | 3.98E-11 | 1.46E-10 |
| MAP7D2 | 0.40736677 | 1.74595553 | 2.0996166 | 1.91E-12 | 8.52E-12 |
| HOMER3 | 1.83961209 | 7.33012137 | 1.9944355 | 6.00E-31 | 2.56E-28 |
| CYP1A1 | 24.2064594 | 10.4321568 | -1.2143546 | 4.03E-09 | 1.16E-08 |
| MAGEA8 | 0.1824728 | 1.03320003 | 2.50136626 | 0.02529291 | 0.03161782 |
| NR1I3 | 23.274632 | 9.65960109 | -1.2687228 | 4.85E-17 | 4.41E-16 |
| TMC4 | 1.95326184 | 14.3810699 | 2.88021374 | 2.80E-09 | 8.22E-09 |
| ZNF204P | 0.47614977 | 1.27610378 | 1.42225831 | 3.98E-09 | 1.14E-08 |
| PRKAR1B-AS2 | 0.19526832 | 2.20524507 | 3.49740919 | 2.33E-05 | 4.20E-05 |
| IGLV1-47 | 10.526503 | 29.342116 | 1.47894668 | 0.00039839 | 0.0006203 |
| AC254562.2 | 0.89932248 | 0.44404016 | -1.0181484 | 7.98E-06 | 1.52E-05 |
| TPSAB1 | 0.93718908 | 3.2818201 | 1.80808412 | 0.0016153 | 0.0023439 |
| LINC02428 | 5.44463407 | 2.07745048 | -1.390021 | 6.88E-09 | 1.92E-08 |
| RNU6-1189P | 2.66321871 | 1.28402977 | -1.0524923 | 4.06E-07 | 9.00E-07 |
| CFHR4 | 18.9775904 | 4.86410088 | -1.9640518 | 3.44E-25 | 1.79E-23 |
| C12orf75 | 2.55605526 | 11.3031531 | 2.14473435 | 1.43E-11 | 5.59E-11 |
| CYCSP34 | 0.37282138 | 0.77251498 | 1.0510783 | 7.03E-09 | 1.95E-08 |
| MMP7 | 3.58375651 | 14.4010463 | 2.00662911 | 5.84E-11 | 2.09E-10 |
| IGSF23 | 16.2517896 | 6.63650707 | -1.2921026 | 7.88E-16 | 5.87E-15 |
| G6PC3 | 3.66044598 | 8.1958879 | 1.16288082 | 2.69E-29 | 5.67E-27 |
| PAPPA2 | 0.97512793 | 0.42623165 | -1.1939538 | 3.06E-08 | 7.81E-08 |
| EGLN3 | 0.93063673 | 2.5462481 | 1.45208296 | 1.71E-17 | 1.69E-16 |
| LIMCH1 | 0.87446952 | 2.07160265 | 1.2442673 | 0.0075667 | 0.01010207 |
| IGHV1-69 | 1.59995993 | 7.11206911 | 2.15223357 | 0.00032193 | 0.00050587 |
| NXPH4 | 1.35880951 | 6.01045469 | 2.14513092 | 2.04E-13 | 1.05E-12 |
| FOXO6 | 0.31017222 | 0.91486952 | 1.56049651 | 6.58E-12 | 2.71E-11 |
| HOXB7 | 0.49927028 | 2.22030079 | 2.1528622 | 1.45E-08 | 3.87E-08 |
| FABP3 | 3.2026197 | 13.658319 | 2.09245553 | 1.89E-07 | 4.39E-07 |
| SPRING1 | 1.62623363 | 4.50944766 | 1.4714162 | 2.54E-29 | 5.60E-27 |
| KRT23 | 8.42965421 | 22.4173782 | 1.4110722 | 1.46E-05 | 2.70E-05 |
| ITGB1-DT | 0.87765422 | 2.63381172 | 1.58542765 | 1.60E-06 | 3.30E-06 |
| TNIK | 0.61259429 | 1.40522388 | 1.19779617 | 4.47E-09 | 1.28E-08 |
| IGLV7-46 | 0.90807763 | 8.23694817 | 3.18122236 | 0.00013975 | 0.00022942 |
| GGT6 | 0.4149227 | 1.13683667 | 1.45411052 | 5.44E-06 | 1.06E-05 |
| AC005224.3 | 0.41615395 | 1.27826425 | 1.61899689 | 0.00013337 | 0.00021959 |
| MARVELD1 | 1.70166153 | 4.02003134 | 1.24026265 | 1.18E-10 | 4.06E-10 |
| SLC6A19 | 0.43210648 | 1.24994035 | 1.53240046 | 7.90E-10 | 2.48E-09 |
| TGFB1 | 7.26426804 | 20.8246471 | 1.5194027 | 3.65E-14 | 2.10E-13 |
| SOGA1 | 0.88665801 | 1.85379374 | 1.06403108 | 2.23E-17 | 2.15E-16 |
| LTB | 3.78626536 | 15.8721249 | 2.06764786 | 2.88E-15 | 1.95E-14 |
| MMP2 | 5.58805033 | 14.4738818 | 1.37303498 | 2.21E-05 | 4.00E-05 |
| DNER | 0.36163224 | 2.41249348 | 2.73792983 | 1.68E-08 | 4.44E-08 |
| NDUFA4L2 | 7.11479377 | 16.0801043 | 1.17638292 | 8.29E-14 | 4.50E-13 |
| FOSL1 | 0.57343669 | 1.50680255 | 1.39378427 | 0.00061429 | 0.00093506 |
| ZNF880 | 0.34107244 | 1.25483004 | 1.87934189 | 3.86E-15 | 2.56E-14 |
| GDPD3 | 0.4540117 | 1.62127781 | 1.83632994 | 5.50E-24 | 2.08E-22 |
| TKTL1 | 0.16269642 | 2.23592166 | 3.78061524 | 1.00E-06 | 2.13E-06 |
| EPS8L1 | 0.22927804 | 2.08038988 | 3.18168386 | 1.69E-18 | 2.05E-17 |
| GBA3 | 12.8113447 | 3.86545817 | -1.7287106 | 7.65E-17 | 6.72E-16 |
| CES5A | 0.83035307 | 0.38704513 | -1.1012231 | 1.88E-10 | 6.31E-10 |
| DEPDC1B | 0.81072134 | 2.98398654 | 1.879963 | 2.55E-25 | 1.38E-23 |
| AC021074.3 | 12.8551452 | 5.93622648 | -1.1147279 | 3.18E-12 | 1.37E-11 |
| CDCP1 | 0.23366816 | 1.82599339 | 2.96614847 | 2.33E-19 | 3.32E-18 |
| PRMT2 | 2.36211582 | 4.89246761 | 1.05048259 | 2.08E-30 | 6.99E-28 |
| SYTL1 | 0.38592877 | 0.960879 | 1.31602018 | 4.50E-14 | 2.55E-13 |
| MDFI | 0.56331954 | 3.14628136 | 2.48162228 | 3.02E-14 | 1.75E-13 |
| KLF5 | 1.94031638 | 4.32348269 | 1.155902 | 3.76E-07 | 8.35E-07 |
| VLDLR | 0.69594167 | 1.49228654 | 1.10048627 | 0.00156993 | 0.0022811 |
| SH2D1A | 0.40039546 | 0.85171127 | 1.08893883 | 0.00871023 | 0.01153698 |
| SLC52A3 | 0.38093412 | 1.27705735 | 1.74520987 | 0.00031895 | 0.00050126 |
| PTHLH | 0.29430457 | 2.59830822 | 3.1421907 | 1.41E-14 | 8.63E-14 |
| AC006504.7 | 0.46903534 | 1.11806491 | 1.25323543 | 5.82E-20 | 9.23E-19 |
| MPZL1 | 7.20240508 | 14.4810858 | 1.00761913 | 1.49E-26 | 1.20E-24 |
| GFPT2 | 0.3517743 | 0.87420119 | 1.31331524 | 2.18E-07 | 5.01E-07 |
| UNC5B | 1.62669846 | 3.42080519 | 1.0723891 | 1.14E-09 | 3.52E-09 |
| PLBD1 | 1.52079871 | 6.33192316 | 2.05781453 | 6.66E-13 | 3.17E-12 |
| NXN | 1.50871005 | 3.34168553 | 1.14726041 | 0.01293678 | 0.01678044 |
| GPLD1 | 9.5786527 | 2.86394667 | -1.7418181 | 9.07E-16 | 6.66E-15 |
| RPL39P36 | 0.45944602 | 0.99511176 | 1.11496319 | 1.78E-18 | 2.15E-17 |
| FETUB | 57.8400202 | 23.169289 | -1.3198543 | 1.09E-15 | 7.91E-15 |
| NT5DC2 | 3.14694333 | 10.7471227 | 1.77192735 | 4.46E-19 | 6.05E-18 |
| AC010280.2 | 1.22438941 | 0.40591303 | -1.5928199 | 3.00E-10 | 9.82E-10 |
| AL035461.2 | 1.25518519 | 3.21149449 | 1.35534459 | 2.93E-20 | 4.90E-19 |
| FMNL1 | 1.0444502 | 2.54958647 | 1.28751956 | 5.38E-16 | 4.12E-15 |
| FZD1 | 0.7726763 | 2.43741873 | 1.65741807 | 1.81E-12 | 8.07E-12 |
| SEPTIN5 | 0.90902414 | 2.3461378 | 1.36789725 | 6.96E-16 | 5.23E-15 |
| IMPDH1 | 2.05841936 | 7.49603654 | 1.86459106 | 3.40E-23 | 1.07E-21 |
| TPM4 | 12.639932 | 29.0274809 | 1.19943067 | 1.59E-18 | 1.94E-17 |
| AFP | 97.5121078 | 375.779751 | 1.94623406 | 3.54E-16 | 2.79E-15 |
| TRAM1L1 | 0.30113071 | 0.87224752 | 1.53434773 | 9.41E-14 | 5.06E-13 |
| H2AW | 1.50727471 | 5.10386938 | 1.75964903 | 5.00E-18 | 5.43E-17 |
| PTGDS | 35.2042253 | 170.210218 | 2.27349715 | 5.08E-05 | 8.80E-05 |
| ARID3A | 1.36570541 | 4.86181527 | 1.83184876 | 2.00E-25 | 1.12E-23 |
| TRIM60P18 | 0.45500293 | 1.02864024 | 1.17679076 | 6.10E-15 | 3.93E-14 |
| AKR7A3 | 48.5751341 | 19.0765276 | -1.3484194 | 1.96E-22 | 5.27E-21 |
| SERPINH1 | 13.4493025 | 34.159063 | 1.34473705 | 1.43E-23 | 4.80E-22 |
| MIR3189 | 1.0587773 | 2.17384783 | 1.03785178 | 0.00297299 | 0.00416686 |
| CD6 | 0.58393708 | 1.20290223 | 1.04263456 | 7.49E-07 | 1.61E-06 |
| CAPS | 2.00803816 | 5.08641229 | 1.34086172 | 1.48E-13 | 7.77E-13 |
| CEACAM6 | 0.11401076 | 4.72005506 | 5.37156182 | 8.61E-09 | 2.37E-08 |
| AC112206.3 | 0.70038469 | 1.44063277 | 1.04048319 | 0.00100875 | 0.00149909 |
| HLA-DOB | 0.81491744 | 1.92050615 | 1.23676078 | 9.78E-05 | 0.00016355 |
| IKBKE | 1.11191884 | 3.33222775 | 1.58343552 | 6.57E-26 | 4.29E-24 |
| IGHV4-34 | 2.58718182 | 10.1975977 | 1.97877598 | 0.00016567 | 0.00026944 |
| ERP27 | 0.36024568 | 2.21750366 | 2.62188344 | 1.25E-13 | 6.64E-13 |
| C5orf34 | 0.39199922 | 0.85687653 | 1.12823658 | 1.68E-21 | 3.66E-20 |
| RAP1GAP | 6.52113506 | 14.763013 | 1.17879219 | 1.74E-09 | 5.25E-09 |
| IGHV1-3 | 0.55681627 | 1.70268535 | 1.61253857 | 0.00017101 | 0.00027768 |
| IGHV3-21 | 4.11207251 | 15.9820939 | 1.95851882 | 0.00135501 | 0.00198314 |
| CFTR | 0.31487293 | 4.71010603 | 3.90291789 | 4.25E-05 | 7.44E-05 |
| KCNJ11 | 0.72035886 | 1.63195017 | 1.17980931 | 3.89E-10 | 1.26E-09 |
| ZNF56 | 0.53212874 | 1.06625502 | 1.00270532 | 8.29E-14 | 4.50E-13 |
| FGFR1 | 0.73621585 | 2.25401782 | 1.6142982 | 3.15E-07 | 7.07E-07 |
| CTAG2 | 2.40672659 | 11.5689421 | 2.26511278 | 0.00427252 | 0.00587606 |
| MT1CP | 5.63148188 | 0.77848713 | -2.8547695 | 2.30E-08 | 5.97E-08 |
| CCDC162P | 0.32347753 | 0.7866408 | 1.2820395 | 1.34E-08 | 3.60E-08 |
| GAL3ST1 | 2.5900245 | 12.8599428 | 2.31184658 | 7.05E-20 | 1.10E-18 |
| EHF | 1.29789811 | 3.23341137 | 1.31687993 | 1.91E-05 | 3.49E-05 |
| AL365226.1 | 0.50929544 | 1.49258034 | 1.55123387 | 1.33E-05 | 2.47E-05 |
| CNDP1 | 1.44334994 | 0.35093531 | -2.0401441 | 0.00044418 | 0.00068755 |
| KCND3 | 2.03969507 | 0.99587981 | -1.0343099 | 1.09E-06 | 2.29E-06 |
| C8A | 115.635616 | 39.2312128 | -1.559512 | 1.01E-32 | 7.13E-30 |
| AC102953.2 | 0.37180085 | 0.74935429 | 1.01111791 | 4.98E-14 | 2.81E-13 |
| IGHV4-59 | 5.33485983 | 29.9972262 | 2.49130683 | 0.00180685 | 0.00260415 |
| ACAP1 | 0.63318705 | 1.2734443 | 1.00803221 | 4.36E-08 | 1.09E-07 |
| AL035071.1 | 1.34153718 | 2.92723263 | 1.12565036 | 5.77E-25 | 2.86E-23 |
| MAL2 | 15.1150104 | 35.4055777 | 1.22799469 | 7.95E-15 | 5.05E-14 |
| FUT4 | 0.60544336 | 2.0439955 | 1.75532812 | 8.35E-14 | 4.52E-13 |
| ZNF28 | 0.78584798 | 1.73452217 | 1.14221613 | 3.26E-13 | 1.62E-12 |
| EVI2B | 1.98066097 | 3.97302045 | 1.00425427 | 1.59E-05 | 2.94E-05 |
| HPD | 738.114382 | 241.78307 | -1.6101312 | 3.09E-21 | 6.34E-20 |
| SLCO4A1 | 0.35111196 | 0.83629785 | 1.2520857 | 3.29E-07 | 7.36E-07 |
| FAM96AP2 | 0.35941484 | 0.91564563 | 1.34913939 | 0.00120501 | 0.00177429 |
| CHTF18 | 1.33065092 | 2.74898758 | 1.04676825 | 7.73E-21 | 1.45E-19 |
| SNAP25 | 0.72982479 | 3.45422551 | 2.2427402 | 6.93E-15 | 4.44E-14 |
| KCNB1 | 1.15313853 | 0.41768737 | -1.4650704 | 4.69E-10 | 1.51E-09 |
| TMEM54 | 6.51887388 | 16.2342042 | 1.31634199 | 4.50E-06 | 8.86E-06 |
| PDK4 | 45.7461122 | 14.213109 | -1.686427 | 1.15E-17 | 1.16E-16 |
| CDH11 | 0.47512543 | 0.99310807 | 1.0636423 | 8.13E-05 | 0.00013752 |
| NCF4 | 2.15772202 | 4.78638562 | 1.14942762 | 1.68E-12 | 7.56E-12 |
| TNNT1 | 0.22957704 | 1.7072971 | 2.89466387 | 1.95E-14 | 1.17E-13 |
| ALDH3B1 | 1.45460002 | 3.9636102 | 1.44619259 | 4.64E-22 | 1.15E-20 |
| CBX6 | 2.17812065 | 4.39322782 | 1.01219744 | 4.75E-12 | 2.00E-11 |
| KCNQ1 | 1.1550951 | 4.7805417 | 2.04916248 | 1.02E-10 | 3.55E-10 |
| WFDC21P | 1.17496625 | 3.4054397 | 1.53522177 | 6.81E-05 | 0.00011645 |
| TGFB3 | 1.65859421 | 3.61533096 | 1.12416676 | 1.05E-06 | 2.22E-06 |
| PKIB | 1.39013578 | 4.19714526 | 1.59418259 | 3.96E-10 | 1.28E-09 |
| TTC39A | 0.67871862 | 2.64756956 | 1.96378309 | 3.85E-14 | 2.21E-13 |
| PSPH | 6.38259338 | 12.8035694 | 1.00433142 | 3.63E-18 | 4.07E-17 |
| SLC10A1 | 94.9804968 | 20.1479329 | -2.2369995 | 3.79E-27 | 3.61E-25 |
| DUOXA2 | 1.01676372 | 5.19705413 | 2.35370962 | 0.00119215 | 0.00175701 |
| NAPSB | 1.36625239 | 3.2142018 | 1.23423649 | 2.72E-08 | 6.98E-08 |
| MYCN | 0.64417624 | 1.75079807 | 1.44248535 | 0.00345502 | 0.00480093 |
| CACNG4 | 0.94503159 | 3.15150195 | 1.7376051 | 1.57E-08 | 4.16E-08 |
| PLXNA3 | 0.85215583 | 2.28470964 | 1.42282164 | 7.80E-21 | 1.46E-19 |
| TLCD3A | 1.03002074 | 3.11732199 | 1.5976338 | 7.92E-27 | 6.97E-25 |
| CRYBA4 | 0.02602653 | 1.65744804 | 5.99283685 | 0.00080065 | 0.00120302 |
| BMF | 2.03756557 | 5.1075896 | 1.32579612 | 9.73E-20 | 1.49E-18 |
| IGKV1D-16 | 0.3809985 | 0.83920294 | 1.1392344 | 0.02393688 | 0.0299945 |
| COMP | 0.55031904 | 2.17382718 | 1.98189711 | 2.89E-06 | 5.82E-06 |
| SFRP2 | 0.49175561 | 1.98809567 | 2.01537377 | 2.03E-05 | 3.71E-05 |
| P3H4 | 2.31245147 | 6.71179203 | 1.53727493 | 1.14E-19 | 1.73E-18 |
| CREB3L1 | 1.02374642 | 3.60724517 | 1.81703907 | 4.00E-07 | 8.88E-07 |
| IGHV4-4 | 0.478533 | 1.726359 | 1.85104218 | 0.00907762 | 0.01200217 |
| COL2A1 | 2.23860357 | 5.7700036 | 1.36597315 | 0.00132941 | 0.001947 |
| COL8A2 | 0.30077692 | 1.23539107 | 2.03820203 | 1.13E-09 | 3.50E-09 |
| GPNMB | 6.07213272 | 14.0896507 | 1.21436062 | 6.21E-07 | 1.35E-06 |
| NDRG1 | 15.8764215 | 40.7814959 | 1.36102892 | 8.62E-14 | 4.66E-13 |
| ARMCX1 | 1.0462704 | 2.56060101 | 1.29122671 | 3.15E-09 | 9.17E-09 |
| SPATC1L | 1.41550213 | 4.37487856 | 1.62792905 | 1.55E-22 | 4.33E-21 |
| IGHG2 | 33.6808684 | 118.64231 | 1.81661735 | 9.48E-07 | 2.01E-06 |
| CYTH4 | 1.03325182 | 2.20835245 | 1.09577854 | 1.16E-12 | 5.35E-12 |
| ITIH5 | 0.3629313 | 5.22552427 | 3.84780739 | 4.70E-06 | 9.23E-06 |
| LIMK1 | 1.59379313 | 4.72425044 | 1.56762106 | 1.77E-24 | 7.60E-23 |
| ZNF320 | 0.59843033 | 1.70537726 | 1.51083571 | 8.10E-14 | 4.41E-13 |
| CFD | 3.07619771 | 7.37768633 | 1.26202023 | 3.93E-09 | 1.13E-08 |
| CYS1 | 0.43452715 | 2.19173041 | 2.33455212 | 1.80E-06 | 3.71E-06 |
| POF1B | 0.62117945 | 1.97702053 | 1.67024585 | 5.21E-09 | 1.47E-08 |
| TAT | 229.095242 | 44.6582889 | -2.3589476 | 1.78E-28 | 2.62E-26 |
| CLGN | 3.0585108 | 6.78663735 | 1.14986755 | 2.16E-07 | 4.97E-07 |
| TTLL3 | 0.41026101 | 0.85704108 | 1.0628223 | 1.17E-15 | 8.47E-15 |
| GNG4 | 1.55576593 | 3.12145706 | 1.0045946 | 1.77E-09 | 5.33E-09 |
| CHAF1B | 0.84530377 | 2.26097705 | 1.41940456 | 2.45E-24 | 1.01E-22 |
| TNFRSF18 | 0.72394616 | 2.27636673 | 1.65277869 | 5.77E-17 | 5.16E-16 |
| MCM2 | 5.09908486 | 11.2338837 | 1.13954652 | 1.37E-17 | 1.37E-16 |
| AC244394.2 | 1.23296275 | 0.47275401 | -1.3829676 | 1.54E-11 | 6.00E-11 |
| SLC44A3 | 4.1049222 | 9.58502488 | 1.2234273 | 3.84E-12 | 1.63E-11 |
| TMSB10 | 584.400281 | 1827.10342 | 1.64452952 | 1.12E-26 | 9.43E-25 |
| CERCAM | 0.82402713 | 3.02717708 | 1.87720933 | 2.60E-13 | 1.31E-12 |
| CORO2A | 1.41129868 | 2.94299778 | 1.06026311 | 5.24E-09 | 1.48E-08 |
| AC092687.3 | 0.58242463 | 1.26419998 | 1.11808143 | 3.72E-17 | 3.45E-16 |
| CTSK | 3.95814027 | 12.5766367 | 1.66785152 | 1.65E-10 | 5.56E-10 |
| RAVER2 | 0.75985715 | 1.80549124 | 1.24859129 | 1.24E-14 | 7.66E-14 |
| CLIC3 | 0.40550242 | 2.63788388 | 2.70159862 | 1.02E-14 | 6.34E-14 |
| ARHGAP9 | 0.71699458 | 1.62070334 | 1.17658591 | 4.62E-11 | 1.67E-10 |
| TRBC2 | 4.46967999 | 10.020013 | 1.16464093 | 2.24E-07 | 5.14E-07 |
| MUC20 | 1.2395937 | 4.41313907 | 1.83193788 | 0.03899401 | 0.04756824 |
| SFI1 | 0.85971227 | 1.97008733 | 1.19633379 | 7.51E-28 | 8.97E-26 |
| ZNF71 | 0.50571631 | 1.05545524 | 1.06146518 | 5.46E-19 | 7.27E-18 |
| EPS8L3 | 3.59627185 | 8.79984883 | 1.29097666 | 1.32E-12 | 6.03E-12 |
| AJM1 | 0.874853 | 1.86828844 | 1.09460469 | 5.28E-15 | 3.44E-14 |
| CCL5 | 10.6122806 | 22.2490681 | 1.06801019 | 6.80E-05 | 0.00011623 |
| COL4A5 | 0.69903591 | 1.53961045 | 1.1391269 | 4.98E-07 | 1.09E-06 |
| CDH6 | 0.3878409 | 1.83032317 | 2.23856153 | 1.55E-10 | 5.25E-10 |
| MBOAT4 | 0.44427075 | 0.90308692 | 1.02342568 | 6.39E-12 | 2.63E-11 |
| NAP1L1 | 7.32134788 | 15.5896909 | 1.09041115 | 8.63E-29 | 1.41E-26 |
| UGT2B7 | 152.794103 | 61.6099263 | -1.3103541 | 4.22E-18 | 4.67E-17 |
| AC137056.2 | 0.70227672 | 0.2906851 | -1.2725825 | 1.73E-22 | 4.75E-21 |
| ANG | 359.573237 | 158.598066 | -1.1809105 | 2.33E-15 | 1.61E-14 |
| HK3 | 0.69648947 | 1.58783934 | 1.1888915 | 2.20E-06 | 4.47E-06 |
| TUBA1A | 5.46424932 | 13.4666549 | 1.30129632 | 1.72E-12 | 7.71E-12 |
| TMEM147 | 22.2054672 | 44.9844796 | 1.01851241 | 6.83E-31 | 2.83E-28 |
| PLAGL2 | 1.39626805 | 2.81269549 | 1.01037744 | 1.73E-19 | 2.53E-18 |
| HRG | 558.726842 | 183.115933 | -1.6093858 | 8.67E-21 | 1.60E-19 |
| CRYGS | 0.66894666 | 2.97323912 | 2.15207241 | 4.52E-16 | 3.49E-15 |
| ZNF256 | 0.74306902 | 1.53832332 | 1.04979064 | 1.54E-13 | 8.04E-13 |
| TMC8 | 1.02333329 | 2.10554943 | 1.04092065 | 9.55E-08 | 2.30E-07 |
| KIF11 | 1.17144945 | 3.08142878 | 1.39530475 | 2.25E-22 | 5.95E-21 |
| UBE2C | 6.75779161 | 23.4600922 | 1.79558492 | 9.56E-25 | 4.42E-23 |
| PDE4A | 0.81047531 | 1.98428798 | 1.29178127 | 3.95E-17 | 3.64E-16 |
| PRAMEF10 | 1.9594696 | 0.68949163 | -1.5068582 | 6.38E-08 | 1.57E-07 |
| PLCH2 | 1.26355623 | 0.5844086 | -1.1124405 | 0.00352399 | 0.00489242 |
| RAD54L | 0.53617524 | 1.42399016 | 1.40916267 | 4.82E-18 | 5.26E-17 |
| SNORC | 4.47539094 | 11.5956386 | 1.37349665 | 0.00584443 | 0.0079243 |
| PLEKHG6 | 1.55358772 | 3.19597156 | 1.04065087 | 2.68E-08 | 6.91E-08 |
| IGHV3-43 | 1.17369443 | 4.36948892 | 1.89640769 | 0.01135461 | 0.01482916 |
| TBXAS1 | 0.81004877 | 1.62397312 | 1.00344707 | 4.75E-09 | 1.35E-08 |
| IGLV4-60 | 0.55637798 | 2.6873033 | 2.27202192 | 0.00870006 | 0.01152568 |
| IGKV3D-20 | 1.07067224 | 7.86307106 | 2.87657599 | 4.80E-05 | 8.36E-05 |
| HOXA10 | 0.51865365 | 1.09321698 | 1.07573642 | 3.23E-07 | 7.23E-07 |
| LMNB1 | 4.92731961 | 10.3917841 | 1.07656841 | 5.38E-17 | 4.85E-16 |
| BIRC7 | 0.071401 | 1.32386768 | 4.21267088 | 9.05E-14 | 4.88E-13 |
| EPHB3 | 0.27397441 | 1.33508911 | 2.284823 | 2.40E-18 | 2.81E-17 |
| SLC29A4 | 2.87090953 | 7.42012997 | 1.36993659 | 2.47E-16 | 2.00E-15 |
| MACIR | 0.69874827 | 1.78883442 | 1.35617514 | 5.93E-12 | 2.46E-11 |
| TMEM132A | 0.68982727 | 5.60932256 | 3.02351948 | 7.74E-22 | 1.82E-20 |
| KCNMB2-AS1 | 0.41482373 | 1.22389912 | 1.56091432 | 2.44E-05 | 4.39E-05 |
| ZNF528 | 0.33103642 | 0.76406921 | 1.20671336 | 8.21E-08 | 1.99E-07 |
| AL162582.1 | 2.45065955 | 0.9960386 | -1.2988965 | 0.00018054 | 0.0002924 |
| IGHV3-74 | 3.0722508 | 6.18346266 | 1.00911896 | 0.00398859 | 0.00550222 |
| MT1X | 123.802455 | 48.3690004 | -1.3558853 | 2.61E-08 | 6.73E-08 |
| NDC80 | 1.53984871 | 3.70416027 | 1.26635791 | 1.53E-18 | 1.88E-17 |
| SMIM6 | 1.73675584 | 4.60246457 | 1.40601167 | 1.93E-08 | 5.05E-08 |
| IGHV2-5 | 0.53937158 | 1.5192365 | 1.49399506 | 0.00648004 | 0.0087382 |
| IGHV1-18 | 7.93962476 | 27.8690047 | 1.81151875 | 0.00022791 | 0.00036547 |
| MSI1 | 1.50649538 | 4.0946428 | 1.44254136 | 3.33E-09 | 9.66E-09 |
| C3P1 | 22.8672121 | 9.15994456 | -1.3198697 | 3.49E-16 | 2.74E-15 |
| GCNT1 | 0.23194417 | 0.91511983 | 1.98018311 | 4.75E-07 | 1.05E-06 |
| IGHV1-2 | 3.29127018 | 16.195714 | 2.29889571 | 0.02780806 | 0.03453531 |
| DBF4B | 0.50500572 | 1.02171564 | 1.0166221 | 1.72E-20 | 2.99E-19 |
| CENPO | 0.84384346 | 1.93343158 | 1.19611642 | 3.31E-28 | 4.32E-26 |
| NFATC4 | 0.53285711 | 1.35552668 | 1.34703289 | 2.77E-14 | 1.62E-13 |
| GPX8 | 0.80719146 | 2.13756895 | 1.40498813 | 1.30E-08 | 3.48E-08 |
| S100A8 | 2.12301385 | 10.9060296 | 2.36094029 | 0.00015833 | 0.0002583 |
| GBGT1 | 0.40524822 | 1.02574888 | 1.33979982 | 4.88E-13 | 2.37E-12 |
| RIDA | 209.347062 | 90.5267646 | -1.2094804 | 6.57E-27 | 5.94E-25 |
| TSPYL5 | 0.38573639 | 1.02053133 | 1.40363331 | 4.99E-05 | 8.67E-05 |
| DKK1 | 3.4572615 | 14.8205235 | 2.09989477 | 5.08E-12 | 2.13E-11 |
| PIF1 | 0.38798835 | 1.10746565 | 1.5131767 | 2.21E-26 | 1.68E-24 |
| RAB7B | 0.67021965 | 1.38899817 | 1.0513388 | 3.70E-16 | 2.90E-15 |
| ABCC9 | 2.59298965 | 1.28073764 | -1.0176415 | 2.17E-11 | 8.23E-11 |
| IGLV3-19 | 14.9797187 | 116.804275 | 2.96301064 | 0.00092725 | 0.00138293 |
| ALKAL2 | 0.35242236 | 1.73629029 | 2.30063079 | 0.01246167 | 0.01619371 |
| PYCARD | 6.47825417 | 14.8919259 | 1.20085337 | 5.91E-15 | 3.83E-14 |
| GNA15 | 0.70954955 | 1.631996 | 1.20166217 | 3.87E-13 | 1.90E-12 |
| SMAGP | 1.90489062 | 4.29370015 | 1.17251328 | 4.38E-19 | 5.95E-18 |
| HAVCR1 | 0.55207299 | 1.32672084 | 1.26493391 | 1.94E-07 | 4.50E-07 |
| UROC1 | 16.1178848 | 6.22947293 | -1.3714804 | 4.74E-05 | 8.26E-05 |
| CCDC9B | 0.86510404 | 2.34360453 | 1.43778359 | 2.00E-05 | 3.65E-05 |
| SMPDL3B | 0.42360492 | 1.30589148 | 1.62424377 | 5.64E-12 | 2.34E-11 |
| MOGAT2 | 8.87325746 | 2.50490832 | -1.824706 | 1.78E-17 | 1.75E-16 |
| ABCA8 | 2.83970622 | 0.97365432 | -1.5442601 | 1.59E-16 | 1.33E-15 |
| ELOVL7 | 1.20725104 | 4.38413375 | 1.86056611 | 2.11E-12 | 9.31E-12 |
| CENPM | 2.33436172 | 7.08594818 | 1.60193278 | 1.74E-25 | 9.97E-24 |
| CDKN1C | 1.61871869 | 6.06153216 | 1.90483022 | 1.01E-14 | 6.30E-14 |
| TACSTD2 | 1.10870047 | 5.57449653 | 2.32997185 | 0.00017521 | 0.00028417 |
| FGD6 | 0.90556856 | 2.08554813 | 1.20353083 | 3.77E-09 | 1.09E-08 |
| SELENOM | 8.1190894 | 20.5952859 | 1.34292431 | 4.65E-18 | 5.09E-17 |
| SLAMF6 | 0.58664858 | 1.19880999 | 1.03103457 | 0.02795499 | 0.03470249 |
| FAM99A | 7.1507702 | 3.40189609 | -1.0717596 | 7.99E-05 | 0.00013531 |
| ATP2A3 | 1.25097725 | 2.68780846 | 1.10337478 | 4.94E-08 | 1.23E-07 |
| APLP1 | 0.55388717 | 2.75971685 | 2.31685621 | 4.69E-08 | 1.17E-07 |
| SIX2 | 0.13589047 | 1.35036135 | 3.31282934 | 1.22E-13 | 6.49E-13 |
| BAIAP2L2 | 4.44054401 | 10.4051774 | 1.22849322 | 2.12E-10 | 7.05E-10 |
| SLC9A1 | 1.11405452 | 2.71174422 | 1.28340127 | 3.39E-15 | 2.27E-14 |
| MMP11 | 1.77288628 | 7.29884945 | 2.04156906 | 7.23E-21 | 1.36E-19 |
| PTPN7 | 0.52870938 | 1.23261306 | 1.22117316 | 4.95E-11 | 1.78E-10 |
| XCL2 | 0.77137176 | 1.56278469 | 1.0186208 | 0.00015831 | 0.0002583 |
| AC112206.2 | 1.6626624 | 0.54447134 | -1.6105673 | 3.40E-11 | 1.26E-10 |
| CHST10 | 0.40154877 | 1.3045329 | 1.69988621 | 4.15E-19 | 5.67E-18 |
| INAVA | 0.89394372 | 2.42963837 | 1.44248569 | 7.42E-13 | 3.51E-12 |
| SLC4A3 | 0.21444575 | 1.30046538 | 2.60034336 | 1.30E-15 | 9.35E-15 |
| KAAG1 | 0.19765854 | 1.13943101 | 2.52723141 | 1.04E-09 | 3.22E-09 |
| KLHDC7B | 0.54116283 | 1.5584326 | 1.52596111 | 0.00133418 | 0.00195367 |
| LINC01506 | 0.79895692 | 0.27049635 | -1.5625086 | 0.00070383 | 0.00106435 |
| LAMA5 | 3.30190219 | 8.05458591 | 1.28651304 | 4.07E-10 | 1.31E-09 |
| SSTR5-AS1 | 1.00656981 | 2.14998617 | 1.09488015 | 5.32E-05 | 9.20E-05 |
| CERKL | 0.57162414 | 1.15238965 | 1.01148985 | 9.87E-12 | 3.95E-11 |
| ZG16B | 0.40761653 | 1.54642602 | 1.92365336 | 6.27E-09 | 1.75E-08 |
| MARCKSL1 | 26.9042572 | 67.3298679 | 1.32341216 | 9.10E-21 | 1.67E-19 |
