# Supplementary material for: MTFR2-dependent mitochondrial fission promotes HCC progression
Source: J Transl Med. 2024 Jan 18;22:73. doi: 10.1186/s12967-023-04845-6 (PMC10795309; doi:10.1186/s12967-023-04845-6)
Supplement: Supplementary file 2 — Additional file 2: Table S1. The symbols of mitochondrial dynamical genes. Table S2. The differentially expressed genes in the two clusters (|log2FC| > 1 and false discovery rate (FDR) < 0.001). Table S3. The differentially expressed genes in the two clusters (|log2FC| > 0.7 and false discovery rate (FDR) < 0.001). Table S4. The genes selected from the differentially expressed genes of the two clusters by univariate Cox regression analysis (P < 0.05). Table S5. The 11 topological analysis method results calculated by CytoHubba. [file 12967_2023_4845_MOESM2_ESM.zip › Supplementary tables/Table S4.docx]

Table S4. The genes selected from the differentially expressed genes of the two clusters by univariate Cox regression analysis (P < 0.05).

| ID | HR | HR.95L | HR.95H | pvalue |
| --- | --- | --- | --- | --- |
| BCORL1 | 1.23436964 | 1.12693444 | 1.35204707 | 5.84E-06 |
| XXYLT1 | 1.10429302 | 1.01140148 | 1.20571612 | 0.02690848 |
| ASPDH | 0.99162061 | 0.98332217 | 0.99998908 | 0.04970293 |
| NEURL3 | 1.01109811 | 1.00205194 | 1.02022596 | 0.01608407 |
| TGFB2 | 1.03507015 | 1.00677227 | 1.0641634 | 0.01480178 |
| ARNT2 | 1.11471562 | 1.01586 | 1.22319109 | 0.02190131 |
| TRIP10 | 1.03923041 | 1.004052 | 1.07564135 | 0.02851566 |
| ELFN1 | 0.95660412 | 0.92644448 | 0.98774558 | 0.00664094 |
| SFN | 1.00257451 | 1.0007999 | 1.00435226 | 0.00444739 |
| HK2 | 1.05278883 | 1.02154425 | 1.08498904 | 0.00081789 |
| C6 | 0.99426131 | 0.99021581 | 0.99832335 | 0.00566374 |
| RIBC2 | 1.27796099 | 1.14377451 | 1.42789009 | 1.47E-05 |
| FANCI | 1.15730271 | 1.06414337 | 1.25861758 | 0.00064506 |
| TEDC2 | 1.13071118 | 1.05482853 | 1.2120527 | 0.00052834 |
| SPI1 | 1.02057198 | 1.00272944 | 1.03873202 | 0.02364442 |
| PDP1 | 1.09239245 | 1.00986578 | 1.18166324 | 0.02745985 |
| TRAIP | 1.26717402 | 1.13406711 | 1.41590387 | 2.89E-05 |
| PON1 | 0.99487082 | 0.99234478 | 0.99740329 | 7.36E-05 |
| SKA3 | 1.30033065 | 1.17914033 | 1.43397672 | 1.43E-07 |
| HIC2 | 1.24098822 | 1.10492291 | 1.39380923 | 0.00026857 |
| ATP13A2 | 1.06287564 | 1.03102541 | 1.09570979 | 8.56E-05 |
| MUC5B | 1.00940072 | 1.00302622 | 1.01581573 | 0.003794 |
| MARCKS | 1.017364 | 1.01038192 | 1.02439432 | 9.61E-07 |
| RAB42 | 1.15810932 | 1.05716365 | 1.26869401 | 0.00160697 |
| LRP12 | 1.45444624 | 1.23654487 | 1.71074573 | 6.07E-06 |
| PRR36 | 1.25983164 | 1.06316116 | 1.49288352 | 0.00764846 |
| ELF4 | 1.07385918 | 1.02086318 | 1.12960637 | 0.00578704 |
| ZWINT | 1.05040831 | 1.03029079 | 1.07091865 | 6.21E-07 |
| FAM117B | 1.07540606 | 1.01777861 | 1.13629643 | 0.00967915 |
| IGHV3-73 | 1.0043654 | 1.0017144 | 1.00702342 | 0.00123688 |
| FABP5 | 1.03509507 | 1.01174742 | 1.05898151 | 0.00304365 |
| ARMCX6 | 1.16840845 | 1.06342809 | 1.28375235 | 0.0011942 |
| CCNJ | 1.23698256 | 1.03607063 | 1.47685477 | 0.01868195 |
| RDH16 | 0.99534873 | 0.99129574 | 0.99941829 | 0.02512525 |
| SLC7A7 | 1.07490108 | 1.02216382 | 1.13035924 | 0.00489231 |
| ORC6 | 1.30434291 | 1.15976882 | 1.46693927 | 9.30E-06 |
| CARMIL1 | 1.14121508 | 1.05750284 | 1.23155401 | 0.00067789 |
| FSCN1 | 1.00664174 | 1.0016106 | 1.01169816 | 0.00961214 |
| TRIM6 | 1.40035907 | 1.14465372 | 1.7131867 | 0.00106315 |
| CUX2 | 0.9674648 | 0.93679529 | 0.9991384 | 0.04417696 |
| NRGN | 1.07192783 | 1.01942789 | 1.12713148 | 0.00670891 |
| G6PC | 0.99793005 | 0.99657736 | 0.99928457 | 0.00275248 |
| C1QC | 1.00140694 | 1.00025119 | 1.00256402 | 0.01702117 |
| KIF2C | 1.10734443 | 1.07289872 | 1.14289602 | 2.55E-10 |
| UGT2B15 | 0.99795393 | 0.99620024 | 0.99971071 | 0.02246694 |
| SMIM10 | 1.11663372 | 1.03337886 | 1.20659606 | 0.00526279 |
| AC004812.2 | 1.50830338 | 1.07130863 | 2.12355154 | 0.01854304 |
| RAB6B | 1.18147601 | 1.05257799 | 1.3261588 | 0.00466428 |
| RNF24 | 1.2563517 | 1.11038322 | 1.42150888 | 0.00029283 |
| TES | 1.02256774 | 1.00114249 | 1.04445151 | 0.0388613 |
| WASF1 | 1.11774175 | 1.06659287 | 1.1713435 | 3.20E-06 |
| CCNB1 | 1.04403769 | 1.02938868 | 1.05889516 | 2.26E-09 |
| B3GNT7 | 1.01793416 | 1.00465299 | 1.03139089 | 0.00798359 |
| NRM | 1.02147052 | 1.00469232 | 1.03852892 | 0.01193836 |
| EXO1 | 1.2738349 | 1.15343412 | 1.40680366 | 1.77E-06 |
| PHLDB1 | 1.17927048 | 1.01677914 | 1.36772953 | 0.02926147 |
| MKI67 | 1.08361854 | 1.04706075 | 1.12145274 | 4.51E-06 |
| TMEM237 | 1.47990283 | 1.27719336 | 1.71478528 | 1.84E-07 |
| GLYATL1 | 0.98191972 | 0.96784127 | 0.99620296 | 0.01327629 |
| TUBB6 | 1.02141279 | 1.00080898 | 1.04244079 | 0.04157597 |
| RELT | 1.64812297 | 1.23452841 | 2.20028093 | 0.00070125 |
| SLC39A4 | 1.01474594 | 1.00006695 | 1.02964038 | 0.04895669 |
| SFT2D1 | 1.12840222 | 1.06478244 | 1.19582323 | 4.50E-05 |
| SULT1C2 | 1.05485711 | 1.00779779 | 1.10411387 | 0.02181638 |
| G6PD | 1.01282417 | 1.00872471 | 1.0169403 | 7.37E-10 |
| CDC25B | 1.02159379 | 1.01098393 | 1.03231499 | 6.05E-05 |
| MYG1-AS1 | 1.52132211 | 1.21040934 | 1.91209774 | 0.00032185 |
| LOX | 1.06594059 | 1.02567185 | 1.10779031 | 0.00115384 |
| RHEX | 1.03513851 | 1.01033181 | 1.06055429 | 0.00526252 |
| NAALADL1 | 1.0220439 | 1.00627971 | 1.03805505 | 0.00597251 |
| KPNA2 | 1.02658289 | 1.01835418 | 1.0348781 | 1.67E-10 |
| CKAP2L | 1.31581353 | 1.15928082 | 1.49348218 | 2.17E-05 |
| EME1 | 1.32226101 | 1.12865861 | 1.54907266 | 0.0005435 |
| EPHB6 | 1.04007486 | 1.00427986 | 1.07714568 | 0.02788003 |
| TYMS | 1.02878435 | 1.01174818 | 1.04610739 | 0.00086572 |
| ENO2 | 1.03438796 | 1.00434211 | 1.06533266 | 0.02457301 |
| ANKS6 | 1.16313807 | 1.02017948 | 1.32612957 | 0.02391148 |
| CFHR3 | 0.99042081 | 0.98401693 | 0.99686637 | 0.00363441 |
| PHF19 | 1.16713549 | 1.08184388 | 1.25915142 | 6.56E-05 |
| PIGS | 1.07942717 | 1.03717069 | 1.12340527 | 0.00017598 |
| ECM2 | 0.91967914 | 0.8738919 | 0.96786538 | 0.00131118 |
| ANXA10 | 0.97043291 | 0.95128871 | 0.98996238 | 0.00315375 |
| RNF227 | 1.73733615 | 1.30193502 | 2.31834682 | 0.0001751 |
| PKMYT1 | 1.20703145 | 1.08534438 | 1.34236188 | 0.00051956 |
| ZNF107 | 1.30631499 | 1.03164815 | 1.65410936 | 0.02650927 |
| PCK1 | 0.99825979 | 0.99661653 | 0.99990577 | 0.03825882 |
| SERPINC1 | 0.99982384 | 0.99965981 | 0.99998791 | 0.03534015 |
| PBK | 1.10954155 | 1.06833173 | 1.15234098 | 7.33E-08 |
| ASF1B | 1.05447171 | 1.02564247 | 1.08411129 | 0.00017674 |
| PKM | 1.00296523 | 1.00030529 | 1.00563225 | 0.02887054 |
| PIGZ | 1.13893834 | 1.06584144 | 1.21704833 | 0.00012101 |
| LINC02362 | 0.92673199 | 0.86772981 | 0.98974608 | 0.02338745 |
| FCGR1A | 1.10667887 | 1.03172665 | 1.18707618 | 0.00461315 |
| SLC2A1 | 1.06070771 | 1.03388208 | 1.08822937 | 6.50E-06 |
| SLC7A8 | 1.07410517 | 1.0172647 | 1.13412164 | 0.00996577 |
| PIMREG | 1.15137409 | 1.05543628 | 1.25603251 | 0.00149612 |
| BEND3 | 2.02039506 | 1.54343483 | 2.64474801 | 3.07E-07 |
| FANCG | 1.13568696 | 1.06562083 | 1.21036004 | 9.00E-05 |
| UGT1A10 | 1.13009448 | 1.06350403 | 1.20085444 | 7.92E-05 |
| DUSP9 | 1.00807737 | 1.00321993 | 1.01295833 | 0.00109683 |
| AL390198.1 | 1.10023197 | 1.01539548 | 1.19215657 | 0.01964083 |
| GPR35 | 1.07409818 | 1.0003082 | 1.15333144 | 0.04901631 |
| KIF23 | 1.19480552 | 1.11595169 | 1.27923121 | 3.23E-07 |
| IL2RG | 1.01007334 | 1.00004807 | 1.02019912 | 0.04890621 |
| ALDOB | 0.99983772 | 0.99970329 | 0.99997216 | 0.01799511 |
| AC141557.1 | 1.2601201 | 1.11056553 | 1.42981446 | 0.00033466 |
| AACS | 1.20699608 | 1.05585345 | 1.37977437 | 0.00584796 |
| AURKB | 1.03447359 | 1.01850879 | 1.05068862 | 1.95E-05 |
| TMEM91 | 1.09855506 | 1.01826411 | 1.18517701 | 0.01520896 |
| VXN | 0.71978343 | 0.52283844 | 0.99091449 | 0.04380809 |
| TRPM2 | 1.24984675 | 1.11280569 | 1.40376429 | 0.00016736 |
| PLA2G7 | 1.02462869 | 1.01082215 | 1.03862381 | 0.00043963 |
| TK1 | 1.0122131 | 1.00456475 | 1.01991968 | 0.00170774 |
| HTRA3 | 1.03714893 | 1.00560369 | 1.06968372 | 0.02063738 |
| CGAS | 1.36075038 | 1.09360103 | 1.69316007 | 0.0057387 |
| CDK19 | 1.13793868 | 1.05447151 | 1.22801273 | 0.00088547 |
| CLIP2 | 1.03495435 | 1.00118748 | 1.06986007 | 0.04234766 |
| SGO2 | 1.53304002 | 1.33758159 | 1.75706044 | 8.26E-10 |
| GPRIN1 | 1.38913918 | 1.22025043 | 1.58140298 | 6.71E-07 |
| ACKR2 | 0.85975942 | 0.76590524 | 0.96511451 | 0.01040621 |
| CYP8B1 | 0.99704807 | 0.99477256 | 0.99932879 | 0.01121517 |
| ESPL1 | 1.22091959 | 1.11012966 | 1.34276624 | 3.91E-05 |
| ITGA3 | 1.028379 | 1.00610505 | 1.05114606 | 0.01225384 |
| ANKRD13D | 1.11781125 | 1.02326236 | 1.22109641 | 0.01351305 |
| PLPP2 | 1.02700968 | 1.00768107 | 1.04670904 | 0.00597227 |
| CHEK1 | 1.37656487 | 1.20948006 | 1.56673177 | 1.29E-06 |
| CSAG1 | 1.01841295 | 1.00595623 | 1.03102392 | 0.00366408 |
| CDH24 | 1.18821259 | 1.059312 | 1.33279822 | 0.00324593 |
| AC004816.1 | 1.2091472 | 1.08114076 | 1.35230953 | 0.00087954 |
| VNN2 | 1.01773679 | 1.00819788 | 1.02736595 | 0.00025295 |
| TACC3 | 1.06611352 | 1.03841209 | 1.09455393 | 1.88E-06 |
| TMEM44 | 1.17141153 | 1.05419551 | 1.3016608 | 0.00327047 |
| FAM110A | 1.10889239 | 1.03390065 | 1.18932349 | 0.00381431 |
| TOP2A | 1.03242945 | 1.01699141 | 1.04810184 | 3.30E-05 |
| ZNF124 | 1.56033261 | 1.14418313 | 2.12783933 | 0.00493921 |
| PRR11 | 1.22044756 | 1.14490743 | 1.30097177 | 9.90E-10 |
| SLC41A1 | 1.07305435 | 1.02641687 | 1.12181091 | 0.00187059 |
| GALNT3 | 1.07784084 | 1.0110306 | 1.149066 | 0.02167732 |
| DOK1 | 1.13288151 | 1.0071715 | 1.27428201 | 0.03761354 |
| ZSWIM4 | 1.09833838 | 1.01752498 | 1.1855701 | 0.01615 |
| PIP4P2 | 1.13518093 | 1.06351541 | 1.21167566 | 0.00013854 |
| MAT1A | 0.99849346 | 0.99721539 | 0.99977316 | 0.02104775 |
| DNMT3B | 1.23269445 | 1.08433948 | 1.40134675 | 0.00138589 |
| GLS | 1.06371277 | 1.02591183 | 1.10290654 | 0.00082087 |
| CDT1 | 1.045903 | 1.01765668 | 1.07493333 | 0.00131376 |
| FKBP10 | 1.00965017 | 1.00191198 | 1.01744813 | 0.01442229 |
| KIFC1 | 1.06268566 | 1.03676779 | 1.08925144 | 1.39E-06 |
| KNTC1 | 1.16864285 | 1.06116986 | 1.28700047 | 0.00154445 |
| BCAT1 | 1.06539709 | 1.01241569 | 1.12115109 | 0.01492919 |
| TNFRSF21 | 1.01312247 | 1.00300783 | 1.02333911 | 0.01087716 |
| LYPD1 | 1.01948342 | 1.0015443 | 1.03774385 | 0.03314467 |
| PLG | 0.99864174 | 0.99728851 | 0.99999681 | 0.04946361 |
| KIF3C | 1.11285571 | 1.03437931 | 1.19728595 | 0.00415814 |
| TMEM65 | 1.12213057 | 1.04630841 | 1.20344729 | 0.00124591 |
| MAPRE1 | 1.02500805 | 1.01276077 | 1.03740344 | 5.64E-05 |
| AC012073.1 | 1.41097718 | 1.23059304 | 1.61780258 | 8.09E-07 |
| DBN1 | 1.02400401 | 1.00806607 | 1.04019393 | 0.00303936 |
| ZNF468 | 1.15345486 | 1.05273657 | 1.26380915 | 0.00219566 |
| UBE2T | 1.04455781 | 1.02453364 | 1.06497334 | 1.01E-05 |
| SMYD3 | 1.17290769 | 1.03185098 | 1.33324722 | 0.01470458 |
| FCGBP | 1.04484554 | 1.01156164 | 1.07922459 | 0.00790946 |
| AP000240.1 | 1.27832197 | 1.06210299 | 1.538558 | 0.00939703 |
| MYBL2 | 1.02066897 | 1.01128503 | 1.03013998 | 1.42E-05 |
| MEAK7 | 1.38622152 | 1.08907534 | 1.76444186 | 0.00797358 |
| ZFPM2-AS1 | 1.09620742 | 1.05632169 | 1.1375992 | 1.19E-06 |
| GAS5 | 1.00506621 | 1.00025208 | 1.00990351 | 0.0391268 |
| F11 | 0.98032492 | 0.96593836 | 0.99492575 | 0.00842897 |
| SMTN | 1.08606657 | 1.01192747 | 1.1656375 | 0.02210041 |
| CENPA | 1.18052742 | 1.11943795 | 1.24495063 | 9.25E-10 |
| TBX19 | 1.47463821 | 1.15970418 | 1.87509702 | 0.00153118 |
| ADAM9 | 1.04049201 | 1.02327429 | 1.05799944 | 3.12E-06 |
| ZIC2 | 1.12725297 | 1.06343412 | 1.19490172 | 5.62E-05 |
| TUBA1C | 1.03445047 | 1.0214261 | 1.04764091 | 1.61E-07 |
| PARPBP | 1.44158865 | 1.19821992 | 1.73438766 | 0.00010585 |
| MPP3 | 1.227038 | 1.04371971 | 1.44255421 | 0.01320199 |
| CDCA2 | 1.50591481 | 1.29607649 | 1.74972653 | 8.93E-08 |
| BORA | 2.12834213 | 1.5252935 | 2.96981545 | 8.84E-06 |
| ADA | 1.09068174 | 1.03515712 | 1.14918463 | 0.00112952 |
| RACGAP1 | 1.10642595 | 1.06504695 | 1.1494126 | 1.99E-07 |
| MAP3K21 | 1.08147507 | 1.00878135 | 1.15940716 | 0.02736781 |
| PFKP | 1.00832513 | 1.00028687 | 1.01642799 | 0.04233604 |
| DNM1 | 1.0955452 | 1.0192517 | 1.17754944 | 0.01322216 |
| NPTX2 | 1.01157901 | 1.00593447 | 1.01725523 | 5.52E-05 |
| SPATS2 | 1.24867996 | 1.14772767 | 1.35851185 | 2.43E-07 |
| CCDC183 | 1.18490842 | 1.02934478 | 1.36398221 | 0.01814072 |
| CCNF | 1.3607014 | 1.20959903 | 1.53067938 | 2.92E-07 |
| SPP1 | 1.0001852 | 1.00006301 | 1.00030741 | 0.00297177 |
| SMOX | 1.04671878 | 1.02098115 | 1.07310522 | 0.00032486 |
| DCAF16 | 1.13220237 | 1.06100283 | 1.20817983 | 0.00017907 |
| ZNF813 | 1.36502277 | 1.05071033 | 1.77335952 | 0.01978348 |
| C3orf85 | 0.79570227 | 0.63720681 | 0.99362106 | 0.04375517 |
| OSBPL3 | 1.24936015 | 1.07890108 | 1.4467506 | 0.00293305 |
| WDHD1 | 1.49301852 | 1.27497185 | 1.7483557 | 6.50E-07 |
| SLC27A5 | 0.99544722 | 0.99160882 | 0.99930049 | 0.02061504 |
| DTNBP1 | 1.09967742 | 1.03346465 | 1.17013237 | 0.00270985 |
| TMEM156 | 1.03117122 | 1.00177161 | 1.06143363 | 0.03753456 |
| FAAP24 | 1.22823092 | 1.06327204 | 1.41878197 | 0.00521076 |
| CHST11 | 1.08619 | 1.0211552 | 1.1553667 | 0.00867705 |
| RBP2 | 1.01936115 | 1.01015063 | 1.02865566 | 3.46E-05 |
| AC107959.3 | 1.35107146 | 1.14046567 | 1.60056908 | 0.00050115 |
| TNFAIP8L3 | 1.11732264 | 1.03241449 | 1.20921383 | 0.00594052 |
| TEAD2 | 1.0185078 | 1.00254112 | 1.03472878 | 0.02291967 |
| TRIM47 | 1.02154086 | 1.00757884 | 1.03569635 | 0.00240319 |
| E2F5 | 1.39153469 | 1.20894064 | 1.6017071 | 4.15E-06 |
| PGAP4 | 1.05840329 | 1.00683149 | 1.11261669 | 0.02594105 |
| CNTNAP1 | 1.14612191 | 1.05082542 | 1.25006057 | 0.00207489 |
| MECOM | 1.22347918 | 1.09783019 | 1.36350895 | 0.00026416 |
| MEX3A | 1.18348701 | 1.1129467 | 1.25849827 | 7.75E-08 |
| ARHGEF39 | 1.4725781 | 1.21647807 | 1.78259379 | 7.18E-05 |
| BUB1 | 1.25705662 | 1.15188076 | 1.37183587 | 2.87E-07 |
| UAP1L1 | 1.0726159 | 1.03802501 | 1.1083595 | 2.77E-05 |
| LPCAT1 | 1.03398624 | 1.01854547 | 1.04966109 | 1.34E-05 |
| MFSD6 | 1.05809122 | 1.00665946 | 1.11215072 | 0.02634849 |
| SNHG7 | 1.02935466 | 1.00484084 | 1.0544665 | 0.01863994 |
| KIF18A | 1.46617493 | 1.27900124 | 1.68074029 | 3.99E-08 |
| CLDN4 | 1.01159825 | 1.00412356 | 1.01912857 | 0.00230776 |
| SLC1A7 | 1.02974097 | 1.016969 | 1.04267334 | 4.18E-06 |
| LDOC1 | 1.02285661 | 1.00780355 | 1.0381345 | 0.00281206 |
| EIF5A2 | 1.13578274 | 1.05722282 | 1.22018028 | 0.00049849 |
| MIR4653 | 1.11393946 | 1.00227381 | 1.23804605 | 0.04527334 |
| TOR4A | 1.10402025 | 1.04983321 | 1.16100416 | 0.00011626 |
| SPRED1 | 1.18033527 | 1.07580165 | 1.29502624 | 0.00045787 |
| HSD17B6 | 0.99847436 | 0.99701762 | 0.99993322 | 0.0404017 |
| SOX12 | 1.03825032 | 1.01202534 | 1.06515487 | 0.00403078 |
| COLCA2 | 1.10413024 | 1.04021308 | 1.17197487 | 0.0011308 |
| HPSE | 1.27156145 | 1.05186067 | 1.53715085 | 0.0130503 |
| CDC6 | 1.09696068 | 1.04831077 | 1.14786833 | 6.38E-05 |
| PRR7 | 1.12901304 | 1.03728631 | 1.22885112 | 0.00500492 |
| STK17A | 1.05807104 | 1.00590398 | 1.11294354 | 0.02865829 |
| MDK | 1.00147291 | 1.00009492 | 1.00285279 | 0.03616513 |
| TM4SF1 | 1.00294001 | 1.00019839 | 1.00568914 | 0.03555322 |
| GLYAT | 0.9914269 | 0.9836373 | 0.99927818 | 0.03240462 |
| EPPK1 | 1.15459088 | 1.02798922 | 1.29678413 | 0.0152742 |
| OPN1SW | 1.0790374 | 1.02067497 | 1.140737 | 0.00733409 |
| CIP2A | 1.49719657 | 1.31294039 | 1.707311 | 1.71E-09 |
| AC008549.1 | 0.98397298 | 0.97145157 | 0.99665579 | 0.01341293 |
| TCF3 | 1.09641117 | 1.05366827 | 1.14088798 | 5.71E-06 |
| PRMT1 | 1.01681092 | 1.00216361 | 1.03167231 | 0.02432835 |
| HAO1 | 0.99562196 | 0.99270892 | 0.99854355 | 0.00333669 |
| SKA1 | 1.10321004 | 1.05387902 | 1.15485019 | 2.57E-05 |
| SHCBP1 | 1.21437381 | 1.1271922 | 1.3082984 | 3.22E-07 |
| MMP1 | 1.06305986 | 1.0327972 | 1.09420928 | 3.32E-05 |
| PACSIN1 | 1.14215009 | 1.01409057 | 1.28638098 | 0.02848261 |
| PON3 | 0.99140074 | 0.9833444 | 0.99952309 | 0.03802825 |
| CYP2C9 | 0.99671028 | 0.99493941 | 0.99848431 | 0.0002815 |
| LPA | 0.94315265 | 0.8949416 | 0.99396086 | 0.0287984 |
| FCGR3A | 1.0097559 | 1.003097 | 1.01645899 | 0.00402793 |
| HPX | 0.99933028 | 0.99890383 | 0.99975693 | 0.00209617 |
| TMEM158 | 1.01324993 | 1.00110419 | 1.02554301 | 0.03240928 |
| RAB3IL1 | 1.05814939 | 1.02393615 | 1.09350582 | 0.00075026 |
| GLIS2 | 1.04821188 | 1.01814227 | 1.07916955 | 0.00152077 |
| BAAT | 0.99840986 | 0.99698438 | 0.99983738 | 0.02903114 |
| TEAD4 | 1.07231667 | 1.02044737 | 1.12682248 | 0.00577804 |
| CENPK | 1.36953445 | 1.12910833 | 1.6611556 | 0.00140888 |
| PRAME | 1.02406988 | 1.00859856 | 1.03977853 | 0.00219643 |
| PLP2 | 1.00474212 | 1.0014811 | 1.00801377 | 0.0043411 |
| MMD | 1.03349343 | 1.00983013 | 1.05771123 | 0.0053083 |
| NKD2 | 1.08578099 | 1.00573398 | 1.172199 | 0.03517875 |
| MCM10 | 1.51667422 | 1.32101789 | 1.74130927 | 3.41E-09 |
| F13B | 0.99386677 | 0.98842186 | 0.99934167 | 0.02816962 |
| FCER1G | 1.00456893 | 1.00212761 | 1.00701619 | 0.00024069 |
| RABL6 | 1.06146984 | 1.01231664 | 1.11300967 | 0.01366317 |
| STK39 | 1.03247708 | 1.00407418 | 1.06168344 | 0.02472683 |
| ZNF83 | 1.08373923 | 1.02300104 | 1.14808361 | 0.00628122 |
| SLC1A5 | 1.01240851 | 1.00608198 | 1.01877482 | 0.00011534 |
| CDC20 | 1.02492858 | 1.01653288 | 1.03339363 | 4.43E-09 |
| OIP5 | 1.12065489 | 1.04409023 | 1.20283414 | 0.00160537 |
| MYLK-AS1 | 1.24027389 | 1.01712716 | 1.51237661 | 0.03335614 |
| FCGR2A | 1.06653835 | 1.02410581 | 1.11072903 | 0.00187142 |
| H2AX | 1.01898256 | 1.00905741 | 1.02900533 | 0.00016624 |
| RCC2 | 1.03388026 | 1.01836529 | 1.04963161 | 1.57E-05 |
| AC124798.1 | 1.21630961 | 1.08988241 | 1.35740246 | 0.0004705 |
| SPINK4 | 1.03486378 | 1.00985139 | 1.06049568 | 0.00604603 |
| NCK2 | 1.01833274 | 1.0038176 | 1.03305776 | 0.01313241 |
| FOLH1B | 0.85283447 | 0.73780339 | 0.98580006 | 0.03128494 |
| MIR4292 | 1.12801941 | 1.0627182 | 1.1973332 | 7.52E-05 |
| BAK1 | 1.04556833 | 1.01727008 | 1.07465379 | 0.00145718 |
| SLC25A24 | 1.19327509 | 1.10001448 | 1.29444245 | 2.08E-05 |
| FMO4 | 0.97037036 | 0.94536909 | 0.99603281 | 0.02391806 |
| EHHADH | 0.99398052 | 0.98838879 | 0.99960388 | 0.03593941 |
| RFC4 | 1.09079886 | 1.05037572 | 1.13277767 | 6.46E-06 |
| NRSN2 | 1.02878653 | 1.00834023 | 1.04964743 | 0.00559037 |
| AC022415.1 | 1.38485841 | 1.03261344 | 1.85726113 | 0.02968459 |
| RGS1 | 1.01773397 | 1.00109252 | 1.03465207 | 0.03663813 |
| SUZ12P1 | 1.33336376 | 1.08010589 | 1.64600428 | 0.00742926 |
| DAGLA | 1.24106043 | 1.07880733 | 1.42771648 | 0.00251862 |
| TCEAL9 | 1.00925903 | 1.00272043 | 1.01584027 | 0.00544952 |
| ANKRD13B | 1.31969714 | 1.14848673 | 1.51643069 | 9.13E-05 |
| SGO1 | 1.41904603 | 1.22217392 | 1.647631 | 4.37E-06 |
| PYCR1 | 1.02005806 | 1.00904811 | 1.03118815 | 0.00033481 |
| SNRPB | 1.00394099 | 1.00191872 | 1.00596734 | 0.00013172 |
| PDLIM7 | 1.038787 | 1.00917521 | 1.06926769 | 0.00991031 |
| TTC36 | 0.99070113 | 0.98151725 | 0.99997093 | 0.04928921 |
| BUB1B | 1.16169263 | 1.09091655 | 1.23706049 | 2.97E-06 |
| KCTD17 | 1.02594457 | 1.0107921 | 1.04132418 | 0.00074107 |
| HELLS | 1.14871327 | 1.02417859 | 1.28839071 | 0.01788345 |
| MCM7 | 1.00510852 | 1.00056662 | 1.00967103 | 0.02744684 |
| CDCA7 | 1.09791971 | 1.04004173 | 1.15901859 | 0.00072263 |
| BICC1 | 1.01703793 | 1.00313626 | 1.03113226 | 0.01613304 |
| TRIM71 | 1.07253265 | 1.00229177 | 1.14769604 | 0.04274392 |
| TREM2 | 1.01834286 | 1.00317485 | 1.03374021 | 0.01759856 |
| RNF145 | 1.06213639 | 1.03203133 | 1.09311964 | 3.97E-05 |
| HSPA12A | 1.2786567 | 1.03068812 | 1.58628292 | 0.02543259 |
| DCLRE1C | 1.66945399 | 1.25436971 | 2.22189408 | 0.0004417 |
| LMNB2 | 1.03768308 | 1.02100964 | 1.0546288 | 7.61E-06 |
| E2F2 | 1.66128338 | 1.31009788 | 2.10660783 | 2.80E-05 |
| YJEFN3 | 1.21638809 | 1.05273381 | 1.40548349 | 0.00788317 |
| PHLDA2 | 1.01113451 | 1.00183811 | 1.02051719 | 0.01879131 |
| FUT2 | 1.08580743 | 1.00011557 | 1.17884153 | 0.0496788 |
| RAD51 | 1.29201517 | 1.13821069 | 1.466603 | 7.44E-05 |
| PAQR5 | 1.03196235 | 1.00277814 | 1.06199592 | 0.03159446 |
| KRBA1 | 1.15708854 | 1.01871052 | 1.31426334 | 0.0247539 |
| AC016394.1 | 1.32266263 | 1.12301034 | 1.55780973 | 0.00080947 |
| TTK | 1.32903211 | 1.21191502 | 1.45746716 | 1.51E-09 |
| ARMCX2 | 1.05296107 | 1.00268445 | 1.10575866 | 0.03870026 |
| FCHSD1 | 1.25222522 | 1.01238107 | 1.54889109 | 0.0381355 |
| ZNF682 | 1.22712937 | 1.01946732 | 1.47709148 | 0.03048055 |
| PRKCD | 1.07416782 | 1.04050156 | 1.10892337 | 1.06E-05 |
| SNHG12 | 1.13253467 | 1.03488474 | 1.23939867 | 0.00682388 |
| ADHFE1 | 0.95397978 | 0.92078122 | 0.9883753 | 0.00913437 |
| C15orf39 | 1.06258722 | 1.00815489 | 1.11995845 | 0.0236552 |
| SORD | 0.98529421 | 0.97642694 | 0.99424201 | 0.00131854 |
| CENPE | 1.60564056 | 1.33387944 | 1.93276959 | 5.59E-07 |
| KIF4A | 1.08125457 | 1.04328078 | 1.12061054 | 1.85E-05 |
| SIGLEC9 | 1.22037811 | 1.07940123 | 1.37976749 | 0.00147319 |
| GIT1 | 1.10729676 | 1.06395996 | 1.15239873 | 5.63E-07 |
| RTL8B | 1.06052942 | 1.00597714 | 1.11803997 | 0.02917299 |
| CKAP4 | 1.00619041 | 1.00119047 | 1.01121533 | 0.0151795 |
| MTFR2 | 1.54220717 | 1.28637919 | 1.84891281 | 2.85E-06 |
| SNHG3 | 1.0687667 | 1.03912396 | 1.09925505 | 3.58E-06 |
| CDK16 | 1.04842814 | 1.02045224 | 1.077171 | 0.00060999 |
| A1BG | 0.98048047 | 0.96976753 | 0.99131175 | 0.00043692 |
| GNAZ | 1.04527174 | 1.01108302 | 1.08061652 | 0.00906538 |
| GINS4 | 1.26636468 | 1.04797185 | 1.53026963 | 0.01448028 |
| SLC16A3 | 1.04811928 | 1.02325217 | 1.07359071 | 0.00012494 |
| RASSF3 | 1.03620529 | 1.0098404 | 1.06325852 | 0.00683782 |
| PLEKHG2 | 1.10302925 | 1.00354938 | 1.21237035 | 0.04200851 |
| ALDOA | 1.00247957 | 1.00075343 | 1.00420869 | 0.00485482 |
| OGFRL1 | 1.19410378 | 1.06744843 | 1.33578709 | 0.00192922 |
| ASNS | 1.05258651 | 1.01256693 | 1.09418777 | 0.00955747 |
| CSF1 | 1.02811117 | 1.014033 | 1.04238479 | 8.12E-05 |
| CDC45 | 1.1251178 | 1.0657502 | 1.18779247 | 2.02E-05 |
| PTTG1 | 1.04021919 | 1.0246531 | 1.05602175 | 2.96E-07 |
| ADORA1 | 1.07724541 | 1.02920429 | 1.12752899 | 0.00139031 |
| ZNF816 | 1.32798021 | 1.11890982 | 1.5761158 | 0.0011726 |
| CPVL | 1.02931096 | 1.01420467 | 1.04464225 | 0.00012827 |
| ARHGAP11A | 1.15777417 | 1.05898879 | 1.26577453 | 0.00128401 |
| POLD1 | 1.10923699 | 1.05812674 | 1.16281598 | 1.65E-05 |
| RUSC1 | 1.10617134 | 1.05694738 | 1.15768774 | 1.39E-05 |
| IGSF3 | 1.06907752 | 1.03492687 | 1.10435507 | 5.52E-05 |
| HROB | 1.24142307 | 1.07847553 | 1.42899046 | 0.00259287 |
| HMGA1 | 1.0063107 | 1.00300331 | 1.009629 | 0.00018015 |
| CDCA4 | 1.14413399 | 1.09154633 | 1.19925518 | 2.04E-08 |
| AL627309.6 | 1.18355846 | 1.07366834 | 1.30469585 | 0.00069978 |
| GAL3ST4 | 1.10403885 | 1.02316345 | 1.19130701 | 0.01077465 |
| SLCO1B1 | 0.99558358 | 0.99143848 | 0.99974601 | 0.03759036 |
| PCLAF | 1.09350881 | 1.04368947 | 1.14570623 | 0.00017171 |
| RP9P | 1.11071782 | 1.02832829 | 1.19970838 | 0.00757733 |
| RAI1 | 1.1436903 | 1.03284953 | 1.26642601 | 0.00983986 |
| IYD | 0.85500088 | 0.76716822 | 0.95288944 | 0.00461842 |
| STMN1 | 1.02302673 | 1.01280525 | 1.03335137 | 8.85E-06 |
| MX2 | 1.12348491 | 1.00054927 | 1.26152543 | 0.04892426 |
| ZBTB12 | 1.1160165 | 1.04098425 | 1.19645694 | 0.00199423 |
| EDA | 1.13280668 | 1.00237259 | 1.28021357 | 0.04572394 |
| PRC1 | 1.06835455 | 1.02769052 | 1.11062758 | 0.00083922 |
| FHL3 | 1.0701011 | 1.03867218 | 1.10248102 | 8.40E-06 |
| SUSD1 | 1.17256322 | 1.0022691 | 1.37179179 | 0.04678158 |
| ANO1 | 0.98170093 | 0.96716634 | 0.99645395 | 0.01523529 |
| SLC52A2 | 1.02660437 | 1.01404097 | 1.03932342 | 2.92E-05 |
| SOAT2 | 1.02008406 | 1.00716769 | 1.03316607 | 0.00222462 |
| KCNJ8 | 0.98460327 | 0.97010738 | 0.99931577 | 0.04032426 |
| PTPRE | 1.18061562 | 1.03665865 | 1.34456338 | 0.01232761 |
| GINS1 | 1.16917073 | 1.10403942 | 1.23814438 | 9.08E-08 |
| CYP26B1 | 1.10556803 | 1.05438125 | 1.15923977 | 3.33E-05 |
| PFN2 | 1.02364542 | 1.01201392 | 1.03541061 | 6.12E-05 |
| CENPL | 1.41778498 | 1.22928702 | 1.63518706 | 1.62E-06 |
| SOX4 | 1.02554888 | 1.01111459 | 1.04018922 | 0.00048606 |
| DLGAP5 | 1.15114583 | 1.09699802 | 1.20796637 | 1.03E-08 |
| AC099560.2 | 1.08270922 | 1.01145429 | 1.15898392 | 0.02214556 |
| JPT1 | 1.01015357 | 1.00395643 | 1.01638897 | 0.00129269 |
| CKS2 | 1.01291096 | 1.00788182 | 1.0179652 | 4.38E-07 |
| PLAC8 | 1.08689448 | 1.00468419 | 1.17583179 | 0.03785514 |
| OTC | 0.99538821 | 0.99166814 | 0.99912223 | 0.01553602 |
| EZH2 | 1.1975444 | 1.12910775 | 1.27012909 | 1.92E-09 |
| ZNF883 | 1.17313159 | 1.01843024 | 1.35133234 | 0.02689235 |
| B3GALNT1 | 1.25495029 | 1.10719983 | 1.42241732 | 0.00038035 |
| UNC119 | 1.06674697 | 1.00825665 | 1.12863039 | 0.02471944 |
| DEPDC1 | 1.26995423 | 1.14605161 | 1.40725227 | 5.05E-06 |
| NUMBL | 1.11699255 | 1.00416057 | 1.24250284 | 0.04171196 |
| LPCAT4 | 1.09054847 | 1.03265607 | 1.15168642 | 0.0018419 |
| ADH1B | 0.99922001 | 0.99844767 | 0.99999295 | 0.04794627 |
| HSPA7 | 1.04195332 | 1.01752839 | 1.06696455 | 0.00068444 |
| CD24 | 1.00353595 | 1.00150657 | 1.00556945 | 0.00063183 |
| LINC00189 | 1.0797212 | 1.01226019 | 1.15167808 | 0.01979818 |
| PLXNA1 | 1.14035061 | 1.06759591 | 1.21806341 | 9.44E-05 |
| YBX3 | 1.03413572 | 1.00274542 | 1.06650866 | 0.03281887 |
| IGF2BP3 | 1.31739194 | 1.15717125 | 1.49979663 | 3.10E-05 |
| PSRC1 | 1.22150766 | 1.15283155 | 1.29427492 | 1.23E-11 |
| SRC | 1.04941482 | 1.0177333 | 1.08208256 | 0.0020435 |
| FBP1 | 0.99850775 | 0.99701919 | 0.99999853 | 0.04977408 |
| SEMA6A | 1.06199966 | 1.02525228 | 1.10006414 | 0.00081402 |
| C8B | 0.99666312 | 0.99413214 | 0.99920055 | 0.00998207 |
| CD7 | 1.01284558 | 1.00554778 | 1.02019635 | 0.00054125 |
| NIBAN2 | 1.01184875 | 1.00280936 | 1.02096961 | 0.01009075 |
| INSIG1 | 0.99838573 | 0.99697392 | 0.99979955 | 0.02524554 |
| XRCC2 | 1.74528828 | 1.35349267 | 2.25049699 | 1.76E-05 |
| IBSP | 1.01675129 | 1.00173844 | 1.03198913 | 0.02861013 |
| AGXT2 | 0.9775431 | 0.9568137 | 0.99872159 | 0.03780705 |
| PPP1R18 | 1.0254223 | 1.0065824 | 1.04461482 | 0.00796838 |
| LINC02331 | 1.10442811 | 1.02385027 | 1.19134749 | 0.01017675 |
| RAB27B | 1.16862864 | 1.01396844 | 1.34687909 | 0.03143793 |
| WDR54 | 1.08515949 | 1.01618772 | 1.15881259 | 0.01471833 |
| MAD2L1 | 1.18554733 | 1.0869328 | 1.29310889 | 0.0001224 |
| AC068987.3 | 1.1217051 | 1.05242204 | 1.19554919 | 0.00041448 |
| CDK1 | 1.06085095 | 1.03429385 | 1.08808995 | 4.95E-06 |
| NCDN | 1.07677273 | 1.03070633 | 1.12489802 | 0.00091417 |
| CYTOR | 1.0349804 | 1.01152526 | 1.0589794 | 0.0032847 |
| SH3BGRL3 | 1.00312981 | 1.00056284 | 1.00570336 | 0.01683084 |
| TMSB4XP8 | 1.01645261 | 1.00073298 | 1.03241915 | 0.04015957 |
| ARHGEF2 | 1.08332387 | 1.03745337 | 1.13122251 | 0.00028823 |
| FLNA | 1.00581382 | 1.00211898 | 1.00952228 | 0.00202008 |
| BIRC5 | 1.0271599 | 1.01421971 | 1.04026519 | 3.43E-05 |
| ZNF532 | 1.24946757 | 1.08567729 | 1.43796801 | 0.00189256 |
| NEK2 | 1.09101746 | 1.04554815 | 1.13846417 | 6.05E-05 |
| BLMH | 1.03785118 | 1.00993904 | 1.06653473 | 0.00756304 |
| UPB1 | 0.98454584 | 0.97434625 | 0.9948522 | 0.00337511 |
| SGPP2 | 1.04048167 | 1.01039123 | 1.07146823 | 0.00803988 |
| ADGRE5 | 1.02931563 | 1.01157935 | 1.04736289 | 0.00112129 |
| ST6GALNAC4 | 1.09770929 | 1.04631432 | 1.15162879 | 0.0001387 |
| SLC25A36 | 1.16038628 | 1.01186792 | 1.33070362 | 0.03327031 |
| IGF2BP1 | 1.05442556 | 1.00653401 | 1.10459582 | 0.02544537 |
| OSBPL7 | 1.29884534 | 1.02101908 | 1.65227002 | 0.03322436 |
| PFKFB3 | 1.01003362 | 1.00206804 | 1.01806252 | 0.01345994 |
| GBP5 | 1.0158213 | 1.00080229 | 1.03106571 | 0.03887684 |
| GFRA1 | 0.93525017 | 0.88621108 | 0.98700287 | 0.01484906 |
| HID1 | 1.04596123 | 1.00549839 | 1.08805235 | 0.02559067 |
| TPX2 | 1.04511452 | 1.03039324 | 1.06004613 | 1.08E-09 |
| SLC38A1 | 1.04335244 | 1.0165686 | 1.07084197 | 0.00138179 |
| RBL1 | 1.4336687 | 1.2224013 | 1.68144942 | 9.47E-06 |
| ZIC5 | 1.27905514 | 1.09048511 | 1.50023329 | 0.00249123 |
| CDH16 | 1.05208138 | 1.0070526 | 1.09912356 | 0.02291439 |
| ACNATP | 0.71722617 | 0.53105518 | 0.96866274 | 0.03018838 |
| KIF15 | 1.37600545 | 1.18855703 | 1.59301653 | 1.94E-05 |
| SDC3 | 1.03425088 | 1.01417542 | 1.05472373 | 0.00075875 |
| KIAA1841 | 2.56582712 | 1.82787847 | 3.6016994 | 5.15E-08 |
| AC137723.1 | 0.87253578 | 0.76799188 | 0.99131087 | 0.03626101 |
| UHRF1 | 1.13827346 | 1.06184836 | 1.22019917 | 0.00025991 |
| DNASE1L3 | 0.9474953 | 0.91262099 | 0.98370228 | 0.0048209 |
| CDCA5 | 1.08564408 | 1.04592438 | 1.12687215 | 1.55E-05 |
| ABAT | 0.9859651 | 0.97688455 | 0.99513006 | 0.00275265 |
| ZNF213 | 1.21987985 | 1.05853031 | 1.40582356 | 0.00603658 |
| GSDME | 1.20783557 | 1.09406175 | 1.33344097 | 0.00018335 |
| AC026401.3 | 1.08673115 | 1.03922314 | 1.13641098 | 0.00026544 |
| C1QB | 1.0012087 | 1.00001197 | 1.00240686 | 0.04775155 |
| SPINDOC | 1.10878993 | 1.06513246 | 1.15423682 | 4.69E-07 |
| DSCC1 | 1.16187422 | 1.08050494 | 1.24937116 | 5.12E-05 |
| CTSC | 1.02224457 | 1.0049712 | 1.03981483 | 0.01139719 |
| MYL6B | 1.02305286 | 1.00291319 | 1.04359695 | 0.02465755 |
| PNMA1 | 1.05014762 | 1.02108113 | 1.08004152 | 0.00063385 |
| SPC25 | 1.20860917 | 1.12678424 | 1.29637608 | 1.18E-07 |
| AC083809.1 | 1.01122361 | 1.00405332 | 1.0184451 | 0.00211123 |
| RNF144A | 1.11016679 | 1.03268338 | 1.19346385 | 0.00463735 |
| MEP1A | 1.01139954 | 1.00307241 | 1.0197958 | 0.00720467 |
| TRIP13 | 1.14236627 | 1.0911043 | 1.19603661 | 1.33E-08 |
| SALL2 | 1.13063059 | 1.01389745 | 1.26080357 | 0.02723049 |
| SPIB | 1.01624183 | 1.0004368 | 1.03229655 | 0.04394974 |
| APOL5 | 0.85776855 | 0.74093632 | 0.9930231 | 0.04000632 |
| COL16A1 | 1.05310007 | 1.00416301 | 1.10442205 | 0.03308249 |
| ADRA1A | 0.87079236 | 0.75942107 | 0.99849658 | 0.04753382 |
| SLC22A15 | 1.11394182 | 1.00036489 | 1.24041376 | 0.04922784 |
| PTGES | 1.02954994 | 1.00965569 | 1.04983618 | 0.00344237 |
| CDCA8 | 1.11302552 | 1.07833728 | 1.14882961 | 3.39E-11 |
| PSMC3IP | 1.38789379 | 1.21367693 | 1.58711855 | 1.67E-06 |
| STK26 | 1.06875556 | 1.01231367 | 1.12834438 | 0.0163027 |
| ARHGAP4 | 1.02431851 | 1.00029131 | 1.04892285 | 0.04725423 |
| CTSV | 1.01368578 | 1.00465077 | 1.02280205 | 0.00292286 |
| SEC14L2 | 0.99101542 | 0.98390114 | 0.99818114 | 0.01407988 |
| ESR1 | 0.7417899 | 0.56064768 | 0.98145818 | 0.0365294 |
| S100A2 | 1.09445854 | 1.01115617 | 1.18462363 | 0.02544116 |
| FZD7 | 1.07232747 | 1.03139082 | 1.11488892 | 0.00043755 |
| CENPH | 1.17446589 | 1.08001605 | 1.27717558 | 0.00017024 |
| HMCN2 | 0.76782009 | 0.60377013 | 0.97644395 | 0.03121323 |
| DDX39A | 1.01693607 | 1.00108591 | 1.03303719 | 0.03613756 |
| PNMA3 | 1.04672521 | 1.01822629 | 1.07602179 | 0.0011852 |
| C5orf46 | 1.16243844 | 1.07299745 | 1.25933489 | 0.00022894 |
| KIF18B | 1.18232574 | 1.10551568 | 1.26447248 | 1.02E-06 |
| FAM241B | 1.05842759 | 1.02742637 | 1.09036423 | 0.00018121 |
| NCAPG | 1.17496603 | 1.115664 | 1.23742022 | 1.05E-09 |
| CCNB2 | 1.0755906 | 1.03792411 | 1.11462402 | 6.16E-05 |
| CD300C | 1.28673074 | 1.03023042 | 1.60709291 | 0.02624801 |
| STX3 | 1.08613613 | 1.04259441 | 1.13149628 | 7.55E-05 |
| TMEM130 | 1.02691753 | 1.00796092 | 1.04623065 | 0.00520485 |
| TBC1D30 | 1.49893404 | 1.1768028 | 1.90924364 | 0.00104267 |
| RTL8A | 1.01932607 | 1.00980848 | 1.02893337 | 6.35E-05 |
| SLC2A6 | 1.04785583 | 1.00072813 | 1.09720293 | 0.0464848 |
| ACSM2A | 0.98672707 | 0.97667718 | 0.99688038 | 0.01052278 |
| CLIC1 | 1.00290322 | 1.00110863 | 1.00470102 | 0.00151127 |
| MAMSTR | 1.1866803 | 1.07654324 | 1.30808506 | 0.00057305 |
| OTULINL | 1.20016719 | 1.03601826 | 1.39032422 | 0.01503611 |
| FANCD2 | 1.32647088 | 1.1402282 | 1.54313409 | 0.00025222 |
| FBLL1 | 1.07393061 | 1.0341454 | 1.11524642 | 0.00021291 |
| FBXO5 | 1.38017481 | 1.22362104 | 1.55675853 | 1.56E-07 |
| PLGLA | 0.88568955 | 0.81371008 | 0.96403621 | 0.00500241 |
| SNHG1 | 1.04176473 | 1.01463397 | 1.06962096 | 0.00237353 |
| RAD51AP1 | 1.17265022 | 1.08675888 | 1.26532994 | 4.07E-05 |
| NCAPH | 1.13460048 | 1.07800441 | 1.19416788 | 1.32E-06 |
| DMGDH | 0.97734697 | 0.96268631 | 0.9922309 | 0.00296467 |
| EFNA5 | 1.08314271 | 1.01405131 | 1.15694159 | 0.01755472 |
| ZDHHC13 | 1.24707502 | 1.03361439 | 1.50461925 | 0.02115967 |
| CDCA3 | 1.27205834 | 1.1634546 | 1.39079979 | 1.26E-07 |
| AC099850.3 | 1.144192 | 1.09233965 | 1.19850573 | 1.25E-08 |
| IGF1R | 1.15761319 | 1.01968304 | 1.31420083 | 0.02375373 |
| IL4I1 | 1.03272911 | 1.00360756 | 1.06269568 | 0.02733414 |
| PLK1 | 1.12842683 | 1.08275368 | 1.17602659 | 9.95E-09 |
| CBX2 | 1.20487674 | 1.1271011 | 1.28801928 | 4.39E-08 |
| ARHGAP39 | 1.18569337 | 1.08426637 | 1.2966083 | 0.00018908 |
| H1-12P | 1.1669116 | 1.05456989 | 1.29122091 | 0.00280146 |
| ABCC1 | 1.07302235 | 1.0360814 | 1.11128041 | 8.05E-05 |
| MAFG-DT | 1.07176173 | 1.02644879 | 1.11907503 | 0.00166442 |
| LHFPL2 | 1.14865149 | 1.0757135 | 1.22653498 | 3.47E-05 |
| ZNF239 | 1.34606867 | 1.21037645 | 1.49697301 | 4.21E-08 |
| EFNA3 | 1.12692089 | 1.05431559 | 1.20452614 | 0.00043715 |
| LGALS9 | 1.02283735 | 1.00030447 | 1.0458778 | 0.04695025 |
| CXCL5 | 1.00869373 | 1.00122738 | 1.01621576 | 0.02239749 |
| SNX25P1 | 1.28992779 | 1.05435705 | 1.57813116 | 0.01334518 |
| LRRC1 | 1.08658667 | 1.01876195 | 1.15892686 | 0.01156285 |
| TTYH3 | 1.01332194 | 1.00533153 | 1.02137586 | 0.00105134 |
| CALML3 | 1.01767637 | 1.00188656 | 1.03371502 | 0.0280774 |
| IGF2BP2 | 1.02364866 | 1.000388 | 1.04745017 | 0.04625679 |
| WDR62 | 1.22649135 | 1.05920865 | 1.42019332 | 0.00635619 |
| PLEKHB2 | 1.02742513 | 1.00421271 | 1.05117411 | 0.02031272 |
| CDCA7L | 1.13849116 | 1.08671562 | 1.19273348 | 4.71E-08 |
| LAMB1 | 1.02047114 | 1.00927889 | 1.03178751 | 0.00031649 |
| MELK | 1.14369154 | 1.08393725 | 1.20673991 | 9.40E-07 |
| TLDC2 | 1.09290517 | 1.01969011 | 1.17137717 | 0.0120352 |
| CYBB | 1.01954212 | 1.00102738 | 1.0383993 | 0.03847289 |
| MTND4P20 | 0.98123508 | 0.96729115 | 0.99538001 | 0.00948406 |
| SFXN3 | 1.0352383 | 1.00513511 | 1.06624307 | 0.02143955 |
| PDE7A | 1.15233709 | 1.04498879 | 1.27071293 | 0.00448319 |
| PIK3CD-AS2 | 1.23710789 | 1.13143698 | 1.35264797 | 3.00E-06 |
| MIR210HG | 1.14139748 | 1.06848039 | 1.21929071 | 8.62E-05 |
| TPM3P9 | 1.10553935 | 1.00531658 | 1.2157536 | 0.03851555 |
| MAGEA6 | 1.02088757 | 1.00733003 | 1.03462757 | 0.00244023 |
| CDKN3 | 1.04628967 | 1.0211655 | 1.07203197 | 0.00026336 |
| B3GNT9 | 1.15858413 | 1.06432623 | 1.26118961 | 0.00067411 |
| SAPCD2 | 1.21725075 | 1.13302322 | 1.30773964 | 7.72E-08 |
| FOXM1 | 1.06618949 | 1.03789018 | 1.09526041 | 3.02E-06 |
| NEIL3 | 1.45841877 | 1.28862435 | 1.650586 | 2.30E-09 |
| RCAN3 | 1.2041606 | 1.04386687 | 1.38906865 | 0.01080304 |
| ADH1C | 0.99874834 | 0.99797495 | 0.99952233 | 0.00153074 |
| LAIR1 | 1.07811671 | 1.01374665 | 1.14657409 | 0.01663699 |
| AC239859.5 | 1.10047142 | 1.03553002 | 1.16948551 | 0.0020357 |
| TROAP | 1.11590782 | 1.06065108 | 1.17404328 | 2.31E-05 |
| DTYMK | 1.03689125 | 1.0207716 | 1.05326545 | 5.85E-06 |
| PFKFB4 | 1.25708865 | 1.14496643 | 1.38019058 | 1.59E-06 |
| STIL | 1.45949286 | 1.20247565 | 1.77144493 | 0.00013046 |
| TRNP1 | 1.00990734 | 1.00411982 | 1.01572821 | 0.00077366 |
| GYS2 | 0.9815461 | 0.96837844 | 0.9948928 | 0.00687151 |
| ENTPD2 | 1.09398173 | 1.0528834 | 1.13668429 | 4.27E-06 |
| AC009005.1 | 1.1665113 | 1.074685 | 1.26618368 | 0.00023161 |
| BICDL1 | 1.07500485 | 1.0187464 | 1.13437007 | 0.00835976 |
| PLAUR | 1.0233612 | 1.00349602 | 1.04361963 | 0.02094891 |
| F11-AS1 | 0.85238244 | 0.76331513 | 0.95184254 | 0.00456149 |
| SH3BP1 | 1.10603219 | 1.01255673 | 1.20813696 | 0.02528997 |
| CTHRC1 | 1.0206259 | 1.00564971 | 1.03582512 | 0.00679057 |
| CENPF | 1.11442072 | 1.06011762 | 1.17150542 | 2.13E-05 |
| CYP2C8 | 0.99817194 | 0.99644383 | 0.99990305 | 0.03848657 |
| CLCF1 | 1.08544083 | 1.02448592 | 1.15002244 | 0.00543033 |
| ACADL | 0.90276343 | 0.83380738 | 0.97742217 | 0.01162704 |
| AC010761.1 | 1.42812399 | 1.14050542 | 1.7882757 | 0.00189787 |
| UBE2S | 1.04242141 | 1.02196739 | 1.06328481 | 3.97E-05 |
| NDRG3 | 1.03185984 | 1.01525334 | 1.04873797 | 0.00015145 |
| CDC25A | 1.2349716 | 1.11363325 | 1.36953064 | 6.34E-05 |
| AC040970.1 | 1.20165938 | 1.06755527 | 1.35260936 | 0.00234448 |
| CPEB3 | 0.81802595 | 0.68887098 | 0.97139591 | 0.02196504 |
| ORC1 | 1.26504797 | 1.1588202 | 1.38101352 | 1.49E-07 |
| FRAS1 | 1.10275599 | 1.02082712 | 1.19126025 | 0.01301716 |
| ANLN | 1.13578834 | 1.08860722 | 1.18501432 | 4.06E-09 |
| TUBA3C | 1.01801631 | 1.00754101 | 1.02860052 | 0.0007155 |
| S100A11 | 1.0014082 | 1.00068111 | 1.00213581 | 0.0001463 |
| MFAP2 | 1.04996568 | 1.01054335 | 1.09092592 | 0.01252083 |
| SLC7A1 | 1.14735535 | 1.06333548 | 1.23801407 | 0.00039611 |
| ACSM2B | 0.99093989 | 0.98309238 | 0.99885003 | 0.02485764 |
| GAS2L3 | 1.33376256 | 1.1869831 | 1.49869241 | 1.29E-06 |
| ZNF529-AS1 | 1.28356121 | 1.03026559 | 1.59913074 | 0.02602668 |
| CDR2L | 1.04188442 | 1.00498614 | 1.08013744 | 0.02572608 |
| AC106900.1 | 1.124063 | 1.00783921 | 1.2536897 | 0.03571195 |
| NFE2L3 | 1.05563983 | 1.01196687 | 1.10119756 | 0.01201199 |
| SNHG14 | 1.27064733 | 1.07243038 | 1.50550065 | 0.0056388 |
| ADH4 | 0.99870781 | 0.99794049 | 0.99947572 | 0.00097638 |
| CEP55 | 1.11160554 | 1.06551027 | 1.15969496 | 9.76E-07 |
| TMEM201 | 1.30480606 | 1.15000845 | 1.4804403 | 3.64E-05 |
| EPO | 1.03183089 | 1.015774 | 1.0481416 | 9.01E-05 |
| ZNF160 | 1.21816701 | 1.03348007 | 1.43585822 | 0.0186458 |
| GTSE1 | 1.19121553 | 1.11108073 | 1.27712991 | 8.46E-07 |
| DQX1 | 1.08616117 | 1.01213448 | 1.16560212 | 0.0217409 |
| PLEKHA2 | 1.09230298 | 1.02151671 | 1.1679944 | 0.00980242 |
| GLDN | 1.077826 | 1.01496539 | 1.1445798 | 0.01450677 |
| LINC00665 | 1.11885711 | 1.03881906 | 1.20506187 | 0.00302055 |
| ITGA2 | 1.10417832 | 1.01310054 | 1.20344399 | 0.02405224 |
| RBP4 | 0.99988545 | 0.99978611 | 0.99998479 | 0.02382354 |
| NFKBIE | 1.0234454 | 1.00308861 | 1.04421532 | 0.02377115 |
| MSC | 1.01007368 | 1.00538811 | 1.0147811 | 2.39E-05 |
| SYDE1 | 1.09004068 | 1.00287896 | 1.18477774 | 0.04260384 |
| FBXL19 | 1.16809565 | 1.06235574 | 1.28436022 | 0.00133002 |
| RAB38 | 1.16810348 | 1.03632957 | 1.31663302 | 0.01094999 |
| MTMR2 | 1.29410584 | 1.17090082 | 1.4302748 | 4.40E-07 |
| OSBP2 | 1.28264163 | 1.13863678 | 1.44485895 | 4.19E-05 |
| ATP1A1 | 1.00199255 | 1.00052363 | 1.00346362 | 0.00782937 |
| KRT17 | 1.02356036 | 1.01131045 | 1.03595866 | 0.00015016 |
| LAPTM4B | 1.00423533 | 1.00173756 | 1.00673932 | 0.0008801 |
| DHDH | 1.15122815 | 1.04127072 | 1.272797 | 0.00596773 |
| HJURP | 1.18105011 | 1.11916537 | 1.24635679 | 1.36E-09 |
| AC023090.1 | 1.06629438 | 1.00096018 | 1.13589304 | 0.04662257 |
| TIGD1 | 1.16680604 | 1.07737381 | 1.26366198 | 0.00014962 |
| CRABP2 | 1.02244363 | 1.00364757 | 1.0415917 | 0.01904929 |
| BARD1 | 1.5341664 | 1.24938188 | 1.8838648 | 4.40E-05 |
| PAK4 | 1.04592034 | 1.00853331 | 1.08469332 | 0.01562792 |
| SLC1A2 | 0.96761333 | 0.94146763 | 0.99448513 | 0.01849035 |
| TTR | 0.99974404 | 0.99952111 | 0.99996702 | 0.0244611 |
| HILPDA | 1.03320456 | 1.01151642 | 1.05535772 | 0.00254568 |
| CENPI | 1.55922157 | 1.29091804 | 1.88328912 | 4.02E-06 |
| AR | 0.96370481 | 0.93088474 | 0.99768201 | 0.03650708 |
| PTGS1 | 1.16337704 | 1.07239043 | 1.26208337 | 0.00027049 |
| ZC2HC1A | 1.5820105 | 1.31408205 | 1.90456693 | 1.27E-06 |
| TFR2 | 0.99770354 | 0.99557667 | 0.99983496 | 0.03472463 |
| BLM | 1.48688719 | 1.13663489 | 1.94506921 | 0.00379816 |
| MIR621 | 0.99657356 | 0.99412995 | 0.99902319 | 0.00614053 |
| C11orf49 | 1.15730575 | 1.05326401 | 1.27162476 | 0.00236828 |
| P2RY6 | 1.22475144 | 1.11676995 | 1.34317376 | 1.67E-05 |
| MELTF-AS1 | 1.14280593 | 1.02699501 | 1.27167648 | 0.01434231 |
| ITPR3 | 1.05038022 | 1.0125178 | 1.08965848 | 0.00868761 |
| SLC10A3 | 1.03450335 | 1.01112922 | 1.05841782 | 0.00362415 |
| C1orf116 | 1.05139416 | 1.00729055 | 1.09742882 | 0.02189399 |
| DTL | 1.06554357 | 1.01476964 | 1.11885797 | 0.01081751 |
| ETV4 | 1.01983769 | 1.00428699 | 1.03562919 | 0.01222366 |
| CENPW | 1.0321457 | 1.0159875 | 1.04856088 | 8.49E-05 |
| MTHFD1L | 1.16211945 | 1.07947569 | 1.25109035 | 6.56E-05 |
| RAP1GAP2 | 1.1524331 | 1.01606167 | 1.30710771 | 0.02724861 |
| CSF3R | 1.11141706 | 1.05102155 | 1.17528312 | 0.00021091 |
| VSIG4 | 1.02089211 | 1.00747243 | 1.03449054 | 0.00219371 |
| NCAPD2 | 1.08907369 | 1.0591269 | 1.11986723 | 2.00E-09 |
| KIF20A | 1.14855547 | 1.10252143 | 1.19651159 | 3.21E-11 |
| IQCD | 1.2341703 | 1.07542138 | 1.41635303 | 0.00274421 |
| PTDSS2 | 1.06077334 | 1.01768504 | 1.10568598 | 0.00529464 |
| ABCA6 | 0.95109793 | 0.90964469 | 0.99444022 | 0.02744173 |
| RAVER1 | 1.10739277 | 1.02628729 | 1.19490785 | 0.00857382 |
| HNRNPA1P16 | 1.26819031 | 1.04317608 | 1.54174036 | 0.01711976 |
| ATP1B3 | 1.02661175 | 1.01429499 | 1.03907807 | 2.00E-05 |
| TYROBP | 1.00362756 | 1.00005568 | 1.0072122 | 0.04652802 |
| SCRN1 | 1.04588287 | 1.02147041 | 1.07087878 | 0.000197 |
| S100A9 | 1.00096663 | 1.00053479 | 1.00139866 | 1.14E-05 |
| PPM1H | 1.0685166 | 1.0003652 | 1.14131091 | 0.04874416 |
| NUF2 | 1.11240072 | 1.06238117 | 1.16477532 | 5.68E-06 |
| LDHD | 0.98766533 | 0.9791978 | 0.99620609 | 0.00472483 |
| CPS1 | 0.99847195 | 0.99709394 | 0.99985185 | 0.02999001 |
| ECT2 | 1.12452886 | 1.07151999 | 1.18016013 | 1.90E-06 |
| DAB2 | 1.03444586 | 1.019553 | 1.04955627 | 4.71E-06 |
| SLC25A15 | 0.98686603 | 0.97607368 | 0.9977777 | 0.01844775 |
| LINC00205 | 1.23448534 | 1.101078 | 1.3840564 | 0.000306 |
| NCS1 | 1.07397662 | 1.02359205 | 1.12684129 | 0.00360151 |
| NCF2 | 1.04240237 | 1.0208081 | 1.06445345 | 0.00010099 |
| PTK7 | 1.06862249 | 1.03545465 | 1.10285277 | 3.70E-05 |
| KCNH2 | 1.03697409 | 1.01120157 | 1.06340348 | 0.00469201 |
| CMTM7 | 1.12597345 | 1.05156409 | 1.20564806 | 0.00067062 |
| TTLL4 | 1.04176241 | 1.01137555 | 1.07306224 | 0.00675109 |
| FBLN1 | 1.01154883 | 1.00414888 | 1.01900331 | 0.00217545 |
| PITX1 | 1.07526165 | 1.02212186 | 1.13116416 | 0.00501437 |
| RMI2 | 1.05084574 | 1.00815359 | 1.09534576 | 0.01909281 |
| HLA-DQB2 | 1.02121445 | 1.00034943 | 1.04251466 | 0.04624669 |
| MFSD10 | 1.01463688 | 1.00288594 | 1.0265255 | 0.01449207 |
| PLA2G1B | 1.01642759 | 1.00146876 | 1.03160985 | 0.03124148 |
| REEP4 | 1.01840263 | 1.00491654 | 1.03206971 | 0.00733927 |
| AP005262.1 | 1.17080895 | 1.06054224 | 1.29254031 | 0.00177999 |
| PMAIP1 | 1.18127119 | 1.02823801 | 1.35708037 | 0.01860571 |
| SOX9 | 1.01204982 | 1.00111335 | 1.02310576 | 0.03071923 |
| APOL4 | 1.09511125 | 1.01386908 | 1.18286342 | 0.02087769 |
| HOMER3 | 1.0260944 | 1.00041206 | 1.05243605 | 0.04639095 |
| MAGEA8 | 1.06162731 | 1.01192302 | 1.11377301 | 0.01450802 |
| PRKAR1B-AS2 | 1.02508905 | 1.00018216 | 1.05061618 | 0.04832747 |
| LINC02428 | 0.96439571 | 0.93243952 | 0.99744708 | 0.03497559 |
| CFHR4 | 0.98334964 | 0.97025137 | 0.99662472 | 0.01412241 |
| EGLN3 | 1.03769665 | 1.00464911 | 1.07183128 | 0.02503568 |
| LIMCH1 | 1.0661239 | 1.00882502 | 1.12667723 | 0.02310515 |
| FOXO6 | 1.1509102 | 1.01319755 | 1.3073406 | 0.03064864 |
| SLC6A19 | 1.04564911 | 1.01063841 | 1.08187264 | 0.01019952 |
| TGFB1 | 1.0096556 | 1.00244232 | 1.01692078 | 0.00861969 |
| SOGA1 | 1.26371385 | 1.09585037 | 1.45729084 | 0.00128785 |
| TKTL1 | 1.0111805 | 1.00243347 | 1.02000384 | 0.01213209 |
| DEPDC1B | 1.06990087 | 1.02100185 | 1.12114181 | 0.00464397 |
| PRMT2 | 1.06617511 | 1.00210759 | 1.13433864 | 0.04270849 |
| SLC52A3 | 1.07515932 | 1.00135409 | 1.15440438 | 0.04579718 |
| AC006504.7 | 1.39149771 | 1.15690956 | 1.67365364 | 0.0004528 |
| MPZL1 | 1.04832444 | 1.0274481 | 1.06962496 | 4.26E-06 |
| UNC5B | 1.04639733 | 1.00223208 | 1.09250881 | 0.03927454 |
| PLBD1 | 1.0268483 | 1.01117421 | 1.04276534 | 0.0007358 |
| RPL39P36 | 1.4762058 | 1.13419563 | 1.92134717 | 0.0037744 |
| NT5DC2 | 1.02412805 | 1.01250467 | 1.03588486 | 4.24E-05 |
| AL035461.2 | 1.08084975 | 1.01003397 | 1.15663057 | 0.0245293 |
| FZD1 | 1.0570479 | 1.01562601 | 1.10015917 | 0.00652461 |
| IMPDH1 | 1.05228931 | 1.02854118 | 1.07658576 | 1.21E-05 |
| TPM4 | 1.01080242 | 1.00355805 | 1.01809907 | 0.00341388 |
| AFP | 1.00015497 | 1.00000911 | 1.00030086 | 0.0373041 |
| TRAM1L1 | 1.20825421 | 1.03969245 | 1.40414431 | 0.01359757 |
| ARID3A | 1.05528591 | 1.02205821 | 1.08959385 | 0.00097863 |
| TRIM60P18 | 1.28237632 | 1.0767161 | 1.52731906 | 0.00529108 |
| IKBKE | 1.10220839 | 1.03066925 | 1.17871309 | 0.00447978 |
| C5orf34 | 1.5651717 | 1.23311636 | 1.98664338 | 0.00023111 |
| RAP1GAP | 1.01971145 | 1.01039581 | 1.02911298 | 3.06E-05 |
| KCNJ11 | 1.11659038 | 1.02745707 | 1.21345613 | 0.0093738 |
| CTAG2 | 1.01160358 | 1.00294213 | 1.02033983 | 0.00854901 |
| C8A | 0.99638125 | 0.99383453 | 0.99893449 | 0.00549639 |
| AC102953.2 | 1.45915668 | 1.0762083 | 1.97836999 | 0.01498129 |
| MAL2 | 1.01092795 | 1.00410575 | 1.01779651 | 0.00165554 |
| ZNF28 | 1.20199224 | 1.05696957 | 1.36691291 | 0.00503852 |
| CHTF18 | 1.09336589 | 1.00697257 | 1.18717134 | 0.03355212 |
| CBX6 | 1.06519186 | 1.01973079 | 1.11267964 | 0.00454023 |
| PKIB | 1.04630568 | 1.01656863 | 1.07691261 | 0.00209079 |
| PSPH | 1.02713369 | 1.00953283 | 1.04504142 | 0.00239884 |
| MYCN | 1.09214811 | 1.05490533 | 1.13070572 | 6.38E-07 |
| CACNG4 | 1.0158633 | 1.00016721 | 1.03180571 | 0.04759033 |
| PLXNA3 | 1.20180808 | 1.08764635 | 1.32795246 | 0.00030647 |
| TLCD3A | 1.06059374 | 1.00694143 | 1.11710478 | 0.02634157 |
| BMF | 1.05240643 | 1.01514705 | 1.09103335 | 0.00547964 |
| CREB3L1 | 1.01873077 | 1.00177917 | 1.03596922 | 0.03018961 |
| NDRG1 | 1.00727941 | 1.00445644 | 1.01011031 | 4.08E-07 |
| ARMCX1 | 1.10395036 | 1.04536441 | 1.16581968 | 0.00037855 |
| SPATC1L | 1.05816846 | 1.01104322 | 1.10749023 | 0.01499643 |
| LIMK1 | 1.04959251 | 1.01518276 | 1.08516859 | 0.00442744 |
| ZNF320 | 1.16555749 | 1.06028844 | 1.28127801 | 0.00151345 |
| POF1B | 1.08632123 | 1.04400589 | 1.13035169 | 4.42E-05 |
| TAT | 0.99890422 | 0.99786752 | 0.999942 | 0.03850416 |
| GNG4 | 1.04682854 | 1.02205402 | 1.07220358 | 0.00018032 |
| CHAF1B | 1.1882411 | 1.08215891 | 1.30472234 | 0.00030057 |
| MCM2 | 1.04780532 | 1.02849562 | 1.06747755 | 8.63E-07 |
| CORO2A | 1.08082442 | 1.02034062 | 1.14489358 | 0.00816185 |
| RAVER2 | 1.1493374 | 1.04269034 | 1.26689239 | 0.00508901 |
| CLIC3 | 1.05088466 | 1.01627071 | 1.08667754 | 0.00367896 |
| SFI1 | 1.20966594 | 1.04543364 | 1.3996983 | 0.01056423 |
| ZNF71 | 1.56067967 | 1.17267912 | 2.07705671 | 0.00227143 |
| EPS8L3 | 1.04037849 | 1.02242821 | 1.05864392 | 8.28E-06 |
| AJM1 | 1.14905223 | 1.00358916 | 1.31559914 | 0.04423653 |
| NAP1L1 | 1.03170958 | 1.01425789 | 1.04946154 | 0.00033523 |
| HK3 | 1.08034374 | 1.00627672 | 1.15986246 | 0.03295442 |
| TMEM147 | 1.00694661 | 1.0003561 | 1.01358055 | 0.0388066 |
| PLAGL2 | 1.21188553 | 1.09464319 | 1.34168518 | 0.00021402 |
| HRG | 0.99942601 | 0.99896925 | 0.99988297 | 0.0138243 |
| KIF11 | 1.11943695 | 1.0588332 | 1.18350945 | 7.10E-05 |
| UBE2C | 1.01577347 | 1.00945413 | 1.02213237 | 8.87E-07 |
| PDE4A | 1.1048743 | 1.01026273 | 1.20834628 | 0.0289983 |
| RAD54L | 1.26205236 | 1.12859318 | 1.41129344 | 4.48E-05 |
| TBXAS1 | 1.1182383 | 1.01126119 | 1.23653208 | 0.0293888 |
| LMNB1 | 1.05359812 | 1.03010567 | 1.07762633 | 5.68E-06 |
| MACIR | 1.20199562 | 1.11254138 | 1.29864246 | 3.12E-06 |
| KCNMB2-AS1 | 1.17419089 | 1.04446508 | 1.32002905 | 0.00718197 |
| IGHV3-74 | 0.96614142 | 0.93597908 | 0.99727576 | 0.03329247 |
| NDC80 | 1.1709178 | 1.11099652 | 1.23407092 | 3.93E-09 |
| MSI1 | 1.03953543 | 1.000575 | 1.0800129 | 0.04665062 |
| DBF4B | 1.6500113 | 1.32319917 | 2.05754156 | 8.72E-06 |
| CENPO | 1.3984067 | 1.236477 | 1.58154279 | 9.27E-08 |
| S100A8 | 1.0039577 | 1.00140529 | 1.0065166 | 0.00235632 |
| PIF1 | 1.42115803 | 1.15775369 | 1.74449036 | 0.00077798 |
| RAB7B | 1.1729851 | 1.00375261 | 1.37075015 | 0.04474098 |
| ABCC9 | 0.91030145 | 0.83053686 | 0.99772661 | 0.0445787 |
| GNA15 | 1.14424893 | 1.02879895 | 1.27265451 | 0.01302152 |
| HAVCR1 | 1.05335799 | 1.0322635 | 1.07488354 | 4.74E-07 |
| SMPDL3B | 1.10435105 | 1.00773964 | 1.21022455 | 0.03358453 |
| MOGAT2 | 0.97380944 | 0.95127031 | 0.9968826 | 0.02633143 |
| ABCA8 | 0.90940623 | 0.83768706 | 0.9872657 | 0.02346703 |
| CENPM | 1.04351529 | 1.01540711 | 1.07240156 | 0.00223236 |
| BAIAP2L2 | 1.01501054 | 1.00294696 | 1.02721922 | 0.01459251 |
| CHST10 | 1.19332818 | 1.01417325 | 1.40413104 | 0.03321027 |
| INAVA | 1.08938127 | 1.03987315 | 1.14124646 | 0.00030908 |
| MARCKSL1 | 1.00535222 | 1.00238365 | 1.00832957 | 0.00040324 |
