# Supplementary material for: MTFR2-dependent mitochondrial fission promotes HCC progression
Source: J Transl Med. 2024 Jan 18;22:73. doi: 10.1186/s12967-023-04845-6 (PMC10795309; doi:10.1186/s12967-023-04845-6)
Supplement: Supplementary file 2 — Additional file 2: Table S1. The symbols of mitochondrial dynamical genes. Table S2. The differentially expressed genes in the two clusters (|log2FC| > 1 and false discovery rate (FDR) < 0.001). Table S3. The differentially expressed genes in the two clusters (|log2FC| > 0.7 and false discovery rate (FDR) < 0.001). Table S4. The genes selected from the differentially expressed genes of the two clusters by univariate Cox regression analysis (P < 0.05). Table S5. The 11 topological analysis method results calculated by CytoHubba. [file 12967_2023_4845_MOESM2_ESM.zip › Supplementary tables/Table S5.docx]

Table S5. The 11 topological analysis method results calculated by CytoHubba.

| node_name | MCC | DMNC | MNC | Degree | EPC | BottleNeck | EcCentricity | Closeness | Radiality | Betweenness | Stress | ClusteringCoefficient |
| --- | --- | --- | --- | --- | --- | --- | --- | --- | --- | --- | --- | --- |
| MTFR2 | 6 | 0 | 1 | 6 | 7.833 | 10 | 0.5 | 8.5 | 4 | 59 | 120 | 0 |
| G6PD | 1 | 0 | 1 | 1 | 4.661 | 1 | 0.33333 | 5.16667 | 3.09091 | 0 | 0 | 0 |
| MFN1 | 4 | 0 | 1 | 4 | 7.499 | 1 | 0.25 | 6.91667 | 3.45455 | 7 | 26 | 0 |
| KPNA2 | 3 | 0 | 1 | 3 | 7.22 | 3 | 0.33333 | 6.5 | 3.45455 | 4.5 | 18 | 0 |
| DNM1L | 4 | 0 | 1 | 4 | 7.559 | 1 | 0.25 | 6.91667 | 3.45455 | 7 | 26 | 0 |
| MIEF1 | 1 | 0 | 1 | 1 | 3.084 | 1 | 0.25 | 4.16667 | 2.36364 | 0 | 0 | 0 |
| MEX3A | 2 | 0 | 1 | 2 | 4.985 | 2 | 0.33333 | 5.83333 | 3.27273 | 20 | 34 | 0 |
| EZH2 | 5 | 0 | 1 | 5 | 7.558 | 3 | 0.33333 | 7.83333 | 3.81818 | 32.5 | 76 | 0 |
| ARMC10 | 1 | 0 | 1 | 1 | 4.344 | 1 | 0.25 | 4.91667 | 2.90909 | 0 | 0 | 0 |
| MFF | 2 | 0 | 1 | 2 | 6.319 | 1 | 0.25 | 5.58333 | 3.09091 | 1 | 4 | 0 |
| CDCA8 | 3 | 0 | 1 | 3 | 7.152 | 1 | 0.33333 | 6.5 | 3.45455 | 4.5 | 18 | 0 |
| KIF20A | 4 | 0 | 1 | 4 | 7.502 | 1 | 0.33333 | 7.16667 | 3.63636 | 12.5 | 42 | 0 |
